# Risk Factors for Hearing Loss Are Comparable in Preterm Versus Term Children: A Systematic Review

**DOI:** 10.1111/apa.70222

**Published:** 2025-07-15

**Authors:** Pauline Roehrs, Anna‐Katharina Rohlfs, Reinhard Vonthein, Camilla Simon, Juliane Spiegler

**Affiliations:** ^1^ Department of Paediatrics University Hospital of Luebeck Luebeck Germany; ^2^ Department of Otorhinolaryngology, Head and Neck Surgery Section of Phoniatrics and Paedaudiology, University Hospital of Ulm Ulm Germany; ^3^ Institute of Medical Biometry and Statistics University of Luebeck Luebeck Germany; ^4^ University of Wuerzburg Wuerzburg Germany; ^5^ Department of Paediatrics University Hospital of Wuerzburg Wuerzburg Germany

**Keywords:** hearing loss, mechanical ventilation, ototoxic medication, preterm children, risk factors

## Abstract

**Aim:**

Hearing loss occurs more frequently in preterm children. However, the influence of prematurity itself is unclear. We examined whether risk factors for hearing loss differ between preterm and term infants.

**Methods:**

We conducted a systematic search of three databases in March 2023 for studies comparing risk factors for hearing loss in preterm and term children. Studies on postnatal trauma, chemotherapy or infections after the age of 5 years were excluded. Risk of bias was assessed. Data were extracted and analyzed using logistic regression to yield odds ratios (95% confidence interval).

**Results:**

Of 10 300 studies screened, 16 met the inclusion criteria, including 9059 preterm and 10 048 term children. Only one study compared risk factors between preterm and term infants as primary outcomes. It identified an increased risk in the preterm but not term group with mechanical ventilation exceeding 5 days, sepsis, and ototoxic medication. No significant differences were found when both groups shared these risk factors, suggesting that prematurity may not be an independent risk factor. Other studies reported variable results.

**Conclusion:**

Evidence of different effect sizes of risk factors in preterm and term children remains inconclusive. Preterm children acquire more risk factors in the neonatal period.

**Abbreviations**
95% CI: 95% confidence intervals
CMV: cytomegalovirus
GRADE: Grading of Recommendations, Assessment, Development and Evaluation
HR: hazard ratio
JCIH: Joint Committee on Infant Hearing
NICU: neonatal intensive care unit
OR: odds ratio
PRISMA: Preferred Reporting Items for Systematic Reviews and Meta‐Analysis
PROSPERO: Prospective Register of Systematic Reviews

**Summary**

- Few studies have compared risk factors for hearing loss in preterm and term children.
- These few studies described a comparable effect of ototoxic medication, infection or prolonged ventilation in preterm and term children on the frequency of hearing loss.
- Preterm children are more frequently exposed to these ototoxic risk factors, which might explain the increased rate of hearing loss seen in preterm populations.

## 1 Introduction

The ability to perceive and process acoustic stimuli is one important way humans interact with their environment. In childhood, hearing is particularly important as it forms the basis for auditory processing and language development, cognition and social interaction. Due to their very immature biological systems, preterm infants are at an increased risk for a variety of long‐term health complications, including the development of hearing loss during childhood [1]. A large Norwegian population‐based study found that the adjusted risk of sensorineural hearing loss is 3.3 times higher for preterm infants born before 32 weeks of gestation and 7.6 times higher for those born before 28 weeks of gestation compared to term‐born infants [2]. Since preterm birth ranged from 4.4%–10.0% in Europe in 2019 [3], the prevention of long‐term health sequelae of prematurity, including the prevention of auditory loss, is of utmost importance. As outlined by the Joint Committee on Infant Hearing (JCIH), a leading authority in this field, hearing loss in infants can arise from a variety of causes [4, 5]. These include genetic factors, infections, and complications during the perinatal period [4]. The prevalence of congenital hearing loss is approximately 1.5 per 1000 newborns globally, with regional variations reported, such as 0.13% in the UK, 0.19% in the USA, 0.12% in Korea, and 0.16% in Japan [6, 7]. Notably, the condition is more prevalent among preterm infants or those admitted to neonatal intensive care units (NICUs). Among NICU graduates, hearing loss has been diagnosed in 1.2% to 11.0% of cases, depending on the gestational age at birth [7]. In a German cohort study, hearing loss or the use of hearing aids was approximately twice as common in preterm as in term children [1]. However, prematurity has not been considered an isolated risk factor since 2000 [5]. The aim of this systematic review was to synthesize the current knowledge on risk factors for hearing loss. The outcome hearing loss was defined by pathological audiometric assessment or medically recorded impairments in preterm compared to term children from birth to 18 years of age. We focused exclusively on studies that allowed a direct comparison between preterm and term infants. Given the importance of early detection and intervention in mitigating the long‐term effects of hearing loss, this review aims to address gaps in the current international literature and to propose future research directions. This review investigates potential differences in risk factors for hearing loss between preterm and term infants.

## 2 Methods

The systematic review was registered in the International Prospective Register of Systematic Reviews (PROSPERO), Registration number: CRD42022364734 and follows the recommendations of the Preferred Reporting Items for Systematic Reviews and Meta‐Analysis (PRISMA) [8]. The risk factors initially specified in the protocol were broadened to include postnatal therapies, interventions and complications of prematurity as risk factors for hearing loss.

### 2.1 Search Strategy

A literature search was conducted on 12 October 2022 and was repeated on 20 March 2024. To identify relevant studies, the electronic databases MEDLINE, Web of Science, Cochrane Library and PROSPERO study register were searched, and the reference lists of secondary literature and included studies were checked. No restrictions were used for publication date or language. The search strategy was developed using search terms as described in Appendix S1.

### 2.2 Inclusion and Exclusion Criteria

We included studies comparing risk factors for hearing loss in preterm and term children from birth to 18 years of age. Preterm birth was defined as birth before the completion of 37 weeks of gestation; term was defined as having completed at least 37 weeks of gestation. We were interested in preterm‐specific risk factors like perinatal or postnatal complications or the frequent recurrent respiratory infections in childhood. Therefore, studies in which the risk factors for hearing loss in childhood were postnatal trauma or chemotherapy were excluded due to established causality. Furthermore, studies identifying childhood infections after the age of 5 years were excluded to focus the evidence base on this field. Review articles, meta‐analyses, study protocols, conference abstracts, and editorials were excluded, but checked for relevant primary literature.

### 2.3 Study Selection

**FIGURE 1:**
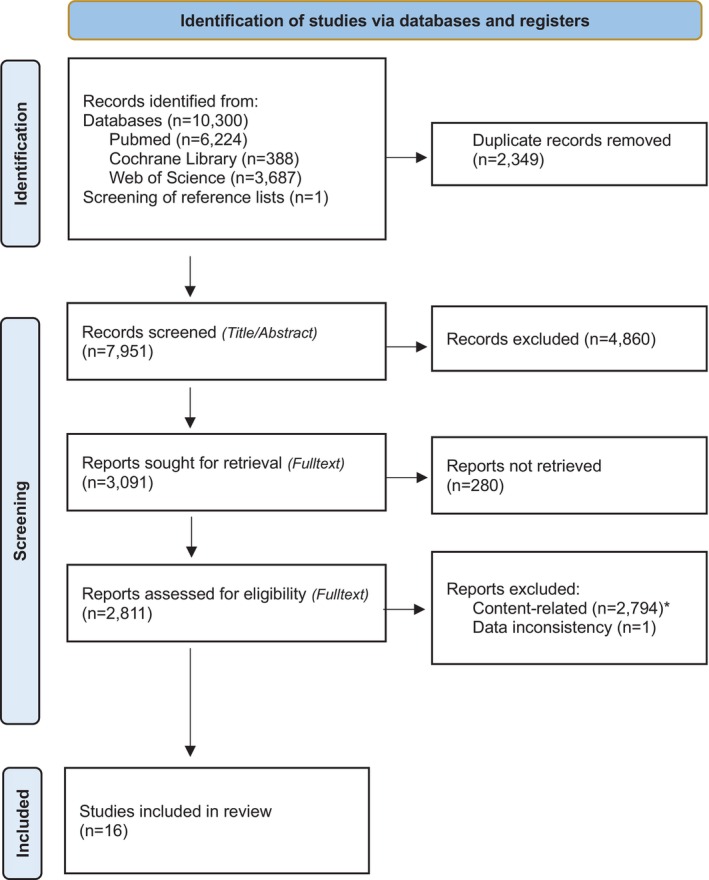
PRISMA 2020 flow diagram for new systematic reviews which included searches of databases and registers [10]. *#* Preferred Reporting Items for Systematic Reviews and Meta‐Analyses. *Detailed reasons for exclusion in Table S2: Characteristics of excluded studies.

The initial search results were imported into Endnote (version 20.4, Clarivate Analytics, Pennsylvania, USA) and duplicates were removed. Any duplicates not identified by the software were afterwards removed manually. Titles and abstracts were screened by two independent authors (P.R. and C.S.) to identify potentially relevant studies. At each stage of the screening, data extraction process, and quality assessment, disagreements about the acceptability of a study were resolved by discussion or consultation of a third author (J.S.). Interrater agreement was high (title: *k* = 0.93, abstract: *k* = 0.95). Full texts of the selected studies were retrieved and checked for eligibility independently by P.R. and C.S. If several reports on a cohort were available, the study with the largest sample size was used. The reasons for exclusion were documented at each stage and justified during the full‐text review. The study selection process is shown in a PRISMA [9] flowchart (Figure 1).

### 2.4 Data Extraction

A customised standard form (Microsoft EXCEL, version 16.77.1, Microsoft Corporation, Washington, USA) was used for the data extraction, collection of study characteristics and outcome data.

### 2.5 Missing Data

In the case of incomplete or missing data that could not be determined from other available data, these were requested from the original authors. If an author did not respond after two requests, the study was excluded due to data inconsistency.

### 2.6 Quality Assessment

Cohort studies were evaluated using the modified Newcastle‐Ottawa Scale [11], while cross‐sectional studies were assessed using the Joanna Briggs Institute Critical Appraisal Checklist for Analytical Cross‐Sectional Studies [12]. Cohort studies were assessed based on selection bias, comparability of study groups, exposure and outcome assessment and loss to follow up. Cross‐sectional studies were evaluated for quality of study groups, exposure and outcome assessment, handling of cofounding factors and the statistical analysis of results. Studies were rated as high quality if they reached at least seven points on the Newcastle‐Ottawa scale or at least 80% ‘Yes’ responses in the Joanna Briggs Institute checklist. A moderate rating was assigned to studies with five and six points on the Newcastle‐Ottawa scale or between 50% and 80% ‘Yes’ responses in the Joanna Briggs Institute checklist. Studies scoring below five points on the Newcastle‐Ottawa Scale or with fewer than 50% ‘Yes’ responses in the Joanna Briggs Institute checklist were rated as low quality.

### 2.7 Identification of Heterogeneity

The clinical diversity of the study populations is described in the overview text and in the study tables (Table 2), which summarise the most important clinical and methodological characteristics.

### 2.8 Statistics

The data are summarised narratively, as the heterogeneity of the included studies was expected to be high and precluded a meta‐analysis, subgroup analysis, and assessment of publication bias. The results were structured according to the study design.

In studies investigating hearing as the primary outcome, the risk factors for hearing loss were determined for preterm and term infants and compared using binomial logistic regression analysis with an interaction term to estimate marginal means from the raw study data. In studies investigating hearing in preterm and term‐born infants in a population with an underlying condition, odds ratios (OR) and risk differences with 95% confidence intervals (95% CI) were estimated by logistic regression from the raw data of the studies. The results were presented through narrative synthesis and were not further analysed if absolute numbers could not be reconstructed. The chi‐square test was used when the expected number of cases per cell was > 5; Fisher's exact test was used when < 5. A *p*‐value < 0.05 was considered statistically significant. The statistical program used was jamovi (Version 2.3.28, The jamovi Project, Sydney, Australia); graphics were created using Graphpad Prism (Version 10.0.0 for Mac OS X, GraphPad Software, Massachusetts, USA). Due to the low number of studies, reporting bias could not be analysed.

## 3 Results

**FIGURE 2:**
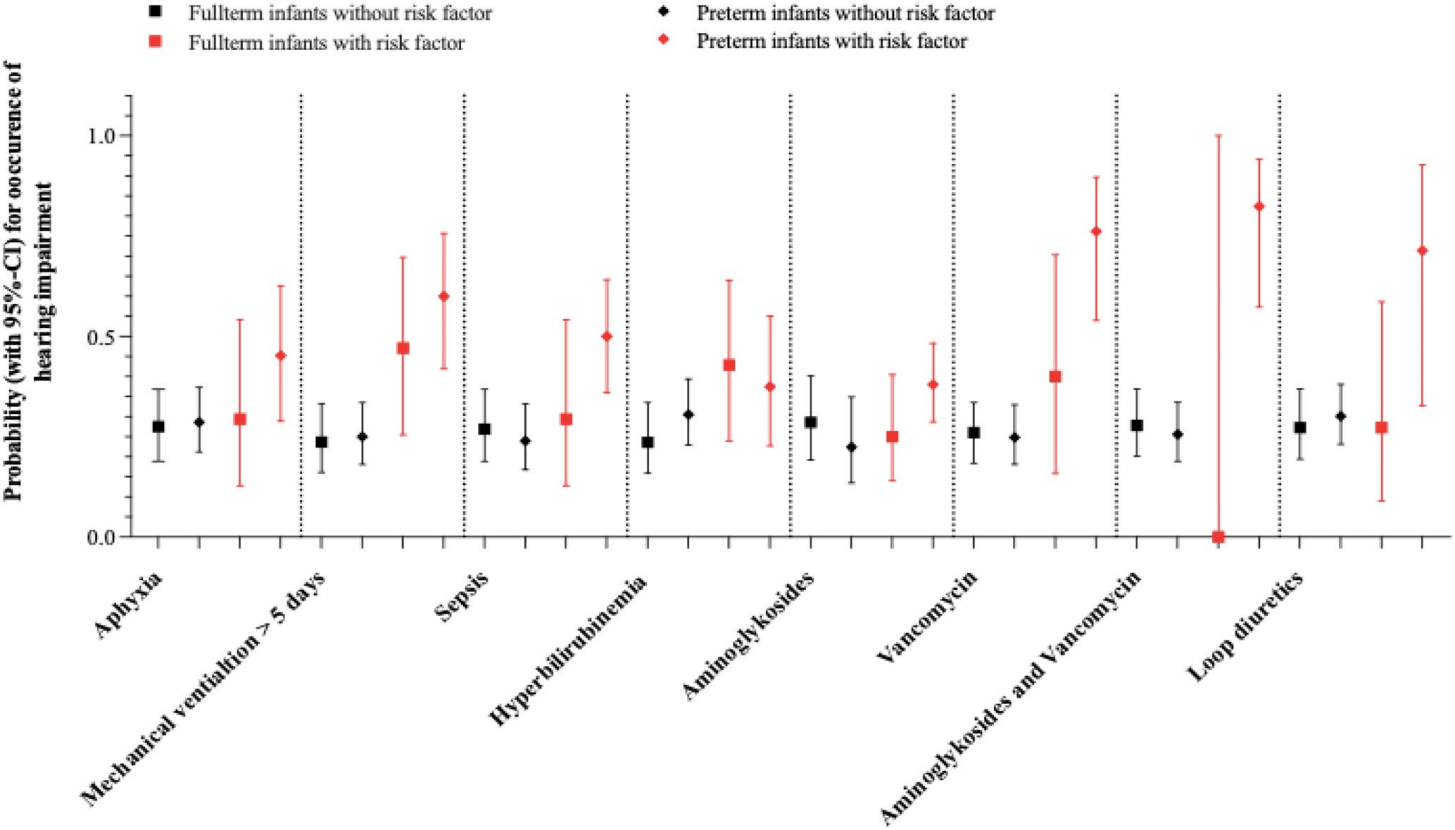
Estimated marginal means for the occurrence of hearing impairment depending on the presence of risk factors comparing preterm to term infants (Analysis of data examined by Khairy et al. [28]).

**FIGURE 3:**
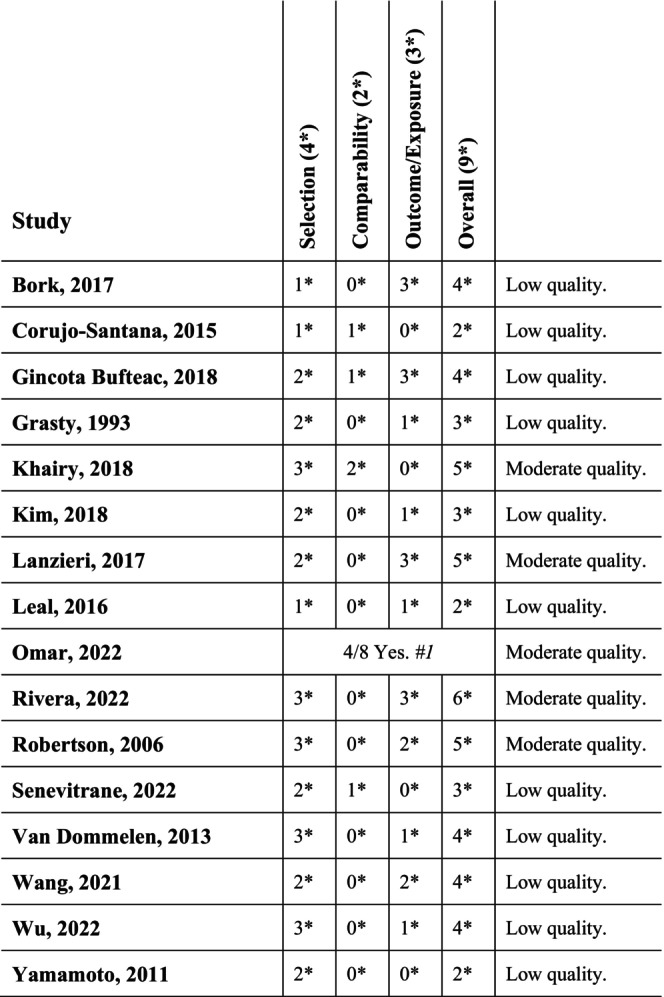
Quality assessment of included studies. * Assessed by modified Newcastle‐Ottawa Scale. Grading: < 7 High quality; 5–7 Moderate quality; > 5 Low quality. ^#^1 Only study assessed by *Joanne Briggs Institute Critical appraisal checklist for analytical cross‐sectional studies*. Grading: > 80% Yes as high quality; 50%–80% Yes as moderate quality; < 50% Yes as low quality.

The PRISMA flowchart summarising the search results is presented in Figure 1. A total of 10 300 studies were identified. One additional study was retrieved after screening reference lists. Ultimately, 15 cohort studies and one prospective cross‐sectional study examining factors that might increase the risk of hearing loss in a total of 9059 preterm versus 10 048 term children were included in the analysis. The distribution of the gestational age of 76 infants was not clearly reported. Table 1 summarises the findings on the association between influencing factors and hearing loss using the Grading of Recommendations, Assessment, Development and Evaluation (GRADE) criteria. An overview of the characteristics of the included studies is presented in Table 2 with further details available in Appendix S2. The extracted data from included studies are summarised in Tables 1, 2, 3, 4 and Figure 2. Table 3, Figure 2 and Table S1 present an analysis of data from Khairy et al. [28], the only study directly comparing hearing loss as a primary outcome between preterm and term‐born infants. Table 4 highlights studies investigating differences in hearing outcomes between preterm and term infants under various underlying conditions as a secondary outcome [13, 15, 16, 17, 18, 20, 21, 22, 24, 25, 26, 27]. Three studies [14, 19, 23] were excluded from further analysis and are only presented as part of the narrative synthesis in the text due to missing absolute numbers. None of the included studies were rated as high quality, five studies as moderate quality, and 11 studies as low quality. A detailed risk of bias assessment is shown in Figure 3, with additional information provided in Appendix S2. To describe the available evidence, studies of low quality were not excluded but were rated and labelled as such (Tables 2, 3, 4 and Figure 3). The overall quality of evidence was assessed by the authors P.R. and J.S. using GRADE criteria and is summarised in Table 1. The clinical diversity of the study populations was high and described in the overview text and in the study tables (Table 2). A list of excluded studies, along with the corresponding reasons, is provided in Table S2.

**TABLE 1:** Summary of findings.

Risk factor	Study results	Certainty of evidence	Comment
*Perinatal infections*			
Congenital CMV infection	OR ranging from 0.000 (0.00‐NaN) to 0.615 (0.323–1.17) Data from 478 participants (*n* = 128 preterm, *n* = 350 term) in five studies [13, 16, 17, 18, 20]. HR ranging from 1.0 (0.5–1.7) to 2.09 (1.29–3.39) Data from 5969 participants (*n* = 744 preterm, *n* = 5149 term)^f^ in 2 studies [14, 19]. Time of infection not specified as pre‐ or postnatal.	Very low^b^, ^c^, ^d^	No conclusion can be drawn whether CMV infection is associated with a further increased or decreased risk for hearing impairment in preterm born compared to term born.
Congenital ZIKA virus infection (and microcephaly)	OR of 0.551 (0.0278–10.90) Data from 64 participants (*n* = 8 preterm, *n* = 56 term) in one study [15].	Very low^a^, ^c^, ^d^, ^e^	No conclusion can be drawn whether ZIKA virus infection is associated with a further increased or decreased risk for hearing impairment in preterm born compared to term born.
Sepsis	OR of 2.400 (0.7282–7.91) Data from 260 participants (*n* = 150 preterm, *n* = 110 term) in one study [28].	Very low^c^, ^e^	No conclusion can be drawn whether sepsis is associated with a further increased or decreased risk for hearing impairment in preterm born compared to term born.
*Drug exposure*			
Aminoglycosides	OR of 1.842 (0.803–4.226) Data from 260 participants (*n* = 150 preterm, *n* = 110 term) in one study [28].	Very low^c^, ^e^	No conclusion can be drawn whether aminoglycosides are associated with a further increased or decreased risk for hearing impairment in preterm born compared to term born.
Vancomycin	OR of 4.800 (0.9544–24.14) Data from 260 participants (*n* = 150 preterm, *n* = 110 term) in one study [28].	Very low^c,e^	No conclusion can be drawn whether vancomycin is associated with a further increased or decreased risk for hearing impairment in preterm born compared to term born.
Loop diuretics	OR of 6.667 (0.8087–54.96) Data from 260 participants (*n* = 150 preterm, *n* = 110 term) in one study [28].	Very low^c,e^	No conclusion can be drawn whether loop diuretics are associated with a further increased or decreased risk for hearing impairment in preterm born compared to term born.
*Neonatal treatment/complications*			
Perinatal asphyxia	OR of 1.976 (0.560–6.97) Data from 260 participants (*n* = 150 preterm, *n* = 110 term) in one study [28].	Very low^c^, ^e^	No conclusion can be drawn whether perinatal asphyxia is associated with a further increased or decreased risk for hearing impairment in preterm born compared to term born.
NICU stay	OR varying from 0.678 (0.507–0.906) to 18.197 (1.199–276.128) Data from 11 018 participants (*n* = 7778 preterm, *n* = 3240 term) in two studies [23, 25]. Substantial heterogeneity in effect estimates between studies and lack of detailed information regarding reasons for NICU stay among term controls.	Very low^b^, ^d^, ^e^	No conclusion can be drawn whether NICU stay is associated with a further increased or decreased risk for hearing impairment in preterm born compared to term born.
Ventilation > 5 days	OR of 1.687 (0.508–5.60) Data from 260 participants (*n* = 150 preterm, *n* = 110 term) in one study [28].	Very low^c^, ^e^	No conclusion can be drawn whether ventilation > 5 days is associated with a further increased or decreased risk for hearing impairment in preterm born compared to term born.
Neonatal respiratory failure	OR of 2.58 (0.735–9.03) Data from 81 participants (*n* = 14 preterm, *n* = 67 term) in one study [24].	Very low^c^, ^d^, ^e^	No conclusion can be drawn whether neonatal respiratory failure is associated with a further increased or decreased risk for hearing impairment in preterm born compared to term born.
Cerebral palsy	OR of 0.270 (0.0334–2.19) Data from 185 participants (*n* = 52 preterm, *n* = 133 term) in one study [22].	Very low^a^, ^c^, ^d^, ^e^	No conclusion can be drawn whether cerebral palsy is associated with a further increased or decreased risk for hearing impairment in preterm born compared to term born.
Hyperbilirubinemia	OR varying from 0.800 (0.260–2.46) to 1.09 (0.443–2.69) Data from 352 participants (*n* = 212 preterm, *n* = 140 term) in two studies [21, 28]. Heterogeneity in effect estimates across studies and lack of a clear definition of hyperbilirubinemia in one study.	Very low^b^, ^c^, ^d^, ^e^	No conclusion can be drawn whether Hyperbilirubinemia is associated with a further increased or decreased risk for hearing impairment in preterm born compared to term born.
*Interventions*			
Complex cardiac surgery	OR varying from 2.07 (0.921–4.67) to 2.68 (1.39–5.17) Data from 1036 participants (*n* = 123 preterm, *n* = 913 term) in two studies [26, 27]. Studies with small sample sizes for preterm infants and indirect results. Outcome measurements and age at hearing assessment differed between studies.	Very low^a^, ^d^, ^e^	No conclusion can be drawn whether complex cardiac surgery is associated with a further increased or decreased risk for hearing impairment in preterm born compared to term born.
*Socioeconomic factors*			
No studies identified.			

Abbreviation: NaN, not a number (missing, undefined or unrepresentable values in numerical datasets).

^a^ Downgraded by one level for risk of bias (≥ 80% of studies at moderate quality or less for risk of bias).

^b^ Downgraded by one level for inconsistency (heterogeneity in effect estimates reported by studies).

^c^ Downgraded by one level for imprecision (wide 95% CIs, includes benefit and potential harm).

^d^ Downgraded by one level for indirectness.

^e^ Downgraded by one level for size of study and/or control group < 200 each in one study minimum.

^f^ Distribution of gestational age of 76 infants was not further stated.

**TABLE 2:** Characteristics of included studies.

Study	Risk factors	Study collective	Mean gestational age in weeks (weeks)	Outcome measurement	Age at hearing assessment	Indicated type of hearing loss and stated definition	Geographical region	Study type	Study period	Study quality^b^
*Perinatal infections*										
Kim 2018 [13]	Congenital CMV infection	Preterm infants (*n* = 18)	Not stated < 37 weeks	Review of medical records (including ABR, AABR, PTA, OAE, DPOAE and TEOAE)	1–145 months (0–12 years).	*SNHL*; hearing threshold > 25 dB in audiologic assessment	Chungnam/Seoul, South Korea	Cohort study	2005–2016	Low
		Term infants (*n* = 9)	Not stated ≥ 37 weeks							
Lanzieri 2017 [14]	Symptomatic cCMV infection	Absolute distribution of gestational age of study groups was not specified. 76 infants with cCMV (*n* = 56 infants with SNHL and *n* = 20 infants without SNHL) were analysed		Click and tone‐burst auditory brainstem response, behavioural audiometry, from 0.25–8 kHz and tympanometry	Case patients were followed with hearing evaluations during infancy, preschool, elementary/middle/and high school years	*SNHL*; defined as hearing level ⩾ 25 dB for ABR or at any frequency for the TB or PTA, in the absence of middle ear disorder	Houston, Texas, USA	Cohort study	1983–2005	Moderate
Leal 2016 [15]	Microcephaly and congenital ZIKA virus infection	Preterm infants (*n* = 8)	Not stated < 37 weeks	Auditory brainstem response (ABR)	0–10 months, mean time to follow‐up is 97 days (3 months).	*SNHL*; defined as hearing thresholds > 25 dB	Recife, Pernambuco, Brazil	Cohort study	2015–2016	Low
		Term infants (*n* = 56)	Not stated ≥ 37 weeks							
Rivera 2002 [16]	Symptomatic congenital CMV infection	Preterm infants (*n* = 55)	Not stated < 37 weeks	Auditory brainstem response audiometry (ABR) or behavioural audiometric evaluations appro‐ priate for child's developmental level	Newborn period, than every 6 months until age of 2 years, annual follow‐up thereafter.	*SNHL*; defined as air conduction thresholds in ABR > 25 dB; or > 20 dB on behavioural audiometric evaluations (while normal bone conduction threshold and middle ear function)	Birmingham, Alabama	Cohort study	1966–1997	Moderate
		Term infants (*n* = 125)	Not stated ≥ 37 weeks							
Seneviratne 2022 [17]	cCMV infection	Preterm infants (*n* = 26)	< 32 weeks (*N* = 19) 32–37 weeks (*N* = 7)	Examination of audiometry reports (including TEOAE, ABR and tympanometry)	24 months (2 years)	*SNHL*; definition not further specified	Townsville City, Australien	Cohort study	2005–2020	Low
		Term infants (*n* = 19)	Not stated ≥ 37 weeks							
Wang 2021 [18]	cCMV infection	Preterm infants (*n* = 4)	Not stated < 37 weeks	ABR (hearing screening and children < 3 years); DPOAEs/TOAEs (children > 3 years) and tympanometry	Anually, up to 4 years	*Hearing loss*; defined as ABR threshold > 20 dB with broad‐band click stimuli, > 40 dB at 500 Hz or 30 dB at 1000, 2000 or 4000 Hz with frequency specific tone pip stimuli; behavioural threshold > 20 dB in any frequency	Shandong, China	Cohort study	2011–2018	Low
		Term infants (*n* = 137)	Not stated ≥ 37 weeks							
Wu 2022 [19]	cCMV infection (< 3 months)	Preterm infants (*n* = 744)	< 32 weeks (*N* = 693) 32–37 weeks (*N* = 7491)	Examination of medical records (ABR or OAE test, SNHL related ICD‐9/ICD‐10 code) using National Health Insurance Research Database in Taiwan	Follow‐up at 3–10 years	*SNHL*; defined as receipt of a diagnosis according to the ICD‐9/10 diagnostic codes; arrangement of ABR or OAE before the presence of SNHL‐related diagnostic codes; diagnosis made by an otorhinolaryngologist	Taiwan	Cohort study	2005–2009	Low
		Term infants (*n* = 5149)	37–41 weeks (*N* = 55 077) > 41 weeks (*N* = 1562)							
Yamamoto 2011 [20]	cCMV infection	Preterm infants (*n* = 25)	Not stated < 37 weeks	ABR (at least 2 within the first year of life and follow‐ups prior to the age of 3); pure tone conditioned play audiometry (follow‐ups after the age of 3 years)	At median age of 12 months, range 15 days–51 months (for initial ABR testing); at median age of 47 months, range 12–48 months (for latest hearing evaluation).	*SNHL*; air conduction thresholds > 30 dB in at least two ABR evaluations on two different occasions	São Paulo, Brazil	Cohort study	2003–2009	Low
		Term infants (*n* = 60)	Not stated ≥ 37 weeks							
*Neonatal treatment/complications*										
Corujo‐Santana 2014 [21]	Hyperbilirubinemia (TBB > 5 mg/dL)	Preterm infants (*n* = 62)	Not stated < 37 weeks	OAEs and EAPBs	First 48 h of life (OAEs); EAPBs following for diagnosis or follow‐up	*Hearing loss*; definition not further specified	Gran Canaria, Spain	Cohort study	2007–2011	Low
		Term infants (*n* = 30)	Not stated ≥ 37 weeks							
Gincota Bufteac 2018 [22]	Cerebral palsy	Preterm infants (*n* = 52)	< 28 weeks (*N* = 10) 28‐31 weeks (*N* = 12) 32–36 weeks (*N* = 30)	Examination of medical records	7–8 years	*Hearing impairment*; definition not further specified	Chisinau, Moldova	Cohort study	2016	Low
		Term infants (*n* = 133)	Not stated ≥ 37 weeks							
Omar 2022 [23]	NICU stay	Preterm infants (*n* = 123)	Not stated < 37 weeks	Hearing screening by TEOAE; diagnostic by ABR	Neonatal period (TEOAEs) and within 3 months (diagnostic ABR)	*Hearing impairment*; definition not further specified	Assiut, Egypt	Cross‐sectional study	2020–2021	Moderate
		Term infants (*n* = 77)	Not stated ≥ 37 weeks							
Robertson 2006 [24]	Neonatal respiratory failure	Preterm infants (*n* = 14)	Not stated < 37 weeks	Bilateral pure‐tone responses (within speech range 250–4000 Hz) bone conduction, and tympanometry	At 18–24 months (outcome measurement not further described) and at 4 years of age	*SNHL*; definition not further specified	Canada	Cohort study	1994–1996	Moderate
		Term infants (*n* = 67)	Not stated ≥ 37 weeks							
van Dommelen 2010 [25]	NICU stay	Preterm infants (*n* = 7655)	< 32 weeks (*N* = 4429) > 32/< 37 weeks (*N* = 3226)	Examination of medical records (AABR and ABR from matched database)	Neonatal period, newborns before discharge from NICU	*Hearing loss*; defined as ABR levels > 35 dB in at least one ear	Netherlands	Cohort study	2002–2005	Low
		Term infants (*n* = 3163)	Not stated ≥ 37 weeks							
*Interventions*										
Bork 2017 [26]	CCS with cardiopulmonary bypass at ≤ 6 weeks	Preterm infants (*n* = 76)	Not stated < 37 weeks	Visually reinforced audiometry (first visit); ABR (diagnostic or first method not possible); conditioned play audiometry (older infants)	6–8 months and follow‐up at age 2 years	*Permanent hearing loss*; defined as CHL or SNHL with responses of more than 25 dB at either ear in any of the frequencies of 500–4000 Hz	Edmonton, Alberta	Cohort study	1996–2015	Low
		Term infants (*n* = 615)	Not stated ≥ 37 weeks							
Grasty 2018 [27]	Survival of surgical treatment of CHD with CPB	Preterm infants (*n* = 47)	Not stated < 37 weeks	Standard paediatric assessment methods based on developmental ability	4 years	*Hearing loss*; defined as average PTA thresholds ≥ 20 dB at frequencies (500, 1000, 2000) or ≥ 25 dB at 2 or more frequencies above 2000 Hz. Further classified in SNHL and CHL but only stated as hearing loss	Philadelphia, USA	Cohort study	1998–2008	Low
		Term infants (*n* = 298)	Not stated ≥ 37 weeks							
*Multiple risk factors*										
Khairy 2017 [28]	Multiple risk factors^a^	Preterm infants (*n* = 150)	32.7 weeks (26–36 weeks)	TEOAE and AABR	Neonatal period, newborns after discharge from NICU	*Hearing loss*; defined as pathological AABR	Kairo, Egypt	Cohort study	2013–2014	Moderate
		Term infants (*n* = 110)	38.5 weeks (38–40 weeks)							

Abbreviations: CCS, complex cardiac surgery; CHD, congenital heart disease; CHL, conductive hearing loss; CPB, cardiopulmonary bypass; PTA, pure‐tone audiometry; SNHL, sensorineural hearing loss.

^a^ Perinatal asphyxia, mechanical ventilation > 5 days, sepsis, hyperbilirubinaemia, use of aminoglycosides and/or vancomycin, loop diuretics.

^b^ Study quality assessed with modified Newcastle‐Ottawa Scale or Joanne Briggs Institute Critical Appraisal Checklist for Analytical Cross‐Sectional Studies; further and more detailed information is in Figure 3 or Appendix S2.

**TABLE 3:** Risk factors for hearing impairment in preterm and term born infants (data analysis of Khairy et al. [28]).

Risk factors for hearing impairment	Hearing impairment		No hearing impairment		OR 1 (95% CI)^a^	OR 2 (95% CI)^b^	OR 3 (95% CI)^c^	Study quality^e^
	Preterm (*n* = 48)		Term (*n* = 30)		Preterm (*n* = 102)	Term (*n* = 80)		
*Neonatal treatment/complications*								
Perinatal asphyxia	14/48 (29.2%)	5/30 (16.7%)	17/102 (16.7%)	12/80 (15.0%)	2.059 (0.915–4.635)	1.133 (0.363–3.542)	1.976 (0.560–6.97)	
Mechanical ventilation > 5 days	18/48 (37.5%)	8/30 (26.7%)	12/102 (11.8%)	9/80 (11.3%)	4.500. (1.944–10.415)	2.869 (0.988–8.327)	1.687 (0.508–5.60)	
Sepsis	23/48 (47.9%)	5/30 (16.7%)	23/102 (22.5%)	12/80 (15.0%)	3.160 (1.5193–6.573)	1.13 (0.363–3.542)	2.400 (0.7282–7.91)	
Hyperbilirubinaemia	12/48 (25.0%)	9/30 (30.0%)	20/102 (19.6%)	12/80 (15.0%)	1.367 (0.604–3.090)	2.429 (0.900–6.557)	0.800 (0.260–2.46)	
*Medication*								
Use of aminoglycosides	35/48 (72.9%)	10/30 (33.3%)	57/102 (55.9%)	30/80 (37.5%)	2.126 (1.007–4.486)	0.833 (0.344–2.017)	1.842 (0.803–4.226)	
Use of vancomycin	16/48 (33.3%)	4/30 (13.3%)	5/102 (4.9%)	6/80 (7.5%)	9.7 (3.2916–28.585)	1.897 (0.496–7.259)	4.800 (0.9544–24.14)	
Use of vancomycin with aminoglycoside	14/48 (29.2%)	0/30 (0.0%)	3/102 (2.9%)	2/80 (2.5%)	13.588 (3.680–50.179)	—^d^	0.00 (0.00‐inf.)	
Use of loop diuretics	5/48 (10.4%)	3/30 (10.0%)	2/102 (2.0%)	8/80 (10.0%)	5.814 (1.0854–31.143)	1.00 (0.247–4.050)	6.667 (0.8087–54.96)	

^a^ Preterm infants with risk factors to preterm infants without risk factors.

^b^ Full‐term infants with risk factor to full‐term infants without risk factor.

^c^ Preterm infants with risk factor to full‐term infants with risk factor.

^d^ Could not be calculated because of ample size *N* = 0.

^e^ Assessed with modified Newcastle‐Ottawa Scale.

**TABLE 4:** Analysis of differences in occurrence of hearing impairment comparing preterm to term born infants with underlying disease.

Study	Risk factor	Study collective	Hearing loss	No hearing loss	OR (95% CI)	RD (95% CI)	*p*	Study quality^#^
*Perinatal infections*								
Leal 2016 [15]	Microcephaly and congenital ZIKA virus infection	Preterm infants (*n* = 8)	0/8 (=0%)	8/8 (=100%)	0.551 (0.0278–10.9)	−0.0893 (−0.164 to 0.0146)	1.000	Low
		Term infants (*n* = 56)	5/56 (=8.9%)	51/56 (=91.1%)				
Kim 2018 [13]	Congenital CMV infection	Preterm infants (*n* = 18)	02/18 (=11.11%)	16/18 (=88.89%)	0.0625 (0.00829–0.471)	−0.556 (−0.896 to −0.215)	0.006	Low
		Term infants (*n* = 9)	6/9 (=66.67%)	3/9 (=33.33%)				
Rivera 2002 [16]	Symptomatic congenital CMV infection	Preterm infants (*n* = 55)	22/55 (=40.0%)	33/55 (=60.0%)	0.615 (0.323–1.17)	−0.120 (−0.276 to 0.0363)	0.138	Moderate
		Term infants (*n* = 125)	65/125 (=52.0%)	60/125 (=48.0%)				
Seneviratne 2022 [17]	Congenital CMV infection	Preterm infants (*n* = 26)	3/26 (=11.5%)	23/26 (=88.5%)	0.365 (0.0754–1.77)	−0.148 (−0.381 to 0.0852)	0.253	Low
		Term infants (*n* = 19)	5/19 (=26.3%)	14/19 (73.7%)				
Wang 2021 [18]	cCMV infection	Preterm infants (*n* = 4)	0/4 (=0%)	4/4 (=100%)	0.000 (0.000‐NaN)	−0.0292 (−0.0574 to −0.00101)	1.000	Low
		Term infants (*n* = 137)	4/137 (=2.9%)	133/137 (=97.1%)				
Yamamoto, 2011 [20]	cCMV infection	Preterm infants (*n* = 25)	2/25 (=8.0%)	23/25 (=92.0%)	0.565 (0.111–2.87)	−0.0533 (−0.190 to 0.0834)	0.716	Low
		Term infants (*n* = 60)	8/60 (=13.33%)	52/60 (=86.67%)				
*Neonatal treatment/complications*								
Corujo‐Santana 2014 [21]	Hyperbilirubinemia (TBB > 5 mg/dL)	Preterm infants (*n* = 62)	24/62 (38.7%)	38/62 (73.1%)	1.09 (0.443–2.69)	0.0204 (−0.190 to 0.231)	0.850	Low
		Term infants (*n* = 30)	11/30 (36.7%)	19/30 (63.3%)				
Gincota Bufteac 2018 [22]	Cerebral palsy	Preterm infants (*n* = 52)	1/52 (=1.9%)	51/52 (=98.1%)	0.270 (0.0334–2.19)	−0.0484 (−0.105 to 0.00827)	0.287	Low
		Term infants (*n* = 133)	9/133 (=6.8%)	124/133 (=93.2%)				
Robertson, 2006 [24]	Neonatal respiratory failure	Preterm infants (*n* = 14)	10/14 (=71.4%)	4/14 (=28.6%)	2.58 (0.735–9.03)	0.222 (−0.0434 to 0.487)	0.131	Moderate
		Term infants (*n* = 67)	34/67 (=50.7%)	33/67 (=49.3%)				
van Dommelen, 2010 [25]	NICU stay	Preterm infants (*n* = 7655)	124/7655 (=1.6%)	7531/7655 (=98.4%)	0.678 (0.507–0.906)	−0.00751 (−0.0135 to −0.00150)	0.008	Low
		Term infants (*n* = 3163)	75/3163 (=2.4%)	3088/3163 (=97.6%)				
*Interventions*								
Bork, 2017 [26]	CCS with cardiopulmonary bypass at ≤ 6 weeks	Preterm infants (*n* = 76)	8/76 (=10.5%)	68/76 (=89.5%)	2.07 (0.921–4.67)	0.0516 (−0.0197 to 0.123)	0.116	Low
		Term infants (*n* = 615)	33/615 (=5.4%)	582/615 (=94.6%)				
Grasty 2018 [27]	Survival of surgical treatment of CHD with cardiopulmonary bypass	Preterm infants (*n* = 47)	18/47 (=38.3%)	29/47 (=61.7%)	2.68 (1.39–5.17)	0.195 (0.0492 to 0.341)	0.002	Low
		Term infants (*n* = 298)	56/298 (=18.8%)	242/298 (=81.2%)				

*Note:* “^#^”—study quality assessed with Newcastle‐Ottawa Scale or Joanne Briggs Institute Critical Appraisal Checklist for Analytical Cross‐Sectional Studies.

Abbreviations: CCS, complex cardiac surgery; CHD, congenital heart disease; TBB, total blood bilirubin.

### 3.1 Assessment of Hearing Loss

Hearing loss was assessed using objective and subjective methods, as well as through the extraction of diagnoses from medical records. Ten studies used objective hearing tests, including auditory brainstem response, automated auditory brainstem response, distortion product otoacoustic emissions, transiently evoked otoacoustic emissions and tympanometry [14, 15, 16, 18, 20, 21, 23, 24, 26, 28]. Five studies used subjective hearing assessments, such as behavioural audiometric evaluations, visually reinforced audiometry, pure‐tone conditioned‐play behavioural audiometry and bilateral pure tone response testing [14, 16, 20, 24, 26]. Five studies reviewed medical records for evidence of hearing loss [13, 17, 19, 22, 25]. One study conducted a standard paediatric hearing assessment based on the developmental abilities of children, which were inadequately described [27]. The age at which hearing assessments were conducted ranged from the neonatal period [16, 21, 23, 25, 28] to 12 years [13]. Eight studies explicitly defined hearing loss as sensorineural hearing loss [13, 14, 15, 16, 17, 19, 20, 24]. Four studies assessed hearing loss based on clearly defined auditory thresholds [18, 25, 26, 27], while three examined hearing loss without specifying precise auditory thresholds [21, 22, 23]. Finally, one study defined hearing loss as pathological findings in automated auditory brainstem response testing [28]. Since hearing loss was assessed in a heterogeneous manner, at variable ages and with different definitions of hearing loss, it was not possible to conduct a meta‐analysis. Certainty of evidence was very low for all risk factors examined in included studies (Table 1).

### 3.2 Perinatal Infections

Studies reporting on the association between hearing loss and perinatal infections included 6771 infants (*n* = 1030 preterm). The hearing assessments were conducted between birth and 12 years of age, using various objective tests or examinations of medical records (Table 2). A detailed description of each study and their results is provided Tables 2, 3, 4 and Appendix S2.

#### 3.2.1 Cytomegalovirus

Congenital cytomegalovirus (CMV) infection emerged as the most frequently examined risk factor across studies, with seven cohort studies [13, 14, 16, 17, 18, 19, 20], involving a total of 6447 participants (*n* = 872 preterm). The studies investigated the association between congenital CMV infection and hearing loss in preterm compared to term infants. However, the timing of CMV infection was not further specified as prenatal, perinatal or postnatal onset. Nearly all studies reported ORs < 1 for preterm infants [13, 16, 17, 18, 20] indicating a lower risk for hearing loss in preterm compared to term infants. The exception was Wu et al. [19], who found an increased risk of hearing loss in preterm infants with an infection diagnosed within the first 3 months of life (adjusted hazard ratio (HR) = 2.09 (1.29–3.39)) involving 5893 infants (*n* = 744 preterm). Notably, Kim et al. [13] demonstrated a statistically significant decreased risk for preterm infants, analysing 27 participants and achieving a low quality rating. Lanzieri et al. [14] showed no differences in occurrence of hearing loss between preterm and term‐born infants with symptomatic congenital CMV infection (HR = 1.0 (0.5–1.7), *p* = 0.92), examining 76 infants. All studies examined sensorineural hearing loss [13, 14, 16, 17, 18, 19, 20]. Wang et al. [18] specifically investigated late‐onset sensorineural hearing loss and defined it as hearing loss diagnosed after 1 year of age, with clear criteria to rule out conductive hearing loss. The timing of hearing assessment varied across studies, covering a range from birth to 12 years of age, though most studies concentrated on the first years of life.

#### 3.2.2 Zika Virus Infection

One cohort study [15] involving 64 participants (*n* = 8 preterm) with prenatal Zika virus infection and microcephaly reported a decreased risk for hearing loss in preterm compared to term infants. Hearing loss was defined as sensorineural hearing loss with a hearing threshold > 25 dB assessed using auditory brainstem response from birth to 10 months of age. The study quality was classified as low.

#### 3.2.3 Sepsis

One cohort study [28] assessed the association of hearing loss and culture‐proven sepsis directly comparing preterm and term‐born infants. This study included 260 participants (*n* = 150 preterm). Hearing assessment was conducted in the neonatal period after discharge from NICU using transient evoked otoacoustic emissions and automated auditory brainstem response (Table 2). The results indicated an increased risk of hearing loss in preterm infants with sepsis compared to those without, which reached statistical significance. Differences in hearing loss comparing preterm and term infants with sepsis were not significant, suggesting that prematurity does not further increase the risk factor for sepsis for hearing loss (Table 3 and Figure 2).

### 3.3 Drug Exposure

Khairy et al. [28] reported an association between hearing loss and drug exposure, comparing preterm and term‐born infants directly. This cohort study included 260 participants (*n* = 150 preterm) with hearing assessments conducted in the neonatal period after discharge from NICU using transient evoked otoacoustic emissions and automated auditory brainstem response (Table 2). The study was categorised as moderate quality, with a low risk of bias.

#### 3.3.1 Aminoglycosides, Vancomycin and Loop Diuretics

The results indicated an increased risk for hearing loss in preterm infants exposed to potentially ototoxic drugs, reaching statistical significance (Table 3) [28]. Differences in hearing loss comparing preterm and term infants with exposition to aminoglycosides, vancomycin, both or loop diuretics were not significant, suggesting that prematurity alone might not be an additive risk factor for hearing loss (Table 3 and Figure 2).

### 3.4 Neonatal Complications and Treatment

Six studies [21, 22, 23, 24, 25, 28] reported on associations between hearing loss and neonatal complications or treatment, including 11 636 infants (*n* = 8056 preterm). Hearing assessment was conducted at ages ranging from the neonatal period to 8 years, using both subjective and objective hearing tests or examination of medical records (Table 2).

#### 3.4.1 Perinatal Asphyxia, Mechanical Ventilation Exceeding Five Days, Hyperbilirubinemia

Only one study [28] assessed perinatal asphyxia, mechanical ventilation exceeding 5 days, and hyperbilirubinemia as risk factors for hearing loss, comparing preterm and term infants directly. This cohort study comprised 260 participants (*n* = 150 preterm), including individuals with and without the specified risk factors. Hearing loss was defined as a pathological result on automated auditory brainstem response after discharge from NICU. The study was considered to be of moderate quality with a moderate risk of bias. The results indicated an increased risk for hearing loss in preterm infants with mechanical ventilation exceeding 5 days, perinatal asphyxia, and hyperbilirubinemia, but only ventilation exceeding 5 days reached statistical significance. The risk of hearing loss in the presence of these specified neonatal risk factors did not differ significantly between the preterm and term cohorts. This suggests that prematurity alone may not be an additive risk factor for hearing loss (Table 3 and Figure 2).

#### 3.4.2 NICU Stay

One cohort study [25], involving 10 818 infants (*n* = 7655 preterm) reported a reduced risk for hearing loss assessed through automated auditory brainstem response. One cross‐sectional study [23] involving 200 infants reported an increased risk for hearing loss associated with NICU stay assessed through both auditory brainstem response and transient evoked otoacoustic emissions. The increased risk described by Omar et al. [23] was based on two cases of hearing loss in preterm infants compared to none in term infants. Detailed information regarding reasons for the NICU stay among preterm infants and term controls or confounding factors has not been provided. Therefore, a direct comparison between the groups within each study or between both studies was not possible. In addition, different definitions of hearing loss, such as unilateral hearing loss versus auditory brainstem response level > 35 dB, and different assessment methods, such as medical records versus transient evoked otoacoustic emissions and automated auditory brainstem response, made comparisons impossible.

#### 3.4.3 Neonatal Respiratory Failure

One cohort study [24] involving 81 participants (*n* = 14 preterm) suffering from neonatal respiratory failure reported an increased risk for sensorineural hearing loss in preterm compared to term infants. Hearing was assessed using bilateral pure‐tone response at age 18–24 months, but sensorineural hearing loss was not further defined. The study quality was classified as moderate but did not control for other risk factors than the presence of neonatal respiratory failure.

#### 3.4.4 Cerebral Palsy

One cohort study [22] involving 185 participants (*n* = 52 preterm) diagnosed with cerebral palsy showed a decreased risk for hearing loss in preterm compared to term infants. Hearing loss was not further defined and assessed by examination of medical records at ages of 7–8 years. The study quality was classified as low.

### 3.5 Interventions

#### 3.5.1 Complex Cardiac Surgery

Two cohort studies [26, 27] assessed complex cardiac surgery as a risk factor for hearing loss, one study involving 691 infants (*n* = 76 preterm) and the other study involving 345 infants (*n* = 47 preterm). Hearing assessment was conducted at an age ranging from 6 months to 4 years, using subjective hearing tests or a standard paediatric assessment, which was not further defined (Table 2).

Both studies reported an increased risk for hearing loss in preterm infants but showed low study quality and small sample sizes for preterm infants. The outcome measurements between the studies, namely visually reinforced audiometry versus standard paediatric assessment methods, were not comparable. Furthermore, the age at which hearing was assessed differed, with one study assessing hearing at 6–8 months and the other at 4 years.

### 3.6 Socioeconomic Risk Factors

No studies were identified examining socioeconomic risk factors for the development of hearing loss.

## 4 Discussion

Preterm infants exhibited a higher incidence of hearing loss [1], an increased exposure to these risks and a greater accumulation of risk factors [2, 29]. Hypotheses suggested that an immature blood–brain barrier may contribute to this vulnerability [30, 31]. However, the available evidence does not conclusively establish that prematurity itself is an additive risk factor for hearing loss. This suggests that, at present, preterm infants are sufficiently screened within international newborn hearing screening programmes that adhere to the JCIH recommendations. In particular, the JCIH recommends the use of automated auditory brainstem response as the preferred and approved method for hearing screening in this population, alongside appropriate follow‐up protocols.

### 4.1 Risk Factors and Their Impact

A systematic review by Han et al. [32], published in 2024, described 14 risk factors for infant hearing loss. While these included risks commonly seen in preterm born infants such as low birth weight and very low birth weight, NICU stay exceeding 5 days, mechanical ventilation exceeding 5 days, intrauterine infection, sepsis and intraventricular haemorrhage, no association with preterm birth was found. Although the increased risk of hearing loss in preterm infants is well established, only one study included in this systematic review has primarily compared risk factors between preterm and term infants. All other studies analysed risk factors for hearing loss, comparing preterm and term infants as a secondary outcome. None of the analysed risk factors showed an additive risk in preterm children despite the presumed higher vulnerability of the immature blood–brain barrier [30, 31].

### 4.2 Risk Factors by Joint Committee on Infant Hearing

The latest update of the JCIH guidelines [4], published in 2019, highlights perinatal risk factors such as family history of hearing loss, NICU stays exceeding 5 days, hyperbilirubinemia requiring exchange transfusion, aminoglycoside therapy exceeding 5 days, asphyxia or hypoxic–ischaemic encephalopathy, extracorporeal membrane oxygenation, in utero infections like CMV, syphilis, rubella, herpes, toxoplasmosis, maternal Zika virus infection, craniofacial malformations and congenital syndromes. Postnatal risk factors include culture‐positive infections like meningitis, head trauma, chemotherapy and caregiver concern. As our systematic review shows no indication of a higher vulnerability in preterm born infants, we suggest applying these screening recommendations in preterm infants as well.

#### 4.2.1 Family History of Hearing Loss

This risk factor suggests a genetic influence that has not been studied in the preterm group. Depending on the pathophysiology, the risk of hearing loss might be further increased by preterm birth. However, large registries combining clinical and genetic data are needed to answer this question.

#### 4.2.2 NICU Stays Exceeding 5 Days

Infants in NICUs often have more severe comorbidities [33]. In addition, they are exposed to high‐decibel noise, which can disrupt cochlear development and damage hair cells, potentially resulting in sensorineural hearing loss [34]. We did not identify any studies that controlled for other established risk factors for hearing loss, or at least reported their frequencies. For example, none reported noise exposure [35, 36] in the different NICU environments, like incubator or open crib nor mitigation measures such as use of ear muffs. Most very preterm infants in developed countries remain in the NICU for more than 5 days [37], making them eligible for automated auditory brainstem response as part of the newborn hearing screening and a follow up at the age of 9 months according to the JCIH criteria [4]. However, NICU stay duration alone might not be a sufficient risk indicator, as the underlying reasons for admission might vary significantly between preterm and term infants. Preterm infants with an unremarkable neonatal course might stay longer in the NICU due to their immaturity and need for close monitoring, even in the absence of major complications. In contrast, term‐born infants who require NICU admission often do so due to severe medical conditions such as respiratory failure, asphyxia or antibiotic treatment. These factors are associated with an increased risk of hearing loss themselves and should have been controlled for.

Especially in this vulnerable group, survival bias might be a problem. Critically ill preterm infants who die before discharge might be classified as risk‐free in terms of hearing loss. The studies included in our review examining the relationship between NICU stay and hearing outcomes exhibited moderate methodological quality. This was primarily due to the limited comparability between preterm and term infants and the reporting of divergent results, likely attributable to unmeasured variables.

#### 4.2.3 Hyperbilirubinemia (Requiring Exchange Transfusion)

The immaturity of the blood–brain barrier in preterm infants may increase their susceptibility to bilirubin‐related damage in auditory pathways [38]. This is reflected in the lower bilirubin threshold for exchange transfusions in infants of decreasing gestational age. Preterm infants are monitored more frequently for hyperbilirubinemia compared to term infants. Exchange transfusions are rarely necessary in preterm infants. Of the studies included in our review, only Khairy et al. [28] used the JCHI definition [4] of this risk factor, whereas Corujo‐Santana et al. [21] used a threshold of 5 mg/dL, which is far from neurotoxic levels.

#### 4.2.4 Aminoglycosid Therapy Exceeding 5 Days

The use of aminoglycoside antibiotics in the included study was not specifically defined as exceeding 5 days [28]. Therefore, we cannot assess this factor as presented by the JCIH due to inconsistent definitions. The included study did not show an additive risk for hearing loss in preterm infants [28]. Nevertheless, the application of ototoxic medication appears to be a statistically significant risk factor for hearing loss in the preterm cohort. This has already been documented in the literature [39]. Ototoxic medications are frequently used in NICUs and are known to increase the risk of sensorineural hearing loss in preterm infants [13, 28]. However, these findings need to be interpreted with caution. Possible comorbidities are often associated with the use of ototoxic medication. Aminoglycosides can directly damage cochlear hair cells [40], and loop diuretics disrupt cochlear ion gradients, potentially leading to irreversible sensorineural hearing loss [28]. This is particularly concerning when synergistic toxicity amplifies the risk of auditory damage. The implementation of drug monitoring might mitigate these effects and needs to be controlled for in future studies. Methodological limitations, such as a small study sample and the lack of precise definition of hearing thresholds, restrict the generalisability of these findings.

#### 4.2.5 Asphyxia

As the term asphyxia was not clearly defined in the included study [28], we cannot draw any conclusions. The included study did not show a statistically significant increased risk for hearing loss in preterm infants with asphyxia [28]. This result contrasts with the broader literature linking asphyxia to sensorineural hearing loss and auditory neuropathy [41, 42]. For better comparability, the definition of asphyxia should include clinical and metabolic parameters [43]. Comorbidities can act as confounders, requiring caution in interpretation. Confounders were only partially controlled for in this study [28].

#### 4.2.6 Extracorporeal Membrane Oxygenation

Extracorporeal membrane oxygenation is only relevant for more mature preterm infants whose gestational age and weight allow for catheter placement. This risk factor was not analysed in the included studies. Two studies examined complex cardiac surgery, involving cardiopulmonary bypass [26, 27]. These studies demonstrated a statistically significant increased risk for hearing loss in preterm infants compared to term‐born infants. This suggests that prematurity might be an additive risk for hearing loss in infants undergoing complex cardiac surgery. However, the presence of multiple comorbidities and the smaller sample size of preterm infants in these studies may contribute to a misinterpretation of the risk of hearing loss. Survival bias among preterm infants requiring cardiac surgery with cardiopulmonary bypass might be a further confounding factor. The studies by Bork et al. [26] and Grasty et al. [27] emphasise the need for long‐term follow‐up after cardiac surgeries, including hearing assessments.

#### 4.2.7 In Utero Infections

Most of the included studies reported a decreased risk of sensorineural hearing loss in preterm infants with congenital CMV infection, although effect sizes varied. Due to low or moderate study quality, lack of clear definition of gestational age at infection, deficient hearing tests, insufficient follow‐up periods, and conflicting results, no definitive conclusions can be drawn. Congenital CMV infection is a known risk factor for sensorineural hearing loss [4, 39, 44], as viral replication can cause inflammation, cochlear damage, and spiral ganglion cell loss [45]. Therefore, continuous, developmentally appropriate follow‐up evaluations are required up to the age of 3 years and longer [46].

#### 4.2.8 Maternal Zika Virus Infection

The included study showed a decreased risk of hearing loss in preterm infants with Zika virus infection and related microcephaly [15]. Yet, no conclusions can be drawn due to low study quality, differences in the definition of Zika virus infection between the study and the JCIH, insufficient hearing tests and short follow‐up periods. In addition, conclusions about a causal relationship may be constrained by the small sample size and the associated low statistical power.

Khairy et al. [28] demonstrated that sepsis is a significant risk factor for hearing loss in preterm infants, which aligns with previous research [47, 48]. Methodological limitations, such as a small study sample size and an imprecise definition of hearing thresholds, restrict the generalisability of these findings.

In addition to the risk factors for hearing loss outlined by the JCIH, the following potential risk factors for hearing loss in preterm and term‐born infants were reported.

#### 4.2.9 Cerebral Palsy

Based on data from Gincota Bufteac et al. [22] a reduced risk of hearing loss was observed in preterm infants with cerebral palsy compared to term infants. Information about the aetiology and the severity or classification of cerebral palsy was lacking. The complexity of survival‐related biases and the lack of comparability between the characteristics of preterm and term infants made a direct comparison challenging. Cerebral palsy is associated with an increased risk of hearing loss, and early intervention is crucial [49].

#### 4.2.10 Mechanical Ventilation and Neonatal Respiratory Failure

The relationship between ventilation, respiratory failure and hearing loss is not yet fully understood. Further studies are required to understand its impact on conductive hearing loss and sensorineural hearing loss. Mechanical ventilation is associated with a higher likelihood of failing the hearing screening. In contrast, the use of continuous positive airway pressure devices was not associated with increased risks [50]. Khairy et al. [28] found that mechanical ventilation is a significant risk factor for hearing loss in preterm infants, which is consistent with previous studies [42, 47, 48]. Similarly, Robertson et al. [24] identified an increased risk of hearing loss in preterm infants with neonatal respiratory failure, but no additional risk from prematurity itself. While these studies highlight the association between ventilation, respiratory failure and hearing loss, they do not specifically address conductive hearing loss. Khairy et al. [28] focused on general hearing loss, while Robertson et al. [24] examined sensorineural hearing loss. Neither study addressed conductive hearing loss, which could be a consequence due to higher respiratory infection rates. Hypoxia, oxidative stress and barotrauma [48] can cause cochlear and neural damage [47] contributing to sensorineural hearing loss. Methodological limitations, such as small sample sizes and unclear hearing thresholds [28] limited the generalisability of these findings. To adequately compare preterm and term‐born infants, it is essential to account for confounders like NICU stay or drug exposure, as well as interventions like less invasive surfactant application or non‐invasive respiratory support.

The following risk factors, which were published by the JCIH, were not investigated in any of the included studies. Therefore, no conclusions can be drawn from the current evidence regarding the association of these risk factors with hearing loss in preterm and term‐born children, including craniofacial malformations, congenital syndromes, and caregiver concern. It is worth noting that caregiver concern may be more pronounced in parents of preterm infants.

#### 4.2.11 Socioeconomic Risk Factors

None of the included studies directly investigated the influence of socioeconomic risk factors on hearing loss. This may play a crucial role, particularly for preterm infants who require more intensive medical follow‐up. Socioeconomic factors, such as passive smoking [51], are particularly relevant in the context of conductive middle ear hearing loss. Limited access to healthcare and lower parental education levels are likely to influence the recognition of hearing loss. Parental awareness and knowledge play a crucial role in identifying potential hearing issues in children [52]. Given these challenges, socioeconomic disadvantage could be a potential barrier to the early detection and intervention of hearing loss.

#### 4.2.12 Conductive Hearing Loss

None of the studies that met the inclusion criteria addressed risk factors for conductive hearing loss in preterm compared to term infants. Further research is needed in this area.

### 4.3 Prematurity and Hearing Loss

Preterm infants exhibit a higher incidence of hearing loss [1] and a greater frequency and accumulation of risk factors [29]. One hypothesis suggests that an immature blood–brain barrier may contribute to this vulnerability [30]. However, the limited evidence identified in this systematic review does not confirm this hypothesis. Nearly all very preterm infants exhibit the risk factor of a NICU stay exceeding 5 days. This risk factor for hearing loss is well supported by evidence [37] and was confirmed in our review in both preterm and term infants. This is reflected in the recommendation to use automated auditory brainstem response testing for newborn hearing screening in both the German guidelines [53] and the JCIH [4]. Despite the limited evidence, the JCIH reached a consensus to recommend further follow‐up for infants with risk factors, at 9 months of age. This is recommended for those with a NICU stay exceeding 5 days to identify progressive or delayed‐onset sensorineural hearing loss or acquired conductive hearing loss [4]. Due to the accumulation of neonatal risk factors and higher rates of respiratory infections in very preterm infants, we advocate for adopting this international recommendation in Germany to improve the detection of progressive or delayed‐onset hearing loss. To improve diagnostic accuracy, it is essential to accompany automated auditory brainstem response with tympanometry (1000 Hz for small ear canals) [54] and otoscopy, where possible, to exclude middle ear effusion as a cause for false‐positive automated auditory brainstem response results. Conductive hearing loss is common in very preterm children, often resulting from recurrent middle ear infections, such as otitis media [53]. In addition, conductive hearing loss and early sensory deprivation in preterm infants have been linked to an increased risk of developing auditory processing disorders later in life [16]. If middle ear effusion persists beyond 3 months, despite conservative management by a paediatrician or general practitioner, referral to an Ear, Nose and Throat specialist or paediatric audiologist is warranted [54]. Newborn hearing screening with automated auditory brainstem response provides an established early detection strategy, and congenital CMV screening is increasingly being considered. However, no comparable strategies exist to systematically detect and adequately treat conductive or sensorineural hearing loss in other high‐risk paediatric populations such as preterm born children, children with intellectual disabilities, or those with neurodevelopmental disorders. This highlights an important research gap, which the German project HörGeist [55] aims to address. This project focuses on investigating and developing diagnostic and examination algorithms for hearing disorders in individuals with intellectual disabilities and offers guidelines for healthcare professionals. Until further evidence is provided, we suggest offering audiologic evaluation to any very preterm born child with either established risk factors, recurrent respiratory infections (exceeding five per season), any developmental delay, or parental concern about hearing [1, 56]. Adequate algorithms for the detection of hearing impairment in high‐risk and preterm children are presented in Figure S1.

### 4.4 Studies Published After the Search Date

A study by Hemmingsen et al. [2] examined risk factors for sensorineural hearing loss in preterm infants compared to term infants, based on a Norwegian national registry data. Sensorineural hearing loss was defined by using ICD‐10 codes without explicitly mentioning hearing loss characteristics and the used hearing assessments. Brainstem auditory evoked responses and pure‐tone audiometry were assumed as standard practice. The study analysed the different gestational age groups, extremely preterm, very preterm, and moderately preterm compared to term infants. Known risk factors or proxies for these, such as APGAR score, small for gestational age, caesarean section, antibiotic therapy, non‐invasive respiratory support, mechanical ventilation, intracranial haemorrhage, jaundice therapy, and parental consanguinity, were confirmed in very preterm, moderately preterm and term infants. However, for extremely preterm infants, only intracranial haemorrhage significantly increased the risk for sensorineural hearing loss, indicating that extremely preterm birth itself was the determining risk factor [2]. Due to its publication date after the review process had been completed, this study was not included in the present systematic review. While the study provided valuable information of improved quality, it does not alter our recommendations. We assume that preterm infants with the mentioned risk factors are likely to have a NICU stay exceeding 5 days and should therefore already be covered by the JCIH follow‐up guidelines [4]. In conclusion, this study represents an important step towards improving research on risk factors for hearing loss comparing preterm and term infants. However, as the authors stated, methodological limitations in the assessment of hearing, definition and details of risk factors, and identification of confounders highlight the need for further research to refine recommendations.

## 5 Strength and Limitations

The strength of our systematic review was the strict adherence to recommendations of the Cochrane methodological standards, ensuring a high‐quality literature search and synthesis. We conducted a comprehensive review of the literature using a broad search strategy. The inclusion criteria were predefined, and any deviations from the protocol, which was published prior to this review, were transparently documented. Two independent reviewers carried out the review process.

Several gaps in the existing research on hearing loss in preterm versus term infants limit our study. Methodological weaknesses, such as small cohort sizes, inconsistent study designs and unmeasured confounding variables, are prevalent. In addition, inconsistent definitions of hearing loss and risk factors, alongside survival bias, likely lead to underestimation of hearing loss risks in preterm infants. Substantial variation in the timing and methods of hearing assessments complicated comparisons and precluded meta‐analysis. Many studies used heterogeneous audiometric methods and lacked confirmatory diagnostics. Moreover, screening results identified as atypical or pathological did not necessarily indicate progressive hearing loss. In addition, middle ear issues were not investigated, and socioeconomic risk factors were not addressed in relation to conductive hearing loss in the population of preterm infants. Although the risk of hearing loss in preterm infants is well established, only two studies directly compared risk factors between preterm and term infants as a primary research question. To draw more reliable conclusions, future studies should standardise hearing assessments based on developmental age. They should clearly define the types of hearing loss, control for relevant risk factors and ensure comparability between preterm and term groups. These limitations highlight the need for further research to identify subgroups of preterm infants at higher risk for hearing loss and to improve screening and prevention strategies. Such efforts will be essential in designing targeted, evidence‐based interventions, optimising healthcare resource allocation, and improving long‐term outcomes for preterm infants. Detailed proposals for a suitable study design concerning conductive hearing loss are outlined in Appendix S3, offering practical guidance for addressing these research gaps.

## 6 Conclusion

The available evidence indicates but does not definitively establish whether prematurity itself is an independent risk factor for hearing loss. However, preterm infants have a higher prevalence of hearing loss and are exposed to an accumulation of risk factors. The available evidence supports the recommendation for newborn hearing screening using automated auditory brainstem response in preterm infants. Despite lacking scientific evidence, the consensus recommendation for an audiologic follow‐up in very preterm‐born children at an age of 9 months seems justified due to their obligatory NICU stay exceeding 5 days. Until further data on optimal audiologic follow‐up of preterm‐born infants is published, we suggest audiologic follow‐up during childhood for very preterm‐born children with either established risk factors, increased rates of respiratory infections, or any developmental delay. In addition, more advanced algorithms for detecting auditory neuropathy should be developed.

## Author Contributions

**Pauline Roehrs:** conceptualization, investigation, writing – original draft, methodology, validation, visualization, writing – review and editing, formal analysis, project administration, data curation, resources, supervision. **Anna‐Katharina Rohlfs:** writing – review and editing, conceptualization, supervision, validation. **Reinhard Vonthein:** conceptualization, software, formal analysis, supervision, writing – review and editing, data curation, validation. **Camilla Simon:** conceptualization, investigation, methodology, writing – review and editing, validation. **Juliane Spiegler:** supervision, resources, conceptualization, methodology, validation, writing – review and editing, project administration, investigation.

## Conflicts of Interest

The authors declare no conflicts of interest.

## Supporting information


Appendix S1.
