# Supplementary material for: Risk Factors for Hearing Loss Are Comparable in Preterm Versus Term Children: A Systematic Review
Source: Acta Paediatr. 2025 Jul 15;114(11):2763–82. doi: 10.1111/apa.70222 (PMC12520271; doi:10.1111/apa.70222)
Supplement: Supplementary file 1 — Appendix S1. [file APA-114-2763-s001.pdf]

Table of Contents

Appendix S1. Search strategy 2

Appendix S2. Study description and quality assessment of included studies (ordered by name of author) 3

Appendix S3. Summary of a Prospective study on CHL, APD and Language Development in Preterm Children 35

Table S1. Binomial logistic regression analysis of predictors for hearing impairment in subgroups of infants with different risk factors (Data of Khairy et al., 2018) 36

Table S2. Characteristics of excluded studies (ordered by name of author) 37

Figure S1. Diagnostic Algorithm for Early Detection and Monitoring of Hearing Impairment in Preterm and High-Risk Infants 138

Appendix S1. Search strategy

PEO schema

- *Patient/Population:* preterm infants (gestational age, <37 weeks) and full-term infants (gestational age ≥37 weeks);
- *Exposition*: present risk factor;
- *Outcome*: hearing impairment/hearing loss (objective or subjective hearing tests or examination of medical records).

General search strategy

(children OR infant OR “child”[MESH] OR “infant”[MESH] OR preterm OR premature OR low birth weight)

AND

(risk factors OR “Risk Factors”[MESH] OR infection OR drug exposure)

AND

(hearing loss OR hearing impairment OR “Hearing loss”[MESH])

Search strategy for Cochrane Library

#1	children OR infant OR preterm OR premature OR low birth weight
#2	MeSH descriptor: [Child] explode all trees
#3	MeSH descriptor: [Infant] explode all trees
#4	MeSH descriptor: [Infant, Low Birth Weight] explode all trees
#5	MeSH descriptor: [Infant, Premature] explode all trees
#6	risk factors OR infection OR drug exposure
#7	MeSH descriptor: [Risk Factors] explode all trees
#8	hearing loss OR hearing impairment
#9	MeSH descriptor: [Hearing Loss] explode all trees
#10	#1 or #2 or #3 or #4 or #5
#11	#6 or #7
#12	#8 or #9
#13	#10 and #11 and #12

Search strategy for Pubmed

(((children OR infant OR "child"[MESH] OR "infant"[MESH] OR preterm OR premature OR low birth weight OR “Infant, Low Birth Weight”[MESH] OR “Infant, Premature”[MESH])) AND ((risk factors or "Risk Factors"[MESH] OR infection OR drug exposure))) AND ((hearing loss OR hearing impairment OR "Hearing loss"[MESH]))

Search strategy for Web of Science

((ALL=(child* OR infant* OR preterm OR premature OR low birth weight)) AND ALL=(risk factor* OR infection* OR drug exposure)) AND ALL=(hearing loss OR hearing impairment)

Appendix S2. Study description and quality assessment of included studies (ordered by name of author)

Bork, 2017

**Methods**	Prospective observational study (1996-2015) to report audiology follow-up in a cohort by registered pediatric-experienced audiologist (at 6-8 months post surgery and age 2 years) after complex cardiac surgery with cardiopulmonary bypass at ≤6 weeks of life.
**Participants**	**N = 691 children who underwent complex cardiac surgery**<br>*Initially 841 infants ≤ 6 weeks with complex cardiac surgery, death before age 2 years was 135 infants, incomplete follow-up for 15 infants.*

Group I:	**41 infants with permanent hearing loss**<br>N (preterm infants) = 8 infants<br>N (full-term born infants) = 33 infants
Group II:	**650 infants without permanent hearing loss**<br>N (preterm infants) = 68 infants<br>N (full-term born infants) = 582 infants
Inclusion criteria:	- survivors of complex cardiac surgery at ≤42 days of life at Stollery Children's Hospital, Edmonton, Alberta from September 1996 until May 2015<br>- survival to age of 2 years<br>- audiology follow-up
**Exclusion criteria**:	Not stated but there was no exclusion.

Characteristics:	***Group I:***	***Group II:***
- Sex (m/f):	419/231 (64.5%/35.5%)	26/15 (63.4%/26.6%)
- Birthweight (kg):	3.3 (±0.6)	3.2 (±0.7)
- Prematurity (<37 weeks):	68 (10.5%)	8 (19.5%)
- Syndrome or genetic abnormalities:	70 (10.7%)	17 (41.5%)
- SV defect	173 (26.9%)	21 (51.2%)
- Family socioeconomic index:	42.9 (±14.4)	40.1 (±11.1)
- Weight at surgery (kg):	3.4 (±0.6)	3.3 (±0.7)
- Year of surgery:	2006.9 (±5.1)	2006.2 (±4.1)
- Cardiopulmonary bypass time (min):	113.2 (±47)	116.2 (±50.7)
- Deep hypothermic circulatory arrest:	454 (69.8%)	29 (70.7%)
- Dialysis:	84 (13.1%)	9 (22.0%)
- Cardiopulmonary resuscitation:	30 (4.6%)	6 (14.6%)
- Convulsion:	46 (7.1%)	5 (12.2%)
- ECMO:	33 (5.1%)	4 (9.8%)
- Total ventilation (d):	14.2 (±13.4)	28.7 (±25.5)
- Total hospitalization (d):	29.2 (±26.0)	56 (±53.2)

**Outcomes**	***Ascertainment of hearing impairment:*** Audiological examinations in sound booths were administered by registered pediatric-experienced audiologists at a minimum of 6-8 months post surgery, at age 2 years, and as required throughout and thereafter to complete diagnoses and monitor thresholds. Developmentally appropriate and clinically standardized hearing testing procedures were used.
**Notes**	Study was conducted from 1996-2015 at Stollery Children's Hospital in Edmonton, Alberta, a referral hospital in western Canada for neonatal and pediatric complex cardiac surgery.<br><br>Ethics board approvals were obtained at all sites (institutional review board approval: Health Research Ethics Board, University of Alberta, Edmonton, Alberta, Canada, ID# Pro00001030; last renewal date, November 17, 2017).<br>All parents or guardians signed consent.<br><br>***Funding Source:*** Multidisciplinary follow-up after Early Cardiac Surgery in Alberta has been supported by contributions from Alberta Health, Glenrose Rehabilitation Hospital, Edmonton, AB, Stollery Children's Hospital, Edmonton, AB, and referral- site follow-up clinics (Saskatoon and Regina SK, Winnipeg MB, Calgary AB, Vancouver BC). These funding agencies had no role in the study design and conduct of the study; analysis or interpretation of data; preparation, review, or approval of the manuscript; or decision to submit the manuscript for publication.<br><br>***Declaration of interest:*** The authors declare no conflicts of interest.

Risk of bias (Newcastle-Ottawa Quality Assessment Scale)

Bias	***Authors' judgement***	***Support for judgement***
Selection	1/4*	
- Representativeness of exposed cohort	0/1*	Selected group, only preterm infants who underwent complex cardiac surgery at less than 6 weeks of age and survived to age of 2 years.
- Selection of the non-exposed cohort	1/1*	Drawn from the same community as the exposed cohort.
- Ascertainment of exposure	0/1*	Ascertainment of gestational age not clearly stated, neither in this paper nor in therefore referred sources.
- Demonstration that outcome of interest	0/1*	Not demonstrated.<br>was not present at start of study

Comparability	0/2*	
- Comparability of cohorts on the basis of the design or analysis	0/2*	Study controls only for performed complex cardiac surgery with cardiopulmonary bypass in preterm and full-term born infants. No comparability because there are no information about e.g. grade of prematurity, mediaction and complications. Pre- and full-term infants cannot be adequately compared based on available information.

Outcome	3/3*	
- Assessment of outcome	1/1*	Audiological examinations were administered by registered pediatric-experienced audiologists. Developmentally appropriate and clinically standardized hearing testing procedures were used.
- Was follow-up long enough for outcomes to occur	1/1*	7-9 months and 2 years of age sufficient for detection of hearing loss resulting from CCS.
- Adequacy of follow up of cohorts	1/1*	97.9% of 2-year-old survivors completed audiological follow-up.
Overall result:	4/9*	**Low quality.**

Corujo-Santana, 2014

Methods

Retrospective cohort study of children born in the Complejo Hospitalario Universitario Insular Materno-Infantil of Gran Canaria during the period of 2007-2011to examine hyperbilirubinemia at birth as a risk factor for sensorineural hearing loss in children.
Study as part of the Program for the Early Detection of Childhood Hearing Loss.

Participants

N = 92 infants with hyperbilirubinemia

Initially 796 infants were examined, but to answer the question about differences in hearing impairment in preterm and full-term born infants only the subgroup of 92 infants with hyperbilirubinemia was analyzed.

Group I: 62 preterm infants with hyperbilirubinemia
N (Hearing impairment)=24 infants
N (No hearing impairment) = 38 infants

Initially 427 preterm infants, for relevant analysis only subset of 62 infants used.

Group II: 30 full-term infants with hyperbilirubinemia
N (Hearing impairment)=11 infants

N (No hearing impairment) = 19 infants

Initially 427 fullterm infants, for relevant analysis only subset of 62 infants used.

Inclusion criteria: - diagnosis of perinatal hyperbilirubinemia
- included in the Program for the Early Detection of Childhood Hearing Loss of the Complejo Hospitalario Universitario Insular Materno-Infantil de Gran Canaria.

Characteristics:	***Preterm group***	***Full-term group***
- Gender (m/f):	258/169 (60.4%/39.6%)	217/152 (58.8%/41.2%)
- Total Blood Bilirubin		
o 5-13.99 mg/dl:	207 (48.5%)	74 (20.1%)
o 14-19.99 mg/dl:	188 (44.0%)	210 (56.9%)
o ≥ 20 mg/dl:	32 (7.5%)	85 (23.0%)
- Hearing loss:	24/62 (38.7%)	11/30 (36.7%)

Outcomes

Ascertainment of hearing loss:
- first examination: otoacustic emission (OAEs) during first 48h of life
- diagnosis and follow-up using evoked auditory potentials in the brain stem (EAPBS)

Notes

Study conducted at Complejo Hospitalario Universitario Insular Materno-Infantil de Gran Canaria from 2007-2011.
Study was approved by the Clinical Trials Committee of the Complejo Hospitalario Universitario Insular Materno- Infantil, Gran Canaria.

Funding Source: Not stated.

Declaration of interest: Authors declare to have no conflict of interest.

Risk of bias (Newcastle-Ottawa Quality Assessment Scale)

Bias	***Authors' judgement***	***Support for judgement***
Selection	1/4*	
- Representativeness of exposed cohort	0/1*	Selected group of preterm infants with hyperbilirubinemia.
- Selection of the non-exposed cohort	1/1*	Full-term born infants drawn from the same community as the exposed cohort.
- Ascertainment of exposure	0/1*	No description.
- Demonstration that outcome of interest was not present at start of study	0/1*	Not stated.
Comparability	1/2*	
- Comparability of cohorts on the basis of the design or analysis	1/2*	Study controls for sex and bilirubinlevel of preterm and full-term born

		infants, but not for different levels of bilirubin in preterm groups. No information about e.g. grade of prematurity, mediaction and complications given. Pre- and full-term infants can only be compared very limited based on available information.
Outcome	0/3*	
- Assessment of outcome	0/1*	OAEs and EAPBS.
- Was follow-up long enough for outcomes to occur	0/1*	No, first 48 hours of life not long enough for detection of hearing impairment caused by hyperbilirubinemia.
- Adequacy of follow up of cohorts	0/1*	Follow up rate <50% (92/185 infants).
Overall result:	2/9*	**Low quality.**

Gincota Bufteac, 2018

**Methods**	Retrospective cohort study to estimate the prevalence of cerebral palsy and to describe the severity of associated problems (e.g. severe hearing impairment) in Moldova.	
**Participants**	**N = 185 children with cerebral palsy**<br>*Initially 207 infants, 22 infants were lost to follow-up/had incomplete data.*	
	Group I:	10 infants with hearing loss<br>*N(preterm) = 9 infants*<br>*N (full-term) = 1 infant*
	Group II:	175 infants without hearing loss<br>*N (preterm) = 51 infants*<br>*N (full-term) = 124 infants*
	Inclusion criteria:	- children with CP treated at the National Hospital Institute of Mother and Child, in Chisinau, Moldova<br>- born between January 1st 2009 and December 31st 2010
	Exclusion criteria:	- acquired CP lesions after the neonatal period

Characteristics:

- Gestational age:
  - <28 w: 10 infants (5%)
  - 28-31 w: 12 infants (7%)
  - 32-36 w: 30 infants (16%)
  - ≥37 w: 133 infants (72%)
- Birthweight:
  - <1000g: 6 infants (3%)
  - 1000-1499g: 11 infants (6%)

- o 1500-2499g: 38 infants (21%)
  - o ≥2500g: 130 infants
- Cerebral palsy:
  - o Unilateral CP: 37 infants
  - o Bilateral CP: 113 infants
  - o Dyskinetic CP: 22 infants
  - o Ataxic CP: 9 infants
  - o Not classified CP: 4 infants

**Outcomes**	***Ascertainment of hearing function***:<br>- Medical records.
**Notes**	The study was approved by the National Committee for Ethical Expertise in Clinical Trials (Nr. 266) and by the Centre of Early Intervention 'Voinicel' Ethical Committee (nr. 01/17).<br><br>***Funding Source*****:** This study was supported by a student grant from the Ministry of Foreign Affairs of Norway and AHEAD-Moldova, Norway, within the PhD program at Oslo Metropolitan University, Faculty of Health Sciences, Oslo, Norway.<br><br>***Declaration of interest:*** The authors declare that they have no competing interests.

Risk of bias (Newcastle-Ottawa Quality Assessment Scale)

Bias	***Authors' judgement***	***Support for judgement***
Selection	2/4*	
- Representativeness of exposed cohort	0/1*	Selected group of preterm infants with CP in Moldova.
- Selection of the non-exposed cohort	1/1*	Drawn from the same community as the exposed cohort.
- Ascertainment of exposure	1/1*	Medical records.
- Demonstration that outcome of interest was not present at start of study	0/1*	No.
Comparability	1/2*	
- Comparability of cohorts on the basis of the design or analysis	0/2*	Study only controls for presence of cerebral palsy in preterm and full-term born infants. No comparability because there are no information about e.g. grade of cerebral palsy, grade of prematurity, mediaction and complications of prematurity are available. Pre- and full-term infants cannot be adequately compared based on available information.
Outcome	1/3*	

- Assessment of outcome	0/1*	Hearing function was assessed by medical records.
- Was follow-up long enough for outcomes to occur	0/1*	Only one assessment at age 7-8 years, not sufficient to diagnose permanent hearing loss.
- Adequacy of follow up of cohorts	1/1*	Follow-up rate 89.4%, audiology results for 185/207 infants available.
Overall result:	4/9*	**Low quality.**

Grasty, 2018

**Methods**	Prospective observational cohort study to investigate the prevalence of hearing loss after cardiac surgery in infancy, patient and operative factors associated with hearing loss and the relationship of hearing loss to neurodevelopmental outcomes.
**Participants**	**N = 345 infants with with cardiac surgery in infancy**<br>*Initially 550 infants eligible, 64 infants died, 105 were lost to follow-up, no hearing test was administered in 33 infants and gestational age was not available for 3 infants.*

Group I:	74 infants with hearing loss<br>*N (preterm) = 18 infants*<br>*N (full-term) = 56 infants*
Group II:	271 infants without hearing loss<br>*N (preterm) = 29 infants*<br>*N (full-term) = 242 infants*
Inclusion criteria:	- ≤6 months of age<br>- undergoing surgical treatment of CHD with cardiopulmonary bypass, with or without deep hypothermic circulatory arrest (DHCA).<br>- completed standard audiologic evaluation as part of a comprehensive neurodevelopmental evaluation at 4 years of age (2003-2008).
Exclusion criteria:	- multiple congenital anomalies<br>- recognizable genetic or phenotypic syndrome other than chromosome 22q11 microdeletion syndrome<br>- language other than English spoken at home

Characteristics:	***Group I:***	***Group II:***
- Gestational age		
- <37 weeks	18/47 (38.3%)	29/47 (61.7%)
- ≥37 weeks	56 (18.8%)	242 (81.2%)
- Birthweight (kg):	3.1 (± 0.7)	3.2 (± 0.6)
- Sex (f/m):	32 (42.7%)/43(57.3%)	118(43.2%)/155(56.8%)
- Age at first	35.8 (± 48.5)	44.9 (± 55.5)

surgery (d):

**Outcomes**	***Ascertainment of hearing loss:***<br>- Audiologic evaluations were conducted using standard pediatric assessment methods based on developmental ability
**Notes**	Study was conducted at Children's Hospital of Philadelphia and approved by the institutional review board.<br>Informed consent was obtained from parents or guardians.<br><br>***Funding Source:*** Supported by the Fannie E. Rippel Foundation, an American Heart Association National Grant-in-Aid (9950480N), the National Institutes of Health (HL071834), the National Institute of Neurological Disease and Stroke (1R01NS-072338 [to D.J.L.]) 1R01NS060653 (to D.J.L.), and the June and Steve Wolfson Family Foundation, Philadelphia, Pennsylvania (to D.J.L.).<br><br>***Declaration of interest:*** The other authors declare no conflicts of interest.

Risk of bias (Newcastle-Ottawa Quality Assessment Scale)

Bias	***Authors' judgement***	***Support for judgement***
Selection	2/4*	
- Representativeness of exposed cohort	0/1*	Selected group of users, only preterm infants with congenital heart disease who underwent cardiac surgery in infancy.
- Selection of the non-exposed cohort	1/1*	Drawn from the same community as the exposed cohort.
- Ascertainment of exposure	1/1*	Review of medical records.
- Demonstration that outcome of interest was not present at start of study	0/1*	No.
Comparability	0/2*	
- Comparability of cohorts on the basis of the design or analysis	0/2*	Study only controls for presence of congenital heart disease and cardiac surgery in infancy in preterm and full-term born infants.<br>No comparability because there are no information about e.g. grade of prematurity, mediaction and complications of prematurity are available. Pre- and full-term infants cannot be adequately compared based on available information.
Outcome	1/3*	

- Assessment of outcome	0/1*	Measurement of pure tone air conduction and pure tone bone conduction.
- Was follow-up long enough for outcomes to occur	1/1*	Yes, after 4 years.
- Adequacy of follow up of cohorts	0/1*	Follow-up rate <80%, 550 infants enrolled, 486 infants eligible, 381 infants with completed followed up, 345 with complete data on hearing test and gestational age.<br>345/486 (=71.0%) < 80%.
Overall result:	**3/9***	**Low quality.**

Khairy, 2017

Methods Cohort study to identify the most significant risk factors for hearing impairment in high-risk neonates hospitalized at Neonatal Intensive Care Unit (NICU) and to assess the sensitivity of hearing screening tests.

Participants **N = 260 NICU-newborns**

Initially 320 infants, 70 lost to follow-up.

Group I: **150 preterm born infants**
N (with hearing impairment) = 48 infants
N (without hearing impairment) = 102 infants

Group II: **110 full-term born infants**
N (with hearing impairment) = 30 infants
N (without hearing impairment) = 80 infants

Inclusion criteria: Newborns discharged from the NICU after a stay of more than 48 hours and one or more of the following postnatal risk factors: treatment with ototoxic drugs (aminoglycosides, vancomycin, loop diuretics), indirect hyperbilirubinemia with necessitating exchange transfusion, culture proven sepsis, perinatal asphyxia, mechanical ventilation more than 5 days. Willingness of family to participate in study.

Exclusion criteria: Cases with multiple congenital anomalies, suspected perinatal infection (toxoplasma, rubella, herpes simplex, syphilis) or family history of hereditary hearing loss.

Characteristics:	***Preterm group***	***Term group***
- Mean gestational age:	32.7w (26-36w)	38.5w (38-40w)
- Birth weight:	1750g (930-3230g)	3100g (2000-4000g)
- Sex (m/f):	72/78<br>(52%/48%)	43/67<br>(60.9%/39.1%)
- NICU-stay:	17d (2-60d)	9.5d (4-45d)

	- Perinatal asphyxia:	31/150 (20.7%)	17/110 (15.5%)
	- Mechanical ventilation > 5d:	30/150 (20.0%)	17 (15.5%)
	- Sepsis*:	46/150 (30.7%)	17(15.5%)
	- Hyperbilirubinemia:	32 (21.3%)	21 (19.1%)
	- Use of Aminoglykosides*:	92 (61.3%)	40 (36.3%)
	- Use of Vancomycin:	21 (14.0%)	10 (9.1%)
	- Combined use of Aminoglykosides and Vancomycin*:	17 (11.3%)	2 (1.8%)
	- Use of loop diuretics:	7 (4.7%)	11 (10.0%)
	* Difference between groups was statistically significant.		
**Outcomes**	***Ascertainment of hearing impairment:***<br>- TEOAE and AABR at the Audiology Department of Cairo University.<br>- Clinical ear, nose and throat examination to rule out any evidence of infection or debris in the ear by tympanometry.		
**Notes**	Study conducted at Children Hospital Cairo University, Pediatrics, New Children Hospital, (Abu El Rish), Cairo University Hospitals, Ali Basha Ebrahim, Cairo, 11562 Egypt.<br>***Funding Source:*** Not stated.<br>***Declaration of interest:*** No conflicts of interest reported.		

Risk of bias (Newcastle-Ottawa Quality Assessment Scale)

Bias	***Authors' judgement***	***Support for judgement***
Selection	3/4*	
- Representativeness of exposed cohort	1/1*	Somewhat representative of the average preterm newborn with NICU stay in the community.
- Selection of the non-exposed cohort	1/1*	Drawn from the same community as the exposed cohort.
- Ascertainment of exposure	1/1*	Patients/medical records.
- Demonstration that outcome of interest was not present at start of study	0/1*	Not stated.
Comparability	2/2*	
- Comparability of cohorts on the basis of the design or analysis	2/2*	Comparability of preterm and full-term born infants given for, e.g. NICU stay, perinatal asphyxia, mechanical ventilation >5d.
Outcome	0/3*	

- Assessment of outcome	0/1*	Hearing outcome measured by AABR or TOAEs without tympanometrie.
- Was follow-up long enough for outcomes to occur	0/1*	No, directly after discharge from NICU following a stay of at least 48 hours. Not long enough to occur.
- Adequacy of follow up of cohorts	0/1*	260/330 (78.79%) patients, follow-up rate <80%.
Overall result:	**5/9***	**Moderate quality.**

Kim, 2018

Methods

Cohort study to investigate the epidemiology of cCMV infection-related SNHL and to demonstrate its clinical manifestations, specifically detailed audiological features in the Korean pediatric population.

Participants

N = 27 infants with congenital CMV infection

Initially 38 infants with cCMV infection examined, medical records only available for 11 infants.

Group I: 8 infants with SNHL
N(preterm) = 2 infants
N (full-term) = 6 infants

Group II: 19 infants without SNHL
N(preterm) = 16 infants
N (full-term) = 3 infants

Inclusion criteria: - Diagnosis of cCMV through urine PCR within the first two weeks of life. Identification through clinically driven diagnosis, routine newborn CMV screening according to NICU protocol.

Characteristics:

- Sex (m/f): 15 infants (55.6%)/12 infants (44.4%)
- Prematurity: 18/27 infants (66.7%)
- GG <1500g (VLBW): 15/27 infants (55.56%)
- NICU-stay > 5d: 22/27 infants (81.5%)

Outcomes

Ascertainment of hearing impairment:

- Review of medical records/of audiologic assessments (including ABR, AABR, PTA, OAE, DPOAE and TEOAE) for a loss of more than 25 dB.

Notes

This study was approved by the institutional review boards of Seoul National University Hospital (SNUH, no. J-1704-109-847) and Seoul National University Bundang Hospital (SNUBH, no. B-1612/376-106).

Funding Source: The authors declare that there are no conflicts of interest regarding the publication of this paper.

Declaration of interest: This work was supported by the Korean Health Technology R&D project, Ministry for Health, Welfare, Republic of Korea (no. HI12C0014), Basic Science Research Program through the National Research Foundation of Korea (NRF) funded by the Ministry of Education and the Brain Research Program through the National Research Foundation of Korea (NRF) funded by the Ministry of Science, ICT & Future Planning.

Risk of bias (Newcastle-Ottawa Quality Assessment Scale)

Bias	*Authors' judgement*	*Support for judgement*
Selection	2/4*	
- Representativeness of exposed cohort	0/1*	Selected group of users, only preterm infants with congenital CMV infection.
- Selection of the non-exposed cohort	1/1*	Drawn from the same community as the exposed cohort.
- Ascertainment of exposure	1/1*	Medical records.
- Demonstration that outcome of interest was not present at start of study	0/1*	Not stated.
Comparability	0/2*	
- Comparability of cohorts on the basis of the design or analysis	0/2*	Study controls for presence of cCMV infection in preterm and full-term born infants in analyzed subset of infants. No comparability because there are no information about e.g. grade of prematurity, mediaction and complications of prematurity are available. Pre- and full-term infants cannot be adequately compared based on available information.
Outcome	1/3*	
- Assessment of outcome	0/1*	Medical records.
- Was follow-up long enough for outcomes to occur	0/1*	One assessment at mean time to follow-up 39.8 months (1-145 months).
- Adequacy of follow up of cohorts	1/1*	Follow-up rate > 80%, 24/27 infants (88.89%).
Overall result:	**3/9***	**Low quality.**

Lanzieri, 2017

**Methods**	Longitudinal cohort study to assess risk factors for sensorineural hearing loss in infants with confirmed symptomatic cCMV disease. The study included children born during 1983–2005 who were enrolled in the Congenital CMV Longitudinal Study as case-patients with confirmed symptomatic congenital CMV disease, defined as newborns with CMV infection detected by culture of urine samples collected within 3 weeks of life who presented at least one defined CMV-related sign at birth.

Participants

N = 70 infants with confirmed symptomatic cCMV disease

Initially 76 infants with confirmed symptomatic congenital CMV disease and audiologic assessment, 6 infants were excluded, only 70 infants were included into the analysis of prematurity as risk factors for hearing loss.

Group I: **56 infants with sensorineural hearing loss**
Distribution of gestational age not specified.

Group II: **20 infants without sensorineural hearing loss**
Distribution of gestational age not specified.

Inclusion criteria: - infants born during 1983–2005 who were enrolled in the Congenital CMV Longitudinal Study as case-patients with confirmed symptomatic congenital CMV disease

Exclusion criteria: - infants who were small for gestational age (SGA) or had congenital SNHL in the absence of at least one of the defined signs of having symptomatic congenital CMV disease

Characteristics:
All participants with audiologic evaluations:
- Median age at first evaluation: 18 days (4 days – 8 years)

Group with sensorineural hearing loss:
- Onset of hearing loss
- Congenital/early-onset SNHL: 56 (79%)
- Delayed onset SNHL: 12 (21%)
- Laterality of sensorineural hearing loss:
- Bilateral SNHL: 47 (84%)
- Unilateral SNHL: 9 (16%)
- Severity of sensorineural hearing loss:
    - 25 dB in isolated frequency: 1 (2%)
    - Slight (16–25 dB): 5 (11%)
    - Mild (26–40 dB): 2 (4%)
    - Moderate (41–55 dB): 4 (9%)
    - Moderately severe (56–70 dB): 1 (2%)
    - Severe (71–90 dB): 7 (15%)
    - Profound (490 dB): 27 (57%)

Outcomes

Ascertainment of hearing impairment:
Hearing evaluations included click and tone-burst auditory brainstem response, behavioral audiometry from 0.25 to 8 kHz and tympanometry.

**Notes**	Study was conducted from 1983-2005 using case-patients enrolled in the Congenital CMV Longitudinal Study. 73 participants were referrals from several hospitals and 4 were identified by routine newborn CMV screening at Women's Hospital of Texas (Houston, Texas, USA).<br>All Parents of infants enrolled in Congenital CMV Longitudinal Study provided consent.<br>The institutional review board for Human Subject Research for Baylor College of Medicine and Affiliated Hospitals approved the study protocol.<br>***Funding Source:*** Not stated.<br>***Declaration of interest:*** Dr Demmler-Harrison's institution received funding from Merck Sharpe & Dohme Corporation since July 2016 to assist with salary support for further analysis on long- term outcomes of congenital CMV infection not included in this report. The remaining authors declare no conflict of interest.

Risk of bias (Newcastle-Ottawa Quality Assessment Scale)

Bias	***Authors' judgement***	***Support for judgement***
Selection	2/4*	
- Representativeness of exposed cohort	0/1*	Selected group, only preterm born infants with symptomatic congenital CMV disease detected at birth.
- Selection of the non-exposed cohort	1/1*	Drawn from the same community as the exposed cohort.
- Ascertainment of exposure	0/1*	Ascertainment of gestational age was not described.
- Demonstration that outcome of interest was not present at start of study	1/1*	Exclusion of infants with at least one of the defined signs of having cCMV disease, e.g. SNHL.
Comparability	0/2*	
- Comparability of cohorts on the basis of the design or analysis	0/2*	Study controls for presence of congenital CMV disease at birth in preterm and full-term born infants.<br>No comparability because there are no information about e.g. grade of prematurity, mediaction and complications of prematurity are available. Pre- and full-term infants cannot be adequately compared based on available information.
Outcome	3/3*	
- Assessment of outcome	1/1*	Hearing evaluations included click and tone-burst auditory

		brainstem response, behavioral audiometry from 0.25 to 8 kHz and tympanometry.
- Was follow-up long enough for outcomes to occur	1/1*	Yes, case patients were followed with neurodevelopmental, hearing and ophthalmologic evaluations during infancy, preschool, elementary, middle and high school years.
- Adequacy of follow up of cohorts	1/1*	Audiologic assessment was performed in all 76 case-patients.
Overall result:	5/9*	**Moderate quality.**

Leal, 2016

Methods Cohort study to investigate hearing loss in infants with microcephaly and evidence of congenital zika virus infection, aged 0-10 months, referred to Hospital Agamenon Magalháes in Pernambuco, Brazil.

Participants **N = 64 infants with microcephaly and congenital ZIKA virus infection**

Initially 150 infants, information about gestational age was not available for 6 infants, 80 were excluded for reasons.

Group I: 5 infants with SNHL
N(preterm infants) = 0 infants
N (full-term infants) = 5 infants

Group II: 59 infants without SNHL
N (preterm infants) = 8 infants
N (full-term infants) = 51 infants

Inclusion criteria:
- Microcephaly (defined as head circumference ≤ 32 cm for term born infants; or at least two SD below the mean for gestational age and sex for preterm infants (Fenton curve))
- and laboratory evidence of Zika virus infection (by positive virurs specific IgM in ELISA of cerebrospinal fluid)
- and evaluated during 11/2015 – 05/2016.

Exclusion criteria:
- Other infectious causes of congenital sensorineural hearing loss (CMV, toxoplasmosis, herpes simplex and syphilis), detected by serologic testing of infants and their mothers.

Characteristics:	***Group I:***	***Group II:***
- Gestational age:		
- Preterm:	0	8 (14%)
- Term/Postterm:	5 (100%)	51 (86%)
- Sex (male/female):	3 (60%)/2(40%)	36 (55%)/29 (45%)
- Degree of microcephaly		

- Severe:	4 (100%)	39 (65%)
- Other:	0	21 (35%)

Outcomes *__Ascertainment of hearing impairment:__*

- Auditory brainstem response (ABR) as screening and diagnostic test.

Notes Although all investigations were carried out as part of routine clinical care, and human subjects review was not required, the protocol was submitted for ethical review and approved by Hospital Agamenon Magalhães.

Funding Source: Not stated.

Declaration of interest: Not stated.

Risk of bias (Newcastle-Ottawa Quality Assessment Scale)

Bias	*Authors' judgement*	*Support for judgement*
Selection	1/4*	
- Representativeness of exposed cohort	0/1*	Selected group of users, only preterm infants with microcephalie and ZIKA virus infection.
- Selection of the non-exposed cohort	1/1*	Drawn from the same community as the exposed cohort.
- Ascertainment of exposure	0/1*	No description.
- Demonstration that outcome of interest was not present at start of study	0/1*	Not stated.
Comparability	0/2*	
- Comparability of cohorts on the basis of the design or analysis	0/2*	Study controls for presence of congenital ZIKA virus infection in preterm and full-term born infants. No comparability because there are no information about e.g. grade of microcephalie, grade of prematurity, medication and complications of prematurity are available. Pre- and full-term infants cannot be adequately compared based on available information.
Outcome	1/3*	
- Assessment of outcome	0/1*	Auditory brainstem response (ABR).
- Was follow-up long enough for outcomes to occur	0/1*	Yes, 0-10 months, mean time to follow-up is 97 days.
- Adequacy of follow up of cohorts	1/1*	Follow-up rate > 80%, 64/70 infants (91.4%) with completed

follow-up.

Overall result: **2/9*** **Low quality.**

Omar, 2022

**Methods**	Prospective cross-sectional study that was conducted on 200 neonates who were admitted to NICU at Assiut University Hospital.<br>The study aimed to assess the prevalence of hearing loss and the percentage of different risk factors among the high-risk register neonates admitted to NICU at Assiut University Hospital in the period between March 2020 and January 2021.
**Participants**	**N = 200 neonates admitted to NICU**<br><br>***Group I:*** **191 infants without hearing loss**<br>*Distribution of gestational age not specified.*<br><br>***Group II:*** **9 infants with hearing loss**<br>*Distribution of gestational age not specified.*<br><br>***Inclusion criteria***: - neonates of both sexes who were admitted to NICU at Assiut University Hospital<br>- between March 2020 and January 2021<br><br>***Exclusion criteria:*** Not stated.<br><br>***Characteristics***:<br>*All participants with audiologic evaluations:*<br>- Gender (m/f): 118(59%)/82(41%)<br>- Gestational age (weeks): 34.8±3.5 (26-40)<br>- Preterm: 123 (61.5%)<br>- Full-term: 77 (38.5%)<br>- Bodyweight:<br>- 500g – 1.7 kg: 124 (62%)<br>- > 1.7 kg: 76 (38%)<br>- Delivery:<br>- Caesarean: 120 (60%)<br>- Vaginal delivery: 80 (40%)<br>- Low birthweight: 21 (10.5%)<br>- Hyperbilirubiemia with blood transfusion: 5 (2.5%)<br>- Craniofacial anomalies: 1 (0.5%)<br>- Brain anoxia: 1 (0.5%)<br>- ≥1 of risk factors: 122 (61%)
**Outcomes**	***Ascertainment of hearing impairment:***<br>- *Hearing screening:* all neonates were screened by TEOAE<br>- *Diagnostic ABR* was performed for the neonates who failed the second stage screening, and for the neonates with hyperbilirubinemia.

Notes The research was carried out between March 2020 and January 2021 at Faculty of Medicine, Assiut University, Egypt.
Informed written consent was obtained from all parents/ guardians of all participants under 16 years old in the study.
Ethical approval was obtained by the ethical committee, Faculty of Medicine, Assiut University, Egypt. Approval number (17100662).

Funding Source: Not applicable.

Declaration of interest: The authors declare that they have no competing interests.

Quality assessment *(JBI Critical appraisal checklist for analytical cross sectional studies)*

1. **Were the criteria for inclusion in the sample size clearly defined?**
   a. **Authors judgement:** Yes.
   b. **Support for judgement:** The study included 200 neonates admitted to the NICU at Assiut University Hospital with risk factors for hearing impairment (defined by the Joint Committee on Infant Hearing (JCIH) 2007 guidelines). Exclusion criteria were not separately stated.
2. **Were the study subjects and the setting described in detail?**
   a. Authors judgement: Yes.
   b. Support for judgement: The population is clearly described as neonates admitted to the NICU with multiple high-risk factors for hearing loss. Demographic factors and risk factor (e.g., prematurity, low birth weight, hyperbilirubinemia) are presented in detail.
3. **Was the exposure measured in a valid and reliable way?**
   a. Authors‘ judgement: No.
   b. Support for judgement: The measurement/assessment of risk factors/exposition was not described in detail.
4. **Were objective, standard criteria used for measurement of the condition?**
   a. Authors‘ judgement: Yes.
   b. Support for judgement: Hearing impairment was measured using standard diagnostic procedures (TEOAE and ABR) and interpreted according to Rhode Island Criteria.
5. **Were confounding factors identified?**
   a. Authors‘ judgement: Unclear.
   b. Support for judgement: Multiple risk factors and different demographic data (e.g. gender, bodyweight, delivery) were stated but not described/treated as cofounders.
6. **Were strategies to deal with confounding factors stated?**
   a. Authors‘ judgement: No.
   b. **Support for judgement:** There was no explicit statistical adjustement for cofounding variables.
7. **Were the outcomes measured in a valid and reliable way?**
   a. Authors‘ judgement: Yes.
   b. Support for judgement: Hearing screening by TEOAE and diagnostic ABR.
8. **Was appropriate statistical analysis used?**
   a. Authors‘ judgement: Yes.
   b. Support for judgement: Logistic regression analysis for ABR results according to risk factors was used.

Overall result: **548 Yes. (50,0%)** **Moderate quality.**

Rivera, 2002

Methods Prospective follow-up cohort study to evaluate hearing loss in children who had symptomatic congenital CMV infection. born between 1966

	and 1997 and evaluated by the investigators at the University of Alabama Hospitals.
**Participants**	**N = 180 infants with congenital CMV infection.**<br>*Initially 190 infants enrolled in prospective follow-up study, 6 infants died in early infancy and 4 infants were lost to follow-up.*

Group I: 87 infants with hearing loss
N (preterm) = 22 infants
N (full-term) = 65 infants

***Group II*:** 93 infants without hearing loss
N (preterm) = 33 infants
N (full-term) = 60 infants

***Inclusion criteria*:**
- children who had symptomatic congenital CMV Infection
- and were born between 1966 and 1997
- and were evaluated by the investigators at the University of Alabama Hospitals

***Exclusion criteria*:** Not stated.

***Characteristics*:**	***Group I:***	***Group II:***
- Race (black/white):	34/53 (39%/61%)	44/49 (47%/53%)
- Gender (f/m):	39/48	42/51
(45%/55%)	(45%/55%)	
- Referral status:		
- referred:	64 (74%)	53 (57%)
-screened:	23 (26%)	40 (43%)
- IUGR:	44 (51%)	30 (32%)
- Petechia:	68 (78%)	50 (54%)
- Jaundice:	49 (56%)	49 (53%)
- Hepatosplenomegaly:	47 (54%)	34 (37%)
- Microcephaly:	46 (53%)	39 (42%)
- Seizures:	3 (3%)	5 (5%)
- ALAT >80 IU/ml:	47/52 (90%)	25/44 (57%)
- Direct bilirubin > 4 mg/dl:	35/60 (58%)	19/57 (33%)
- Thrombocytopenia*:	54/87 (62%)	38/93 (41%)
- Intracranial calcifications:	25/36 (69%)	9/32 (28%)

* IUGR = intrauterine growth retardation; Thrombocytopenia = platelet count <100.000/mm^3

Outcomes

Ascertainment of hearing impairment:
Study children were followed in a special interdisciplinary clinic at the University of Alabama at Birmingham (UAB) and were monitored with serial audiologic evaluations using a standard protocol as described previously. Audiologic evaluations were administered during the newborn period, then every 6 months until 24 months of age, and followed with annual evaluations thereafter.
Full text with mentioned standard protocol was not publicly accessible for reviewers but auditory brainstem response audiometry (ABR)

	and/or behavioral audiometric evaluations appropriate for child's developmental level were used.
**Notes**	Study was conducted from 1966-1997 at University of Alabama Hospitals, Univeristy of Alabama School of Medicine, Birmingham, Alabama.<br>Informed consent was obtained from the parents or legal guardians of the study children.<br>***Funding Source:*** This work was supported in part by grants from the National Institutes of Health, the National Institute of Child Health and Human Development (P01 HD10699), the National Institute of Allergy and Infectious Diseases (P01 AI43681), the National Institute on Deafness and Other Communication Disorders (R01 DC04163 and R01DC02139), and the General Clinical Research Center (M01R00032).<br>***Declaration of interest:*** Not stated.

Risk of bias (Newcastle-Ottawa Quality Assessment Scale)

Bias	***Authors' judgement***	***Support for judgement***
Selection	3/4*	
- Representativeness of exposed cohort	1/1*	Selected group, only preterm born infants with symptomatic cCMV infection in Alabama.
- Selection of the non-exposed cohort	1/1*	Drawn from the same community as exposed cohort.
- Ascertainment of exposure	1/1*	Demographic data and clinical findings at birth were collected from maternal and newborn hospital records and from parents at the time of initial evaluation of the subjects.
- Demonstration that outcome of interest was not present at start of study	0/1*	Not stated.
Comparability	0/2*	
- Comparability of cohorts on the basis of the design or analysis	0/2*	Study controls for presence of symptomatic congenital CMV infection.<br>No comparability because there are no information about e.g. grade of microcephalie, grade of prematurity, medication and complications of prematurity are available. Pre- and full-term infants cannot be adequately compared based on available information.

Outcome	3/3*	
- Assessment of outcome	1/1*	Study children were followed in a special interdisciplinary clinic at the University of Alabama at Birmingham (UAB) and were monitored with serial audiologic evaluations using a standard protocol as described previously.
- Was follow-up long enough for outcomes to occur	1/1*	Yes, audiologic evaluations were administered during the newborn period, then every 6 months until 24 months of age, and followed with annual evaluations thereafter.
- Adequacy of follow up of cohorts	1/1*	From initially 190 enrolled infants, 4 infants were lost to follow-up and 6 infants died during early infancy (180/190=94.7%).
Overall result:	6/9*	**Moderate quality.**

Robertson, 2006

Methods Prospective, longitudinal outcome study to determine relationship between ototoxic drugs and 4-year sensorineural hearing loss (SNHL) in survivors of severe neonatal respiratory failure.

Participants **N = 81 infants with neonatal respiratory failure**

Initially 122 infants, 32 infants died, 9 were lost to follow up.

Group I: **43 infants with sensorineural hearing loss**
N (preterm infants) = 4 infants
N (full-term born infants) = 39 infants

Group II: **38 infants without sensorineural hearing loss**
N (preterm infants) = 10 infants
N (full-term born infants) = 28 infants

Inclusion criteria:
- born January 1994 through April 1996
- survivors from one of the nine Canadian sites as part of the multicenter North American trial of INO

Characteristics:	***Group I:***	***Group II:***
- Sex (m/f):	18/25 (41.9%/58.1%)	17/21 (44.7%/55.3%)
- CHD*:	0 (0%)	15 (34.9%)
- PPHD*:	5 (11.63%)	6 (16%)
- Pneumonia/Sepsis:	4 (9.3%)	4 (11%)
- Meconium aspiration syndrome:	17 (39.5%)	23 (61%)
- RDS*:	2 (4.7%)	4 (11.0%)
- Pulmonary hypoplasia:	0 (0.0%)	1 (2.6%)
- Inhaled NO:	22 (51.16%)	22 (58%)

	- ECMO:	21 (48.8%)	12 (32%)
	- Duration of mechanical ventilation > 7 days:	38 (88.4%)	28 (74%)
	- Neonatal seizures:	9 (20.9%)	7 (18.0%)
	- Intracranial abnormality*:	6 (14.0%)	2 (5.0%)
	- Hospitalization > 21 d:	38 (88.4%)	20 (53%)
	- Maximum bilirubin >200 umol/l:	8 (18.6%)	3 (8%)
	- Aminoglykosides, >7d:	16 (37.2%)	20 (53%)
	- Diuretics, >14d:	29 (67.4%)	7 (18%)
	- Neuromuscular blockers, > 14d:	27 (62.8%)	7 (18%)
	- Vancomycin, > 7d:	18 (41.9%)	6 (16%)

*CHD = congenital diaphragmatic hernia; *PPHD = Primary persistent pulmonary hypertension of newborn; *on post-treatment ultrasound; *RDS = Respiratory distress syndrome

Outcomes

Ascertainment of hearing impairment:

- ***Early audiologic assessment:*** As part of the trial, the only test of hearing required before age 4 years was that done in a soundproof room by experienced certified audiologists at 18-24 months.
- ***Audiologic assessments at 4 years:*** Hearing examinations were administered in soundproof environments by certified audiologists with pediatric experience at eight Canadian sites. A 4-year comprehensive audiologic assessment included bilateral pure-tone responses within the speech range at 250 through 4000 Hz as available from all sites with earphones, bone conduction, and tympanometry.

Notes

Study participants were enrolled from nine Canadian sites including two ECMO centers as part of the multicenter North American trial of INO.

Ethics Boards approvals were obtained as part of the original study and again for retrospective chart reviews from all participating institutions. Individual parental consents were obtained.

All parents or guardians signed consent.

Funding Source: Supported by an operating grant from the Medical Research Council of Canada (#MT-12979).

Declaration of interest: Not stated.

Risk of bias (Newcastle-Ottawa Quality Assessment Scale)

Bias	***Authors' judgement***	***Support for judgement***
Selection	3/4*	
- Representativeness of exposed cohort	0/1*	Selected group, only preterm infants who survived respiratory failure and were treated with loop diuretics,

		aminoglycosides, vancomycin and/or neuromuscular blockers.
- Selection of the non-exposed cohort	1/1*	Drawn from the same community as the exposed cohort.
- Ascertainment of exposure	1/1*	In addition to the data available as part of the original trial, pharmacologic and ventilatory variables were abstracted from neonatal records by one research nurse who visited each participating site. But ascertainment of gestational age was not explicitly stated.
- Demonstration that outcome of interest was not present at start of study	1/1*	Not demonstrated.
Comparability	0/2*	
- Comparability of cohorts on the basis of the design or analysis	0/2*	Study controls for survival of severe respiratory failure in preterm and full-term born infants.<br>No comparability because there are no information about e.g. grade of microcephalie, grade of prematurity, medication and complications of prematurity are available. Pre- and full-term infants cannot be adequately compared based on available information.
Outcome	2/3*	
- Assessment of outcome	0/1*	Audiologic assessments at 4 years: Hearing examinations were administered in soundproof environments by certified audiologists with pediatric experience at eight Canadian sites.
- Was follow-up long enough for outcomes occur	1/1*	Yes, assessments at 18-24 to months and at age 4 years.
- Adequacy of follow up of cohorts	1/1*	9 of 90 infants (=10%) were lost to follow-up.
Overall result:	5/9*	**Moderate quality.**

Seneviratne, 2022

**Methods**	Retrospective cohort study to identify the birth and neurodevelopmental outcomes of neonates diagnosed with symptomatic and asymptomatic cCMV in an Australian tertiary referral hospital stratified by gestation.

Participants

N = 45 infants with cCMV infection

Group I: 8 infants with SNHL
N (preterm) = 3 infants
N (full-term) = 5 infants

Group II: 37 infants without SNHL
N (preterm) = 23 infants
N (full-term) = 14 infants

Inclusion criteria:
- cCMV cases diagnosed at the TUH between 1 January 2005 and 1 January 2020.
- neonates with positive CMV PCR or IgM within first 3 weeks of life
- infants who failed newborn hearing screening with positive Guthrie CMV PCR status.

Exclusion criteria:
- pCMV detected through positive CMV PCR beyond 3 weeks of life.

Characteristics:	Term group	Preterm group
- GA ≤ 1500g	2/19 (10.5%)	18/26 (69.3%)
- CMV infection		
- asymptomatic:	6/19 (31.6%)	4/26 (15.4%)
- symptomatic:	13/19 (68.4%)	22/26 (84.6%)
- Comorbidity in neonatal period:	6/19 (31.6%)	26/26 (100%)
- SNHL:	5/19 (26.3%)	3/26 (11.6%)

Outcomes

Ascertainment of SNHL:
- SNHL was assessed by evaluating audiometry reports (including transient-evoked, otoacoustic emission, brainstem-evoked response audiometry and tympanometry) during follow-up.

Notes

Study was conducted at Townsville University Hospital (TUH), Australia between January 2005 and January 2020.
Electronic data were obtained from the Pathology Queensland Health Support Queensland data custodian from the AUSLAB database for patients admitted to TUH from 1 January 2005 to 1 January 2020.

Funding Source: Project was not funded.

Declaration of interest: None declared.

Risk of bias (Newcastle-Ottawa Quality Assessment Scale)

Bias	***Authors' judgement***	***Support for judgement***
Selection	2/4*	
- Representativeness of exposed cohort	0/1*	Selected group of users, only preterm infants with cCMV infection.

- Selection of the non-exposed cohort	1/1*	Drawn from the same community as the exposed cohort.
- Ascertainment of exposure	1/1*	Review of integrated electronic medical records and hardcopy records.
- Demonstration that outcome of interest was not present at start of study	0/1*	Not stated.
Comparability	1/2*	
- Comparability of cohorts on the basis of the design or analysis	1/2*	Study controls for presence of congenital CMV infection in preterm and full-term born infants, as well as for comorbidities and symptomatic/asymptomatic cCMV infection.
Outcome	0/3*	
- Assessment of outcome	0/1*	Evaluation of audiometry reports.
- Was follow-up long enough for outcomes to occur	0/1*	No, one follow-up to 24 months not definitively long enough to occur.
- Adequacy of follow up of cohorts	0/1*	Follow-up rate <80%, 22/45 (48.8%) infants were lost to-follow up within first 2 years of life.
Overall result:	**3/9***	**Low quality.**

Van Dommelen, 2010

**Methods**	Retrospective cohort study to assess which infants characteristics and specialized procedures are risk indicators for unilateral or bilateral hearing loss (HL).<br>For 2002–2005, data from the NICU hearing screening database in the Netherlands were matched with the national neonatology database in which all NICU infants with their patient characteristics and specialized procedures are registered.	
**Participants**	**N = 10818 infants with NICU stay** *<br>*Initially 10830 infants eligible, for analysis of hearing impairment only subset of 10818 infants was used.*	
	Group I:	199 infants with hearing loss<br>*N (preterm)= 124 infants*<br>*N (full-term) =75 infants*
	Group II:	10619 infants without hearing loss<br>*N (preterm)= 7531 infants*<br>*N (full-term) =3088 infants*

* The exact dates and distribution are taken from an e-mail correspondence with the author

Paula van Dommelen and are not reproduced in the full text.

Inclusion criteria:	- infants from the NICU who survived the admission period from 2002 - 2005<br>- infants that could be matched with a linkage score of >14 across the dimensions date of birth, gender, birth weight in grams, four digit postal code, gestational age in weeks and remaining days, centre code, NICU admission and survival.	
Characteristics:	***Group I:***	***Group II:***
- Gestational age		
- <37 weeks	124/7655 (1.6%)	7531/7655 (=98.4%)
- ≥37 weeks	75/3163 (2.4%)	3088/3163 (=97.6%)
- Birthweight (kg):	3.1 (SD 0.7)	3.2 (SD 0.6)
- Sex (f/m):	32 (42.7%)/43(57.3%)	118(43.2%)/155(56.8%)
- Age at first surgery (d):	35.8 (SD 48.5)	44.9 (SD 55.5)

**Outcomes**	***Ascertainment of hearing loss***<br>- AABR test results from matched databases.
**Notes**	This study made use of data from the NICU Hearing Screening Database (NHSD) in the Netherlands.<br>***Funding Source:*** Not stated.<br>***Declaration of interest:*** None.

Risk of bias (Newcastle-Ottawa Quality Assessment Scale)

Bias	***Authors' judgement***	***Support for judgement***
Selection	3/4*	
- Representativeness of exposed cohort	1/1*	Somewhat representative, NICU infants who survived admission period.
- Selection of the non-exposed cohort	1/1*	Drawn from the same community as the exposed cohort.
- Ascertainment of exposure	1/1*	Data from NICU hearing screening database in the Netherlands were matched with the national neonatology database.
- Demonstration that outcome of interest was not present at start of study	0/1*	No.
Comparability	0/2*	
- Comparability of cohorts on the basis of the design or analysis	0/2*	Study controls for presence of NICU stay in preterm and full-term born infants.<br>No comparability because there are no information about e.g.

		grade of prematurity, medication and complications of prematurity are available. Pre- and full-term infants cannot be adequately compared based on available information.
Outcome	1/3*	
- Assessment of outcome	0/1*	Medical records, data from the NICU Hearing Screening Database matched with National Neonatology Database.
- Was follow-up long enough for outcomes to occur	0/1*	Newborn hearing screening not adequate for detection of hearing loss caused by NICU stay.
- Adequacy of follow up of cohorts	1/1*	241/11071 (2.2%) infants were excluded for incomplete data. Follow-up rate 97.8%.
Overall result:	**4/9***	**Low quality.**

Wang, 2021

Methods

Prospective cohort study to examine the prevalence of late-onset hearing loss and associated risk factors in children with likely asymptomatic cCMV infection in Shandong province in China.
Screening for cCMV infection was carried out in newborns within 1 week after birth in three hospitals from 2011 to 2015 in Pingyin and Yinan Counties of Shandong province, China.

Participants

N = 141 infants

Initially 155 infants, 14 infants were lost to follow-up.

Group I: 4 infants with hearing impairment
N (preterm) = 0 infants
N (full-term) = 4 infants

Group II: 137 infants without hearing impairment
N (preterm) = 4 infants
N (full-term) = 133 infants

Inclusion criteria: - Newborns positively screened for CMV within 1 week after birth from 2011-2015 in Pingyin and Yinan Counties of Shandong province, China by securing more than 5 copies of CMV DNA per PCR reaction of saliva specimens collected within 7 days after birth._

Characteristics:	***Group I:***	***Group II:***
- Gestational age		
- <37 weeks	0/4 (0%)	4/137 (2.9%)

- ≥37 weeks	4/4 (100%)	133/137 (97.1%)
- Sex (m/f):	2(50%)/2(50%)	67(50.4%)/70(49.6%)
- IUGR (yes/no):	0(0%)/4(100%)	5(3.6%)/132(96.4%)

Outcomes

Ascertainment of hearing loss

- initial automated auditory brainstem response (ABR) hearing screen within 1 week after birth
- comprehensive audiologic evaluation:
    - <3 years: ABR test
    - >3 years: DPOAEs/TOAEs
    - tympanometry test to assess integrity of the middle ear

Notes

Children were enrolled as likely asymptomatic based on the absence of microcephaly, petechia and seizure. Neither brain imaging nor ophthalmic assessments were performed.

Funding Source: Supported by a cooperative agreement funded by the US Centers for Disease Control and Prevention to Chinese Center for Disease Control and Prevention, and supported by the Taishan Scholar Program at Shandong Provincial Center for Disease Control and Prevention.

Declaration of interest: Authors have no conflicts of interest to disclose.

Risk of bias (Newcastle-Ottawa Quality Assessment Scale)

Bias	***Authors' judgement***	***Support for judgement***
Selection	2/4*	
- Representativeness of exposed cohort	0/1*	Selected group of users, only infants with likely asymptomatic CMV infection.
- Selection of the non-exposed cohort	1/1*	Drawn from the same community as the exposed cohort.
- Ascertainment of exposure	0/1*	No description.
- Demonstration that outcome of interest was not present at start of study	1/1*	Yes, initial hearing screening (ABR) one week after birth.
Comparability	0/2*	
- Comparability of cohorts on the basis of the design or analysis	0/2*	Study controls for presence of cCMV infection in preterm and full-term born infants. No comparability because there are no information about e.g. grade of prematurity, medication and complications of prematurity are available. Pre- and full-term infants

		cannot be adequately compared based on available information.
Outcome	2/3*	
- Assessment of outcome	1/1*	Hearing test with typmanometry, ABR, TOAEs, DPOAEs.
- Was follow-up long enough for outcomes to occur	0/1*	No, only annually up to 4 years.
- Adequacy of follow up of cohorts	1/1*	141/155 (91%) infants were eligible to follow-up, follow-up rate 97.8%.
Overall result:	**4/9***	**Low quality.**

Wu, 2022

Methods

A retrospective cohort study to evaluate the correlation of neonatal parameters with late-onset sensorineural hearing loss (SNHL) and vestibular dysfunction in infants born from 2009-2015 with congenital cytomegalovirus (cCMV) infection in Taiwan.
Study used data from the National Health Insurance Research Database (NHIRD) in Taiwan.

Participants

N = 5893 infants

Initially 64823 infants were examined, but to answer the question about differences in hearing impairment in preterm and full-term born infants only the subgroup of 5893 infants with cCMV infection was analyzed.

Group I: **5893 infants with congenital CMV infection**
N (preterm infants) = 744 infants
N (full-term infants) = 5149 infants

Group II: 58930 matched controls without CMV infection.

Inclusion criteria:
- infants diagnosed with cCMV infection
- follow up period of at least 3 years

Exclusion criteria: Stillbirth, a non-Taiwanese mother, death before 2 years of age, presence of mental retardation, presence of a congenital brain defect, CMV-infection diagnosed at 3 months old or more, blindness or deafness before index date, any study event before index date, non-matched infants.

Characteristics:	***Group I:***	***Group II:***
- Gestational age:		
o <32 w	63	630
o 32-37 w	681	6810
o 37-41 w	5007	50070
o >41 w	142	1420

	- Gender (m/f):	3310/2583	33100/25830
	- Birthweight:		
	o <1500g:	114	527
	o 1500-2500g:	601	4524
	o 2500-3500g:	4385	45301

Outcomes

Ascertainment of hearing loss *(diagnosis of SNHL):*

- the receipt of a diagnosis of SNHL according to the related ICD-9 or ICD-10 diagnostic codes
- the arrangement of a brainstem auditory evoked response or otoacoustic emission test before the presence of SNHL-related diagnostic codes
- a diagnosis that was made by an otorhinolaryngologist

Notes

Study is using the National Health Insurance Research Database (NHIRD) in Taiwan.
Study is adhered to the 1964 Declaration of Helsinki and its later amendment and was approved by both the local Institutional Review Board of Chung Shan Medical University (Project identification code: CS1-20108) and the National Health Insurance Administration.

Funding Source: Not stated.

Declaration of interest: The authors have no proprietary or commercial interest in any materials mentioned in this article.

Risk of bias (Newcastle-Ottawa Quality Assessment Scale)

Bias	***Authors' judgement***	***Support for judgement***
Selection	3/4*	
- Representativeness of exposed cohort	0/1*	Selected group, only preterm infants with cCMV infection.
- Selection of the non-exposed cohort	1/1*	Full-term born infants drawn from the same community as the exposed cohort.
- Ascertainment of exposure	1/1*	Medical records.
- Demonstration that outcome of interest was not present at start of study	1/1*	Yes, deafness before index date as defined exclusion criteria.
Comparability	0/2*	
- Comparability of cohorts on the basis of the design or analysis	0/2*	Study controls for presence and absence of CMV infection in pre- and full-term born infants.<br>No comparability because there are no information about e.g. grade of prematurity, medication and complications of prematurity are available.<br>Pre- and full-term infants

		cannot be adequately compared based on available information.
Outcome	1/3*	
- Assessment of outcome	0/1*	Medical records.
- Was follow-up long enough for outcomes to occur	0/1*	No, only up to 3 years.
- Adequacy of follow up of cohorts	1/1*	Complete follow up.
Overall result:	4/9*	**Low quality.**

Yamamoto, 2011

Methods Prospective cohort study to assess the rate, associated factors and predictors of congenital CMV-induced SNHL in infants.

Participants **N = 85 infants with congenital CMV infection**

Initially 121 infants, 14 infants were lost to follow-up, 22 were not included in the analysis comparing hearing impairment in preterm and full-term born infants.

Group I: 10 infants with SNHL
N(preterm) = 2 infants
N(full-term) = 8 infants

Group II: 75 infants without SNHL
N(preterm) = 23 infants
N(full-term) = 52 infants

Inclusion criteria:
- infants with congenital CMV-infection, identified by detection of CMV DNA in saliva or urine specimens collected within the first weeks of life and confirmed by virus isolation in tissue culture
- born from March 2003 – May 2009

Characteristics:	**Group I:**	**Group II:**
- Prematurity:	2/10 (20%)	23/75 (30.67%)
- Sex (m/f):	3/7 (30/70%)	46/29 (61.3/38.7%)
- Type of cCMV-infection		
- asymptomatic:	4/10 (40%)	71/75 (94.7%)
- symptomatic:	6/10 (60%)	4/75 (5.3%)

Outcomes ***Ascertainment of SNHL:***
- ABR (at least 2 within the first year of life and method for follow-ups prior to the age of 3); pure tone conditioned play audiometry (method for follow-ups afer the age of 3).

Notes Study conducted at MATER, Ribeirão Preto, State of São Paulo, Brazil and Clinical Hospital of Faculty of Medicine of Ribeirão Preto, University of São Paulo, Brazil.

The study was approved by the Research Ethics Committee of the

University Hospital (Processes 4782/2002, and 9145/2004), and written informed consent was obtained from all mothers.

Funding Source: This study was supported by grants from the National Institutes of Health (NIAID AI 49537; Fogarty International Center, R03TW006480 to WJB), (NIDCD DC04162 to SBB) and Fundação de Amparo à Pesquisa do Estado de São Paulo (FAPESP), Brazil, Process number 02/04166-6.

Declaration of interest: No author has any potential conflict of interest to disclose.

Risk of bias (Newcastle-Ottawa Quality Assessment Scale)

Bias	***Authors' judgement***	***Support for judgement***
Selection	2/4*	
- Representativeness of exposed cohort	0/1*	Selected group of preterm infants with cCMV infection.
- Selection of the non-exposed cohort	1/1*	Drawn from the same community as the exposed cohort.
- Ascertainment of exposure	1/1*	Medical records.
- Demonstration that outcome of interest was not present at start of study	0/1*	Not stated.
Comparability	0/2*	
- Comparability of cohorts on the basis of the design or analysis	0/2*	Study controls for presence of cCMV infection in preterm and full-term born infants as inclusion criterion.<br>No comparability because there are no information about e.g. grade of prematurity, medication and complications of prematurity are available. Pre- and full-term infants cannot be adequately compared based on available information.
Outcome	0/3*	
- Assessment of outcome	0/1*	Only two ABR tests.
- Was follow-up long enough for outcomes to occur	0/1*	No, only up to 12 months.
- Adequacy of follow up of cohorts	0/1*	Follow-up rate <80%, audiology results only for 85/121 (=70.2%) infants.
Overall result:	**2/9***	**Low quality.**

Appendix S3. Summary of a Prospective study on CHL, APD and Language Development in Preterm Children

Background and Objectives

Preterm infants (<37 weeks GA) are at increased risk for otitis media with effusion (OME) and conductive hearing loss (CHL). Long-term effects on language development and auditory processing disorders (APD) remain insufficiently studied. This prospective study aims to explore these relationships, focusing on ventilation, infections, socioeconomic factors, and treatment pathways.

Study Design

Population (P): Children from birth to 10 years, grouped by gestational age:
- EP (<28 weeks), VP (28–31 weeks), MLP (32–36 weeks), FT (≥37 weeks)

Exposure (E): Prematurity and associated neonatal risk factors (e.g., ventilation, infections)

Outcomes (O):
- Primary: CHL (conductive hearing loss)
- Secondary: auditory processing disorder and/or delayed language disorder (receptive and/ or expressive (vocabulary, phonology, syntax, pragmatics)), neurocognitive outcomes (e.g., ADHD, dyslexia, ASD)

Confounding Variables: socioeconomic risk factors (incl. smoking), hospitalizations, therapy access, number of speech therapy sessions

Treatment Groups

Distinction by OME treatment strategy:
- Watchful waiting
- Guideline-based conservative treatment (e.g., nasal sprays, antihistamines)
- Surgical interventions
  – Tympanostomy tubes (unilateral/bilateral)
  – Adenoidectomy with/without tonsillotomy
- Documentation of timing, frequency, combination and recurrences of OME.

Assessment Timeline
- *Hearing assessments*: regularly from birth, every 3 months until ventilation is stable, then at key developmental stages (6 months, 2 years, 5 years, 7 years and 10 years)
- *Language development*: Annually from age 2
- *APD assessment*: From age 7 using standardized and language-dependent tests. However, objective, language-independent diagnostics provide earlier insight into central auditory development.
    - MMN (Mismatch Negativity) to detect auditory discrimination (applicable from ~6 months)
    - P300/LAEP to detect cognitive processing of auditory input (applicable from ~3 years)
    - CERA to detect central auditory maturation (usable from infancy)

APD Diagnostics

Standard language-based central auditory tests are valid from approx. 7 years. To evaluate multilingual or language-impaired children, objective electrophysiological methods should be included. These methods provide early, language-independent assessment of auditory processing, especially in high-risk or non-cooperative children. These tools are especially valuable in multilingual, developmentally delayed or uncooperative children. While they do not replace formal APD diagnostics, they offer a developmental perspective and enable early risk identification and longitudinal monitoring.

Observation Period

From birth to age 10, enabling systematic tracking of hearing, language, APD, and treatment pathway effects.

Note on Sample Size Calculation:

Loss to follow-up must be considered in the sample size estimation, particularly due to the long observation period until age 10.

Table S1. Binomial logistic regression analysis of predictors for hearing impairment in subgroups of infants with different risk factors (Data of Khairy et al., 2018)

Risk factor	Predictor	OR (95%-CI)	p-value
Perinatal asphyxia	Birth (preterm-fullterm)	1.088 (0.593-1.996)	0.785
	Asphyxia (yes-no)	1.133 (0.363-3.542)	0.830
	Birth * Asphyxia	1.817 (0.448-7.359)	0.403
Mechanical ventilation > 5 days	Birth (preterm-fullterm)	1.076 (0.572-2.024)	0.821
	Mechanical ventilation > 5 d (yes-no)	2.869 (0.988-8.327)	0.053
	Birth * Mechanical ventilation > 5 d	1.569 (0.404-6.090)	0.515
Sepsis	Birth (preterm-fullterm)	0.861 (0.453-1.636)	0.647
	Sepsis (yes-no)	1.133 (0.363-3.542)	0.830
	Birth * Sepsis	2.788 (0.720-10.805)	0.138
Hyperbilirubinemia	Birth (preterm-fullterm)	1.422 (0.760-2.661)	0.271
	Hyperbilirubinemia (yes-no)	2.429 (0.900-6.557)	0.080
	Birth * Hyperbilirubinemia	0.563 (0.156-2.035)	0.381
Use of Aminoglykosides	Birth (preterm-fullterm)	0.722 (0.323-1.617)	0.429
	Aminoglykosides (yes-no)	0.833 (0.344-2.017)	0.686
	Birth * Aminoglykosides	2.551 (0.802-8.114)	0.113
Use of Vancomycin	Birth (preterm-fullterm)	0.939 (0.516-1.710)	0.837
	Vancomycin (yes-no)	1.897 (0.496-7.259)	0.349
	Birth * Vancomycin	5.112 (0.913-28.613)	0.063
Use of Vancomycin with Aminoglycosides	Birth (preterm-fullterm)	0.893 (0.503-1.585)	0.699
	Aminoglykoside + Vancomycin (yes-no)	4.52e-7 (0.000-inf.)	0.989
	Birth * Aminoglykoside + Vancomycin	3.01e+7 (0.000-inf)	0.987
Use of loop diuretics	Birth (preterm-fullterm)	1.147 (0.649-2.025)	0.637
	Loop diuretics (yes-no)	1.000 (0.247-4.050)	1.000
	Birth * Loop diuretics	5.814 (0.654-51.676)	0.114

Table S2. Characteristics of excluded studies (ordered by name of author)

Author	Year	Title	Reason for exclusion
Aarhus, L.	2020	Otitis Media in Childhood and Disease in Adulthood: A 40-Year Follow-Up Study	Gestational age of study population was not sufficiently stated.
Aarhus, L.	2015	Childhood Otitis Media: A Cohort Study With 30-Year Follow-Up of Hearing (The HUNT Study)	Gestational age of study population was not sufficiently stated.
Abbott, J.	2017	The Speed of Increasing milk Feeds: a randomised controlled trial	Study protocol as unsuitable study type.
Abdalla, F. M.	2011	The role of the health system in the prevention of hearing loss among children in Sub-Saharan Africa	Review, sources screened for suitable literature for review question.
Abdel Mohsen, A. H.	2013	Risk factors and outcomes of persistent pulmonary hypertension of the newborn in neonatal intensive care unit of Al-minya university hospital in egypt	Connection of risk factors and/or hearing outcome depending on gestational age was not established.
Abdel-Aziz, M.	2014	Eosinophilic granuloma of the temporal bone in children	Gestational age of study population was not sufficiently stated.
Abdollahi, F. Z.	2016	Auditory Brainstem Response Improvements in Hyperbillirubinemic Infants	No comparison of hearing impairment between preterm and full-term born infants.
Abdullah, A.	2020	The Prevalence of Hearing Loss Among Babies in the Neonatal Intensive Care Unit in a Tertiary Hospital in Malaysia	Connection of risk factors and/or hearing outcome depending on gestational age was not established.
Abdullahi, A.	2021	Neonatal Hearing Screening, with Otoacoustic Emission, among Normal Babies in a Northeastern Nigerian Hospital	No comparison of hearing impairment between preterm and full-term born infants.
Abed, A. B.	2013	[Early hearning screening by otoacoustic emissions and auditory brain stem response in Nabeul]	Gestational age of study population was not sufficiently stated.
Abolfotouh, M. A.	2000	The pattern of hearing impairment among schoolboys in an Institute for deaf subjects	Gestational age of study population was not sufficiently stated.
Abou-Elhamd, K. E.	2006	Prevalence of middle ear pathologies in children with bilateral sensorineural hearing loss	Connection of risk factors and/or hearing outcome depending on gestational age was not established.
Abramov, D. M.	2018	Auditory brainstem function in microcephaly related to Zika virus infection	Gestational age of study population was not sufficiently stated.
Abtibol-Bernardino, M. R.	2020	Neurological Findings in Children without Congenital Microcephaly Exposed to Zika Virus in Utero: A Case Series Study	Hearing outcome not examined.
Abu-Shaheen, A.	2014	Prevalence and risk factors of hearing loss among infants in Jordan: Initial results from universal neonatal screening	Connection of risk factors and/or hearing outcome depending on gestational age was not established.
Abuga, J. A.	2022	Neurological impairment and disability in children in rural Kenya	Connection of risk factors and/or hearing outcome depending on gestational age was not established.
Acar, B.	2015	Comparison of risk factors in newborn hearing screening in a developing country	Gestational age of study population was not sufficiently stated.
Acil, D.	2015	Screening of Visually Impaired Children for Health Problems	Gestational age of study population was not sufficiently stated.
Acke, F. R. E.	2022	Twelve years of neonatal hearing screening: audiological and etiological results	Gestational age of study population was not sufficiently stated.
Acke, F. R. E.	2021	Congenital Unilateral Hearing Loss: Characteristics and Etiological Analysis in 121 Patients	Gestational age of study population was not sufficiently stated.
Acosta, E.	2020	Advances in the Development of Therapeutics for Cytomegalovirus Infections	Connection of risk factors and/or hearing outcome depending on gestational age was not established.
Adachi, N.	2010	Risk Factors for Hearing Loss After Pediatric Meningitis in Japan	Gestational age of study population was not sufficiently stated.
Adachi, N.	2010	Etiology and one-year follow-up results of hearing loss identified by screening of newborn hearing in Japan	Gestational age of study population was not sufficiently stated.
Adams-Chapman, I.	2018	Neurodevelopmental Impairment Among Extremely Preterm Infants in the Neonatal Research Network	No comparison of hearing impairment between preterm and full-term born infants.
Adams, D. J.	2016	Otitis Media and Related Complications Among Children with Autism Spectrum Disorders	Gestational age of study population was not sufficiently stated.
Adedeji, T. O.	2015	Management challenges of congenital & early onset childhood hearing loss in a sub-Saharan African country	Gestational age of study population was not sufficiently stated.
Adegbiji, W. A.	2018	Pattern of tympanic membrane perforation in a tertiary hospital in Nigeria	Gestational age of study population was not sufficiently stated.

Adetayo, O. A.	2015	Pediatric Cranial Vault Fractures: Analysis of Demographics, Injury Patterns, and Factors Predictive of Mortality	Gestational age of study population was not sufficiently stated.
Admiraal, R. J.	2000	Changes in the aetiology of hearing impairment in deaf-blind pupils and deaf infant pupils at an institute for the deaf	Connection of risk factors and/or hearing outcome depending on gestational age was not established.
Admiraal, R. J.	1999	Causes of hearing impairment in deaf pupils with a mental handicap	Connection of risk factors and/or hearing outcome depending on gestational age was not established.
Afshar, P. J.	2022	Determination risk factors for severe and profound hearing loss in child candidates for cochlear implantation in southeast of Iran during 2014-2020	No comparison of hearing impairment between preterm and full-term born infants.
Agwu, A. G.	2006	Nontypeable Haemophilus influenzae meningitis complicated by hearing loss in a 9-year-old HIV-infected boy	Case report as unsuitable study type.
Ahlfors, K.	1983	Congenital cytomegalovirus infection: on the relation between type and time of maternal infection and infant's symptoms	Gestational age of study population was not sufficiently stated.
Ahmad, J.	2018	A DESCRIPTIVE RESEARCH ON BACTERIAL MENINGITIS AND IMPAIRMENT IN HEARING IN THE PERSPECTIVE OF ASSOCIATED RISK FACTORS	Gestational age of study population was not sufficiently stated.
Ahmed, A.	2013	Follow-Up of Cases of Haemophilus influenzae Type b Meningitis to Determine Its Long-Term Sequelae	Gestational age of study population was not sufficiently stated.
Ahmed, S.	2017	Risk factors and management modalities for sudanese children with hearing loss or hearing impairment done in Aldwha and Khartoum ENT hospitals, Sudan	No comparison of hearing impairment between preterm and full-term born infants.
Aidan, D.	1999	Auditory screening in neonates by means of transient evoked otoacoustic emissions: a report of 2,842 recordings	Connection of risk factors and/or hearing outcome depending on gestational age was not established.
Airlangga, T. J.	2019	Hearing Loss in Infant with Congenital Cytomegalovirus Infection	Case report as unsuitable study type.
Akpan, U. S.	2022	Congenital Cytomegalovirus Infection	Gestational age of study population was not sufficiently stated.
Al Khabori, M.	2004	Causes of severe to profound deafness in Omani paediatric population	Connection of risk factors and/or hearing outcome depending on gestational age was not established.
Al Muhaimeed, H.	1997	Hearing loss and herpes simplex	Gestational age of study population was not sufficiently stated.
Al-Balas, H. I.	2021	The effects of mode of delivery, maternal age, birth weight, gender and family history on screening hearing results: A cross sectional study	Connection of risk factors and/or hearing outcome depending on gestational age was not established.
Al-Dahhana, J.	2003	'Developmental risks and protective factors for influencing cognitive outcome at 5 1/2 years of age in very-low-birthweight children'	Review, sources screened for suitable literature for review question.
Al-Khatib, T.	2010	Cisplatinum ototoxicity in children, long-term follow up	Gestational age of study population was not sufficiently stated.
Al-Malky, G.	2015	High-frequency audiometry reveals high prevalence of aminoglycoside ototoxicity in children with cystic fibrosis	Gestational age of study population was not sufficiently stated.
Al-Malky, G.	2011	Aminoglycoside antibiotics cochleotoxicity in paediatric cystic fibrosis (CF) patients: A study using extended high-frequency audiometry and distortion product otoacoustic emissions	Gestational age of study population was not sufficiently stated.
Al-Malky, G.	2014	Normal hearing in a child with the m.1555A>G mutation despite repeated exposure to aminoglycosides. Has the penetrance of this pharmacogenetic interaction been overestimated?	Gestational age of study population was not sufficiently stated.
Alaee, E.	2015	Risk Factors for Sensorineural Hearing Loss Among High-Risk Infants in Golestan Province, Iran in 2010-2011	Connection of risk factors and/or hearing outcome depending on gestational age was not established.
Alan, C.	2021	Maternal hypertension, pre-eclampsia, eclampsia and newborn hearing: A retrospective analysis of 454 newborns	Connection of risk factors and/or hearing outcome depending on gestational age was not established.
Alan, M. A.	2021	Hearing screening outcomes in neonates of SARS-CoV-2 positive pregnant women	Connection of risk factors and/or hearing outcome depending on gestational age was not established.
Alberti, P. W.	1985	Issues in early identification of hearing loss	Connection of risk factors and/or hearing outcome depending on gestational age was not established.
Alde, M.	2022	Hearing outcomes in preterm infants with confirmed hearing loss	No comparison of hearing impairment between preterm and full-term born infants.
Alenazi, A.	2021	The prevalence of hearing loss in children with congenital diaphragmatic hernia: A longitudinal population-based study	Gestational age of study population was not sufficiently stated.
Alifieraki, S.	2022	Delays in diagnosis and treatment initiation for congenital cytomegalovirus infection-Why we need universal screening	Connection of risk factors and/or hearing outcome depending on gestational age was not established.

Alkahtani, R.	2019	Age of identification of sensorineural hearing loss and Characteristics‚ÄØof affected children: Findings from two cross-sectional studies in Saudi Arabia	Gestational age of study population was not sufficiently stated.
Alkhunaizi, E.	2018	Warsaw breakage syndrome: Further clinical and genetic delineation	Connection of risk factors and/or hearing outcome depending on gestational age was not established.
Allen, J. C.	1991	Complications of chemotherapy in patients with brain and spinal cord tumors	Review, sources screened for suitable literature for review question.
Almazroua, A. M.	2020	The association between consanguineous marriage and offspring with congenital hearing loss	Gestational age of study population was not sufficiently stated.
Almishaal, A. A.	2021	Prevalence, risk factors, and audiological characteristics of auditory neuropathy	Gestational age of study population was not sufficiently stated.
AlMuhaimeed, H.	1996	Prevalence of sensorineural hearing loss due to toxoplasmosis in Saudi children: A hospital based study	Gestational age of study population was not sufficiently stated.
AlMuhaimeed, H. S.	1996	Hearing impairment among 'at risk' children	Gestational age of study population was not sufficiently stated.
Almuneef, M.	1998	Childhood bacterial meningitis in Saudi Arabia	Gestational age of study population was not sufficiently stated.
Alsanosi, A. A.	2012	Influenza A (H1N1): a rare cause of deafness in two children	Case report as unsuitable study type.
Alsebayel, M. M.	2018	Congenital Esophageal Atresia and Microtia in a Newborn Secondary to Mycophenolate Mofetil Exposure During Pregnancy: A Case Report and Review of the Literature	Case report as unsuitable study type.
Alshaikh, B.	2014	Coagulase-negative staphylococcus sepsis in preterm infants and long-term neurodevelopmental outcome	No comparison of hearing impairment between preterm and full-term born infants.
Altunyurt, S.	2012	Neonatal outcome of fetuses receiving intrauterine transfusion for severe hydrops complicated by Rhesus hemolytic disease	Connection of risk factors and/or hearing outcome depending on gestational age was not established.
Alvarado-Domenech, L. I.	2022	Early Childhood Neurodevelopmental Outcomes in Children with Prenatal Zika Virus Exposure: A Cohort Study in Puerto Rico	Connection of risk factors and/or hearing outcome depending on gestational age was not established.
Alves, N. R.	2011	Prospective study of Kawasaki disease complications: review of 115 cases	Gestational age of study population was not sufficiently stated.
Amani, S.	2015	Study of Effect of Household Parental Smoking on Development of Acute Otitis Media in Children Under 12 Years	Gestational age of study population was not sufficiently stated.
Amaral, J.	2022	Survival and Neurodevelopmental Outcomes of Premature Infants with Severe Peri-Intraventricular Hemorrhage at 24 Months of Age	Connection of risk factors and/or hearing outcome depending on gestational age was not established.
Amatuzzi, M.	2011	Selective Inner Hair Cell Loss in Prematurity: A Temporal Bone Study of Infants from a Neonatal Intensive Care Unit	Hearing outcome not examined.
Ames, M. D.	1970	Central auditory imperception: a significant factor in congenital rubella deafness	Gestational age of study population was not sufficiently stated.
Amin, S. B.	2009	Hyperbilirubinemia and Language Delay in Premature Infants	Hearing outcome not examined.
Amin, S. B.	2017	Auditory toxicity in late preterm and term neonates with severe jaundice	Gestational age of the study population does not meet defined inclusion criteria.
Amin, S. B.	2017	Chronic Auditory Toxicity in Late Preterm and Term Infants With Significant Hyperbilirubinemia	Gestational age of the study population does not meet defined inclusion criteria.
Amini, E.	2014	Assessment of Hearing Loss by OAE in Asphyxiated Newborns	Connection of risk factors and/or hearing outcome depending on gestational age was not established.
Amir, J.	2016	Follow-up of infants with congenital cytomegalovirus and normal fetal imaging	Connection of risk factors and/or hearing outcome depending on gestational age was not established.
Amir, J.	2014	Treatment of Late-Onset Hearing Loss in Infants With Congenital Cytomegalovirus Infection	Gestational age of the study population does not meet defined inclusion criteria.
Amir, J.	2011	Is lenticulostriated vasculopathy a sign of central nervous system insult in infants with congenital CMV infection?	Connection of risk factors and/or hearing outcome depending on gestational age was not established.
Amir, J.	2010	Treatment of symptomatic congenital cytomegalovirus infection with intravenous ganciclovir followed by long-term oral valganciclovir	Gestational age of study population was not sufficiently stated.
Amoils, M.	2015	Patterns and Predictors of Sensorineural Hearing Loss in Children With Congenital Diaphragmatic Hernia	Gestational age of the study population does not meet defined inclusion criteria.
Amorim, R. B.	2009	The maturational process of the auditory system in the first year of life characterized by brainstem auditory evoked potentials	Only investigation of prematurity as risk factor for hearing impairment, other risk factors not analyzed comparing preterm and full-term born infants.
Amorin, M.	2012	[Mucopolysaccharidosis I, Hurler syndrome: a case report]	Case report as unsuitable study type.

Amuzu, C.	2021	Post-Ebola sequelae among Ebola child survivors in Sierra Leone	Gestational age of study population was not sufficiently stated.
Anastasio, A. R. T.	2021	Comprehensive evaluation of risk factors for neonatal hearing loss in a large Brazilian cohort	Gestational age of study population was not sufficiently stated.
Ancora, G.	2007	Cranial ultrasound scanning and prediction of outcome in newborns with congenital cytomegalovirus infection	No comparison of hearing impairment between preterm and full-term born infants.
Anderka, M. T.	2009	Reviewing the Evidence for Mycophenolate Mofetil as a New Teratogen: Case Report and Review of the Literature	Case report as unsuitable study type.
Anderson, P.	2019	Cochlear implantation in children with auditory neuropathy: Lessons from Brown-Vialetto-Van Laere syndrome	Connection of risk factors and/or hearing outcome depending on gestational age was not established.
Andrade, C. L. O.	2019	CONGENITAL HYPOTHYROIDISM AS A RISK FACTOR FOR CENTRAL HEARING PROCESS DISORDERS	Connection of risk factors and/or hearing outcome depending on gestational age was not established.
Andrade, G. M.	2008	Hearing loss in congenital toxoplasmosis detected by newborn screening	Gestational age of study population was not sufficiently stated.
Andrade, J. Q.	2006	Rubella in pregnancy: intrauterine transmission and perinatal outcome during a Brazilian epidemic	Gestational age of study population was not sufficiently stated.
Angrisani, R. M.	2013	Electrophysiological characterization of hearing in small for gestational age premature infants	No comparison of hearing impairment between preterm and full-term born infants.
Anjos, L. P.	2004	Audiologic late prognosis due to meningitis in children	No comparison of hearing impairment between preterm and full-term born infants.
Annapoorna, V.	1996	Evaluation of auditory system in preschool children whose mothers had mid-second trimester amniocentesis	Connection of risk factors and/or hearing outcome depending on gestational age was not established.
Annelies, K.	2021	Results of a multicenter registry for congenital cytomegalovirus infection in Flanders, Belgium: From prenatal diagnosis over neonatal management to therapy	Gestational age of study population was not sufficiently stated.
Ansari, I.	2011	Culture proven bacterial meningitis in children: agents, clinical profile and outcome	Gestational age of study population was not sufficiently stated.
Anteunis, L. J.	1998	Otoacoustic emissions in screening cleft lip and/or palate children for hearing loss--a feasibility study	Gestational age of study population was not sufficiently stated.
Anteunis, L. J.	1999	A longitudinal study of the validity of parental reporting in the detection of otitis media and related hearing impairment in infancy	Investigation of validity of parental opinion as a diagnostic tool for hearing disorders.
Antinmaa, J.	2021	Continuous positive airway pressure treatment may negatively affect auditory maturation in preterm infants	No comparison of hearing impairment between preterm and full-term born infants.
Antonelli, P. J.	2011	Otologic and audiologic outcomes with the Furlow and von Langenbeck with intravelar veloplasty palatoplasties in unilateral cleft lip and palate	Gestational age of study population was not sufficiently stated.
Antoni, M.	2016	Newborn hearing screening: Prevalence and medical and paramedical treatment of bilateral hearing loss in a neonatal series in the Ile-de-France region of France	Only investigation of prematurity as risk factor for hearing impairment, other risk factors not analyzed comparing preterm and full-term born infants.
Anvar, B.	1984	Hearing loss and congenital rubella in Atlantic Canada	Gestational age of study population was not sufficiently stated.
Anyanwu, E. C.	2003	Neurophysiological effects of chronic indoor environmental toxic mold exposure on children	Gestational age of study population was not sufficiently stated.
Apaydin, F.	1998	[Hereditary deafness in Turkey. Initial results]	Gestational age of study population was not sufficiently stated.
Apostolopoulos, N. K.	1999	Otoacoustic emission-based hearing screening of a Greek NICU population	Gestational age of study population was not sufficiently stated.
Appelbaum, E. N.	2018	Analysis of risk factors associated with unilateral hearing loss in children who initially passed newborn hearing screening	Gestational age of study population was not sufficiently stated.
Aram, D. M.	1991	Very-low-birthweight children and speech and language development	Hearing outcome not examined separately.
Ardic, C.	2017	Newborn Hearing Screening Outcomes From Rize; Turkey	Gestational age of study population was not sufficiently stated.
Arditi, M.	1998	Three-year multicenter surveillance of pneumococcal meningitis in children: Clinical characteristics, and outcome related to penicillin susceptibility and dexamethasone use	Gestational age of study population was not sufficiently stated.
Arif, A. A.	2016	The association of childhood asthma with mental health and developmental comorbidities in low-income families	Gestational age of study population was not sufficiently stated.
Arjmandi, F.	2012	Prevalence of deafness and hearing screening in newborns in Isfahan	Connection of risk factors and/or hearing outcome depending on gestational age was not established.

Armstrong, G. T.	2011	Survival and long-term health and cognitive outcomes after low-grade glioma	Gestational age of study population was not sufficiently stated.
Armstrong, J. E.	2008	Olfactory function in Australian Aboriginal children and chronic otitis media	Gestational age of study population was not sufficiently stated.
Armstrong, M.	2013	Barriers to early pediatric cochlear implantation	Gestational age of study population was not sufficiently stated.
Arndt, S.	2010	Spectrum of Hearing Disorders and Their Management in Children With CHARGE Syndrome	Gestational age of study population was not sufficiently stated.
Arnold, B.	1995	[Screening program for selection of hearing loss in newborn infants instituted by the European Community]	Connection of risk factors and/or hearing outcome depending on gestational age was not established.
Arora, M.	2000	Congenital diaphragmatic hernia	Gestational age of study population was not sufficiently stated.
Arora, N. K.	2018	Neurodevelopmental disorders in children aged 2-9 years: Population-based burden estimates across five regions in India	Connection of risk factors and/or hearing outcome depending on gestational age was not established.
Arora, S.	2003	Incidence evaluation of snhl in high risk neonates	Only investigation of prematurity as risk factor for hearing impairment, other risk factors not analyzed comparing preterm and full-term born infants.
Arslan, S.	2013	Universal newborn hearing screening; automated transient evoked otoacoustic emissions	Only investigation of prematurity as risk factor for hearing impairment, other risk factors not analyzed comparing preterm and full-term born infants.
Arteta-Acosta, C.	2022	Sequelae at Hospital Discharge in 61 Children With Invasive Meningococcal Disease, Chile, 2009-2019	Gestational age of study population was not sufficiently stated.
Arts, W. F.	1993	X-linked ataxia, weakness, deafness, and loss of vision in early childhood with a fatal course	Gestational age of study population was not sufficiently stated.
Arulkumaran, S.	1991	No evidence of hearing loss due to fetal acoustic stimulation test	Gestational age of study population was not sufficiently stated.
Asbjornsen, A. E.	2005	Impaired auditory attention skills following middle-ear infections	Gestational age of study population was not sufficiently stated.
Asgharzade, S.	2018	A novel missense mutation in GIPC3 causes sensorineural hearing loss in an Iranian family revealed by targeted next-generation sequencing	Gestational age of study population was not sufficiently stated.
Ashamalla, H. L.	1996	Hyperbaric oxygen therapy for the treatment of radiation-induced sequelae in children. The University of Pennsylvania experience	Gestational age of study population was not sufficiently stated.
Askari, S.	2020	Frameshift variance in SLC19A2 gene causing thiamine responsive megaloblastic anemia (TRMA): a case report from Pakistan	Case report as unsuitable study type.
Aslam, M.	2007	CMV-induced neonatal thrombocytopenia: A case report and review of the literature	Case report as unsuitable study type.
Astbury, J.	1990	Neurodevelopmental outcome, growth and health of extremely low-birthweight survivors: how soon can we tell?	No comparison of hearing impairment between preterm and full-term born infants.
Ates, M.	2017	The endothelial nitric oxide synthase (eNOS) polymorphism in otitis media with effusion (OME)	Gestational age of study population was not sufficiently stated.
Attar, M.		The Incidence and Risk Factors of Cisplatin and Carboplatin Ototoxicity in Pediatric Oncology Patients at Tertiary Oncology Center	Gestational age of study population was not sufficiently stated.
Attias, J.	2007	Transient deafness in young candidates for cochlear implants	Case report as unsuitable study type.
Attias, J.	2006	The prevalence of congenital and early-onset hearing loss in Jordanian and Israeli infants	Gestational age of study population was not sufficiently stated.
Augustine, A. M.	2014	Neonatal hearing screening--experience from a tertiary care hospital in southern India	Connection of risk factors and/or hearing outcome depending on gestational age was not established.
Aust, G.	2002	Tinnitus in childhood	Gestational age of study population was not sufficiently stated.
Austeng, M. E.	2013	Otitis media with effusion in children with in Down syndrome	Gestational age of study population was not sufficiently stated.
Austeng, M. E.	2010	Maternal infection with toxoplasma gondii in pregnancy and the risk of hearing loss in the offspring	Gestational age of study population was not sufficiently stated.
Avettand-Fenoel, V.	2013	Congenital Cytomegalovirus Is the Second Most Frequent Cause of Bilateral Hearing Loss in Young French Children	Missing control collective.
Avnstorp, M. B.	2016	Chronic suppurative otitis media, middle ear pathology and corresponding hearing loss in a cohort of Greenlandic children	Gestational age of study population was not sufficiently stated.
Azam, A. Z.	2001	Prenatal diagnosis of congenital cytomegalovirus infection	No comparison of hearing impairment between preterm and full-term born infants.

Aziz, B.	2021	Colligation of Hearing Loss and Chronic Otitis Media	Gestational age of study population was not sufficiently stated.
Aziza, M.	2022	Hearing Impairment in Infants with Asphyxia	Data incoherence and no response received when querying the author, therefore exclusion.
Azubuike, J. C.	1975	[Juvenile spongy dystrophy of CNS with necrosis of the medulla. A. complication of hydroxyquinoline therapy (author's transl)]	Gestational age of study population was not sufficiently stated.
Babac, S.	2007	[Newborn hearing screening]	No comparison of hearing impairment between preterm and full-term born infants.
Bachor, E.	2001	Neural hearing loss in a child with poliomyelitis: a histopathological study	Case report as unsuitable study type.
Baerts, W.	2010	Auditory neuropathy associated with postnatally acquired cytomegalovirus infection in a very preterm infant	Case report as unsuitable study type.
Bafaqeeh, S. A.	1994	Relevant demographic factors and hearing impairment in Saudi children: epidemiological study	Gestational age of study population was not sufficiently stated.
Bagheri, F.		Relationship between risk factors of hearing loss and the results of otoacoustic emission in newborns	Only investigation of prematurity as risk factor for hearing impairment, other risk factors not analyzed comparing preterm and full-term born infants.
Bagolan, P.	2004	Impact of a current treatment protocol on outcome of high-risk congenital diaphragmatic hernia	Connection of risk factors and/or hearing outcome depending on gestational age was not established.
Bagshaw, R. J.	2011	Hearing impairment in otitis media with effusion: A cross-sectional study based in Pokhara, Nepal	Gestational age of study population was not sufficiently stated.
Baille, M. F.	1996	Prevalence, aetiology, and care of severe and profound hearing loss	Gestational age of study population was not sufficiently stated.
Bakhshaee, M.	2008	Hearing impairment in the neonate of preeclamptic women	Gestational age of study population does not meet defined inclusion criteria for prematurity (<37 weeks) and/or full-term birth (≥37 weeks) with cut-off stated at 37 weeks.
Balatsouras, D. G.	2007	Ramsay Hunt syndrome in a 3-month-old infant	Case report as unsuitable study type.
Baldwin, R. L.	1985	Meningitis and sensorineural hearing loss	Gestational age of study population was not sufficiently stated.
Bale, J. F.	2010	Screening Newborns for Congenital Cytomegalovirus Infection	Position paper as unsuitable study type.
Baljosvic, I.	2012	Application of Fascia of the Temporal Muscle and Cartilage of the Auricular Tragus in Myringoplasty in Children	Connection of risk factors and/or hearing outcome depending on gestational age was not established.
Ballacchino, A.	2013	NEWBORN HEARING SCREENING IN SICILY: LESSON LEARNED	Gestational age of study population was not sufficiently stated.
Ballot, D. E.	1992	SPEECH AND HEARING PROBLEMS IN A HIGH-RISK POPULATION	Review, sources screened for suitable literature for review question.
Balogh, E.	2020	Pseudouridylation defect due to DKC1 and NOP10 mutations causes nephrotic syndrome with cataracts, hearing impairment, and enterocolitis	Gestational age of study population was not sufficiently stated.
Ban, J. H.	2006	A clinical analysis of psychogenic sudden deafness	Gestational age of study population was not sufficiently stated.
Bang, E.	1995	Etiologic aspects and orthodontic treatment of unilateral localized arrested tooth-development combined with hearing loss	Case report as unsuitable study type.
Bansal, R.	1997	Bera in high risk children a 5 year hearing evaluation	Case report as unsuitable study type.
Baquero-Artigao, F.	2009	Prolonged treatment with valganciclovir in an infant with congenital cytomegalovirus infection	Case report as unsuitable study type.
Baraky, L. R.	2012	Disabling hearing loss prevalence in Juiz de Fora, Brazil	Gestational age of study population was not sufficiently stated.
Barbero, P.	2004	Acitretin embryopathy: a case report	Case report as unsuitable study type.
Barbi, M.	2003	A wider role for congenital cytomegalovirus infection in sensorineural hearing loss	Gestational age of study population was not sufficiently stated.
Barbi, M.	2006	Multicity Italian study of congenital cytomegalovirus infection	Connection of risk factors and/or hearing outcome depending on gestational age was not established.
Barbosa, M. H. M.	2020	Normal Hearing Function in Children Prenatally Exposed to Zika Virus	Connection of risk factors and/or hearing outcome depending on gestational age was not established.
Barden, T. P.	1980	Newborn brain stem auditory evoked responses and perinatal clinical events	Connection of risk factors and/or hearing outcome depending on gestational age was not established.

Barkai, G.	2014	Universal neonatal cytomegalovirus screening using saliva - Report of clinical experience	No comparison of hearing impairment between preterm and full-term born infants.
Barr, B.	1961	Deafness following maternal rubella. Retrospective and prospective studies	Gestational age of study population was not sufficiently stated.
Barratt, P. S.	1987	Hearing loss attributed to desferrioxamine in patients with beta-thalassaemia major	Gestational age of study population was not sufficiently stated.
Barreira-Nielsen, C.	2016	Progressive Hearing Loss in Early Childhood	Gestational age of study population was not sufficiently stated.
Barrenäs, M.	2000	Ear and hearing in relation to genotype and growth in Turner syndrome	Gestational age of study population was not sufficiently stated.
Barrenas, M. L.	2005	High risk of sensorineural hearing loss in men born small for gestational age with and without obesity or height catch-up growth: A prospective longitudinal register study on birth size in 245,000 Swedish conscripts	Only investigation of prematurity as risk factor for hearing impairment, other risk factors not analyzed comparing preterm and full-term born infants.
Barthold, J. S.	2018	Cryptorchidism in Boys With Cerebral Palsy Is Associated With the Severity of Disease and With Co-Occurrence of Other Congenital Anomalies	Connection of risk factors and/or hearing outcome depending on gestational age was not established.
Bartlett, A. W.	2018	Recognition, treatment, and sequelae of congenital cytomegalovirus in Australia: An observational study	Gestational age of study population was not sufficiently stated.
Bartov, N.	2019	Management of Acute Mastoiditis With Immediate Needle Aspiration for Subperiosteal Abscess	Gestational age of study population was not sufficiently stated.
Bas A. Y.	2011	Pneumococcal meningitis in the newborn period in a prevaccination era: a 10-year experience at a tertiary intensive care unit	No comparison of hearing impairment between preterm and full-term born infants.
Basar, F.	2008	The Distribution of Risk Factors among High Risk Infants who Failed at Hearing Screening	Connection of risk factors and/or hearing outcome depending on gestational age was not established.
Baser, E.	2022	Effects of Obesity on the Auditory Function of Children and Adolescents	Gestational age of study population was not sufficiently stated.
Bashir, Z. E. H.	2013	Phenotypic variability of CLDN14 mutations causing DFNB29 hearing loss in the Pakistani population	Gestational age of study population was not sufficiently stated.
Basjö, S.	2016	Hearing thresholds, tinnitus, and headphone listening habits in nine-year-old children	Gestational age of study population was not sufficiently stated.
Basonbul, R. A.	2020	Audiologic testing in children with Down Syndrome: Are current guidelines optimal?	Gestational age of study population was not sufficiently stated.
Bass, J. K	2016	Hearing Loss in Patients Who Received Cranial Radiation Therapy for Childhood Cancer	Gestational age of study population was not sufficiently stated.
Bass, J. K.	2018	Auditory Outcomes in Patients Who Received Proton Radiotherapy for Craniopharyngioma	Gestational age of study population was not sufficiently stated.
Bass, J. K.	2020	Association of Hearing Impairment With Neurocognition in Survivors of Childhood Cancer	Gestational age of study population was not sufficiently stated.
Basualdo, W.	2004	Invasive Haemophilus influenzae type b infections in children in Paraguay	Gestational age of study population was not sufficiently stated.
Bauman, N. M.	1994	Mondini dysplasia and congenital cytomegalovirus infection	Gestational age of study population was not sufficiently stated.
Baysal, E.	2013	The polymorphisms of the MBL2 and MIF genes associated with Pediatric Cochlear Implant Patients	Gestational age of study population was not sufficiently stated.
Beckung, E.	2008	Probability of walking in children with cerebral palsy in Europe	Connection of risk factors and/or hearing outcome depending on gestational age was not established.
Bedford, H.	2001	Meningitis in infancy in England and Wales: follow up at age 5 years	Connection of risk factors and/or hearing outcome depending on gestational age was not established.
Behfar, M.	2015	Non-total body irradiation myeloablative conditioning with intravenous busulfan and cyclophosphamide in hematopoietic stem cell transplantation for malignant infantile osteopetrosis	Gestational age of study population was not sufficiently stated.
Bell, E. F.	2010	Impact of timing of birth and resident duty-hour restrictions on outcomes for small preterm infants	No comparison of hearing impairment between preterm and full-term born infants.
Bell, E. F.	2022	Mortality, In-Hospital Morbidity, Care Practices, and 2-Year Outcomes for Extremely Preterm Infants in the US, 2013-2018	No comparison of hearing impairment between preterm and full-term born infants.
Bellia, C. G. D.	2020	Brainstem Auditory Evoked Potentials in infants aged 1 to 24 months during a hearing health care service	Connection of risk factors and/or hearing outcome depending on gestational age was not established.
Benavides-Lara, A.	2021	Zika Virus-Associated Birth Defects, Costa Rica, 2016-2018	Connection of risk factors and/or hearing outcome depending on gestational age was not established.

Bener, A.	2005	Is there any association between consanguinity and hearing loss	No comparison of hearing impairment between preterm and full-term born infants.
Benito Orejas, J. I.	2008	[Results of applying a universal protocol for early detection of hypoacusia in newborn infants for 42 months]	No comparison of hearing impairment between preterm and full-term born infants.
Benito-Orejas, J. I.	2017	Etiology of hearing loss in children	Connection of risk factors and/or hearing outcome depending on gestational age was not established.
Benito-Orejas, J. I.	2021	Analysis of risk factors and targeted surveillance for postnatal hearing loss during 25 years of hearing screening	Connection of risk factors and/or hearing outcome depending on gestational age was not established.
Benjamin, D. K.	2006	Neonatal candidiasis among extremely low birth weight infants: risk factors, mortality rates, and neurodevelopmental outcomes at 18 to 22 months	No comparison of hearing impairment between preterm and full-term born infants.
Bent, J. P.	1994	Bacterial meningitis in the pediatric population: paradigm shifts and ramifications for otolaryngology-head and neck surgery	Gestational age of study population was not sufficiently stated.
Bento, R. F.	2005	Auditory brainstem response and otoacoustic emission assessment of hearing-impaired children of mothers who contracted rubella during pregnancy	No comparison of hearing impairment between preterm and full-term born infants.
Berard, A.	2012	Study of the costs and morbidities of late-preterm birth	Only investigation of prematurity as risk factor for hearing impairment, other risk factors not analyzed comparing preterm and full-term born infants.
Berg, A. L.	2011	High frequency hearing sensitivity in adolescent females of a lower socioeconomic status over a period of 24 years (1985-2008)	Gestational age of study population was not sufficiently stated.
Berg, A. L.	1999	Ototoxic impact of cisplatin in pediatric oncology patients	Gestational age of study population was not sufficiently stated.
Berg, A. L.	2005	Newborn hearing screening in the NICU: profile of failed auditory brainstem response/passed otoacoustic emission	Gestational age of study population does not meet defined inclusion criteria for prematurity (<37 weeks) and/or full-term birth (≥37 weeks) with cut-off stated at 37 weeks.
Berg, M.	1981	Sudden deafness and vertigo in children and juveniles	Gestational age of study population was not sufficiently stated.
Berg, S.	2002	Long-term follow-up of children with bacterial meningitis with emphasis on behavioural characteristics	Gestational age of study population was not sufficiently stated.
Bergamaschi, R.	2008	Hearing loss in Turner syndrome: Results of a multicentric study	Gestational age of study population was not sufficiently stated.
Berger, R.	2010	Newborn hearing screening. Experiences and results from Marburg	Only investigation of prematurity as risk factor for hearing impairment, other risk factors not analyzed comparing preterm and full-term born infants.
Bergman, I.	1985	Cause of hearing loss in the high-risk premature infant	No comparison of hearing impairment between preterm and full-term born infants.
Bergstrom, L.	1979	New patterns in genetic and congenital otonephropathies	Case report as unsuitable study type.
Beria, J.	2007	Hearing impairment and socioeconomic factors: a population-based survey of an urban locality in southern Brazil	Gestational age of study population was not sufficiently stated.
Bermudez, B.	2021	Respiratory and otolaryngological disorders in Down syndrome from one center in Brazil	No comparison of hearing impairment between preterm and full-term born infants.
Bernbaum, J.	1995	Survivors of extracorporeal membrane oxygenation at 1 year of age: the relationship of primary diagnosis with health and neurodevelopmental sequelae	No comparison of hearing impairment between preterm and full-term born infants.
Berrettini, S.	1999	Progressive sensorineural hearing loss in childhood	Gestational age of study population was not sufficiently stated.
Berthold, F.	2017	Incidence, Survival, and Treatment of Localized and Metastatic Neuroblastoma in Germany 1979-2015	Gestational age of study population was not sufficiently stated.
Bertolini, P.	2004	Platinum compound-related ototoxicity in children - Long-term follow-up reveals continuous worsening of hearing loss	Gestational age of study population was not sufficiently stated.
Bertolli, J.	2020	Functional Outcomes among a Cohort of Children in Northeastern Brazil Meeting Criteria for Follow-Up of Congenital Zika Virus Infection	Gestational age of study population was not sufficiently stated.
Best, E. J.	2011	Once-daily Gentamicin in Infants and Children A Prospective Cohort Study Evaluating Safety and the Role of Therapeutic Drug Monitoring in Minimizing Toxicity	Gestational age of study population was not sufficiently stated.
Beswick, R.	2019	Integration of congenital cytomegalovirus screening within a newborn hearing screening programme	No comparison of hearing impairment between preterm and full-term born infants.
Beswick, R.	2012	Targeted surveillance for postnatal hearing loss: A program evaluation	No comparison of hearing impairment between preterm and full-term born infants.
Beswick, R.	2013	Which Risk Factors Predict Postnatal Hearing Loss in Children?	Connection of risk factors and/or hearing outcome depending on gestational age was not established.

Beutner, D.	2007	Risk factors for auditory neuropathy/auditory synaptopathy	No comparison of hearing impairment between preterm and full-term born infants.
Beyea, J. A.	2020	Long-Term Incidence and Predictors of Significant Hearing Loss Requiring Hearing Assistive Devices Among Childhood Cancer Survivors: A Population-Based Study	Gestational age of study population was not sufficiently stated.
Bhagat, S.	2013	Time-frequency analysis of transient-evoked otoacoustic emissions in children exposed to carboplatin chemotherapy	Gestational age of study population was not sufficiently stated.
Bhat, J. A.	2018	Targeted Screening for Hearing Impairment in Neonates: A Prospective Observational Study	Gestational age of study population was not sufficiently stated.
Bhati, P.	2019	Cerebral Palsy in North Indian Children: Clinico-etiological Profile and Comorbidities	Gestational age of study population was not sufficiently stated.
Bhatia, K.	2004	Surgical complications and their management in a series of 300 consecutive pediatric cochlear implantations	Gestational age of study population was not sufficiently stated.
Bhatia, P.	2013	Early Identification of Young Children with Hearing Loss in Federally Qualified Health Centers	Gestational age of study population was not sufficiently stated.
Bhattacharya, H.	2018	Brainstem Auditory Evoked Potential in Preterm Infants and its Relation with Gestational Age	Only investigation of prematurity as risk factor for hearing impairment, other risk factors not analyzed comparing preterm and full-term born infants.
Bhoovarahan, S.	2022	Ototacoustic Emissions-Based Hearing Assessment of Neonates in Tertiary Care Hospital	Only investigation of prematurity as risk factor for hearing impairment, other risk factors not analyzed comparing preterm and full-term born infants.
Bianchin, G.	2022	A regional-based newborn hearing screening program: the Emilia-Romagna model after ten years of legislation	Gestational age of study population was not sufficiently stated.
Bielecki, I.	2011	Risk factors associated with hearing loss in infants: An analysis of 5282 referred neonates	Only investigation of prematurity as risk factor for hearing impairment, other risk factors not analyzed comparing preterm and full-term born infants.
Bielecki, I.	2012	Prevalence and risk factors for Auditory Neuropathy Spectrum Disorder in a screened newborn population at risk for hearing loss	Only investigation of prematurity as risk factor for hearing impairment, other risk factors not analyzed comparing preterm and full-term born infants.
Bilavsky, E.	2016	Clinical Implications for Children Born With Congenital Cytomegalovirus Infection Following a Negative Amniocentesis	Connection of risk factors and/or hearing outcome depending on gestational age was not established.
Bilavsky, E.	2015	Hepatic involvement in congenital cytomegalovirus infection - infrequent yet significant	Gestational age of study population was not sufficiently stated.
Bilavsky, E.	2015	Lenticulostriated vasculopathy is a high-risk marker for hearing loss incongenital cytomegalovirus infections	Gestational age of study population was not sufficiently stated.
Bilavsky, E.	2016	Hearing outcome of infants with congenital cytomegalovirus and hearing impairment	Gestational age of study population was not sufficiently stated.
Bilge, N.	2020	Biotinidase deficiency in differential diagnosis of neuromyelitis optica spectrum disorder	Case report as unsuitable study type.
Binnetoglu, A.	2015	Association between Family History and Idiopathic Sudden Sensorineural Hearing Loss	Gestational age of study population was not sufficiently stated.
Birdane, L.	2016	Evaluation of the Vestibular System and Etiology in Children with Unilateral Sensorineural Hearing Loss	Gestational age of study population was not sufficiently stated.
Birman, C. S.	2012	Pediatric Cochlear Implants: Additional Disabilities Prevalence, Risk Factors, and Effect on Language Outcomes	Connection of risk factors and/or hearing outcome depending on gestational age was not established.
Biswas, A. K.	2012	The Potential Risk Factors and the Identification of Hearing Loss in Infants	Gestational age of study population was not sufficiently stated.
Bitner-Glindzicz, M.	2010	Aminoglycoside-induced deafness during treatment of acute leukaemia	Case report as unsuitable study type.
Bitner-Glindzicz, M.	2014	Gentamicin, genetic variation and deafness in preterm children	Study protocol as unsuitable study type.
Blazquez-Gamero, D.	2019	Prevention and treatment of fetal cytomegalovirus infection with cytomegalovirus hyperimmune globulin: a multicenter study in Madrid	No comparison of hearing impairment between preterm and full-term born infants.
Blomqvist, M.	2019	beta-Mannosidosis caused by a novel homozygous intragenic inverted duplication in MANBA	Case report as unsuitable study type.
Bloom, S.	2005	Congenital rubella syndrome burden in Morocco: a rapid retrospective assessment	Connection of risk factors and/or hearing outcome depending on gestational age was not established.
Bluestone, C. D.	1988	Otitis media and congenital perilymphatic fistula as a cause of sensorineural hearing loss in children	Gestational age of study population was not sufficiently stated.
Bluher, A.	2021	Obesity as a Possible Risk Factor for Pediatric Sensorineural Hearing Loss	Gestational age of study population was not sufficiently stated.

Bolton, P. F.	2012	Autism Spectrum Disorder and Autistic Traits in the Avon Longitudinal Study of Parents and Children: Precursors and Early Signs	Gestational age of study population was not sufficiently stated.
Bonfils, P.	1992	SPONTANEOUS AND EVOKED OTOACOUSTIC EMISSIONS IN PRETERM NEONATES	Connection of risk factors and/or hearing outcome depending on gestational age was not established.
Boo, N. Y.	1989	Management of Flavobacterium meningitis in the neonates: experience with 18 consecutive cases	Gestational age of study population was not sufficiently stated.
Boo, N. Y.	1994	Risk factors associated with hearing loss in term neonates with hyperbilirubinaemia	No comparison of hearing impairment between preterm and full-term born infants.
Boo, N. Y.	1996	Comparison of morbidities in very low birthweight and normal birthweight infants during the first year of life in a developing country	Gestational age of study population was not sufficiently stated.
Booth, T. N.	2013	High-resolution 3-D T2-weighted imaging in the diagnosis of labyrinthitis ossificans: emphasis on subtle cochlear involvement	Gestational age of study population was not sufficiently stated.
Boppana, S. B.	2005	Congenital cytomegalovirus infection: Association between virus burden in infancy and hearing loss	No data on hearing impairment in the full-term born control group, therefore exclusion.
Boppana, S. B.	1997	Neuroradiographic findings in the newborn period and long-term outcome in children with symptomatic congenital cytomegalovirus infection	Connection of risk factors and/or hearing outcome depending on gestational age was not established.
Boppana, S. B.	1996	Transplacentally acquired antiviral antibodies and outcome in congenital human cytomegalovirus infection	Gestational age of study population was not sufficiently stated.
Boppana, S. B.	1993	Virus-specific antibody responses in mothers and their newborn infants with asymptomatic congenital cytomegalovirus infections	Gestational age of study population was not sufficiently stated.
Boppana, S. B.	1992	Symptomatic congenital cytomegalovirus infection: neonatal morbidity and mortality	Connection of risk factors and/or hearing outcome depending on gestational age was not established.
Boppana, S. B.	2001	Predictors of hearing loss in children with symptomatic congenital CMV infection	Gestational age of study population was not sufficiently stated.
Borba, E. F.	2004	Chloroquine gestational use in systemic lupus erythematosus: assessing the risk of child ototoxicity by pure tone audiometry	No comparison of hearing impairment between preterm and full-term born infants.
Borkoski Barreiro, S. A.	2013	Results of an early hearing detection program	No comparison of hearing impairment between preterm and full-term born infants.
Borkoski-Barreiro, S. A.	2013	Evaluation of very low birth weight (,â§ 1,500 g) as a risk indicator for sensorineural hearing loss	Gestational age of study population does not meet defined inclusion criteria for prematurity (<37 weeks) and/or full-term birth (≥37 weeks) with cut-off stated at 37 weeks.
Borradori, C.	1997	Risk factors of sensorineural hearing loss in preterm infants	No comparison of hearing impairment between preterm and full-term born infants.
Bosis, S.	2012	Meningitis complicated by subdural empyema and deafness caused by pneumoccoccal serotype 7F in a 17-month-old child: a case report	Case report as unsuitable study type.
Boskabadi, H.	2018	Risk Factors for Sensorineural Hearing Loss in Neonatal Hyperbilirubinemia	No comparison of hearing impairment between preterm and full-term born infants.
Bosnjak, V. M.	2011	Malformations of Cortical Development in Children with Congenital Cytomegalovirus Infection - A Study of Nine Children with Proven Congenital Cytomegalovirus Infection	Case report as unsuitable study type.
Boston, M.	2007	The large vestibular aqueduct: a new definition based on audiologic and computed tomography correlation	Gestational age of study population was not sufficiently stated.
Botelho, C. T.	2014	Increased prevalence of early cochlear damage in young patients with type 1 diabetes detected by distortion product otoacoustic emissions	Gestational age of study population was not sufficiently stated.
Botelho, F. A.	2010	Prevalence of hearing impairment in children at risk	No comparison of hearing impairment between preterm and full-term born infants.
Botelho, M.	2010	Newborn hearing screening in the limiar clinic in Porto Velho - Rondonia	No comparison of hearing impairment between preterm and full-term born infants.
Botti, C.	2020	Different Audiologic Outcomes in Twins with Congenital Cytomegalovirus Infection	Case report as unsuitable study type.
Boudewyns, A.	2009	Cytomegalovirus DNA Detection in Guthrie Cards: Role in the Diagnostic Work-Up of Childhood Hearing Loss	Gestational age of study population was not sufficiently stated.
Boudewyns, A.	2016	Auditory neuropathy spectrum disorder (ANSD) in referrals from neonatal hearing screening at a well-baby clinic	Gestational age of study population was not sufficiently stated.
Boudewyns, A.	2020	Etiological Work-up in Referrals From Neonatal Hearing Screening: 20 Years of Experience	Gestational age of study population was not sufficiently stated.
Boudewyns, A.	2018	Role of Targeted Next Generation Sequencing in the Etiological Work-Up of Congenitally Deaf Children	Gestational age of study population was not sufficiently stated.

Boughman, J. A.	1983	Usher syndrome: definition and estimate of prevalence from two high-risk populations	Gestational age of study population was not sufficiently stated.
Bovo, R.	2015	Is very early hearing assessment always reliable in selecting patients for cochlear implants? A case series study	Case report as unsuitable study type.
Bowen, J. R.	1993	Extremely low birthweight infants at 3 years: a developmental profile	No comparison of hearing impairment between preterm and full-term born infants.
Bowman, M. K.	2011	Appropriate hearing screening in the pediatric patient with head trauma	Gestational age of study population was not sufficiently stated.
Boyle, C. A.	2011	Trends in the Prevalence of Developmental Disabilities in US Children, 1997-2008	Gestational age of study population was not sufficiently stated.
Boynton, B. R.	1986	Ventriculoperitoneal shunts in low birth weight infants with intracranial hemorrhage: neurodevelopmental outcome	No comparison of hearing impairment between preterm and full-term born infants.
Bozzola, E.	2021	Predicting Parameters for Audiological Complications in Pediatric Patients Affected by Meningitis	Gestational age of study population was not sufficiently stated.
Bradford, R. D.	2005	Detection of cytomegalovirus (CMV) DNA by polymerase chain reaction is associated with hearing loss in newborns with symptomatic congenital CMV infection involving the central nervous system	No comparison of hearing impairment between preterm and full-term born infants.
Braegger, C.	1991	Unknown syndrome: ischiadic hypoplasia, renal dysfunction, immunodeficiency, and a pattern of minor congenital anomalies	Case report as unsuitable study type.
Braga, H.	2021	Congenital hypothyroidism as a risk factor for hearing and parents' knowledge about its impact on hearing	Gestational age of study population was not sufficiently stated.
Bramugy, J.	2022	Short- and Long-term Outcomes of Group B Streptococcus Invasive Disease in Mozambican Children: Results of a Matched Cohort and Retrospective Observational Study and Implications for Future Vaccine Introduction	No comparison of hearing impairment between preterm and full-term born infants.
Brennan, K.	2009	Auditory nerve neuropathy in a neonate after linezolid treatment	Case report as unsuitable study type.
Bresolin, A. U.	1983	[Neurological and cerebrospinal fluid evaluation in children with serogroup A meningococcal meningitis]	Connection of risk factors and/or hearing outcome depending on gestational age was not established.
Briand, C.	2016	Outcomes of bacterial meningitis in children	Gestational age of study population was not sufficiently stated.
Britton, H.	1978	Keratosis follicularis spinulosa decalvans. An infant with failure to thrive, deafness, and recurrent infections	Case report as unsuitable study type.
Brobby, G. W.	1988	Causes of congenital and acquired total sensorineural hearing loss in Ghanaian children	Gestational age of study population was not sufficiently stated.
Brodie, K. D.	2022	Outcomes of an Early Childhood Hearing Screening Program in a Low-Income Setting	Gestational age of study population was not sufficiently stated.
Brodin, N. P.	2011	Radiobiological risk estimates of adverse events and secondary cancer for proton and photon radiation therapy of pediatric medulloblastoma	Gestational age of study population was not sufficiently stated.
Brodsky, L.	1985	Sensorineural hearing loss following live measles virus vaccination	Case report as unsuitable study type.
Brody, R.	1999	Parents cannot detect mild hearing loss in children	Gestational age of study population was not sufficiently stated.
Brookes, J. T.	2008	Cochlear implantation in deafness-dystonia-optic neuronopathy (DDON) syndrome	Case report as unsuitable study type.
Brookhouser, P. E.	1991	Unilateral hearing loss in children	Gestational age of study population was not sufficiently stated.
Brookhouser, P. E.	1994	Fluctuating and/or progressive sensorineural hearing loss in children	Gestational age of study population was not sufficiently stated.
Broomfield, S. J.	2014	Results of a prospective surgical audit of bilateral paediatric cochlear implantation in the UK	Gestational age of study population was not sufficiently stated.
Brough, H.	2020	Acquired auditory neuropathy spectrum disorder after malaria treated with quinine	Case report as unsuitable study type.
Broughton, S. J.	1984	A review of Haemophilus influenzae infections in Cambridge 1975-1981	Gestational age of study population was not sufficiently stated.
Brown, D. R.	1991	Neonatal sensorineural hearing loss associated with furosemide: a case-control study	Connection of risk factors and/or hearing outcome depending on gestational age was not established.
Brown, G.	2009	NICU noise and the preterm infant	Review, sources screened for suitable literature for review question.

Brownstein, Z.	1991	Estimated number of loci for autosomal recessive severe nerve deafness within the Israeli Jewish population, with implications for genetic counseling	Gestational age of study population was not sufficiently stated.
Brucker-Davis, F.	1995	Genetic and clinical features of 42 kindreds with resistance to thyroid hormone. The National Institutes of Health Prospective Study	Gestational age of study population was not sufficiently stated.
Brumbaugh, J. E.	2019	Outcomes of Extremely Preterm Infants With Birth Weight Less Than 400 g	No comparison of hearing impairment between preterm and full-term born infants.
Bruno, R.	2015	Even in the era of congenital hypothyroidism screening mild and subclinical sensorineural hearing loss remains a relatively common complication of severe congenital hypothyroidism	No comparison of hearing impairment between preterm and full-term born infants.
Bryant, M. C.	2022	A case of ANCA-associated vasculitis in a 16-year-old female following SARS-COV-2 infection and a systematic review of the literature	Case report as unsuitable study type.
Bubala, H.	2001	[Deafness, as a complication of Pseudomonas aeruginosa septicemia in a 9-year old boy with acute lymphoblastic leukemia]	Case report as unsuitable study type.
Buchanan, L. H.	2011	Environmental lead exposure and otoacoustic emissions in Andean children	Gestational age of study population was not sufficiently stated.
Buckingham, S. C.	2006	Early vancomycin therapy and adverse outcomes in children with pneumococcal meningitis	Gestational age of study population was not sufficiently stated.
Buckingham, S. C.	2001	Pneumococcal meningitis in children: relationship of antibiotic resistance to clinical characteristics and outcomes	Gestational age of study population was not sufficiently stated.
Bucuvalas, J. C.	2003	Risk of hearing impairment in pediatric liver transplant recipients: A single center study	Gestational age of study population was not sufficiently stated.
Bunikowski, R.	1998	Neurodevelopmental outcome after prenatal exposure to opiates	Hearing outcome not examined separately.
Buonfiglio, P. I.	2022	Predicting pathogenicity for novel hearing loss mutations based on genetic and protein structure approaches	Gestational age of study population was not sufficiently stated.
Burg, J. R.	1999	Health effects of environmental contaminant exposure: an intrafile comparison of the Trichloroethylene Subregistry	Gestational age of study population was not sufficiently stated.
Burke, W. F.	2016	Prevalence and audiological profiles of GJB2 mutations in a large collective of hearing impaired patients	Gestational age of study population was not sufficiently stated.
Burnett, A. C.	2018	Biological and Social Influences on the Neurodevelopmental Outcomes of¬†Preterm Infants	Review, sources screened for suitable literature for review question.
BuSaba, N. Y.	2008	Connexin 26 and 30 genes mutations in patients with chronic rhinosinusitis	Gestational age of study population was not sufficiently stated.
Butcher, E.	2020	Risk factors for permanent childhood hearing impairment	No comparison of hearing impairment between preterm and full-term born infants.
Buysse, C. M.	2008	Long-term health status in childhood survivors of meningococcal septic shock	Gestational age of study population was not sufficiently stated.
Byckova, J.	2020	Etiological profile of hearing loss amongst Lithuanian pediatric cochlear implant users	Missing control collective without hearing impairment.
Byeon, H.	2021	Associations between adolescents' earphone usage in noisy environments, hearing loss, and self-reported hearing problems in a nationally representative sample of South Korean middle and high school students	Gestational age of study population was not sufficiently stated.
Byun, H.	2013	Performance after timely cochlear implantation in prelingually deaf children with cerebral palsy	Gestational age of study population was not sufficiently stated.
Caglar, O.	2021	Evaluation of DNA damages in congenital hearing loss patients	Gestational age of study population was not sufficiently stated.
Cai, L. H.	2021	Multi-Center in-Depth Screening of Neonatal Deafness Genes: Zhejiang, China	No comparison of hearing impairment between preterm and full-term born infants.
Calcutt, T. L.	2016	Newborn hearing screening in Queensland 2009-2011: Comparison of hearing screening and diagnostic audiological assessment between term and preterm infants	Only investigation of prematurity as risk factor for hearing impairment, other risk factors not analyzed comparing preterm and full-term born infants.
Calevo, M. G.	2007	Ligurian experience on neonatal hearing screening: clinical and epidemiological aspects	Only investigation of prematurity as risk factor for hearing impairment, other risk factors not analyzed comparing preterm and full-term born infants.
Calisici, E.	2015	Neurodevelopmental outcomes of premature infants with severe intraventricular hemorrhage	No comparison of hearing impairment between preterm and full-term born infants.
Calkoen, E. A. V.	2019	The etiological evaluation of sensorineural hearing loss in children	Gestational age of study population was not sufficiently stated.
Camet, M. L.	2021	Cisplatin Ototoxicity: Examination of the Impact of Dosing, Infusion Times, and Schedules In Pediatric Cancer Patients	Gestational age of study population was not sufficiently stated.

Canale, A.	2006	Age at diagnosis of deaf babies: A retrospective analysis highlighting the advantage of newborn hearing screening	Connection of risk factors and/or hearing outcome depending on gestational age was not established.
Canet, J. M. S.	2018	Otoacoustic emissions in children treated with gentamicin in a secondary hospital	No comparison of hearing impairment between preterm and full-term born infants.
Cannie, M. M.	2016	Congenital cytomegalovirus infection: contribution and best timing of prenatal MR imaging	No comparison of hearing impairment between preterm and full-term born infants.
Caporali, C.	2022	Neurodevelopmental outcome of Italian preterm ELBW infants: an eleven years single center cohort	No comparison of hearing impairment between preterm and full-term born infants.
Cappuccio, G.	2016	New insights in the interpretation of array-CGH: autism spectrum disorder and positive family history for intellectual disability predict the detection of pathogenic variants	Gestational age of study population was not sufficiently stated.
Capretti, M. G.	2014	Role of cerebral ultrasound and magnetic resonance imaging in newborns with congenital cytomegalovirus infection	No comparison of hearing impairment between preterm and full-term born infants.
Carding, P. N.	2006	The prevalence of childhood dysphonia: A cross-sectional study	Gestational age of study population was not sufficiently stated.
Carleton, B.	2009	Adverse drug reaction active surveillance: developing a national network in Canada's children's hospitals	Gestational age of study population was not sufficiently stated.
Carlsson, P. I.	2012	GJB2 (Connexin 26) gene mutations among hearing-impaired persons in a Swedish cohort	Gestational age of study population was not sufficiently stated.
Carlston, C. M.	2019	Extrapolation of Variant Phase in Mitochondrial Short-Chain Enoyl-CoA Hydratase (ECHS1) Deficiency	Case report as unsuitable study type.
Caroca, C.	2017	Rubella in Sub-Saharan Africa and sensorineural hearing loss: a case control study	Gestational age of study population was not sufficiently stated.
Carroll, D. J.	2013	The effect of cleft palate repair technique on hearing outcomes in children	Gestational age of study population was not sufficiently stated.
Carter, J. A.	2006	Severe falciparum malaria and acquired childhood language disorder	Hearing outcome not examined.
Carter, J. A.	2005	Persistent neurocognitive impairments associated with severe falciparum malaria in Kenyan children	Gestational age of study population was not sufficiently stated.
Carter, M. T.	2009	Phenotypic Delineation of Emanuel Syndrome (Supernumerary Derivative 22 Syndrome): Clinical Features of 63 Individuals	No comparison of hearing impairment between preterm and full-term born infants.
Carzoli, R. P.	1991	Evaluation of auditory brain-stem response in full-term infants of cocaine-abusing mothers	No comparison of hearing impairment between preterm and full-term born infants.
Casado-Flores, J.	2006	Clinical data and factors associated with poor outcome in pneumococcal meningitis	Gestational age of study population was not sufficiently stated.
Casali, R. L.	2010	Auditory Brainstem Evoked Response: response patterns of full-term and premature infants	Only investigation of gestational age as risk factor for hearing impairment, other risk factors not analyzed comparing preterm and full-term born infants.
Casella, E. B.	2004	Sequelae from meningococcal meningitis in children: A critical analysis of dexamethasone therapy	Gestational age of study population was not sufficiently stated.
Castel, V.	2014	Ototoxicity: a worrying problem for survivors of high-risk neuroblastoma	Editorial, sources screened for suitable literature for review question.
Castelan-Martinez, O. D.	2014	Hearing loss in Mexican children treated with cisplatin	Gestational age of study population was not sufficiently stated.
Caylan, R.	2006	Prevalence and risk factors of otitis media with effusion in Trabzon, a city in northeastern Turkey, with an emphasis on the recommendation of OME screening	Gestational age of study population was not sufficiently stated.
Cebeci, S.	2020	Impact of the demographic and aetiological factors and intraoperative findings on postoperative outcomes in chronic otitis media surgery	Gestational age of study population was not sufficiently stated.
Celik, T.	2021	Evaluation of cochlear functions in infants exposed to SARS-CoV-2 intrauterine	No comparison of hearing impairment between preterm and full-term born infants.
Celikel, E.	2015	Evaluation of 98 immunocompetent children with cytomegalovirus infection: importance of neurodevelopmental follow-up	Connection of risk factors and/or hearing outcome depending on gestational age was not established.
Center for Disease Control and Prevention (CDC)	2013	Three cases of congenital rubella syndrome in the postelimination era--Maryland, Alabama, and Illinois, 2012	Case report as unsuitable study type.
Chadha, S.	1997	Auditory brainstem responses in high risk and normal newborns	Connection of risk factors and/or hearing outcome depending on gestational age was not established.
Champion, S.	2021	Assessment of Hearing in High Risk Infants, Using Brainstem Evoked Response Audiometry	Only investigation of prematurity as risk factor for hearing impairment, other risk factors not analyzed comparing preterm and full-term born infants.
Chang, J.	2020	Comparison of newborn hearing screening results between well babies and neonates admitted to the	Gestational age of study population was not sufficiently stated.

		neonatal intensive care unit for more than 5 days: Analysis based on the national database in Korea for 9 years	
Chao, C.	2020	Chronic Comorbidities Among Survivors of Adolescent and Young Adult Cancer	Gestational age of study population was not sufficiently stated.
Chao, C.	2016	Long-term Health Outcomes in Survivors of Childhood Cancer Diagnosed Between 1990 and 2000 in a Large US Integrated Health Care System	Gestational age of study population was not sufficiently stated.
Chao, C. K.	2012	High prevalence of hearing impairment in HIV-infected Peruvian children	Only investigation of prematurity as risk factor for hearing impairment, other risk factors not analyzed comparing preterm and full-term born infants.
Chao, T. K.	2006	Distortion product otoacoustic emissions as a prognostic factor for idiopathic sudden sensorineural hearing loss	Gestational age of study population was not sufficiently stated.
Chapchap, M. J.	2001	Universal newborn hearing screening and transient evoked otoacoustic emission: new concepts in Brazil	No investigation of risk factors for hearing disorders.
Chaudhari, S.	1996	Neurologic sequelae in high risk infants--a three year follow up	No comparison of hearing impairment between preterm and full-term born infants.
Chauhan, N.	2016	Psychiatric manifestations of congenital rubella syndrome: A case report and review of literature	Case report as unsuitable study type.
Chavez, A. M. C.	2019	Association between food insecurity and perinatal risk factors with hearing problems in preterm birth	No comparison of hearing impairment between preterm and full-term born infants.
Chawla, S.	2016	Association of Neurodevelopmental Outcomes and Neonatal Morbidities of Extremely Premature Infants With Differential Exposure to Antenatal Steroids	No comparison of hearing impairment between preterm and full-term born infants.
Chayasirisobhon, S.	1996	Recording of brainstem evoked potentials and their association with gentamicin in neonates	No comparison of hearing impairment between preterm and full-term born infants.
Chebib, E.	2022	Predictors of cochleovestibular dysfunction in children with congenital cytomegalovirus infection	Gestational age of study population was not sufficiently stated.
Chen, B. Z.	2022	Effects of ambient air pollution, fresh fruit and vegetable intakes as well as maternal psychosocial stress on the outcome of newborn otoacoustic emission hearing screening	Only investigation of prematurity as risk factor for hearing impairment, other risk factors not analyzed comparing preterm and full-term born infants.
Chen, D. Y.	2021	Early Biomarkers and Hearing Impairments in Patients with Neonatal Hypoxic-Ischemic Encephalopathy	No comparison of hearing impairment between preterm and full-term born infants.
Chen, J. L.	2008	Newborn hearing screening in infants with cleft palates	No comparison of hearing impairment between preterm and full-term born infants.
Chen, K.	2018	Germinal mosaicism of PAX3 mutation caused Waardenburg syndrome type I	Gestational age of study population was not sufficiently stated.
Chen, K. S.	2013	Hearing Loss and Vestibular Dysfunction Among Children With Cancer After Receiving Aminoglycosides	Gestational age of study population was not sufficiently stated.
Chen, S.	2019	[Application of ventricular shunt for children with post-infective hydrocephalus]	No comparison of hearing impairment between preterm and full-term born infants.
Chen, Y. M.	2022	Novel compound heterozygous SUCLG1 variants may contribute to mitochondria DNA depletion syndrome-9	Case report as unsuitable study type.
Cheng, H.	2019	Missense variants in TAF1 and developmental phenotypes: challenges of determining pathogenicity	Gestational age of study population was not sufficiently stated.
Cherian, B.	2002	Sensorineural hearing loss following acute bacterial meningitis in non-neonates	Gestational age of study population was not sufficiently stated.
Cherukupally, S. R.	2004	Vaccine-preventable pediatric postmeningitic sensorineural hearing loss in southern India	Gestational age of study population was not sufficiently stated.
Chesley, P. M.	2016	Neurodevelopmental and Cognitive Outcomes in Children With Intestinal Failure	No comparison of hearing impairment between preterm and full-term born infants.
Chess, S.	1980	Neurologic damage and behavior disorder in rubella children	Gestational age of study population was not sufficiently stated.
Cheung, P. Y.	2021	Outcomes of Preterm Infants With Congenital Heart Defects After Early Surgery: Defining Risk Factors at Different Time Points During Hospitalization	No comparison of hearing impairment between preterm and full-term born infants.
Cheung, P. Y.	1999	Prolonged use of pancuronium bromide and sensorineural hearing loss in childhood survivors of congenital diaphragmatic hernia	No comparison of hearing impairment between preterm and full-term born infants.
Chiabi, A.	2004	The clinical spectrum of severe malaria in children in the east provincial hospital of Bertoua, Cameroon	Gestational age of study population was not sufficiently stated.
Chiaie, L. D.	2018	No evidence of obstetrical adverse events after hyperimmune globulin application for primary cytomegalovirus infection in pregnancy: experience from a single centre	No investigation of risk factors for hearing impairment.

Chiang, K. L.	2019	Prevalence and demographic characteristics of comorbid epilepsy in children and adolescents with cerebral palsy: a nationwide population-based study	Gestational age of study population was not sufficiently stated.
Chilcote, R. R.	1983	Neutropenia, recurrent bacterial infections, and congenital deafness in patients with monocytopenia. Absence of peripheral blood colony-stimulating activity	Gestational age of study population was not sufficiently stated.
Chimowa, T.	2017	Cryptococcal meningitis in a previously healthy child	Case report as unsuitable study type.
Chinchankar, N.	2002	Diagnosis and outcome of acute bacterial meningitis in early childhood	Gestational age of study population was not sufficiently stated.
Chinetti, V.	2010	Screening for GJB2 and GJB6 gene mutations in patients from Campania region with sensorineural hearing loss	Gestational age of study population was not sufficiently stated.
Chiong, C. M.	2003	Neonatal hearing screening in a neonatal intensive care unit using distortion-product otoacoustic emissions	Only investigation of prematurity as risk factor for hearing impairment, other risk factors not analyzed comparing preterm and full-term born infants.
Chiong, C. M.	2018	The SLC26A4 c.706C > G (p.Leu236Val) Variant is a Frequent Cause of Hearing Impairment in Filipino Cochlear Implantees	Gestational age of study population was not sufficiently stated.
Chiong, M. A.	2017	Clinical, biochemical and molecular characteristics of Filipino patients with mucopolysaccharidosis type II - Hunter syndrome	Gestational age of study population was not sufficiently stated.
Chiriboga, L. F.	2021	Outcomes of a universal neonatal hearing screening program of 9941 newborns over a one-year period in Campinas, Brazil	Gestational age of study population was not sufficiently stated.
Chiu, Y. H.	2010	Mutations in the OTOF gene in Taiwanese patients with auditory neuropathy	Gestational age of study population was not sufficiently stated.
Choeyprasert, W.	2013	Cisplatin-induced Ototoxicity in Pediatric Solid Tumors: The Role of Glutathione S-Transferases and Megalin Genetic Polymorphisms	Gestational age of study population was not sufficiently stated.
Choi, B. Y.	2016	Clinical observations and molecular variables of patients with hearing loss and incomplete partition type III	Gestational age of study population was not sufficiently stated.
Choi, H. G.	2019	Air pollution increases the risk of SSNHL: A nested case-control study using meteorological data and national sample cohort data	Gestational age of study population was not sufficiently stated.
Choi, H. G.	2020	The Relation of Sudden Sensorineural Hearing Loss in Pediatric Patients With Recurrent Otitis Media: A Nested Case-control Study Using a National Sample Cohort	Gestational age of study population was not sufficiently stated.
Choi, J. Y.	2016	The clinical outcomes of deep gray matter injury in children with cerebral palsy in relation with brain magnetic resonance imaging	No comparison of hearing impairment between preterm and full-term born infants.
Choi, K. Y.	2020	Analysis of the Risk Factors Associated with Hearing Loss of Infants Admitted to a Neonatal Intensive Care Unit: A 13-Year Experience in a University Hospital in Korea	No comparison of hearing impairment between preterm and full-term born infants.
Choi, K. Y.	2009	Detection of Cytomegalovirus DNA in Dried Blood Spots of Minnesota Infants Who Do Not Pass Newborn Hearing Screening	Gestational age of study population was not sufficiently stated.
Choi, Y.	2013	Emergence of antiviral resistance during oral valganciclovir treatment of an infant with congenital cytomegalovirus (CMV) infection	Case report as unsuitable study type.
Choi, Y. H.	2017	Environmental Exposures to Lead, Mercury, and Cadmium and Hearing Loss in Adults and Adolescents: KNHANES 2010-2012	Gestational age of study population was not sufficiently stated.
Choong, C. T.	2021	Good hearing outcome in children recovering from non-polio enteroviral meningitis	No comparison of hearing impairment between preterm and full-term born infants.
Choung, Y. H.	2008	Cochlear implantation and connexin expression in the child with keratitis-ichthyosis-deafness syndrome	Case report as unsuitable study type.
Chowdhury, M. A.	2002	Comparative study between tubotympanic and atticoantral types of chronic suppurative otitis media	Gestational age of study population was not sufficiently stated.
Christensen, L. A.	1998	Antiviral therapy in a child with pediatric human immunodeficiency virus (HIV): case study of audiologic findings	Case report as unsuitable study type.
Christie, D.	2011	Long-term outcomes of pneumococcal meningitis in childhood and adolescence	Gestational age of study population was not sufficiently stated.
Christopher, N.	2013	The prevalence of hearing impairment in the 6 months-5 years HIV/AIDS-positive patients attending paediatric infectious disease clinic at Mulago Hospital	Gestational age of study population was not sufficiently stated.
Christopherson, K. M.	2014	Late toxicity following craniospinal radiation for early-stage medulloblastoma	Gestational age of study population was not sufficiently stated.
Chu, C. W.	2015	Government-funded universal newborn hearing screening and genetic analyses of deafness predisposing genes in Taiwan	Gestational age of study population was not sufficiently stated.

Chu, K.	2003	Antecedents of newborn hearing loss	Only investigation of prematurity as risk factor for hearing impairment, other risk factors not analyzed comparing preterm and full-term born infants.
Chudley, A. E.	1997	Bilateral sensorineural deafness and hydrocephalus due to foramen of Monro obstruction in sibs: A newly described autosomal recessive disorder	Case report as unsuitable study type.
Chung, W. N.	2020	Middle Ear Effusion in Children With Congenital Cytomegalovirus Infection	Gestational age of study population was not sufficiently stated.
Church, M. W.	1987	Chronic in utero alcohol exposure affects auditory function in rats and in humans	Gestational age of study population was not sufficiently stated.
Church, M. W.	1988	Hearing disorders in children with fetal alcohol syndrome: findings from case reports	Gestational age of study population was not sufficiently stated.
Church, M. W.	1997	Hearing, language, speech, vestibular, and dentofacial disorders in fetal alcohol syndrome	Gestational age of study population was not sufficiently stated.
Church, M. W.	2009	Abnormal neurological responses in young adult offspring caused by excess omega-3 fatty acid (fish oil) consumption by the mother during pregnancy and lactation	Gestational age of study population was not sufficiently stated.
Cianfrone, F.	2018	Universal newborn hearing screening using A-TEOAE and A-ABR: The experience of¬†a¬†large public hospital	Gestational age of study population was not sufficiently stated.
Ciftdemir, N. A.	2020	Congenital and Perinatal Cytomegalovirus Infections in the Neonatal Period: Case Series	No comparison of hearing impairment between preterm and full-term born infants.
Ciftdogan, D. Y.	2011	Effect on Hearing of Oral Valganciclovir for Asymptomatic Congenital Cytomegalovirus Infection	Case report as unsuitable study type.
Cikrikci, S.	2020	Comparison of hearing screening results of Syrian refugees and Turkish newborns	Only investigation of prematurity as risk factor for hearing impairment, other risk factors not analyzed comparing preterm and full-term born infants.
Cimbalo, C.	2021	Elevated sweat chloride test: is it always cystic fibrosis?	Case presentation as unsuitable study type.
Ciorba, A.	2009	Rehabilitation and outcome of severe profound deafness in a group of 16 infants affected by congenital cytomegalovirus infection	Gestational age of study population was not sufficiently stated.
Clamp, P. J.	2020	Factors associated with the development of paediatric chronic otitis media by age nine: a prospective longitudinal cohort study of 6560 children	Gestational age of study population was not sufficiently stated.
Clark, J. L.	2008	Hearing loss in Mozambique: current data from Inhambane Province	Gestational age of study population was not sufficiently stated.
Claros, P.	2013	Active middle ear implants: Vibroplasty (TM) in children and adolescents with acquired or congenital middle ear disorders	Gestational age of study population was not sufficiently stated.
Clemens, E.	2016	Determinants of ototoxicity in 451 platinum-treated Dutch survivors of childhood cancer: A DCOG late-effects study	Gestational age of study population was not sufficiently stated.
Clemens, E.	2019	Genetic Determinants of Ototoxicity During and After Childhood Cancer Treatment: Protocol for the PanCareLIFE Study	Study protocol as unsuitable study type.
Coenraad, S.	2011	Risk factors for auditory neuropathy spectrum disorder in NICU infants compared to normal-hearing NICU controls	No comparison of hearing impairment between preterm and full-term born infants.
Cohen-Barak, E.	2022	Parental mosaic cutaneous-gonadal GJB2 mutation: From epidermal nevus to inherited ichthyosis-deafness syndrome	Case report as unsuitable study type.
Cohen-Cutler, S.	2021	Hearing Loss Risk in Pediatric Patients Treated with Cranial Irradiation and Cisplatin-Based Chemotherapy	Gestational age of study population was not sufficiently stated.
Cole, P.	2009	Facial clefting and oroauditory pathway manifestations in ankyloblepharon-ectodermal defects-cleft lip/palate (AEC) syndrome	Gestational age of study population was not sufficiently stated.
Colella-Santos, M. F.	2011	Audiological and genetics studies in high-risk infants	Only investigation of prematurity as risk factor for hearing impairment, other risk factors not analyzed comparing preterm and full-term born infants.
Colonna, A. T.	2020	Long-Term Clinical, Audiological, Visual, Neurocognitive and Behavioral Outcome in Children With Symptomatic and Asymptomatic Congenital Cytomegalovirus Infection Treated With Valganciclovir	No comparison of hearing impairment between preterm and full-term born infants.
Combs, J. T.	1996	Acoustic reflectometry: spectral analysis and the conductive hearing loss of otitis media	Gestational age of study population was not sufficiently stated.
Commandeur, A. E.	2010	Simulated effect of pneumococcal vaccination in the Netherlands on existing rules constructed in a non-vaccinated cohort predicting sequelae after bacterial meningitis	Gestational age of study population was not sufficiently stated.

Conboy, T. J.	1987	Early clinical manifestations and intellectual outcome in children with symptomatic congenital cytomegalovirus infection	No comparison of hearing impairment between preterm and full-term born infants.
Cone-Wesson, B.	2005	Prenatal alcohol and cocaine exposure: Influences on cognition, speech, language, and hearing	Review, sources screened for suitable literature for review question.
Cone-Wesson, B.	1987	Electrophysiologic assessment of auditory pathways in high risk infants	No comparison of hearing impairment between preterm and full-term born infants.
Cone-Wesson, B.	2000	Identification of neonatal hearing impairment: Infants with hearing loss	Only investigation of prematurity as risk factor for hearing impairment, other risk factors not analyzed comparing preterm and full-term born infants.
Cone, B. K.	2010	Slight-Mild Sensorineural Hearing Loss in Children: Audiometric, Clinical, and Risk Factor Profiles	Gestational age of study population was not sufficiently stated.
Connolly, J. L.	2005	Universal newborn hearing screening: Are we achieving the Joint Committee on Infant Hearing (JCIH) objectives?	Gestational age of study population was not sufficiently stated.
Conrad, A. L.	2019	Are predictors of reading impairment in isolated cleft similar to those in idiopathic dyslexia?	Gestational age of study population was not sufficiently stated.
Constantinescu, R. M.	2009	Otoacoustic emissions analysers for monitoring aminoglycosides ototoxicity	Gestational age of study population was not sufficiently stated.
Cooper, A. C.	2011	Otoacoustic Emission Screen Results in Critically Ill Neonates Who Received Gentamicin in the First Week of Life	No comparison of hearing impairment between preterm and full-term born infants.
Cortese, M.	2021	Term Birth Weight and Neurodevelopmental Outcomes	Hearing outcome not examined separately.
Cortina, G.	2009	Successful implantation of a cochlear implant in a four-yr-old boy after kidney transplantation: A case report	Case report as unsuitable study type.
Cortisse, N.	2020	[Twin pregnancy and polymalformative syndrome by Enterovirus]	Case report as unsuitable study type.
Coscia, A.	2020	Risk of Symptomatic Infection after Non-Primary Congenital Cytomegalovirus Infection	No comparison of hearing impairment between preterm and full-term born infants.
Coticchia, J. M.	2006	Characteristics of sensorineural hearing loss in children with inner ear anomalies	Gestational age of study population was not sufficiently stated.
Cotter, C. S.	1994	Immune-mediated inner ear disease and parvovirus B19	Gestational age of study population was not sufficiently stated.
Counter, S. A.	2012	Acoustic stapedius muscle reflex in mercury-exposed Andean children and adults	Gestational age of study population was not sufficiently stated.
Courtmans, I.	2015	Incidence of congenital CMV in children at a hearing rehabilitation center	Gestational age of study population was not sufficiently stated.
Coutinho, C. M.	2021	Early maternal Zika infection predicts severe neonatal neurological damage: results from the prospective Natural History of Zika Virus Infection in Gestation cohort study	Gestational age of study population was not sufficiently stated.
Couto, M. I.	1999	[Audiological assessment and follow-up after bacterial meningitis]	No comparison of hearing impairment between preterm and full-term born infants.
Cox, E.	2009	Acute deafness as the presenting symptom of bacterial meningitis	Case report as unsuitable study type.
Cremers, C. W.	1979	Autosomal recessive non-syndromal progressive sensorineural deafness in childhood. A separate clinical and genetic entity	Case report as unsuitable study type.
Cross, C. P.	2015	Effect of sepsis and systemic inflammatory response syndrome on neonatal hearing screening outcomes following gentamicin exposure	No comparison of hearing impairment between preterm and full-term born infants.
Cubillana-Herrero, J. D.	2016	The assessment of the Newborn Hearing Screening Program in the Region of Murcia from 2004 to 2012	Gestational age of study population was not sufficiently stated.
Cushing, S. L.	2019	Etiology and therapy indication for cochlear implantation in children with single-sided deafness Retrospective analysis	Gestational age of study population was not sufficiently stated.
Cushing, S. L.	2008	Successful cochlear implantation in a child with Keratosis, Icthiosis and Deafness (KID) Syndrome and Dandy-Walker malformation	Case report as unsuitable study type.
Cushing, S. L.	2022	Hearing Instability in Children with Congenital Cytomegalovirus: Evidence and Neural Consequences	Gestational age of study population was not sufficiently stated.
Cuvertino, S.	2020	A restricted spectrum of missense KMT2D variants cause a multiple malformations disorder distinct from Kabuki syndrome	Gestational age of study population was not sufficiently stated.
Czech-Kowalska, J.	2021	The Limitations of Cytomegalovirus DNA Detection in Cerebrospinal Fluid of Newborn Infants With Congenital CMV Infection: A Tertiary Care Neonatal Center Experience	Connection of risk factors and/or hearing outcome depending on gestational age was not established.

Czechowicz, J. A.	2010	Hearing impairment and poverty: The epidemiology of ear disease in Peruvian schoolchildren	No comparison of hearing impairment between preterm and full-term born infants.
D'Angio, C. T.	1995	Long-term outcome of Haemophilus influenzae meningitis in Navajo Indian children	Gestational age of study population was not sufficiently stated.
D'Mello, J.	1995	High risk register--an economical tool for early identification of hearing loss	Only investigation of prematurity as risk factor for hearing impairment, other risk factors not analyzed comparing preterm and full-term born infants.
Da Costa, V.	2012	Improvements in sensorineural hearing loss after cord blood transplant in patients with mucopolysaccharidosis	Gestational age of study population was not sufficiently stated.
da Silva, D. P. C.	2015	The importance of retesting the hearing screening as an indicator of the real early hearing disorder	Only investigation of prematurity as risk factor for hearing impairment, other risk factors not analyzed comparing preterm and full-term born infants.
da Silva, D. P. C., Ribeiro, G. E., Castilho, G. L. and Mantovani, J. C.	2018	Outcomes of Automated Auditory Evoked Potential Performed in Different Settings and the Factors Associated with Referred Cases	No comparison of hearing impairment between preterm and full-term born infants.
da Silva, L. P.	2006	Etiology of hearing impairment in children and adolescents of a reference center APADA in the city of Salvador, state of Bahia	Only investigation of prematurity as risk factor for hearing impairment, other risk factors not analyzed comparing preterm and full-term born infants.
da Silva, L. P. A.	2007	Prevalence of newborn bacterial meningitis and sepsis during the pregnancy period for public health care system participants in Salvador, Bahia, Brazil	No comparison of hearing impairment between preterm and full-term born infants.
da Silva, P. C. V.	2011	Waardenburg syndrome type I: case report	Case report as unsuitable study type.
Dabekaussen, K.	2022	Association of Outpatient Oral Macrolide Use With Sensorineural Hearing Loss in Children, Adolescents, and Young Adults	Gestational age of study population was not sufficiently stated.
Daghistani, K. J.	2002	Hearing impairment in low birth weight children	Gestational age of study population was not sufficiently stated.
Dahl, H. H. M.	2013	Etiology and Audiological Outcomes at 3 Years for 364 Children in Australia	Gestational age of study population was not sufficiently stated.
Dahle, A. J.	1988	Audiological findings in children with neonatal herpes	Gestational age of study population was not sufficiently stated.
Dahle, A. J.	1974	Subclinical congenital cytomegalovirus infection and hearing impairment	Gestational age of study population was not sufficiently stated.
Dahle, A. J.	1979	Progressive hearing impairment in children with congenital cytomegalovirus infection	Case report as unsuitable study type.
Dahnsjö, H.	1976	Tone audiometry control of children treated for meningitis with large intravenous doses of ampicillin	Gestational age of study population was not sufficiently stated.
Dai, P.	2006	Extremely low penetrance of deafness associated with the mitochondrial 12S rRNA mutation in 16 Chinese families: implication for early detection and prevention of deafness	Gestational age of study population was not sufficiently stated.
Daikhes, N. A.	2017	[The results of bilateral cochlear implantation in the children who survived meningitis]	No comparison of hearing impairment between preterm and full-term born infants.
Dakovic, I.	2014	Clinical features of cerebral palsy in children with symptomatic congenital cytomegalovirus infection	Connection of risk factors and/or hearing outcome depending on gestational age was not established.
Dall'Igna, P.	2018	Hepatoblastoma in children aged less than six months at diagnosis: A report from the SIOPEL group	Gestational age of study population was not sufficiently stated.
Damen, G. W.	2006	Quality of life and cochlear implantation in Usher syndrome type I	Gestational age of study population was not sufficiently stated.
Dammeyer, J.	2010	Prevalence and aetiology of congenitally deafblind people in Denmark	Gestational age of study population was not sufficiently stated.
Daneshi, A.	2015	Complications in a series of 4400 paediatric cochlear implantation	Gestational age of study population was not sufficiently stated.
Daniel, E.	2002	Ectodermal dysplasia: Otolaryngologic manifestations and management	Gestational age of study population was not sufficiently stated.
Danzer, E.	2019	Autism spectrum disorder and neurodevelopmental delays in children with giant omphalocele	No comparison of hearing impairment between preterm and full-term born infants.
Daoud, A. S.	1995	Bacterial meningitis: still a cause of high mortality and severe neurological morbidity in childhood	Gestational age of study population was not sufficiently stated.
Dar, L.	2017	Congenital Cytomegalovirus Infection and Permanent Hearing Loss in Rural North Indian Children	Gestational age of study population was not sufficiently stated.
Dar, L.	2008	Congenital cytomegalovirus infection in a highly seropositive semi-urban population in India	No comparison of hearing impairment between preterm and full-term born infants.

Darin, N.	1997	Changes in prevalence, aetiology, age at detection, and associated disabilities in preschool children with hearing impairment born in G√∂teborg	No comparison of hearing impairment between preterm and full-term born infants.
Das, V. K.	1988	Aetiology of bilateral sensori-neural deafness in children	No comparison of hearing impairment between preterm and full-term born infants.
Das, V. K.	1990	Prevalence of otitis media with effusion in children with bilateral sensorineural hearing loss	Gestational age of study population was not sufficiently stated.
Das, V. K.	1991	Adverse perinatal factors in the causation of sensorineural hearing impairment in young children	Missing control collective without hearing impairment.
Das, V. K.	1996	Aetiology of bilateral sensorineural hearing impairment in children: A 10 year study	Gestational age of study population was not sufficiently stated.
Daudia, A.	2010	Long-term middle-ear ventilation with subannular tubes	Gestational age of study population was not sufficiently stated.
Dauman, R.	2009	Screening to detect permanent childhood hearing impairment in neonates transferred from the newborn nursery	Gestational age of study population was not sufficiently stated.
Davidson, S.	1989	Tuberculosis of the middle ear in an infant	Case report as unsuitable study type.
Davies, L.	2011	Developmental delay of infants and young children with and without fetal alcohol spectrum disorder in the Northern Cape Province, South Africa	Gestational age of study population was not sufficiently stated.
Davis, A.	1992	The epidemiology of childhood hearing impairment: factor relevant to planning of services	Gestational age of study population was not sufficiently stated.
Davis, C. W.	1995	Invasive pneumococcal infection in children, 1981-92: a hospital-based study	Gestational age of study population was not sufficiently stated.
Davis, N. M.	2001	Auditory function at 14 years of age of very-low-birthweight	Gestational age of study population was not sufficiently stated.
Davoudi-Dehaghani, E.	2015	Allelic heterogeneity among Iranian DFNB7/11 families: report of a new Iranian deaf family with TMC1 mutation identified by next-generation sequencing	Gestational age of study population was not sufficiently stated.
Dawson, J. A.	1990	Detection and prevalence of hearing loss in a cohort of children following serogroup B, meningococcal infection 1983-1987	Gestational age of study population was not sufficiently stated.
Daya, H.	1997	Assessment of cochlear damage after pneumococcal meningitis using otoacoustic emissions	Case report as unsuitable study type.
De Aledo Linos, A. G.	2005	[Universal newborn hearing screening in Cantabria (Spain): results of the first two years]	No comparison of hearing impairment between preterm and full-term born infants.
de Alencar, N. A.	2017	Lifestyle and oral facial disorders associated with sleep bruxism in children	Gestational age of study population was not sufficiently stated.
de Almeida, L. C.	2022	Hearing and communicative skills in the first years of life in children with congenital Zika syndrome	No comparison of hearing impairment between preterm and full-term born infants.
De Barros Boishardy, A.	2005	[Universal hearing screening: 10,835 newborns tested in maternity wards of the geographical Department of Eure, France]	Connection of risk factors and/or hearing outcome depending on gestational age was not established.
De Barros, A.	2014	Rapidly progressive bilateral postmeningitic deafness in children: Diagnosis and management	Gestational age of study population was not sufficiently stated.
de Brouwer, A. P. M.	1993	Arts Syndrome	Review, sources screened for suitable literature for review question.
De Capua, B.	2007	Universal neonatal hearing screening: The Siena (Italy) experience on 19,700 newborns	No comparison of hearing impairment between preterm and full-term born infants.
De Capua, B.	2003	Newborn hearing screening by transient evoked otoacoustic emissions: analysis of response as a function of risk factors	Connection of risk factors and/or hearing outcome depending on gestational age was not established.
De Felice, C.	2008	Recurrent otitis media with effusion in preterm infants with histologic chorioamnionitis - A 3 years follow-up study	No comparison of hearing impairment between preterm and full-term born infants.
de Hoog, M.	2003	Newborn hearing screening: Tobramycin and vancomycin are not risk factors for hearing loss	Connection of risk factors and/or hearing outcome depending on gestational age was not established.
de Hoog, M.	2002	A pilot case control follow-up study on hearing in children treated with tobramycin in the newborn period	Connection of risk factors and/or hearing outcome depending on gestational age was not established.
De Jesus, L. C.	2013	Outcomes of small for gestational age infants born at <27 weeks' gestation	No comparison of hearing impairment between preterm and full-term born infants.
de Jonge, R. C.	2013	Independent validation of an existing model enables prediction of hearing loss after childhood bacterial meningitis	Prediction model as unsuitable study type.
De Kegel, A.	2016	Early motor development of children with a congenital cytomegalovirus infection	No comparison of hearing impairment between preterm and full-term born infants.

De la Calle, M.	2022	Combined treatment with immunoglobulin and valaciclovir in pregnant women with cytomegalovirus infection and high risk of symptomatic fetal disease	Gestational age of study population was not sufficiently stated.
De Luca, L. M.	2022	Audiological Risk Factors, Referral Rates and Dropouts: 9 Years of Universal Newborn Hearing Screening in North Sardinia	Only investigation of prematurity as risk factor for hearing impairment, other risk factors not analyzed comparing preterm and full-term born infants.
De Moura, C. P.	2008	Down syndrome: otolaryngological effects of rapid maxillary expansion	Gestational age of study population was not sufficiently stated.
de Paula-Vernetta, C.	2016	Malformation of the eighth cranial nerve in children	Connection of risk factors and/or hearing outcome depending on gestational age was not established.
De Santis, M.	2020	Valacyclovir in primary maternal CMV infection for prevention of vertical transmission: A case-series	No comparison of hearing impairment between preterm and full-term born infants.
De Schrijver, L.	2019	Prevalence and etiology of sensorineural hearing loss in children with down syndrome: A cross-sectional study	Gestational age of study population was not sufficiently stated.
de Vries, J. J. C.	2013	Cytomegalovirus DNA detection in dried blood spots and perilymphatic fluids from pediatric and adult cochlear implant recipients with prelingual deafness	Gestational age of study population was not sufficiently stated.
De Vries, L. S.	2004	The spectrum of cranial ultrasound and magnetic resonance imaging abnormalities in congenital cytomegalovirus infection	No comparison of hearing impairment between preterm and full-term born infants.
de Vries, L. S.	1985	Relationship of serum bilirubin levels to ototoxicity and deafness in high-risk low-birth-weight infants	No comparison of hearing impairment between preterm and full-term born infants.
De Vries, L. S.	1987	Relationship of serum bilirubin levels and hearing impairment in newborn infants	No comparison of hearing impairment between preterm and full-term born infants.
Dean, J. B.	2008	Hearing loss in pediatric oncology patients receiving carboplatin-containing regimens	Gestational age of study population was not sufficiently stated.
Deben, K.	2003	Epidemiology of hearing impairment at three Flemish institutes for deaf and speech defective children	Gestational age of study population was not sufficiently stated.
Declau, F.	2008	Etiologic and audiologic evaluations after universal neonatal hearing screening: Analysis of 170 referred neonates	Gestational age of study population was not sufficiently stated.
Dedhia, K.	2013	Children With Sensorineural Hearing Loss After Passing the Newborn Hearing Screen	Gestational age of study population was not sufficiently stated.
Dedhia, R. D.	2020	Predicting complications of pediatric temporal bone fractures	Gestational age of study population was not sufficiently stated.
Degeest, S.	2021	Leisure Noise Exposure and Associated Health-Risk Behavior in Adolescents: An Explanatory Study among Two Different Educational Programs in Flanders	Gestational age of study population was not sufficiently stated.
del Rosal, T.	2012	Treatment of symptomatic congenital cytomegalovirus infection beyond the neonatal period	Connection of risk factors and/or hearing outcome depending on gestational age was not established.
Della Volpe, A.	2019	The effects of oral supplements with Sambucus nigra, Zinc, Tyndallized Lactobacillus acidophilus (H122), Arabinogalactans, vitamin D, vitamin E and vitamin C in otitis media with effusion in children: a randomized controlled trial	Gestational age of study population was not sufficiently stated.
Demain, L. A. M.	2020	A recurrent missense variant in HARS2 results in variable sensorineural hearing loss in three unrelated families	Gestational age of study population was not sufficiently stated.
Demircan, T.	2018	Evaluation of symptomatic cytomegalovirus infections in our hospital between 2004 and 2009	No comparison of hearing impairment between preterm and full-term born infants.
Demmler-Harrison, G. J.	2020	Maternal cytomegalovirus immune status and hearing loss outcomes in congenital cytomegalovirus-infected offspring	Gestational age of study population was not sufficiently stated.
Deniz, H., Yazici, A.	2020	Neonates Hearing Screening Results: A Comparison of Chirp and Click Stimuli with an Automated Auditory Brainstem Response Device	Gestational age of study population does not meet defined inclusion criteria for prematurity (<37 weeks) and/or full-term birth (≥37 weeks) with cut-off stated at 37 weeks.
Denne, C.	2007	Intrathecal synthesis of anti-viral antibodies in pediatric patients	Gestational age of study population was not sufficiently stated.
Dennett, K. V.	2014	Sensorineural hearing loss in congenital diaphragmatic hernia survivors is associated with postnatal management and not defect size	No comparison of hearing impairment between preterm and full-term born infants.
Dereköy, F. S.	2000	Etiology of deafness in Afyon school for the deaf in Turkey	Only investigation of prematurity as risk factor for hearing impairment, other risk factors not analyzed comparing preterm and full-term born infants.
Desai, S.	1997	Sensitivity and specificity of the neonatal brain-stem auditory evoked potential for hearing and language deficits in survivors of extracorporeal membrane oxygenation	Gestational age of study population was not sufficiently stated.
Desmond, M. M.	1978	The longitudinal course of congenital rubella encephalitis in nonretarded children	No comparison of hearing impairment between preterm and full-term born infants.

Desmond, M. M.	1985	The health and educational status of adolescents with congenital rubella syndrome	Gestational age of study population was not sufficiently stated.
Deutman, A. F.	1978	Rubella retinopathy and subretinal neovascularization	Case report as unsuitable study type.
Deutsch, E. S.	1998	Sensorineural hearing loss in children after liver transplantation	Gestational age of study population was not sufficiently stated.
Devdariani, T.	2011	Association between the cytomegalovirus seroprevalence and hearing loss in early childhood	Gestational age of study population was not sufficiently stated.
Dhondt, C.	2022	Predicting Early Vestibular and Motor Function in Congenital Cytomegalovirus Infection	Connection of risk factors and/or hearing outcome depending on gestational age was not established.
Dhondt, C.	2019	Episodic Vestibular Symptoms in Children With a Congenital Cytomegalovirus Infection: A Case Series	Case series as unsuitable study type.
Di Berardino, F.	2017	Delayed cochlear implantation in post-meningitic deafness and hereditary complement C2 deficiency	Case report as unsuitable study type.
Di Cicco, M.	2001	Otorhinolaringologic manifestation of Smith-Magenis syndrome	Case report as unsuitable study type.
Di Nardo, W.	2017	Herpes simplex virus-1 and cytomegalovirus DNAs detection in the inner ear of implanted patients with non-congenital infection	Gestational age of study population was not sufficiently stated.
Di Nardo, W.	2009	Multiple Viral Genome Search in Endolabyrinthic Fluids of Profoundly Deaf Patients: Possible Cytomegalovirus Intracochlear Reactivation	Gestational age of study population was not sufficiently stated.
Di Nardo, W.	2011	Cytomegalovirus DNA Retrieval in the Inner Ear Fluids of a Congenitally Deaf Child One Month After Primary Infection: A Case Report	Case report as unsuitable study type.
Dickinson, L. J.	2018	'Asymptomatic' South Auckland preschool children have significant hearing loss and middle ear disease	Gestational age of study population was not sufficiently stated.
Diener, M. L.	2017	Outcomes From a Hearing-Targeted Cytomegalovirus Screening Program	Gestational age of study population was not sufficiently stated.
Diepstraten, F. A.	2022	A Study on Prevalence and Determinants of Ototoxicity During Treatment of Childhood Cancer (SOUND): Protocol for a Prospective Study	Gestational age of study population was not sufficiently stated.
Dietz, A.	2009	Prevalence and etiology of congenital or early acquired hearing impairment in Eastern Finland	Gestational age of study population was not sufficiently stated.
Dimopoulou, D.	2020	Low birth weight and head circumference as potential biomarkers of sensorineural hearing loss in asymptomatic congenitally CMV-infected infants	No comparison of hearing impairment between preterm and full-term born infants.
Din, T. F.	2022	Profile of paediatric tuberculosis mastoiditis - a case series	Case report as unsuitable study type.
Ding, X.	2009	Cochlear implantation in China: review of 1,237 cases with an emphasis on complications	Gestational age of study population was not sufficiently stated.
Diom, E. S.	2013	Management of acquired cholesteatoma in children: a 15 year review in ENT service of CHNU de FANN Dakar	Gestational age of study population was not sufficiently stated.
Divya, D. V.	2017	The Serological Evidence of Cytomegalovirus Infection as a Potent Aetiological Factor for Cleft Lip/Palate, Mental Retardation and Deafness	Gestational age of study population was not sufficiently stated.
Dobbins, G. C.	2019	Association of CMV genomic mutations with symptomatic infection and hearing loss in congenital CMV infection	Gestational age of study population was not sufficiently stated.
Dobrianskyj, F. M.	2019	Correlation Between Sensorineural Hearing Loss and Chronic Otorrhea	Gestational age of study population was not sufficiently stated.
Doctor, B. A.	2001	Clinical outcomes of neonatal meningitis in very-low birth-weight infants	No comparison of hearing impairment between preterm and full-term born infants.
Dodds, A.	1997	Cochlear implantation after bacterial meningitis: the dangers of delay	Case report as unsuitable study type.
Dodge-Khatami, A.	2009	Late Morbidity During Childhood and Adolescence in Previously Premature Neonates After Patent Ductus Arteriosus Closure	Connection of risk factors and/or hearing outcome depending on gestational age was not established.
Dodge, P. R.	1984	Prospective evaluation of hearing impairment as a sequela of acute bacterial meningitis	Gestational age of study population was not sufficiently stated.
Dodson, K. M.	2007	Familial unilateral deafness and delayed endolymphatic hydrops	Case report as unsuitable study type.
Donald, P. R.	1981	Streptomycin ototoxicity in the unborn child	Gestational age of study population was not sufficiently stated.
Donald, P. R.	1991	Hearing loss in the child following streptomycin administration during pregnancy	Gestational age of study population was not sufficiently stated.

Dong, M.	2021	Pharmacokinetic modelling to predict risk of ototoxicity with intravenous tobramycin treatment in cystic fibrosis	Gestational age of study population was not sufficiently stated.
Donti, T. R.	2016	Expanding the phenotypic spectrum of Succinyl-CoA ligase deficiency through functional validation of a new SUCLG1 variant	Case report as unsuitable study type.
Doo, J. G.	2019	Expression of C-type lectin receptor mRNA in otitis media with effusion and chronic otitis media with and without cholesteatoma	Gestational age of study population was not sufficiently stated.
Dorfman, L.	2020	Treatment of congenital cytomegalovirus beyond the neonatal period: an observational study	No comparison of hearing impairment between preterm and full-term born infants.
Dornelles, A. D.	2014	Enzyme replacement therapy for Mucopolysaccharidosis Type I among patients followed within the MPS Brazil Network	Gestational age of study population was not sufficiently stated.
Douglas, S. A.	2008	Meningitis resulting in hearing loss and labyrinthitis ossificans - does the causative organism matter?	Gestational age of study population was not sufficiently stated.
Dowley, A. C.	2009	Auditory neuropathy: unexpectedly common in a screened newborn population	Only investigation of full-term birth as risk factor for hearing impairment, other risk factors not analyzed comparing preterm and full-term born infants.
Drake, R.	2000	Hearing in children after meningococcal meningitis	Gestational age of study population was not sufficiently stated.
Drazin, D.	2010	Successful Surgical Drainage and Aggressive Medical Therapy in a Preterm Neonate with Bacillus cereus Meningitis	Case report as unsuitable study type.
Dreher, A. M.	2014	Spectrum of Disease and Outcome in Children with Symptomatic Congenital Cytomegalovirus Infection	No comparison of hearing impairment between preterm and full-term born infants.
Drews, C. D.	1994	Hearing impairment among 10-year-old children: metropolitan Atlanta, 1985 through 1987	Gestational age of study population was not sufficiently stated.
Driscoll, C.	2015	The validity of family history as a risk factor in pediatric hearing loss	Gestational age of study population does not meet defined inclusion criteria for prematurity (<37 weeks) and/or full-term birth (≥37 weeks) with cut-off stated at 37 weeks.
Du, J.	2016	Associations Between TGFA/TGFB3/MSX1 Gene Polymorphisms and Congenital Non-Syndromic Hearing Impairment in a Chinese Population	Gestational age of study population was not sufficiently stated.
Du, Y.	2016	Analysis of p.V37I compound heterozygous mutations in the GJB2 gene in Chinese infants and young children	Gestational age of study population was not sufficiently stated.
Dualibi, A. P.	2016	The impact of laronidase treatment in otolaryngological manifestations of patients with mucopolysaccharidosis	Gestational age of study population was not sufficiently stated.
Duan, M.	2022	Postnatal hearing loss: a study of children who passed neonatal TEOAE hearing screening bilaterally	Gestational age of study population was not sufficiently stated.
Duara, S.	1986	Neonatal screening with auditory brainstem responses: results of follow-up audiometry and risk factor evaluation	Only investigation of gestational age as risk factor for hearing impairment, other risk factors not analyzed comparing preterm and full-term born infants.
Duclaux, R.	1993	Brainstem auditory evoked potentials following meningitis in children	Gestational age of study population was not sufficiently stated.
Duman, K.	2008	Incidence of auditory neuropathy among the deaf school students	Gestational age of study population was not sufficiently stated.
Dumanch, K. A.	2017	High Risk Factors Associated With Early Childhood Hearing Loss: A 3-Year Review	Gestational age of study population was not sufficiently stated.
Dunmade, A. D.	2007	Profound bilateral sensorineural hearing loss in nigerian children: any shift in etiology?	Gestational age of study population was not sufficiently stated.
Durante, A. S.	2013	Tobacco Smoke Exposure during Childhood: Effect on Cochlear Physiology	Gestational age of study population was not sufficiently stated.
Durgut, O.	2019	The effect of adenoid hypertrophy on hearing thresholds in children with otitis media with effusion	No comparison of hearing impairment between preterm and full-term born infants.
Durisin, M.	2015	Cochlear implantation in children with bacterial meningitic deafness: The influence of the degree of ossification and obliteration on impedance and charge of the implant	Gestational age of study population was not sufficiently stated.
Dursun, F.	2016	A Novel Missense Mutation in the CLPP Gene Causing Perrault Syndrome Type 3 in a Turkish Family	Gestational age of study population was not sufficiently stated.
Dutra, M. D.	2012	Hearing thresholds in children exposed to mercury in the prenatal period	No comparison of hearing impairment between preterm and full-term born infants.
Dutton, D. B.	1985	Socioeconomic status and children's health	Gestational age of study population was not sufficiently stated.
Dyce, O.	2002	Otolaryngologic manifestations of the 22q11.2 deletion syndrome	Gestational age of study population was not sufficiently stated.

Eavey, R. D.	1995	Failure to clinically predict NICU hearing loss	No comparison of hearing impairment between preterm and full-term born infants.
Ebenezer, J.	2010	Preoperative predictors of incudal necrosis in chronic suppurative otitis media	Gestational age of study population was not sufficiently stated.
Eberhard, B. A.	1994	Perhaps vigintiphobia should only apply to infants with Rhesus erythroblastosis	Connection of risk factors and/or hearing outcome depending on gestational age was not established.
Echeverria, P.	1978	Ototoxicity of gentamicin: clinical experience in a children's hospital	Gestational age of study population was not sufficiently stated.
Eckel, H. E.	1998	[Etiology of moderate and profound deafness in childhood]	No comparison of hearing impairment between preterm and full-term born infants.
Eckhardt, S. M.	1998	New form of autosomal-recessive axonal hereditary sensory motor neuropathy	Case report as unsuitable study type.
Edmond, K.	2010	Prospective cohort study of disabling sequelae and quality of life in children with bacterial meningitis in urban Senegal	Gestational age of study population was not sufficiently stated.
Edmonds, J. L.	2002	The otolaryngological manifestations of mitochondrial disease and the risk of neurodegeneration with infection	Gestational age of study population was not sufficiently stated.
Edwards, M. S.	1981	Complications and sequelae of meningococcal infections in children	Gestational age of study population was not sufficiently stated.
Egge, K.	1981	Severe hearing loss and retinopathy in children: on the possible association with rubella virus infection	Gestational age of study population was not sufficiently stated.
Eisenberg, L. S.	1984	Electrical stimulation of the auditory system in children deafened by meningitis	Gestational age of study population was not sufficiently stated.
Eisenberger, T.	2014	Targeted and genomewide NGS data disqualify mutations in MYO1A, the "DFNA48 gene", as a cause of deafness	Gestational age of study population was not sufficiently stated.
Eisenhut, M.	2003	Cerebrospinal fluid glucose levels and sensorineural hearing loss in bacterial meningitis	Gestational age of study population was not sufficiently stated.
Ekmen, S.	2021	Evaluation of Gentamicin Ototoxicity in Newborn Infants: A Retrospective Observational Study	Gestational age of study population was not sufficiently stated.
El Houchi, S. Z.	2017	Prediction of 3- to 5-Month Outcomes from Signs of Acute Bilirubin Toxicity in Newborn Infants	No comparison of hearing impairment between preterm and full-term born infants.
El Mashad, G. M.	2017	Biochemical alteration in children with idiopathic nephrotic syndrome associated with an increased risk of sensorineural hearing loss; additional insights in cochlear renal relationship	Gestational age of study population was not sufficiently stated.
El Shafei, R. R.	2021	The effects of diabetes mellitus type 1 on children's audiovestibular system: a randomized case control study	Gestational age of study population was not sufficiently stated.
El-Badry, M. M.	2014	Epileptiform electroencephalogram abnormality in children with congenital sensorineural hearing loss	No comparison of hearing impairment between preterm and full-term born infants.
El-barbary, M. N.	2015	Gentamicin extended interval regimen and ototoxicity in neonates	Connection of risk factors and/or hearing outcome depending on gestational age was not established.
El-Kersh, K.	2015	Severe Central Sleep Apnea in Vici Syndrome	Case report as unsuitable study type.
Elahi, M. M.	1998	Paediatric hearing loss in rural Pakistan	Gestational age of study population was not sufficiently stated.
ElAlfy, M. S.	2020	Auditory brainstem response in full-term neonates born to mothers with iron deficiency anemia: relation to disease severity	No comparison of hearing impairment between preterm and full-term born infants.
Elango, S.	1993	Aetiology of deafness in children from a school for the deaf in Malaysia	Only investigation of prematurity as risk factor for hearing impairment, other risk factors not analyzed comparing preterm and full-term born infants.
Elemraid, M. A.	2010	Characteristics of hearing impairment in Yemeni children with chronic suppurative otitis media: a case-control study	Gestational age of study population was not sufficiently stated.
Elkabariti, R. H.	2014	Speech evoked auditory brainstem response findings in children with epilepsy	Gestational age of study population was not sufficiently stated.
Ellis, M. I.	1980	Follow-up study of survivors after intra-uterine transfusion	No comparison of hearing impairment between preterm and full-term born infants.
Ellsworth, J.	1979	Meningococcal meningitis in children	Gestational age of study population was not sufficiently stated.
Elsayed, S. M.	2015	Non-manifesting AHI1 truncations indicate localized loss-of-function tolerance in a severe Mendelian disease gene	Gestational age of study population was not sufficiently stated.
Elshahoubi, A.	2019	Feasibility of high-dose chemotherapy protocols to treat infants with malignant central nervous system tumors: Experience from a middle-income country	Gestational age of study population was not sufficiently stated.

Emmett, S. D.	2014	Bilateral Hearing Loss is Associated With Decreased Nonverbal Intelligence in US Children Aged 6 to 16 Years	Gestational age of study population was not sufficiently stated.
Emmett, S. D.	2018	Early childhood undernutrition increases risk of hearing loss in young adulthood in rural Nepal	Gestational age of study population was not sufficiently stated.
Enders, G.	1998	Bilateral deafness due to congenital CMV and not rubella reinfection	Case report as unsuitable study type.
Endo, T.	2009	Detection of Congenital Cytomegalovirus Infection Using Umbilical Cord Blood Samples in a Screening Survey	Hearing outcome not examined.
Engel, J.	1999	Risk factors of otitis media with effusion during infancy	No comparison of hearing impairment between preterm and full-term born infants.
Engel, J.	2000	Predictive value of parent-reported symptoms in the assessment of otitis media with effusion during infancy	Gestational age of study population was not sufficiently stated.
Engel, J. A. M.	1999	Chronic otitis media with effusion during infancy, have parent-reported symptoms prognostic value? A prospective longitudinal study from 0 to 2 years of age	No comparison of hearing impairment between preterm and full-term born infants.
Engman, M. L.	2010	Congenital cytomegalovirus infection: the impact of cerebral cortical malformations	Gestational age of study population was not sufficiently stated.
Engman, M. L.	2008	Congenital CMV infection: Prevalence in newborns and the impact on hearing deficit	No comparison of hearing impairment between preterm and full-term born infants.
Eras, Z.	2013	Impact of oral versus intravenous ibuprofen on neurodevelopmental outcome: a randomized controlled parallel study	No comparison of hearing impairment between preterm and full-term born infants.
Eras, Z.	2014	Postnatal risk factors associated with hearing loss among high-risk preterm infants: tertiary center results from Turkey	No comparison of hearing impairment between preterm and full-term born infants.
Erbek, S. H.	2006	Vertigo in childhood: A clinical experience	Gestational age of study population was not sufficiently stated.
Erdogdu, S.	2021	Our newborn hearing screening results	Gestational age of study population was not sufficiently stated.
Erickson, L.	1998	Complications and sequelae of meningococcal disease in Quebec, Canada, 1990-1994	Gestational age of study population was not sufficiently stated.
Eriksen, V.	2009	Follow-up of 5-to 11-year-old children treated for persistent pulmonary hypertension of the newborn	Connection of risk factors and/or hearing outcome depending on gestational age was not established.
Ersoy, M.	2021	Evaluation of Glycogen Storage Patients: Report of Twelve Novel Variants and New Clinical Findings in a Turkish Population	Gestational age of study population was not sufficiently stated.
Ertl, T.	2001	Hyponatremia and sensorineural hearing loss in preterm infants	No comparison of hearing impairment between preterm and full-term born infants.
Esa, R.	1993	Auricular sinus	Gestational age of study population was not sufficiently stated.
Escobar-Ipuz, F. A.	2019	Early detection of neonatal hearing loss by otoacoustic emissions and auditory brainstem response over 10 years of experience	Gestational age of study population was not sufficiently stated.
Esposito, S.	2000	Ceftazidime for outpatient parenteral antibiotic therapy (OPAT) of chronic suppurative otitis media due to Pseudomonas aeruginosa	Gestational age of study population was not sufficiently stated.
Eviatar, L.	1981	Aminoglycoside ototoxicity in the neonatal period: possible etiologic factor delayed postural control	Connection of risk factors and/or hearing outcome depending on gestational age was not established.
Ewers, E. C.	2015	A Unique Case of Adolescent Neuroborreliosis Presenting With Multiple Cranial Neuritis and Cochlear Inflammation on Magnetic Resonance Imaging	Case report as unsuitable study type.
Ezzeldin, Z. M.	2021	Hearing screening in neonates with hyperbilirubinemia	No comparison of hearing impairment between preterm and full-term born infants.
Fadel, F. I.	2022	Hearing assessment in Egyptian children with chronic renal failure on regular hemodialysis and renal transplantation children	Gestational age of study population was not sufficiently stated.
Faden, H.	1982	Renal and Auditory toxic effects of amikacin in children with cancer	Gestational age of study population was not sufficiently stated.
Fageeh, N. A.	2003	Prospective study of hearing loss in schools for deaf children in Assir region, Saudi Arabia	Connection of risk factors and/or hearing outcome depending on gestational age was not established.
Faistauer, M.	2021	Etiology of early hearing loss in Brazilian children	Gestational age of study population was not sufficiently stated.
Fakhim, S. A.	2010	Study of Prevalence and Causes of Hearing Loss in High Risk Neonates Admitted to Neonatal Ward and Neonatal Intensive Care Unit	Only investigation of prematurity as risk factor for hearing impairment, other risk factors not analyzed comparing preterm and full-term born infants.
Fanaroff, A. A.	2006	Short- and long-term consequences of hypotension in ELBW infants	No comparison of hearing impairment between preterm and full-term born infants.

Fanaroff, J. M.	2006	Treated hypotension is associated with neonatal morbidity and hearing loss in extremely low birth weight infants	No comparison of hearing impairment between preterm and full-term born infants.
Fandino-Cardenas, M.	2019	Zika Virus Infection during Pregnancy and Sensorineural Hearing Loss among Children at 3 and 24 Months Post-Partum	No comparison of hearing impairment between preterm and full-term born infants.
Fang, B. X.	2020	Etiology of newborn hearing impairment in Guangdong province: 10-year experience with screening, diagnosis, and follow-up	Gestational age of study population was not sufficiently stated.
Fang, F.	1991	Effects of cytomegalovirus hepatitis on growth, development and nervous system of infants. A follow-up study	Gestational age of study population was not sufficiently stated.
Fang, T. Y.	2016	Pediatric otitis media in Fiji: Survey findings 2015	Gestational age of study population was not sufficiently stated.
Faria, A. O. P.	2020	Audiological Findings in Children Suspected to Have Been Exposed to the Zika Virus in the Intrauterine Period	Gestational age of study population was not sufficiently stated.
Farrell, A. N.	2019	Sensorineural hearing loss in children with sickle cell disease	Gestational age of study population was not sufficiently stated.
Fasunla, A. J.	2014	Comparison of auditory brainstem response in HIV-1 exposed and unexposed newborns and correlation with the maternal viral load and CD4(+) cell counts	No comparison of hearing impairment between preterm and full-term born infants.
Fasunla, A. J.	2018	Long-term effects of Maternal HIV Infection and Anti-Retroviral Medications on the Hearing of HIV-Exposed Infants	Gestational age of study population was not sufficiently stated.
Fasunla, A. J.	2013	An audit of ear, nose and throat diseases in a tertiary health institution in South-western Nigeria	Gestational age of study population was not sufficiently stated.
Faundes, V.	2021	Clinical delineation, sex differences, and genotype-phenotype correlation in pathogenic KDM6A variants causing X-linked Kabuki syndrome type 2	Connection of risk factors and/or hearing outcome depending on gestational age was not established.
Faure-Bardon, V.	2019	Sequelae of Congenital Cytomegalovirus Following Maternal Primary Infections Are Limited to Those Acquired in the First Trimester of Pregnancy	Gestational age of study population was not sufficiently stated.
Faure-Bardon, V.	2020	Refining the prognosis of fetuses infected with Cytomegalovirus in the first trimester of pregnancy by serial prenatal assessment: a single-centre retrospective study	Gestational age of study population was not sufficiently stated.
Feder, K.	2013	Audiometric thresholds and portable digital audio player user listening habits	Gestational age of study population was not sufficiently stated.
Feinmesser, M.	1982	Follow-up of 40,000 infants screened for hearing defect	Gestational age of study population was not sufficiently stated.
Feldman, W. E.	1982	Relation of concentrations of Haemophilus influenzae type b in cerebrospinal fluid to late sequelae of patients with meningitis	Gestational age of study population was not sufficiently stated.
Fellick, J. M.	2001	Neurodevelopmental outcome in meningococcal disease: a case-control study	Gestational age of study population was not sufficiently stated.
Fellinger, J.	2009	Correlates of mental health disorders among children with hearing impairments	Gestational age of study population was not sufficiently stated.
Feniman, M. R.	2008	Verbal recognition of infants with cleft lip and palate with and without history of risk indicators for hearing loss	Gestational age of study population was not sufficiently stated.
FerberViart, C.	1996	Type of initial brainstem auditory evoked potentials (BAEP) impairment and risk factors in premature infants	No comparison of hearing impairment between preterm and full-term born infants.
Ferreira, L.	2020	Effect of congenital toxoplasmosis on the encoding of speech in infants	Gestational age of study population was not sufficiently stated.
Ficenec, S. C.	2020	Lassa Fever Induced Hearing Loss: The Neglected Disability of Hemorrhagic Fever	Gestational age of study population was not sufficiently stated.
Fierek, O.	2004	Large intraosseous hemangioma of the temporal bone in a child	Case report as unsuitable study type.
Filipe, M.	2020	Suppurative otitis media in Angola: clinical and demographic features	Gestational age of study population was not sufficiently stated.
Finch, L. E.	2021	Incidence of childhood hearing loss after in utero exposure to platinum agents	No comparison of hearing impairment between preterm and full-term born infants.
Finckh-Kramber, U.	2000	Hearing screening in high-risk neonates	Only investigation of prematurity as risk factor for hearing impairment, other risk factors not analyzed comparing preterm and full-term born infants.
Findlay, L. C.	2012	The health of Inuit children under age 6 in Canada	Gestational age of study population was not sufficiently stated.
Findlen, U. M.	2021	Hearing Status of Children and Adolescents With Familial Adenomatous Polyposis	Gestational age of study population was not sufficiently stated.

Fine, J. D.	2007	Tracheolaryngeal complications of inherited epidermolysis bullosa: cumulative experience of the national epidermolysis bullosa registry	Gestational age of study population was not sufficiently stated.
Finitzo-Hieber, T.	1979	Ototoxicity in neonates treated with gentamicin and kanamycin: results of a four-year controlled follow-up study	Gestational age of study population was not sufficiently stated.
Finitzo-Hieber, T.	1981	Abnormalities of the auditory brainstem response in post-meningitic infants and children	Gestational age of study population was not sufficiently stated.
Fior, R.	1984	Late results and complications of tympanostomy tube insertion for prophylaxis of recurrent purulent otitis media in pediatric age	Gestational age of study population was not sufficiently stated.
Fiot, E.	2019	X chromosome gene dosage as a determinant of congenital malformations and of age-related comorbidity risk in patients with Turner syndrome, from childhood to early adulthood	Gestational age of study population was not sufficiently stated.
Firasat, S.	2021	SLC4A11 mutations causative of congenital hereditary endothelial dystrophy (CHED) progressing to Harboyan syndrome in consanguineous Pakistani families	Gestational age of study population was not sufficiently stated.
Firat, Y.	2008	Isolated preauricular pits and tags: is it necessary to investigate renal abnormalities and hearing impairment?	Gestational age of study population was not sufficiently stated.
Fiscaletti, M.	2018	Novel variant in Sp7/Osx associated with recessive osteogenesis imperfecta with bone fragility and hearing impairment	Case report as unsuitable study type.
Fisch, L.	1969	Causes of congenital deafness	Gestational age of study population was not sufficiently stated.
Fischer, T. C.	2009	Genetic evaluation of American minority pediatric cochlear implant recipients	Gestational age of study population was not sufficiently stated.
Fischler, R. S.	1985	Otitis media and language performance in a cohort of Apache Indian children	Gestational age of study population was not sufficiently stated.
Fisgin, T.	2009	Immune thrombocytopenic purpura-related hemotympanum presenting with hearing loss	Case report as unsuitable study type.
Fitzgerald, M. P.	2019	Hearing impairment and hypoxia ischaemic encephalopathy: Incidence and associated factors	Gestational age of study population was not sufficiently stated.
Fitzgibbons, E. J.	2021	Predicting hearing loss from 10 years of universal newborn hearing screening results and risk factors	Gestational age of study population was not sufficiently stated.
Fitzgibbons, E. J.	2022	Childhood hearing loss detected beyond the newborn screen	Gestational age of study population was not sufficiently stated.
Fitzhardinge, P. M.	1974	Long-term sequelae of neonatal meningitis	Gestational age of study population was not sufficiently stated.
Fitzpatrick, E. M.	2020	Progressive Hearing Loss in Children With Mild Bilateral Hearing Loss	Gestational age of study population was not sufficiently stated.
Fleischer, G.	1999	[Documentation of the effects of child cap pistols]	Gestational age of study population was not sufficiently stated.
Fligor, B. J.	2005	Factors associated with sensorineural hearing loss among survivors of extracorporeal membrane oxygenation therapy	Gestational age of study population was not sufficiently stated.
Flint, E. F.	1983	Severe childhood deafness in Glasgow, 1965-1979	Missing control collective without hearing impairment.
Floret, D.	1980	Hyperthyroidism, diabetes mellitus and the congenital rubella syndrome	Case report as unsuitable study type.
Flynn, M.	2004	Universal Newborn Hearing Screening introduced to NICU infants in Canterbury Province, New Zealand	Gestational age of study population was not sufficiently stated.
Foch, C.	2018	In utero drug exposure and hearing impairment in 2-year-old children A case-control study using the EFEMERIS database	Only investigation of prematurity as risk factor for hearing impairment, other risk factors not analyzed comparing preterm and full-term born infants.
Foerst, A.	2006	Prevalence of auditory neuropathy/synaptopathy in a population of children with profound hearing loss	Gestational age of study population was not sufficiently stated.
Folsom, R. C.	2000	Identification of neonatal hearing impairment: Recruitment and follow-up	Gestational age of study population was not sufficiently stated.
Fontes, A. A.	2019	Study of brainstem auditory evoked potentials in early diagnosis of congenital toxoplasmosis	No comparison of hearing impairment between preterm and full-term born infants.
Ford, G. W.	1985	Handicaps and health problems in 2 year old children of birth weight 500 to 1500 g	No comparison of hearing impairment between preterm and full-term born infants.
Ford, L. C.	2000	Otolaryngological manifestations of velocardiofacial syndrome: a retrospective review of 35 patients	Gestational age of study population was not sufficiently stated.

Forgor, A. A.	2005	Emergence of W135 meningococcal meningitis in Ghana	Gestational age of study population was not sufficiently stated.
Forner, G.	2015	High Cytomegalovirus (CMV) DNAemia Predicts CMV Sequelae in Asymptomatic Congenitally Infected Newborns Born to Women With Primary Infection During Pregnancy	No comparison of hearing impairment between preterm and full-term born infants.
Forrest, J. M.	1970	Congenital rubella in schoolchildren and adolescents	No comparison of hearing impairment between preterm and full-term born infants.
Fortnum, H.	1997	Epidemiology of permanent childhood hearing impairment in Trent Region, 1985-1993	Gestational age of study population was not sufficiently stated.
Fortnum, H.	1993	Hearing impairment in children after bacterial meningitis: incidence and resource implications	Gestational age of study population was not sufficiently stated.
Fouladi, M.	2008	Amifostine protects against cisplatin-induced ototoxicity in children with average-risk medulloblastoma	Gestational age of study population was not sufficiently stated.
Foulds, N.	2005	Carbimazole embryopathy: An emerging phenotype	Case report as unsuitable study type.
Foulon, I.	2019	Hearing Loss With Congenital Cytomegalovirus Infection	Gestational age of study population was not sufficiently stated.
Foulon, I.	2012	Hearing thresholds in children with a congenital CMV infection: A prospective study	Gestational age of study population was not sufficiently stated.
Foulon, I.	2008	Hearing Loss in Children With Congenital Cytomegalovirus Infection in Relation to the Maternal Trimester in Which the Maternal Primary Infection Occurred	Gestational age of study population was not sufficiently stated.
Foulon, I.	2008	10-year prospective study of sensorineural hearing loss in children with congenital cytomegalovirus infection	No comparison of hearing impairment between preterm and full-term born infants.
Foulon, I.	2016	Detection of CMV DNA in the perilymph of a 6-year-old boy with congenital cytomegalovirus infection	Case report as unsuitable study type.
Foulon, I.	2015	Hearing configuration in children with cCMV infection and proposal of a flow chart for hearing evaluation	Gestational age of study population was not sufficiently stated.
Fourgeaud, J.	2022	Performance of Targeted Congenital Cytomegalovirus Screening in Newborns Failing Universal Hearing Screening: A Multicenter Study	Gestational age of study population was not sufficiently stated.
Fowler, K. B.	1999	Newborn hearing screening: Will children with hearing loss caused by congenital cytomegalovirus infection be missed?	Gestational age of study population was not sufficiently stated.
Fowler, K. B.	1997	Progressive and fluctuating sensorineural hearing loss in children with asymptomatic congenital cytomegalovirus infection	Gestational age of study population was not sufficiently stated.
Fowler, K. B.	2017	A Targeted Approach for Congenital Cytomegalovirus Screening Within Newborn Hearing Screening	No comparison of hearing impairment between preterm and full-term born infants.
Fowler, K. B.	2018	Racial and Ethnic Differences in the Prevalence of Congenital Cytomegalovirus Infection	Gestational age of study population was not sufficiently stated.
Fowler, K. B.	1992	The outcome of congenital cytomegalovirus infection in relation to maternal antibody status	Connection of risk factors and/or hearing outcome depending on gestational age was not established.
Franck, C.	2017	Prevalence, Risk Factors and Diagnostics of Hearing Impairment in Preterm Infants	No comparison of hearing impairment between preterm and full-term born infants.
Francois, M.	1997	Hearing impairment in infants after meningitis: detection by transient evoked otoacoustic emissions	Gestational age of study population was not sufficiently stated.
Francois, M.	1995	AUDIOLOGICAL ASSESSMENT OF INFANTS AND CHILDREN WITH PREAURICULAR TAGS	Gestational age of study population was not sufficiently stated.
Freeland, A.	2010	Sensorineural deafness in Tanzanian children--is ototoxicity a significant cause? A pilot study by Freeland et al. [Int. J. Pediatr. Otorhinolaryngol. 74 (2) (2010) 516-519]	Gestational age of study population was not sufficiently stated.
Freyer, D. R.	2017	Effects of sodium thiosulfate versus observation on development of cisplatin-induced hearing loss in children with cancer (ACCL0431): a multicentre, randomised, controlled, open-label, phase 3 trial	Gestational age of study population was not sufficiently stated.
Friedman, A. B.	2013	Risk analysis of unilateral severe-to-profound sensorineural hearing loss in children	Gestational age of study population was not sufficiently stated.
Fuchs, A.	2016	Gentamicin Exposure and Sensorineural Hearing Loss in Preterm Infants	No comparison of hearing impairment between preterm and full-term born infants.
Fujita, M.	1986	Risk factors related to hearing impairment and screening with the Crib-O-Gram	Gestational age of study population was not sufficiently stated.
Fukuda, S.	2001	An anti-mumps IgM antibody level in the serum of idiopathic sudden sensorineural hearing loss	Gestational age of study population was not sufficiently stated.

Fukushima, K.	2008	Developmental dysgraphia with profound hearing impairment: Intervention by auditory methods enabled by cochlear implant	Gestational age of study population was not sufficiently stated.
Fukushima, S.	2019	Prediction of poor neurological development in patients with symptomatic congenital cytomegalovirus diseases after oral valganciclovir treatment	No comparison of hearing impairment between preterm and full-term born infants.
Fullerton, B. S.	2018	Severe neurodevelopmental disability and healthcare needs among survivors of medical and surgical necrotizing enterocolitis: A prospective cohort study	No comparison of hearing impairment between preterm and full-term born infants.
Funamura, J. L.	2019	Children with Cleft Palate: Predictors of Otologic Issues in the First 10 Years	Gestational age of study population was not sufficiently stated.
Furuta, S.	2000	Reduced size of the cochlear branch of the vestibulocochlear nerve in a child with sensorineural hearing loss	Case report as unsuitable study type.
Furutate, S.	2011	Clinical profile of hearing loss in children with congenital cytomegalovirus (CMV) infection: CMV DNA diagnosis using preserved umbilical cord	Gestational age of study population was not sufficiently stated.
Gäckler, A.	2010	Positive family history of idiopathic sudden sensorineural hearing loss	Gestational age of study population was not sufficiently stated.
Gainville, A.	2021	Drug-Induced Hearing Loss in Children: An Analysis of Spontaneous Reports in the French PharmacoVigilance Database	No comparison of hearing impairment between preterm and full-term born infants.
Galambos, R.	1980	The auditory brainstem response (ABR) evaluates risk factors for hearing loss in the newborn	Only investigation of gestational age as risk factor for hearing impairment, other risk factors not analyzed comparing preterm and full-term born infants.
Gallach, A. D.	2020	Neurological sequelae in patients with congenital cytomegalovirus	No comparison of hearing impairment between preterm and full-term born infants.
Gallant, E.	2013	Homozygosity for the V37I GJB2 mutation in fifteen probands with mild to moderate sensorineural hearing impairment: further confirmation of pathogenicity and haplotype analysis in Asian populations	Gestational age of study population was not sufficiently stated.
Gallo, J.	2011	[Auditory processing evaluation in children born preterm]	Preterm infants with risk factors were compared to term infants without any risk factors, so no comparison of risk factors for hearing loss between preterm and term born infants was possible.
Gamstorp, I.	1971	School-children with perceptive deafness	Gestational age of study population was not sufficiently stated.
Gamstorp, I.	1974	Bilateral, severe, sensori-neural hearing loss after haemophilus influenzae meningitis in childhood	Gestational age of study population was not sufficiently stated.
Ganapathy, S. H.		Association of High Risk Factors and Hearing Impairment in Infants-A Hospital Based Study	Only investigation of prematurity as risk factor for hearing impairment, other risk factors not analyzed comparing preterm and full-term born infants.
Gao, M.	2020	[Common clinical causes and audiological manifestations of unilateral hearing loss in children]	No comparison of hearing impairment between preterm and full-term born infants.
Gao, X.	2022	TARS2 variants causes combination oxidative phosphorylation deficiency-21: a case report and literature review	Case report and review, sources screened for suitable literature for review question.
Garabli, H.	2010	Hearing Screening Protocols of Babies with Hearing Loss Risk Factors in Turkey	Gestational age of study population was not sufficiently stated.
Garg, S.	2018	An Epidemiological Study on Burden of Hearing Loss and Its Associated Factors in Delhi, India	Gestational age of study population was not sufficiently stated.
Gargus, R. A.	2009	Unimpaired outcomes for extremely low birth weight infants at 18 to 22 months	No comparison of hearing impairment between preterm and full-term born infants.
Garinis, A. C.	2017	Effect of gentamicin and levels of ambient sound on hearing screening outcomes in the neonatal intensive care unit: A pilot study	No comparison of hearing impairment between preterm and full-term born infants.
Garson, A.	1993	The long QT syndrome in children. An international study of 287 patients	Gestational age of study population was not sufficiently stated.
Garza Morales, S.	1997	[Auditory provoked potentials in children with neonatal risk for hypoacusia]	Only investigation of gestational age as risk factor for hearing impairment, other risk factors not analyzed comparing preterm and full-term born infants.
Gaurav, V.	2022	Effects of 'Perinatal Risk Factors Associated with Hearing Loss' on Auditory Outcomes in Cochlear Implant Recipient Children	Gestational age of study population was not sufficiently stated.
Gavrilovici, C.	2017	Message from a turtle: otitis with Salmonella arizonae in children: Case report	Case report as unsuitable study type.
Gawron, W.	2008	Evaluation of hearing organ in patients with Turner syndrome	Gestational age of study population was not sufficiently stated.
Gazeta, R. E.	2021	Three-Year Clinical Follow-Up of Children Intrauterine Exposed to Zika Virus	Connection of risk factors and/or hearing outcome depending on gestational age was not established.

Gazia, F.	2019	NICU INFANTS & SNHL: EXPERIENCE OF A WESTERN SICILY TERTIARY CARE CENTRE	Only investigation of prematurity as risk factor for hearing impairment, other risk factors not analyzed comparing preterm and full-term born infants.
Geal-Dor, M.	2013	Acquisition of early auditory milestones with a cochlear implant	Gestational age of study population was not sufficiently stated.
Gelzinis, A.	2022	Neurotrophic Keratitis Due to Congenital Corneal Anesthesia with Deafness, Hypotonia, Intellectual Disability, Face Abnormality and Metabolic Disorder: A New Syndrome?	Case report as unsuitable study type.
Genc, G. A.	2013	Features of unilateral hearing loss detected by newborn hearing screening programme in different regions of Turkey	Only investigation of prematurity as risk factor for hearing impairment, other risk factors not analyzed comparing preterm and full-term born infants.
George, I. O.	2009	Congenital rubella syndrome: pattern and presentation in a southern Nigerian tertiary hospital	Gestational age of study population was not sufficiently stated.
Gertson, K.	2020	Prevalence of Ototoxicity Following Hematopoietic Stem Cell Transplantation in Pediatric Patients	Gestational age of study population was not sufficiently stated.
Ghafari, N.	2015	The occurrence of auditory dysfunction in children with TB receiving ototoxic medication at a TB hospital in South Africa	Gestational age of study population was not sufficiently stated.
Ghaloul-Gonzalez, L.	2019	Reticular Dysgenesis and Mitochondriopathy Induced by Adenylate Kinase 2 Deficiency with Atypical Presentation	Gestational age of study population was not sufficiently stated.
Gharib, B.	2012	Recurrent bacterial meningitis in a child with hearing impairment, mondini dysplasia: a case report	Case report as unsuitable study type.
Ghirri, P.	2011	Universal neonatal audiological screening: experience of the University Hospital of Pisa	Gestational age of study population was not sufficiently stated.
Ghiselli, S.	2022	Auditory evaluation of infants born to COVID19 positive mothers	No comparison of hearing impairment between preterm and full-term born infants.
Ghosh, P. S.	2011	Lateral sinus thrombosis associated with mastoiditis and otitis media in children: a retrospective chart review and review of the literature	Gestational age of study population was not sufficiently stated.
Giannantonio, S.	2020	Genetic identification and molecular modeling characterization of a novel POU3F4 variant in two Italian deaf brothers	Case report as unsuitable study type.
Giannattasio, A.	2017	Outcomes of congenital cytomegalovirus disease following maternal primary and non-primary infection	No comparison of hearing impairment between preterm and full-term born infants.
Giannattasio, A.	2017	Is lenticulostriated vasculopathy an unfavorable prognostic finding in infants with congenital cytomegalovirus infection?	No comparison of hearing impairment between preterm and full-term born infants.
Gibson, T. M.	2018	Temporal patterns in the risk of chronic health conditions in survivors of childhood cancer diagnosed 1970-99: a report from the Childhood Cancer Survivor Study cohort	Gestational age of study population was not sufficiently stated.
Giebink, G. S.	1990	A controlled trial comparing three treatments for chronic otitis media with effusion	Gestational age of study population was not sufficiently stated.
Gierek, T.	2007	[The results of hearing screening in selected population of primary schoolchildren in Silesia]	No comparison of hearing impairment between preterm and full-term born infants.
Giles, J. P.	1965	THE RUBELLA SYNDROME	No comparison of hearing impairment between preterm and full-term born infants.
Gilliam, A.	2002	Fatal septicemia in an infant with keratitis, ichthyosis, and deafness (KID) syndrome	Case report as unsuitable study type.
Girgis, N. I.	1989	Dexamethasone treatment for bacterial meningitis in children and adults	Gestational age of study population was not sufficiently stated.
Girit, S.	2019	A Rare Diagnosis: Keutel Syndrome	Case report as unsuitable study type.
Givens, K. T.	1993	Congenital rubella syndrome: ophthalmic manifestations and associated systemic disorders	Gestational age of study population was not sufficiently stated.
Gkoltsiou, K.	2008	Serial brain MRI and ultrasound findings: relation to gestational age, bilirubin level, neonatal neurologic status and neurodevelopmental outcome in infants at risk of kernicterus	Connection of risk factors and/or hearing outcome depending on gestational age was not established.
Gkoritsa, E.	2007	Maturation of the auditory system: 2. Transient otoacoustic emission suppression as an index of the medial olivocochlear bundle maturation	Connection of risk factors and/or hearing outcome depending on gestational age was not established.
Glarner, H.	1994	MANAGEMENT OF PETROUS BONE-FRACTURES IN CHILDREN - ANALYSIS OF 127 CASES	Gestational age of study population was not sufficiently stated.
Glatz, C.	2011	Mutation in the mitochondrial tRNA(Val) causes mitochondrial encephalopathy, lactic acidosis and stroke-like episodes	Case report as unsuitable study type.

Gleich, L. L.	1994	Asymptomatic congenital syphilis and auditory brainstem response	Connection of risk factors and/or hearing outcome depending on gestational age was not established.
Glynn, F.	2011	Pierre Robin sequence: an institutional experience in the multidisciplinary management of airway, feeding and serous otitis media challenges	Gestational age of study population was not sufficiently stated.
Goderis, J.	2016	Hearing in Children with Congenital Cytomegalovirus Infection: Results of a Longitudinal Study	Only investigation of prematurity as risk factor for hearing impairment, other risk factors not analyzed comparing preterm and full-term born infants.
Goetghebuer, T.	2000	Outcome of meningitis caused by Streptococcus pneumoniae and Haemophilus influenzae type b in children in The Gambia	Gestational age of study population was not sufficiently stated.
Gohari, N.	2019	The Prevalence and Causes of Auditory Neuropathy/Dys-synchrony (AN/AD) in Children with Hearing Impairment	Gestational age of study population was not sufficiently stated.
Gokdogan, C.	2016	Management of children with auditory neuropathy spectrum disorder (ANSD)	Only investigation of prematurity as risk factor for hearing impairment, other risk factors not analyzed comparing preterm and full-term born infants.
Goldstein, N. A.	1998	Intratemporal complications of acute otitis media in infants and children	Gestational age of study population was not sufficiently stated.
Goldstein, R. F.	2013	Influence of gestational age on death and neurodevelopmental outcome in premature infants with severe intracranial hemorrhage	No comparison of hearing impairment between preterm and full-term born infants.
Gonik, L.	2021	Auditory and Language Development Assessment of Newborns Aged One to Four Years Exposed to Gestational Zika Virus Infection: A Case Series	Connection of risk factors and/or hearing outcome depending on gestational age was not established.
Gonzalez-Jimenez, B.	2017	[Neonatal Hearing Screening and Early Intervention, a screening program to evaluate all infants to identify the hearing impaired]	No comparison of hearing impairment between preterm and full-term born infants.
Göpel, W.	2014	Mitochondrial mutation m.1555A>G as a risk factor for failed newborn hearing screening in a large cohort of preterm infants	No comparison of hearing impairment between preterm and full-term born infants.
Gopineti, L.	2020	Prevalence of Sensorineural Hearing Loss in Children with Palliated or Repaired Congenital Heart Disease	Only investigation of prematurity as risk factor for hearing impairment, other risk factors not analyzed comparing preterm and full-term born infants.
Gorenstein, L.	2021	Quantitative and qualitative analysis of fetal temporal lobe T2 signal in cytomegalovirus infected fetuses and normal controls	Gestational age of study population was not sufficiently stated.
Gorga, M. P.	2000	Identification of neonatal hearing impairment: Distortion product otoacoustic emissions during the perinatal period	Connection of risk factors and/or hearing outcome depending on gestational age was not established.
Görür, K.	2005	The role of factor V Leiden and prothrombin G20210A mutations in sudden sensorineural hearing loss	Gestational age of study population was not sufficiently stated.
Goudy, S.	2006	Conductive hearing loss and otopathology in cleft palate patients	Gestational age of study population was not sufficiently stated.
Gouri, Z. U.	2015	Hearing impairment and its risk factors by newborn screening in north-western India	Only investigation of gestational age as risk factor for hearing impairment, other risk factors not analyzed comparing preterm and full-term born infants.
Gouveia, F. N.	2020	Unilateral and asymmetric hearing loss in childhood	No comparison of hearing impairment between preterm and full-term born infants.
Govender, R.	2011	Neurologic and Neurobehavioral Sequelae in Children With Human Immunodeficiency Virus (HIV-1) Infection	Gestational age of study population was not sufficiently stated.
Gowda, V. K., Kulhalli, P. and Vamyanmane, D. K.	2021	Neurological Manifestations of Congenital Cytomegalovirus Infection at a Tertiary Care Centre from Southern India	Gestational age of study population was not sufficiently stated.
Goycochea-Valdivia, W. A.	2017	Cytomegalovirus DNA Detection by Polymerase Chain Reaction in Cerebrospinal Fluid of Infants With Congenital Infection: Associations With Clinical Evaluation at Birth and Implications for Follow-up	Connection of risk factors and/or hearing outcome depending on gestational age was not established.
Grasso, F.	2018	Otolaryngological features in a cohort of patients affected with 22q11.2 deletion syndrome: A monocentric survey	Gestational age of study population was not sufficiently stated.
Grauer, J. S.	2019	Cochlear Implantation and Facial Nerve Synkinesis in an Infant with Hypoplastic Internal Auditory Canals - A Case Report	Case report as unsuitable study type.
Gravel, J. S.	2000	Effects of otitis media with effusion on hearing in the first 3 years of life	Gestational age of study population was not sufficiently stated.
Gray, P. H.	2001	Conductive hearing loss in preterm infants with bronchopulmonary dysplasia	No comparison of hearing impairment between preterm and full-term born infants.
Gray, R. F.	1989	Causes of deafness in schools for the deaf in Madras	Gestational age of study population was not sufficiently stated.
Graz, M. B.	2015	Being Small for Gestational Age: Does it Matter for the Neurodevelopment of Premature Infants? A Cohort Study	No comparison of hearing impairment between preterm and full-term born infants.

Graziani, L. J.	1997	Clinical antecedents of neurologic and audiologic abnormalities in survivors of neonatal extracorporeal membrane oxygenation	Gestational age of study population was not sufficiently stated.
Graziani, L. J.	1997	Cerebrovascular complications and neurodevelopmental sequelae of neonatal ECMO	Gestational age of study population was not sufficiently stated.
Greczka, G.	2015	Universal Neonatal Hearing Screening Program in Poland--10-year summary	Gestational age of study population was not sufficiently stated.
Greenwalt, J. C.	2016	Long-term Outcomes Following Radiotherapy for Adolescent Patients With Nonmetastatic WHO Type III Nasopharyngeal Carcinoma	Gestational age of study population was not sufficiently stated.
Greinwald, J.	2013	Significance of unilateral enlarged vestibular aqueduct	Gestational age of study population was not sufficiently stated.
Grillner, L.	1983	Outcome of rubella during pregnancy with special reference to the 17th-24th weeks of gestation	Gestational age of study population was not sufficiently stated.
Grimmer, I.	1999	Hearing in newborn infants of opiate-addicted mothers	No comparison of hearing impairment between preterm and full-term born infants.
Grimwood, K.	1995	Adverse outcomes of bacterial meningitis in school-age survivors	Gestational age of study population was not sufficiently stated.
Grimwood, K.	1996	Risk factors for adverse outcomes of bacterial meningitis	Gestational age of study population was not sufficiently stated.
Groene, S. G.	2022	Long-term effects of selective fetal growth restriction (LEMON): a cohort study of neurodevelopmental outcome in growth discordant identical twins in the Netherlands	No comparison of hearing impairment between preterm and full-term born infants.
Grögaard, J. B.	1990	Increased survival rate in very low birth weight infants (1500 grams or less): no association with increased incidence of handicaps	Gestational age of study population was not sufficiently stated.
Gross, M.	2000	Connatal hearing disorders in children. Part I: Connatally acquired hearing loss	Review, sources screened for suitable literature for review question.
Gross, M.	2001	[Congenital hearing loss in children. 2: Genetic hearing loss]	Review, sources screened for suitable literature for review question.
Gruber, R.	2017	Autosomal Recessive Keratoderma-Ichthyosis-Deafness (ARKID) Syndrome Is¬†Caused by VPS33B Mutations Affecting¬†Rab Protein Interaction and¬†Collagen Modification	Case report as unsuitable study type.
Gruss, I.	2007	Etiologies of hearing impairment among infants and toddlers: 1986-1987 versus 2001	Only investigation of prematurity as risk factor for hearing impairment, other risk factors not analyzed comparing preterm and full-term born infants.
Guerin, J. B.	2018	Labyrinthine Sequestrum: A Case Report and Review of the Literature	Case report as unsuitable study type.
Guerra-Hernandez, N.	2014	Clinical and biochemical findings in Mexican patients with distal renal tubular acidosis	Case report as unsuitable study type.
Gugel, I.	2021	Risk Stratification for Immediate Postoperative Hearing Loss by Preoperative BAER (Brainstem Auditory Evoked Response) and Audiometry in NF2-Associated Vestibular Schwannomas	Gestational age of study population was not sufficiently stated.
Guimaraes, C. D.	2019	Clinical findings in congenital infection by Zika virus: a retrospective study in a reference hospital in Central-West Brazil	No comparison of hearing impairment between preterm and full-term born infants.
Guiscafre, H.	1984	Reversible hearing loss after meningitis. Prospective assessment using auditory evoked responses	Gestational age of study population was not sufficiently stated.
Gul, H. C.	2008	Management of neurobrucellosis: An assessment of 11 cases	Case report as unsuitable study type.
Gulati, A.	2022	The Hearing Status of Preterm Infant's <= 34 Weeks as Revealed by Otoacoustic Emissions (OAE) Screening and Diagnostic Brainstem Evoked Response Audiometry (BERA): A Tertiary Center Experience	No comparison of hearing impairment between preterm and full-term born infants.
Gulleroglu, K.	2015	Hearing Status in Pediatric Renal Transplant Recipients	Gestational age of study population was not sufficiently stated.
Gulleroglu, K.	2013	Sudden Hearing Loss Associated With Tacrolimus After Pediatric Renal Transplant	Case report as unsuitable study type.
Gultekin, E.	2010	Prevalence and risk factors for persistent otitis media with effusion in primary school children in Istanbul, Turkey	Gestational age of study population was not sufficiently stated.
Gumpel, S. M.	1972	Clinical and social status of patients with congenital rubella	No comparison of hearing impairment between preterm and full-term born infants.
Gumpel, S. M.	1971	Congenital perceptive deafness: role of intrauterine rubella	Gestational age of study population was not sufficiently stated.

Gumus, E.	2018	Apolipoprotein E allelic variants and cerebral palsy	Connection of risk factors and/or hearing outcome depending on gestational age was not established.
Gunderman, L. M.	2022	Improvement of SLC29A3 spectrum disorder-related sensorineural hearing loss after initiation of IL-6 inhibitor	Case report as unsuitable study type.
Gunes, D.	2009	Platinum-Induced Ototoxicity in Children and Adolescents with Cancer	Gestational age of study population was not sufficiently stated.
Gungor, N.	2000	High frequency hearing loss in Ullrich-Turner syndrome	Gestational age of study population was not sufficiently stated.
Gunkel, J.	2018	Outcome of Preterm Infants With Postnatal Cytomegalovirus Infection	Connection of risk factors and/or hearing outcome depending on gestational age was not established.
Gunn, M. E.	2015	Late morbidity in long-term survivors of childhood brain tumors: a nationwide registry-based study in Finland	Gestational age of study population was not sufficiently stated.
Gupta, A. K.	1991	Evaluation of risk factors for hearing impairment in at risk neonates by brainstem evoked response audiometry (BERA)	Only investigation of prematurity as risk factor for hearing impairment, other risk factors not analyzed comparing preterm and full-term born infants.
Gupta, D.	1989	Toy weapons and firecrackers: a source of hearing loss	Gestational age of study population was not sufficiently stated.
Gupta, N.	2021	Relation between Mode of Delivery and Findings of Oto-Acoustic Emission Test Results	Gestational age of study population was not sufficiently stated.
Gupta, S.	2015	Challenges of Implementing Universal Newborn Hearing Screening at a Tertiary Care Centre from India	Only investigation of gestational age as risk factor for hearing impairment, other risk factors not analyzed comparing preterm and full-term born infants.
Gupta, V.	1993	Hearing evaluation in children with bacterial meningitis	Gestational age of study population was not sufficiently stated.
Ha, R.	2019	Predictive values of neutrophil to lymphocyte ratio (NLR), platelet to lymphocyte ratio (PLR), and other prognostic factors in pediatric idiopathic sudden sensorineural hearing loss	Gestational age of study population was not sufficiently stated.
Ha, S. L.	2000	Salivary gland choristoma of the middle ear: a case report	Case report as unsuitable study type.
Haapaniemi, J.	1995	The 6 kHz acoustic dip in school-aged children in Finland	Gestational age of study population was not sufficiently stated.
Haapaniemi, J. J.	1995	OTOMICROSCOPIC, AUDIOMETRIC, AND IMPEDANCE FINDINGS ASSOCIATED WITH SEPTAL DEVIATIONS IN SCHOOL-AGED CHILDREN	Gestational age of study population was not sufficiently stated.
Habib, R. G.	1979	Hearing impairment in meningococcal meningitis	Gestational age of study population was not sufficiently stated.
Hack, M.	2000	Neurodevelopment and predictors of outcomes of children with birth weights of less than 1000 g: 1992-1995	No comparison of hearing impairment between preterm and full-term born infants.
Haffey, T.	2013	Evaluation of unilateral sensorineural hearing loss in the pediatric patient	Only investigation of prematurity as risk factor for hearing impairment, other risk factors not analyzed comparing preterm and full-term born infants.
Hafström, M.	2018	Cerebral Palsy in Extremely Preterm Infants	Only investigation of gestational age as risk factor for hearing impairment, other risk factors not analyzed comparing preterm and full-term born infants.
Haggard, M. P.	2003	The role of ventilation tube status in the hearing levels in children managed for bilateral persistent otitis media with effusion	Gestational age of study population was not sufficiently stated.
Haginoya, K.	2002	Abnormal white matter lesions with sensorineural hearing loss caused by congenital cytomegalovirus infection: retrospective diagnosis by PCR using Guthrie cards	Case report as unsuitable study type.
Hajare, P.	2021	A Study of JCIH (Joint Commission on Infant Hearing) Risk Factors for Hearing Loss in Babies of NICU and Well Baby Nursery at a Tertiary Care Center	Only investigation of prematurity as risk factor for hearing impairment, other risk factors not analyzed comparing preterm and full-term born infants.
Hakli, S.	2014	Childhood hearing impairment in northern Finland, etiology and additional disabilities	Gestational age of study population was not sufficiently stated.
Hakli, S.	2013	Audiological Follow-Up of Children with the m.1555A > G Mutation in Mitochondrial DNA	Gestational age of study population was not sufficiently stated.
Häkli, S.	2014	WFS1 mutations in hearing-impaired children	Gestational age of study population was not sufficiently stated.
Hall, A. J.	2014	Glue Ear, Hearing Loss and IQ: An Association Moderated by the Child's Home Environment	Connection of risk factors and/or hearing outcome depending on gestational age was not established.
Hall, A. J.	2011	Prevalence and risk factors for mild and high-frequency bilateral sensorineural hearing loss at age 11 years old: A UK prospective cohort study	Only investigation of prematurity as risk factor for hearing impairment, other risk factors not analyzed comparing preterm and full-term born infants.

Hall, J. W.	1986	Auditory brainstem response in young burn-wound patients treated with ototoxic drugs	Gestational age of study population was not sufficiently stated.
Hall, J. W.	1987	Auditory brainstem response in auditory assessment of acute severely burned children	Gestational age of study population was not sufficiently stated.
Hall, R.	1987	Hearing loss due to mumps	Gestational age of study population was not sufficiently stated.
Halpern, J.	1987	Four factors that accurately predict hearing loss in "high risk" neonates	Only investigation of gestational age as risk factor for hearing impairment, other risk factors not analyzed comparing preterm and full-term born infants.
Hamberis, A. O.	2020	Characteristics and progression of hearing loss in children with turner's syndrome	Gestational age of study population was not sufficiently stated.
Hamid, N. A.	2022	The Prevalence of Cytomegalovirus (CMV) Infection among Infants and Correlation between CMV PCR with Clinical Outcomes in a Tertiary Teaching Hospital in Malaysia	Gestational age of study population was not sufficiently stated.
Han, J. J.	2019	Prediction of the Outcome of Cochlear Implantation in the Patients with Congenital Cytomegalovirus Infection based on Magnetic Resonance Imaging Characteristics	Gestational age of study population was not sufficiently stated.
Hanci, F.	2020	Epilepsy and drug-resistant epilepsy in children with cerebral palsy: A retrospective observational study	Connection of risk factors and/or hearing outcome depending on gestational age was not established.
Handelsman, J. A.	2017	Prevalence of hearing and vestibular loss in cystic fibrosis patients exposed to aminoglycosides	Gestational age of study population was not sufficiently stated.
Handzi*f*á-Cuk, J.	1996	Pierre Robin syndrome: characteristics of hearing loss, effect of age on hearing level and possibilities in therapy planning	Gestational age of study population was not sufficiently stated.
Hanege, B. Y.	2017	Is maternal preeclampsia risk factor for neonatal hearing loss?	Gestational age of study population does not meet defined inclusion criteria for prematurity (<37 weeks) and/or full-term birth (≥37 weeks) with cut-off stated at 37 weeks.
Hanna, J. N.	1991	Bacterial meningitis in children under five years of age in Western Australia	Gestational age of study population was not sufficiently stated.
Hanshaw, J. B.	1976	School failure and deafness after "silent" congenital cytomegalovirus infection	Gestational age of study population was not sufficiently stated.
Haque, R.	2012	Large cerebellopontine angle tuberculoma: a case report	Case report as unsuitable study type.
Hardani, A. K.	2020	Prevalence and Risk Factors for Hearing Loss in Neonates Admitted to the Neonatal Intensive Care Unit: A Hospital Study	Only investigation of prematurity as risk factor for hearing impairment, other risk factors not analyzed comparing preterm and full-term born infants.
Harden, L. M.	2022	South African Children: A Matched Cohort Study of Neurodevelopmental Impairment in Survivors of Invasive Group B Streptococcus Disease Aged 5 to 8 Years	Connection of risk factors and/or hearing outcome depending on gestational age was not established.
Harputluoglu, U.	2005	Nasopharyngeal aerobic bacterial flora and Staphylococcus aureus nasal carriage in deaf children	No comparison of hearing impairment between preterm and full-term born infants.
Harris, S.	1984	Congenital cytomegalovirus infection and sensorineural hearing loss	Case report as unsuitable study type.
Harrison, M.	1996	Age of suspicion, identification, and intervention for infants and young children with hearing loss: A national study	Only investigation of prematurity as risk factor for hearing impairment, other risk factors not analyzed comparing preterm and full-term born infants.
Hashim, S. H. A.	2020	Recurrent Otitis Media among Infants and Children in Arar City, Northern Saudi Arabia	Gestational age of study population was not sufficiently stated.
Hashimoto, H.	2009	An Office-Based Prospective Study of Deafness in Mumps	Gestational age of study population was not sufficiently stated.
Haslam, R. H.	1977	The sequelae of group B beta-hemolytic streptococcal meningitis in early infancy	Gestational age of study population was not sufficiently stated.
Hatzenbuehler, L. A.	2017	Pediatric Dental Clinic-Associated Outbreak of Mycobacterium abscessus Infection	Gestational age of study population was not sufficiently stated.
Hatzopoulos, S.	2007	Neonatal hearing screening in Albania: Results from an ongoing universal screening program	No comparison of hearing impairment between preterm and full-term born infants.
Haupt, R.	2004	Permanent consequences in Langerhans cell histiocytosis patients: A pilot study from the Histiocyte Society - Late effects study group	Gestational age of study population was not sufficiently stated.
Haverkamp, F.	2003	Familial factors and hearing impairment modulate the neuromotor phenotype in Turner syndrome	Gestational age of study population was not sufficiently stated.
Havia, M.	2005	Prevalence of Meni√®re's disease in general population of Southern Finland	Gestational age of study population was not sufficiently stated.
Hayakawa, J.	2012	A Neonate with Reduced Cytomegalovirus DNA Copy Number and Marked Improvement of Hearing in the Treatment of Congenital Cytomegalovirus Infection	Case report as unsuitable study type.

Hayes, D.	2000	Catastrophic progressive hearing loss in childhood	Case report as unsuitable study type.
Hazarika, R. D.	2013	Invasive Meningococcal Infection: Analysis of 110 cases from a Tertiary Care Centre in North East India	Gestational age of study population was not sufficiently stated.
Hazebroek, F. W.	2001	The neonate with major malformations: experiences in a university children's hospital in the Netherlands	Gestational age of study population was not sufficiently stated.
He, P.	2017	Children with motor impairment related to cerebral palsy: Prevalence, severity and concurrent impairments in China	Gestational age of study population was not sufficiently stated.
He, V. Y.	2020	The link between hearing impairment and child maltreatment among Aboriginal children in the Northern Territory of Australia: is there an opportunity for a public health approach in child protection?	No comparison of hearing impairment between preterm and full-term born infants.
Heffernan, C. B.	2018	Does Clarithromycin Cause Hearing Loss? A 12-Year Review of Clarithromycin Therapy for Nontuberculous Mycobacterial Lymphadenitis in Children	Gestational age of study population was not sufficiently stated.
Heide, S.	2022	GM3 synthase deficiency in non-Amish patients	Gestational age of study population was not sufficiently stated.
Heidemüller, B.	1994	[Ototoxicity of locally administered aminoglycoside antibiotics]	Gestational age of study population was not sufficiently stated.
Heitzer, A. M.	2020	Effect of sensorineural hearing loss on neurocognitive and adaptive functioning in survivors of pediatric embryonal brain tumor	Gestational age of study population was not sufficiently stated.
Helm, K.	1990	Systemic cytomegalovirus in a patient with the keratitis, ichthyosis, and deafness (KID) syndrome	Case report as unsuitable study type.
Hemmingsen, D.	2020	Hearing in Schoolchildren After Neonatal Exposure to a High-Dose Gentamicin Regimen	No comparison of hearing impairment between preterm and full-term born infants.
Henaff, F.	2017	Risk Factors in Children Older Than 5 Years With Pneumococcal Meningitis: Data From a National Network	Gestational age of study population was not sufficiently stated.
Herini, E. S.	2017	Hospital-based surveillance of congenital rubella syndrome in Indonesia	Gestational age of study population was not sufficiently stated.
Herini, E. S.	2018	Clinical profile of congenital rubella syndrome in Yogyakarta, Indonesia	Connection of risk factors and/or hearing outcome depending on gestational age was not established.
Hernandez-Herrera, R. J.	2007	[Hearing screening and diagnosis of hearing loss: high risk versus low risk neonates]	Only investigation of prematurity as risk factor for hearing impairment, other risk factors not analyzed comparing preterm and full-term born infants.
Hernandez, R. N.	2015	Cerebrospinal fluid otorrhea and pseudomonal meningitis in a child with Mondini dysplasia: case report	Case report as unsuitable study type.
Herrera, T. I.	2018	Outcomes of preterm infants treated with hypothermia for hypoxic-ischemic encephalopathy	No comparison of hearing impairment between preterm and full-term born infants.
Hess, C.	2006	[Hearing impairment in children and adolescents with Down's syndrome]	Gestational age of study population was not sufficiently stated.
Hess, M.	1998	Hearing screening in at-risk neonate cohort	Connection of risk factors and/or hearing outcome depending on gestational age was not established.
Hesseling, P. B.	1990	A prospective study of long-term use of amikacin in a paediatrics department. Indications, administration, side-effects, bacterial isolates and resistance	Connection of risk factors and/or hearing outcome depending on gestational age was not established.
Hicks, K. L.	2022	Environmental Factors for Hearing Loss and Middle Ear Disease in Alaska Native Children and Adolescents: A Cross-Sectional Analysis from a Cluster Randomized Trial	Gestational age of study population was not sufficiently stated.
Hicks, T.	1993	Congenital cytomegalovirus infection and neonatal auditory screening	Connection of risk factors and/or hearing outcome depending on gestational age was not established.
Hickson, L. M.	1991	Progressive hearing loss in children with congenital cytomegalovirus	Case report as unsuitable study type.
High Risk Follow-Up Working Group (Kowloon Region)	2008	Neurodevelopmental outcomes of extreme-low-birth-weight infants born between 2001 and 2002	No comparison of hearing impairment between preterm and full-term born infants.
Hindmarsh, G. J.	2000	Gender differences in cognitive abilities at 2 years in ELBW infants. Extremely low birth weight	No comparison of hearing impairment between preterm and full-term born infants.
Hintz, S. R.	2015	Neuroimaging and Neurodevelopmental Outcome in Extremely Preterm Infants	Hearing outcome not examined separately.
Hintz, S. R.	2018	Preterm Neuroimaging and School-Age Cognitive Outcomes	Hearing outcome not examined separately.
Hirsch, Y.	2021	A synonymous variant in MYO15A enriched in the Ashkenazi Jewish population causes autosomal recessive hearing loss due to abnormal splicing	Gestational age of study population was not sufficiently stated.

Hirvonen, M.	2018	Visual and Hearing Impairments After Preterm Birth	Only investigation of gestational age as risk factor for hearing impairment, other risk factors not analyzed comparing preterm and full-term born infants.
Hizli, S.	2011	Sensorineural hearing loss in pediatric celiac patients	Gestational age of study population was not sufficiently stated.
Ho, J. J.	1999	Neurodevelopmental outcome of very low birth weight babies admitted to a Malaysian nursery	No comparison of hearing impairment between preterm and full-term born infants.
Ho, V.	2002	Otoacoustic emissions and tympanometry screening among 0-5 year olds	Gestational age of study population was not sufficiently stated.
Hochberg, I.	2021	Bi-allelic variants in the mitochondrial RNase P subunit PRORP cause mitochondrial tRNA processing defects and pleiotropic multisystem presentations	Gestational age of study population was not sufficiently stated.
Hodgson, A.	2001	Survival and sequelae of meningococcal meningitis in Ghana	Gestational age of study population was not sufficiently stated.
Hoey, A. W.	2017	Management and outcomes of cochlear implantation in patients with congenital cytomegalovirus (cCMV)-related deafness	Gestational age of study population was not sufficiently stated.
Hofer-Martini, S.	2021	Auditory processing in children and adolescents with cleft palate	Gestational age of study population was not sufficiently stated.
Hoffman, H. J.	2019	Kids Nowadays Hear Better Than We Did: Declining Prevalence of Hearing Loss in US Youth, 1966-2010	Gestational age of study population was not sufficiently stated.
Hogan, D. P.	2000	Family factors and social support in the developmental outcomes of very low-birth weight children	Gestational age of study population was not sufficiently stated.
Holborow, C.	1982	A study of deafness in West Africa	Missing control collective without hearing impairment.
Holgers, K. M.	2005	Noise exposure and subjective hearing symptoms among school children in Sweden	Gestational age of study population was not sufficiently stated.
Holster, I. L.	2009	Evaluation of hearing loss after failed neonatal hearing screening	Connection of risk factors and/or hearing outcome depending on gestational age was not established.
Holten, A.	1985	Aetiology of hearing disorders in children at the schools for the deaf	Gestational age of study population was not sufficiently stated.
Homer, J. J.	2000	Neonatal hearing screening using the auditory brainstem response	Missing control collective without hearing impairment.
Homoe, P.	2012	GJB2 (Connexin-26) mutations are not frequent among hearing impaired patients in east Greenland	Gestational age of study population was not sufficiently stated.
Homoe, P.	2008	Hearing outcomes after mobile ear surgery for chronic otitis media in Greenland	Gestational age of study population was not sufficiently stated.
Homoe, P.	2010	Acute mastoiditis in Greenland between 1994-2007	Gestational age of study population was not sufficiently stated.
Hong, S. M.	2016	Analysis of the Prevalence of and Factors Associated with Hearing Loss in Korean Adolescents	Gestational age of study population was not sufficiently stated.
Hore, I.	2007	The management of general and disease specific ENT problems in children with Epidermolysis Bullosa--a retrospective case note review	Gestational age of study population was not sufficiently stated.
Horn, P.	2021	Detecting Hearing Loss in Infants With a Syndrome or Craniofacial Abnormalities Following the Newborn Hearing Screen	Connection of risk factors and/or hearing outcome depending on gestational age was not established.
Hosking, C. S.	1983	The nerve deaf child--intrauterine rubella or not?	Gestational age of study population was not sufficiently stated.
Hosoya, M.	2018	Elongated EABR wave latencies observed in patients with auditory neuropathy caused by OTOF mutation	Gestational age of study population was not sufficiently stated.
Hoth, S.	2009	Universal newborn hearing screening Methodical aspects	Connection of risk factors and/or hearing outcome depending on gestational age was not established.
Houghton, D. J.	1998	Predictors of outcome in children with otitis media with effusion	Gestational age of study population was not sufficiently stated.
Howell, J. B.	2019	An Analysis of Risk Factors in Unilateral Versus Bilateral Hearing Loss	Gestational age of study population was not sufficiently stated.
Hranilovich, J. A.	2020	Brain Magnetic Resonance Imaging in Congenital Cytomegalovirus With Failed Newborn Hearing Screen	Gestational age of study population was not sufficiently stated.
Hrapcak, S.	2016	Hearing Loss in HIV-Infected Children in Lilongwe, Malawi	Gestational age of study population was not sufficiently stated.
Hrncic, N.	2018	Identification of risk factors for hearing impairment in newborns: a hospital based study	Only investigation of prematurity as risk factor for hearing impairment, other risk factors not analyzed comparing preterm and full-term born infants.

Hsu, C. W.	2011	Cerebellar bacterial brain abscess: report of eight cases	Case report as unsuitable study type.
Hsu, R. H.	2019	Genotypic and phenotypic correlations of biotinidase deficiency in the Chinese population	Gestational age of study population was not sufficiently stated.
Hsu, T. R.	2008	Responsiveness of progressive optic pathway tumors to cisplatin-based chemotherapy in children	Gestational age of study population was not sufficiently stated.
Hu, L.	2014	Focal seizures after instillation of cyclomydril to a neonate with congenital CMV infection	Case report as unsuitable study type.
Hua, C. H.	2008	Hearing loss after radiotherapy for pediatric brain tumors: Effect of cochlear dose	Gestational age of study population was not sufficiently stated.
Huang, H. M.	2001	Auditory abnormalities associated with unilateral renal agenesis	Gestational age of study population was not sufficiently stated.
Huang, L.	2017	[Risk Factors and Prognosis of Secondary Epilepsy in Children with Viral Encephalitis]	No comparison of hearing impairment between preterm and full-term born infants.
Huang, L. L.	2017	An analysis of hearing screening test results in 2291 premature infants of Chinese population	No comparison of hearing impairment between preterm and full-term born infants.
Hultcrantz, M.	2003	Ear and hearing problems in Turner's syndrome	Gestational age of study population was not sufficiently stated.
Hultcrantz, M.	1996	Congenital malformation of the inner ear and recurrent meningitis. A case report	Case report as unsuitable study type.
Hultcrantz, M.	1997	Turner's syndrome and hearing disorders in women aged 16-34	Gestational age of study population was not sufficiently stated.
Hulzebos, C. V.	2013	Evaluation of Treatment Thresholds for Unconjugated Hyperbilirubinemia in Preterm Infants: Effects on Serum Bilirubin and on Hearing Loss?	No comparison of hearing impairment between preterm and full-term born infants.
Humberg, A.	2018	Prevalence of Congenital CMV Infection and Antiviral Therapy in Very-Low-Birth-Weight Infants: Observations of the German Neonatal Network	No comparison of hearing impairment between preterm and full-term born infants.
Hunt, L.	2017	Prevalence of paediatric chronic suppurative otitis media and hearing impairment in rural Malawi: A cross-sectional survey	Gestational age of study population was not sufficiently stated.
Hunter, L. L.	1996	High frequency hearing loss associated with otitis media	Gestational age of study population was not sufficiently stated.
Huygen, P. L. M.	1996	Audiovestibular sequelae of congenital cytomegalovirus infection in 3 children presumably representing 3 symptomatically different types of delayed endolymphatic hydrops	Case report as unsuitable study type.
Hyden, D.	1979	Vestibular symptoms in mumps deafness	Gestational age of study population was not sufficiently stated.
Ibekwe, T. S.	2011	Early-onset sensorineural hearing loss in Lassa fever	Gestational age of study population was not sufficiently stated.
Ida, J. B.	2011	Complications in pediatric osseointegrated implantation	Gestational age of study population was not sufficiently stated.
Iftikhar, U.	2013	Risk of hearing loss in children exposed to gentamicin for the treatment of sepsis in young infancy: A community based cohort study in Pakistan	Connection of risk factors and/or hearing outcome depending on gestational age was not established.
Ikehara, Y.	2001	[Congenital rubella syndrome developing after a 1987-1988 epidemic in Japan]	No comparison of hearing impairment between preterm and full-term born infants.
Ikinciogullari, A.	2002	An intensive approach to the treatment of disseminated BCG infection in a SCID patient	Case report as unsuitable study type.
Ilia, S.	2013	Clinical features and outcome of acute otitis media in early infancy	Gestational age of study population was not sufficiently stated.
Illiano, M.	2021	Long-term morbidity and mortality in 2-year hepatoblastoma survivors treated with SIOPEL risk-adapted strategies	Chemotherapy as exclusion criterion.
Ilveskoski, I.	1996	Ototoxicity in children with malignant brain tumors treated with the "8 in 1" chemotherapy protocol	Gestational age of study population was not sufficiently stated.
Imamura, T.	2011	Oral valganciclovir treatment for congenital cytomegalovirus infection	Case report as unsuitable study type.
Imashuku, S.	2007	Sensorineural hearing loss in a case of familial hemophagocytic lymphohistiocytosis	Case report as unsuitable study type.
Inaba, H.	2001	Polio vaccine virus-associated meningoencephalitis in an infant with transient hypogammaglobulinemia	Case report as unsuitable study type.
Inaba, Y.	2016	Correlation Between White Matter Lesions and Intelligence Quotient. in Patients With Congenital Cytomegalovirus Infection	No comparison of hearing impairment between preterm and full-term born infants.

Inci, A.	2021	Clinical and event-based outcomes of patients with mucopolysaccharidosis VI receiving enzyme replacement therapy in Turkey: a case series	Gestational age of study population was not sufficiently stated.
Indelicato, D. J.	2021	Local Control After Proton Therapy for Pediatric Chordoma	Gestational age of study population was not sufficiently stated.
Inoue, H.	2018	Neurodevelopmental Outcomes in Infants With Birth Weight <= 500 g at 3 Years of Age	No comparison of hearing impairment between preterm and full-term born infants.
Inscoe, J. R.	2016	Additional difficulties associated with aetiologies of deafness: outcomes from a parent questionnaire of 540 children using cochlear implants	Gestational age of study population was not sufficiently stated.
Ionescu, C.	2016	Secondary congenital aphakia	Case report as unsuitable study type.
Iossa, S.	2015	Phenotypic and genetic characterization of a family carrying two Xq21.1-21.3 interstitial deletions associated with syndromic hearing loss	Case report as unsuitable study type.
Iseri, M.	2013	Cerebrospinal fluid otorrhea and recurrent bacterial meningitis in a pediatric case with Mondini dysplasia	Case report as unsuitable study type.
Iskander, I.	2014	Serum Bilirubin and Bilirubin/Albumin Ratio as Predictors of Bilirubin Encephalopathy	Gestational age of study population does not meet defined inclusion criteria for prematurity (<37 weeks) and/or full-term birth (≥37 weeks) with cut-off stated at 37 weeks.
Islam, M. Z.	2021	Otological Presentation of Obstructive Nasal Lesions: A Study of 50 Cases	Gestational age of study population was not sufficiently stated.
Ito, H.	1984	Auditory brainstem response in NICU infants	Connection of risk factors and/or hearing outcome depending on gestational age was not established.
Ito, K.	2005	Nonsyndromic isolated unilateral cochlear nerve aplasia without narrow internal auditory meatus: A previously overlooked cause of unilateral profound deafness in childhood	Case report as unsuitable study type.
Ito, Y.	2013	Risk factors for poor outcome in congenital cytomegalovirus infection and neonatal herpes on the basis of a nationwide survey in Japan	Only investigation of gestational age as risk factor for hearing impairment, other risk factors not analyzed comparing preterm and full-term born infants.
Iuorio, J. L.	1984	Retrospective diagnosis of congenital rubella	Connection of risk factors and/or hearing outcome depending on gestational age was not established.
Iwasaki, S.	2007	Audiological outcome of infants with congenital cytomegalovirus infection in a prospective study	Gestational age of study population was not sufficiently stated.
Iwatate, K.	2017	Population Characteristics and Progressive Disability in Neurofibromatosis Type 2	Gestational age of study population was not sufficiently stated.
Jaba, S.	2020	UNDIFFERENTIATED NASOPHARYNGEAL CARCINOMA IN CHILDREN AND ADOLESCENTS: A REPORT OF 68 CASES IN A MOROCCAN CENTER	Gestational age of study population was not sufficiently stated.
Jacob-Corteletti, L. C.	2018	Acoustic Reflex Testing in Neonatal Hearing Screening and Subsequent Audiological Evaluation	Hearing outcome not examined.
Jacob, A.	1997	Hearing impairment and otitis media in a rural primary school in south India	Gestational age of study population was not sufficiently stated.
Jacobson, G. P.	1990	Infant hearing screening 1984 to 1989: the Henry Ford Hospital experience	Gestational age of study population was not sufficiently stated.
Jadavji, T.	1986	Sequelae of acute bacterial meningitis in children treated for seven days	Gestational age of study population was not sufficiently stated.
Jadia, S.	2019	Role of Otoacoustic Emissions in Hearing Assessment of Neonates: A Prospective Observational Study	Missing control collective without hearing impairment.
Jafari, Z.	2007	Auditory neuropathy: Audiologic and clinical evidence	No comparison of hearing impairment between preterm and full-term born infants.
Jafari, Z.	2007	The ages of suspicion, diagnosis, amplification, and intervention in deaf children	Missing control collective without hearing impairment.
Jahan, I.	2020	Epidemiology of cerebral palsy in Sumba Island, Indonesia	Connection of risk factors and/or hearing outcome depending on gestational age was not established.
Jaimes, C.	2019	Does 3-T fetal MRI induce adverse acoustic effects in the neonate? A preliminary study comparing postnatal auditory test performance of fetuses scanned at 1.5 and 3T	No comparison of hearing impairment between preterm and full-term born infants.
Jais, J. P.	2003	X-linked Alport syndrome: Natural history and genotype-phenotype correlations in girls and women belonging to 195 families: A "European community Alport syndrome concerted action" study	Gestational age of study population was not sufficiently stated.
Jakubikova, J.	2003	Identification of hearing loss in newborns by transient otoacoustic emissions	Gestational age of study population was not sufficiently stated.

Jakubikova, J.	2009	Newborn hearing screening and strategy for early detection of hearing loss in infants	Gestational age of study population was not sufficiently stated.
Jalali, M. M.	2020	Prevalence of Hearing Loss among School-Age Children in the North of Iran	Gestational age of study population was not sufficiently stated.
James, A. L.	2011	The Assessment of Olivocochlear Function in Neonates with Real-Time Distortion Product Otoacoustic Emissions	No comparison of hearing impairment between preterm and full-term born infants.
James, A. L.	2020	The limitation of risk factors as a means of prognostication in auditory neuropathy spectrum disorder of perinatal onset	Only investigation of prematurity as risk factor for hearing impairment, other risk factors not analyzed comparing preterm and full-term born infants.
Jamil, K. M.	2015	Effectiveness Study of Paromomycin IM Injection (PMIM) for the Treatment of Visceral Leishmaniasis (VL) in Bangladesh	Gestational age of study population was not sufficiently stated.
Janeschik, S.	2013	Influence of etiologic factors on speech perception of cochlear-implanted children	Missing control collective without hearing impairment.
Janky, K. L.	2018	Predictive Factors for Vestibular Loss in Children With Hearing Loss	Gestational age of study population was not sufficiently stated.
Jansen, C. F. M.	2005	Treatment of symptomatic congenital cytomegalovirus infection with valganciclovir	Case report as unsuitable study type.
Januario, G. C.	2016	Health Vulnerability Index and newborn hearing screening: urban inequality	No comparison of hearing impairment between preterm and full-term born infants.
Jatsho, J.	2022	An unusual presentation of scrub typhus in a child: a case report	Case report as unsuitable study type.
Jatto, M. E.	2020	Pediatric Hearing Thresholds Post-bacterial Meningitis	Gestational age of study population was not sufficiently stated.
Jawed, R.	2020	DENTAL CARIES AND ITS DETERMINANTS AMONG CHILDREN WITH SPECIAL HEALTH CARE NEEDS IN DISTRICT KARACHI, PAKISTAN	Gestational age of study population was not sufficiently stated.
Jayagobi, P. A.	2020	Hearing screening outcome in neonatal intensive care unit graduates from a tertiary care centre in Singapore	Gestational age of study population was not sufficiently stated.
Jayarajan, V.	1999	Delayed deterioration of hearing following bacterial meningitis	Case report as unsuitable study type.
Jazmati, D.	2021	Feasibility of Proton Beam Therapy for Infants with Brain Tumours: Experiences from the Prospective KiProReg Registry Study	Gestational age of study population was not sufficiently stated.
Jecmenica, J. R.	2015	Characteristics of Brain Stem Auditory Evoked Potentials in Children With Hearing Impairment Due to Infectious Diseases	Gestational age of study population was not sufficiently stated.
Jeevan, D. S.	2015	Cerebrospinal fluid leaks and encephaloceles of temporal bone origin: nuances to diagnosis and management	Gestational age of study population was not sufficiently stated.
Jeffery, H.	1977	Deafness after bacterial meningitis	Gestational age of study population was not sufficiently stated.
Jeffries, L.	2018	A novel SAMD9 mutation causing MIRAGE syndrome: An expansion and review of phenotype, dysmorphology, and natural history	Case report as unsuitable study type.
Jehanne, M.	2009	Analysis of Ototoxicity in Young Children Receiving Carboplatin in the Context of Conservative Management of Unilateral or Bilateral Retinoblastoma	Chemotherapy as defined reason for exclusion.
Jensema, C.	1975	Children in educational programs for the hearing impaired whose impairment was caused by mumps	Gestational age of study population was not sufficiently stated.
Jensen, J. S.	2021	Examination of hearing loss among school-aged children in Greenland	Gestational age of study population was not sufficiently stated.
Jensen, R. G.	2013	The risk of hearing loss in a population with a high prevalence of chronic suppurative otitis media	Gestational age of study population was not sufficiently stated.
Jeong, J.	2021	Neonatal and maternal risk factors for hearing loss in children based on population-based data of Korea	Gestational age of study population was not sufficiently stated.
Jeong, S. W.	2016	Delayed-onset hearing loss in pediatric candidates for cochlear implantation	Gestational age of study population was not sufficiently stated.
Jeyakumar, A.	2009	Otolaryngologic manifestations of mitochondrial cytopathies	Gestational age of study population was not sufficiently stated.
Ji, K.	2020	Mitochondrial encephalopathy Due to a Novel Pathogenic Mitochondrial tRNA(Gln) m.4349C>T Variant	Gestational age of study population was not sufficiently stated.
Jiang, F.	2020	Etiology of Childhood Bilatera Sensorineural Hearing Loss in Shandong Province, China	Connection of risk factors and/or hearing outcome depending on gestational age was not established.
Jiang, Z. D.	1999	Outcome of brain stem auditory electrophysiology in children who survive purulent meningitis	Gestational age of study population was not sufficiently stated.

Jiang, Z. D.	2017	Auditory impairment in infants with neonatal chronic lung disease is alleviated after term	Connection of risk factors and/or hearing outcome depending on gestational age was not established.
Jiang, Z. D.	2010	Relationship between brainstem auditory function during the neonatal period and depressed Apgar score	No comparison of hearing impairment between preterm and full-term born infants.
Jiang, Z. D.	2001	Hearing impairment in preterm very low birthweight babies detected at term by brainstem auditory evoked responses	Hearing impairment compared for preterm infants with risk factor to full-term born infants without risk factor. Therefore no relevant information for review question.
Jiang, Z. D.	2009	Changes in BAER wave amplitudes in relation to total serum bilirubin level in term neonates	No comparison of hearing impairment between preterm and full-term born infants.
Jiang, Z. D.	2007	Changes in brainstem auditory evoked response latencies in term neonates with hyperbilirubinemia	No comparison of hearing impairment between preterm and full-term born infants.
Jiang, Z. D.	2004	One-third of term babies after perinatal hypoxia-ischaemia have transient hearing impairment: dynamic change in hearing threshold during the neonatal period	No comparison of hearing impairment between preterm and full-term born infants.
Jiang, Z. D.	2015	Changes in hearing threshold between 28 and 42 weeks of age in babies born at under 30 weeks of gestation	No comparison of hearing impairment between preterm and full-term born infants.
Jim, W. T.	2015	Outcome of Preterm Infants With Postnatal Cytomegalovirus Infection via Breast Milk A Two-Year Prospective Follow-Up Study	No comparison of hearing impairment between preterm and full-term born infants.
Jimenez-Romero, M. S.	2016	Language Impairment Resulting from a de novo Deletion of 7q32.1q33	Case report as unsuitable study type.
Jin, H. D.	2017	Long-term Visual and Ocular Sequelae in Patients With Congenital Cytomegalovirus Infection	No comparison of hearing impairment between preterm and full-term born infants.
Jin, L. J.	2007	Mitochondrial tRNA(Ser(UCN)) gene is the hot spot for mutations associated with aminoglycoside-induced and non-syndromic hearing loss	Gestational age of study population was not sufficiently stated.
Jin, Y.	2022	Analysis of the Results of Cytomegalovirus Testing Combined with Genetic Testing in Children with Congenital Hearing Loss	Gestational age of study population was not sufficiently stated.
Jivraj, I.	2014	Identification of ocular and auditory manifestations of congenital rubella syndrome in mbingo	Gestational age of study population was not sufficiently stated.
Johansson Kostenniemi, U.	2021	Psychiatric Disabilities and Other Long-term Consequences of Childhood Bacterial Meningitis	Gestational age of study population was not sufficiently stated.
Johnson, J. L.	2005	A multicenter evaluation of how many infants with permanent hearing loss pass a two-stage otoacoustic emissions/automated auditory brainstem response newborn hearing screening protocol	Gestational age of study population was not sufficiently stated.
Johnson, K.	2016	High-Frequency Sensorineural Hearing Loss in Children	Only investigation of prematurity as risk factor for hearing impairment, other risk factors not analyzed comparing preterm and full-term born infants.
Johnson, L. C.	2018	Age and Other Factors Affecting the Outcome of AABR Screening in Neonates	Only investigation of prematurity as risk factor for hearing impairment, other risk factors not analyzed comparing preterm and full-term born infants.
Johnson, R. F.	2010	Genetic mutations and aminoglycoside-induced ototoxicity in neonates	No comparison of hearing impairment between preterm and full-term born infants.
Johnson, S.	2015	Neurodevelopmental outcomes following late and moderate prematurity: a population-based cohort study	Only investigation of prematurity as risk factor for hearing impairment, other risk factors not analyzed comparing preterm and full-term born infants.
Johnson, S. J.	1986	Prevalence of sensorineural hearing loss in premature and sick term infants with perinatally acquired cytomegalovirus infection	Gestational age of study population does not meet defined inclusion criteria for prematurity (<37 weeks) and/or full-term birth (≥37 weeks) with cut-off stated at 37 weeks.
Jonard, L.	2008	A familial case of Keratitis-Ichthyosis-Deafness (KID) syndrome with the GJB2 mutation G45E	Case report as unsuitable study type.
Jones, F. E.	1977	H. influenzae meningitis treated with ampicillin or chloramphenicol, and subsequent hearing loss	Gestational age of study population was not sufficiently stated.
Jones, R.	2006	Are there health benefits from improving basic nutrition in a remote Aboriginal community?	Gestational age of study population was not sufficiently stated.
Jones, R.	1995	Controlled trial of dexamethasone in neonatal chronic lung disease: a 3-year follow-up	No comparison of hearing impairment between preterm and full-term born infants.
Joubert, K.	2019	Contributing factors to high prevalence of hearing impairment in the Elias Motsoaledi Local Municipal area, South Africa: A rural perspective	Gestational age of study population was not sufficiently stated.
Jubran, R. F.	2020	A single-arm study of systemic and sub-Tenon chemotherapy for Groups C and D intraocular retinoblastoma: A Children's Oncology Group study (ARET 0231)	Gestational age of study population was not sufficiently stated.
Judge, P. D.	2019	Medical Referral Patterns and Etiologies for Children With Mild-to-Severe Hearing Loss	Only investigation of prematurity as risk factor for hearing impairment, other risk factors not analyzed comparing preterm and full-term born infants.

Jullien, V.	2016	Pilot Evaluation of the Population Pharmacokinetics of Bumetanide in Term Newborn Infants With Seizures	No comparison of hearing impairment between preterm and full-term born infants.
Jun, H. J.	2015	The prevalence of hearing loss in South Korea: data from a population-based study	Gestational age of study population was not sufficiently stated.
Juneja, M.	2011	Atypical Cogan syndrome mimicking acute rheumatic fever	Case report as unsuitable study type.
Jung, E. Y.	2017	Relation between amniotic fluid infection or cytokine levels and hearing screen failure in infants at 32 wk gestation or less	No comparison of hearing impairment between preterm and full-term born infants.
Jure, R.	1991	HEARING-IMPAIRED AUTISTIC-CHILDREN	Missing control collective without hearing impairment.
Kaaijk, C. K.	1977	Longitudinal Study of hearing loss in childhood. Relationship between hearing impairment, poor learning and family background	Gestational age of study population was not sufficiently stated.
Kaaresen, P. I.	1995	Prognostic factors in childhood bacterial meningitis	Gestational age of study population was not sufficiently stated.
Kadambari, S.	2019	Enterovirus and parechovirus meningitis in infants younger than 90 days old in the UK and Republic of Ireland: a British Paediatric Surveillance Unit study	Gestational age of study population was not sufficiently stated.
Kaewboonchoo, O.	1998	Hearing impairment among young Chinese in an urban area	Gestational age of study population was not sufficiently stated.
Kaga, K.	1998	Unilateral total loss of auditory and vestibular function as a complication of mumps vaccination	Case report as unsuitable study type.
Kaga, K.	2003N1103	Auditory agnosia in children after herpes encephalitis	Case report as unsuitable study type.
Kaga, M.	2000	Long-term follow-up of auditory agnosia as a sequel of herpes encephalitis in a child	Case report as unsuitable study type.
Kaksonen, R.	2000	Hereditary hearing loss--the role of environmental factors	Gestational age of study population was not sufficiently stated.
Kalambe, S.		Comparison of Otoacoustic Emission (OAE) and Brainstem Evoked Response Audiometry (BERA) in High Risk Infants and Children under 5 Years of Age for Hearing Assessment in Western India: A Modification in Screening Protocol	Missing control collective without hearing impairment.
Kallio, M. J.	1994	The effect of a recent previous visit to a physician on outcome after childhood bacterial meningitis	Gestational age of study population was not sufficiently stated.
Kam, A. C. S.	2014	Automated hearing screening for preschool children	Gestational age of study population was not sufficiently stated.
Kanchanalarp, C.	2006	Indication and surgical consideration of cochlear implantation at Ramathibodi Hospital	Gestational age of study population was not sufficiently stated.
Kandiah, R.	2019	A rare entity in a child: Unilateral sensorineural hearing loss with an ipsilateral vascular loop - Is there any correlation? A case report and literature review	Case report as unsuitable study type.
Kanecki, K.	2020	Congenital cytomegalovirus infections in Poland - a national hospital register-based study	Gestational age of study population was not sufficiently stated.
Kaneko, M.	2013	Maternal IgG avidity, IgM and ultrasound abnormalities: combined method to detect congenital cytomegalovirus infection with sequelae	Gestational age of study population was not sufficiently stated.
Kanemura, T.	1979	Frontometaphyseal dysplasia with congenital urinary tract malformations	Case report as unsuitable study type.
Kanerva, M.	2008	Human herpesvirus-6 and -7 DNA in cerebrospinal fluid of facial palsy patients	Gestational age of study population was not sufficiently stated.
Kang, J. W.	2010	Clinical and radiologic evaluation of cytomegalovirus-induced thrombocytopenia in infants between 1 and 6 months of age	Connection of risk factors and/or hearing outcome depending on gestational age was not established.
Kang, M. Y.	2012	Changes in the Hearing Thresholds of Infants Who Failed the Newborn Hearing Screening Test and in Infants Treated in the Neonatal Intensive Care Unit	Gestational age of study population was not sufficiently stated.
Kanji, A.	2012	The occurrence of high-risk factors for hearing loss in very-low-birth-weight neonates: a retrospective exploratory study of targeted hearing screening	No comparison of hearing impairment between preterm and full-term born infants.
Kanji, A.	2021	Risk factors for hearing impairment in neonates in South Africa: scoping the context for newborn hearing screening planning	Missing control collective without hearing impairment.
Kanne, T. J.	1999	Potential pitfalls of initiating a newborn hearing screening program	Gestational age of study population was not sufficiently stated.
Kanra, G.	2002	Mumps meningoencephalitis effect on hearing	Gestational age of study population was not sufficiently stated.

Kanra, G. Y.	1995	BENEFICIAL-EFFECTS OF DEXAMETHASONE IN CHILDREN WITH PNEUMOCOCCAL MENINGITIS	Gestational age of study population was not sufficiently stated.
Kantaputra, P. N.	1998	Rapp-Hodgkin syndrome with palmoplantar keratoderma, glossy tongue, congenital absence of lingual frenum and of sublingual caruncles: newly recognized findings	Case report as unsuitable study type.
Kapadia, M.	2022	UNHS: A Decade Long Feasibility and Sustenance Study from a Tertiary Care Hospital in India	Connection of risk factors and/or hearing outcome depending on gestational age was not established.
Kaplama, M. E.	2020	The results of hearing screening in refugee school children living in Sanliurfa/Turkey and the related risk factors	Gestational age of study population was not sufficiently stated.
Kaplama, M. E.	2020	Newborn hearing screening results: Comparison of Syrian and Turkish newborns; factors influencing the difference	Missing control collective without hearing impairment.
Kaplan, D. M.	1996	Audiometric findings in children with chronic suppurative otitis media without cholesteatoma	Gestational age of study population was not sufficiently stated.
Kaplan, S. L.	1984	Onset of hearing loss in children with bacterial meningitis	Gestational age of study population was not sufficiently stated.
Kaplan, S. L.	1984	Prospective comparative trial of moxalactam versus ampicillin or chloramphenicol for treatment of Haemophilus influenzae type b meningitis in children	Gestational age of study population was not sufficiently stated.
Kaplan, S. L.	2006	Multicenter surveillance of invasive meningococcal infections in children	Gestational age of study population was not sufficiently stated.
Kaplan, S. L.	1986	Association between preadmission oral antibiotic therapy and cerebrospinal fluid findings and sequelae caused by Haemophilus influenzae type b meningitis	Gestational age of study population was not sufficiently stated.
Kapoor, R. K.	1996	Brainstem auditory evoked response (BAER) in childhood bacterial meningitis	Gestational age of study population was not sufficiently stated.
Karaca, C. T.	2014	Is hearing loss in infants associated with risk factors? Evaluation of the frequency of risk factors	Gestational age of study population was not sufficiently stated.
Karademir, F.	2010	Bronchopulmonary dysplasia, ophtalmological and auditory problems in prematurely born infants	No comparison of hearing impairment between preterm and full-term born infants.
Karampalis, C.	2014	Surgical treatment of scoliosis in Treacher Collins syndrome: a case report	Case report as unsuitable study type.
Karanja, B. W.	2013	Risk Factors for Hearing Loss in Children following Bacterial Meningitis in a Tertiary Referral Hospital	Gestational age of study population was not sufficiently stated.
Karanja, B. W.	2014	Prevalence of hearing loss in children following bacterial meningitis in a tertiary referral hospital	Gestational age of study population was not sufficiently stated.
Karimian, P.	2016	Prevalence, Characteristics, and One-Year Follow-Up of Congenital Cytomegalovirus Infection in Isfahan City, Iran	Connection of risk factors and/or hearing outcome depending on gestational age was not established.
Karltorp, E.	2012	Congenital cytomegalovirus infection - a common cause of hearing loss of unknown aetiology	Gestational age of study population was not sufficiently stated.
Karltorp, E.	2014	Impaired balance and neurodevelopmental disabilities among children with congenital cytomegalovirus infection	No comparison of hearing impairment between preterm and full-term born infants.
Karmody, C. S.	1968	Subclinical maternal rubella and congenital deafness	Gestational age of study population was not sufficiently stated.
Karppinen, M.	2015	Hearing impairment after childhood bacterial meningitis dependent on etiology in Luanda, Angola	Gestational age of study population was not sufficiently stated.
Kashiwagi, Y.	2011	Efficacy of prolonged valganciclovir therapy for congenital cytomegalovirus infection	Case report as unsuitable study type.
Kaspar, A.	2021	Development of a Risk-Factor Questionnaire for the Infant Ear and Hearing Program in Samoa	Review, sources screened for suitable literature for review question.
Kaspar, A.	2018	Prevalence of otitis media and risk-factors for sensorineural hearing loss among infants attending Child Welfare Clinics in the Solomon Islands	Connection of risk factors and/or hearing outcome depending on gestational age was not established.
Kasuga, M.	2021	Frequency and natural course of congenital cytomegalovirus-associated hearing loss in children	Gestational age of study population was not sufficiently stated.
Kasztelewicz, B.	2017	Cytokine gene polymorphism associations with congenital cytomegalovirus infection and sensorineural hearing loss	No comparison of hearing impairment between preterm and full-term born infants.
Kataoka, Y.	2011	[Progressive or delayed early-onset pediatric sensorineural hearing loss]	No comparison of hearing impairment between preterm and full-term born infants.
Kataoka, Y.	2020	Prevalence and risk factors for delayed-onset hearing loss in early childhood: A population-based observational study in Okayama Prefecture, Japan	Gestational age of study population was not sufficiently stated.

Kato, K.	2013	Progressive hearing loss following acquired cytomegalovirus infection in an immunocompromised child	Case report as unsuitable study type.
Katsushika, M.	2018	Outcomes of cochlear implantations for mumps deafness: A report of four pediatric cases	Case report as unsuitable study type.
Katz, T. A.	2022	Severity of Bronchopulmonary Dysplasia and Neurodevelopmental Outcome at 2 and 5 Years Corrected Age	Connection of risk factors and/or hearing outcome depending on gestational age was not established.
Kavaliotis, J.	1989	Treatment of childhood bacterial meningitis with ceftriaxone once daily: open, prospective, randomized, comparative study of short-course versus standard-length therapy	Gestational age of study population was not sufficiently stated.
Kavitha, Y.	2017	A Study of Effect of Consanguinity on Cochlear Morphology in Patients with Congenital Bilateral Profound Sensorineural Hearing Loss	Gestational age of study population was not sufficiently stated.
Kawada, J. I.	2015	Viral load in children with congenital cytomegalovirus infection identified on newborn hearing screening	Gestational age of study population was not sufficiently stated.
Kawashiro, N.	1996	Delayed post-neonatal intensive care unit hearing disturbance	No comparison of hearing impairment between preterm and full-term born infants.
Kayiran, S. M.	2010	Newborn hearing screening in the neonatal intensive care unit: a private hospital experience	Only investigation of prematurity as risk factor for hearing impairment, other risk factors not analyzed comparing preterm and full-term born infants.
Keane, W. M.	1979	Meningitis and hearing loss in children	Gestational age of study population was not sufficiently stated.
Kecskemeti, N.	2019	Etiological factors of sensorineural hearing loss in children after cochlear implantation	No comparison of hearing impairment between preterm and full-term born infants.
Keefe, D. H.	2000	Identification of neonatal hearing impairment: Ear-canal measurements of acoustic admittance and reflectance in neonates	Gestational age of study population was not sufficiently stated.
Keihanidost, Z.	2018	Risk Factors for Hearing Loss and Its Prevalence in Neonates Older than 6 Months with History of Hospitalization in Intensive Care Unit	Gestational age of study population was not sufficiently stated.
Keilty, D.	2021	Hearing Loss After Radiation and Chemotherapy for CNS and Head-and-Neck Tumors in Children	Gestational age of study population was not sufficiently stated.
Keith, C. G.	1991	Congenital rubella infection from reinfection of previously immunised mothers	Case report as unsuitable study type.
Kellner, J. D.	2002	Outcome of penicillin-nonsusceptible Streptococcus pneumoniae meningitis: a nested case-control study	Connection of risk factors and/or hearing outcome depending on gestational age was not established.
Kelly, E. N.	2018	Inhaled and systemic steroid exposure and neurodevelopmental outcome of preterm neonates	No comparison of hearing impairment between preterm and full-term born infants.
Kenna, M. A.	2001	Connexin 26 studies in patients with sensorineural hearing loss	Gestational age of study population was not sufficiently stated.
Kennedy, C.	2014	Quality of survival and growth in children and young adults in the PNET4 European controlled trial of hyperfractionated versus conventional radiation therapy for standard-risk medulloblastoma	Gestational age of study population was not sufficiently stated.
Kennedy, W. A.	1991	The role of corticosteroid therapy in children with pneumococcal meningitis	Gestational age of study population was not sufficiently stated.
Kepekci, A. H.	2018	Evaluation of 1808 Newborns Hearing Screening Outcome	Gestational age of study population was not sufficiently stated.
Kermorvant-Duchemin, E.	2008	Outcome and prognostic factors in neonates with septic shock	Hearing outcome not examined separately.
Khabori, M. A.	2008	Consanguinity and deafness in Omani children	Gestational age of study population was not sufficiently stated.
Khaimook, W.	2008	The high-risk neonatal hearing screening program in Songklanagarind Hospital	Connection of risk factors and/or hearing outcome depending on gestational age was not established.
Khairi, M. D.	2005	Hearing screening of infants in Neonatal Unit, Hospital Universiti Sains Malaysia using transient evoked otoacoustic emissions	Gestational age of study population was not sufficiently stated.
Khairi, M. D. M.	2009	Auditory neuropathy: three cases among a group with sensorineural hearing loss	Case report as unsuitable study type.
Khalid, S.	2015	Spontaneous improvement in sensorineural hearing loss developed as a complication of neonatal hyperbilirubinemia	Case report as unsuitable study type.
Khalique, N.	2022	Clinico-epidemiological study of safe and unsafe chronic suppurative otitis media	Case report as unsuitable study type.
Khan, S.	2016	Hypomorphic MKS1 mutation in a Pakistani family with mild Joubert syndrome and atypical features: Expanding the phenotypic spectrum of MKS1-related ciliopathies	Gestational age of study population was not sufficiently stated.

Khandaker, G.	2019	Epidemiology of cerebral palsy in Bangladesh: a population-based surveillance study	Gestational age of study population was not sufficiently stated.
Khandaker, G.	2014	Infectious Causes of Childhood Disability: Results from a Pilot Study in Rural Bangladesh	Gestational age of study population was not sufficiently stated.
Khandekar, R.	2004	An epidemiological and clinical study of ocular manifestations of congenital rubella syndrome in Omani children	Gestational age of study population was not sufficiently stated.
Khetpal, V.	2007	Cortical visual impairment: Etiology, associated findings, and prognosis in a tertiary care setting	Gestational age of study population was not sufficiently stated.
Khosroshahi, H. E.	1989	Keutel syndrome: a report of four cases	Case report as unsuitable study type.
Khowaja, A. R.	2013	Mortality and neurodevelopmental outcomes of acute bacterial meningitis in children aged <5 years in Pakistan	Connection of risk factors and/or hearing outcome depending on gestational age was not established.
Khurana, S.	2017	Long-term neurodevelopment outcome of caffeine versus aminophylline therapy for apnea of prematurity	No comparison of hearing impairment between preterm and full-term born infants.
Kiatchoosakun, P.	2012	Incidence and risk factors associated with hearing loss in high-risk neonates in Srinagarind Hospital	Gestational age of study population was not sufficiently stated.
Kielmovitch, I. H.	1988	Unilateral sensorineural deafness in children	Case report as unsuitable study type.
Kiese-Himmel, C.	2001	[Unilateral hearing loss in childhood. An empirical analysis comparing bilateral hearing loss]	Gestational age of study population was not sufficiently stated.
Kilic, K.	2018	Evaluation of Hearing in Children with Metabolic Syndrome	Gestational age of study population was not sufficiently stated.
Kilpi, T.	1995	Oral glycerol and intravenous dexamethasone in preventing neurologic and audiologic sequelae of childhood bacterial meningitis. The Finnish Study Group	Gestational age of study population was not sufficiently stated.
Kim, A. R.	2017	The Analysis of A Frequent TMPRSS3 Allele Containing P.V116M and P.V291L in A Cis Configuration among Deaf Koreans	Gestational age of study population was not sufficiently stated.
Kim, J. H.	2020	Renal Syndromic Hearing Loss Is Common in Childhood-onset Chronic Kidney Disease	Only investigation of prematurity as risk factor for hearing impairment, other risk factors not analyzed comparing preterm and full-term born infants.
Kim, J. H.	2020	Audiologic Status of Children with Confirmed Cytomegalovirus Infection: a Case Series	Gestational age of study population was not sufficiently stated.
Kim, S. H.	2020	Prevalence and clinical aspects of hearing loss among the South Korean adolescent: Data from a population-based study	Gestational age of study population was not sufficiently stated.
Kim, S. H.	2017	Maternal and Placental Factors Associated with Congenital Hearing Loss in Very Preterm Neonates	No comparison of hearing impairment between preterm and full-term born infants.
Kim, S. Y.	2018	Risk factors for failure in the newborn hearing screen test in very preterm twins	No comparison of hearing impairment between preterm and full-term born infants.
Kim, S. Y.	2019	Migraine increases the proportion of sudden sensorineural hearing loss: A longitudinal follow-up study	Gestational age of study population was not sufficiently stated.
Kim, S. Y.	2020	Herpes zoster does not increase the risk of sudden sensory neural hearing loss: a longitudinal follow-up study using a national sample cohort	Gestational age of study population was not sufficiently stated.
Kim, S. Y.	2020	Novel Splice Site Pathogenic Variant of EFTUD2 Is Associated with Mandibulofacial Dysostosis with Microcephaly and Extracranial Symptoms in Korea	Case report as unsuitable study type.
Kim, S. Y.	2018	Hearing impairment increases the risk of distal radius, hip, and spine fractures: A longitudinal follow-up study using a national sample cohort	Gestational age of study population was not sufficiently stated.
Kim, S. Y.	2013	Prevalence of p.V37I variant of GJB2 in mild or moderate hearing loss in a pediatric population and the interpretation of its pathogenicity	Gestational age of study population was not sufficiently stated.
Kim, S. Y.	2018	Sudden sensory neural hearing loss is not predictive of myocardial infarction: A longitudinal follow-up study using a national sample cohort	Gestational age of study population was not sufficiently stated.
Kim, Y. S.	2022	Full etiologic spectrum of pediatric severe to profound hearing loss of consecutive 119 cases	Gestational age of study population was not sufficiently stated.
Kimani, J. W.	2010	Sensorineural Hearing Loss in a Pediatric Population Association of Congenital Cytomegalovirus Infection With Intracranial Abnormalities	Gestational age of study population was not sufficiently stated.
Kimberlin, D. W.	2015	Valganciclovir for symptomatic congenital cytomegalovirus disease	No comparison of hearing impairment between preterm and full-term born infants.
Kimberlin, D. W.	2003	Effect of ganciclovir therapy on hearing in symptomatic congenital cytomegalovirus disease involving the central nervous system: a randomized, controlled trial	Connection of risk factors and/or hearing outcome depending on gestational age was not established.

Kimitsuki, T.	1999	Congenital malformation of the inner ear associated with recurrent meningitis	Gestational age of study population was not sufficiently stated.
King, S. M.	1994	Dexamethasone therapy for bacterial meningitis: Better never than late?	Gestational age of study population was not sufficiently stated.
Kinoshita, T.	2022	A case of acute focal bacterial nephritis caused by methicillin-resistant Staphylococcus saprophyticus in a 13-year-old adolescent girl treated with daptomycin	Case report as unsuitable study type.
Kirk, M.	1987	Sensorineural hearing loss and mumps	Gestational age of study population was not sufficiently stated.
Kirkim, G.	2008	The frequency of auditory neuropathy detected by universal newborn hearing screening program	Gestational age of study population was not sufficiently stated.
Kirkpatrick, B.	1994	A review of the clinical presentation, laboratory features, antimicrobial therapy and outcome of 77 episodes of pneumococcal meningitis occurring in children and adults	Gestational age of study population was not sufficiently stated.
Kirkwood, A.	2007	Is gentamicin ototoxic to the fetus?	Gestational age of study population was not sufficiently stated.
Kirtane, M. V.	2010	Cochlear stenting: how I do it	Case report as unsuitable study type.
Kishon-Rabin, L.	2015	Delay in auditory behaviour and preverbal vocalization in infants with unilateral hearing loss	Connection of risk factors and/or hearing outcome depending on gestational age was not established.
Kitamura, A.	2007	A familial childhood-onset relapsing nephrotic syndrome	Gestational age of study population was not sufficiently stated.
Kitano, M.	2021	Retrospective study of cochlear implantations at a single facility focusing on postoperative complications	Gestational age of study population was not sufficiently stated.
Kitazawa, K.	2004	Mondini dysplasia and recurrent bacterial meningitis in a girl with relapsing Langerhans cell histiocytosis	Case report as unsuitable study type.
Kitchen, W. H.	1983	Collaborative study of very-low-birth-weight infants. Correlation of handicap with risk factors	No comparison of hearing impairment between preterm and full-term born infants.
Kitsantas, P.	2013	Chronic physical health conditions among children of different racial/ethnic backgrounds	Gestational age of study population was not sufficiently stated.
Kivekas, I.	2015	Unilateral common cavity deformity: Recurrent meningitis due to insufficient newborn hearing screening	Case report as unsuitable study type.
Kiykim, E.	2015	Biotinidase deficiency mimicking primary immune deficiencies	Case report as unsuitable study type.
Kjollesdal, M.		Variation in disease in children according to immigrant background	Gestational age of study population was not sufficiently stated.
Kline, M. W.	1989	Sudden-onset deafness. Serodiagnosis of recent meningococcal infection	Case report as unsuitable study type.
Klis, S.	2014	Long Term Streptomycin Toxicity in the Treatment of Buruli Ulcer: Follow-up of Participants in the BURULICO Drug Trial	Gestational age of study population was not sufficiently stated.
Klobassa, D. S.	2014	The burden of pneumococcal meningitis in Austrian children between 2001 and 2008	Gestational age of study population was not sufficiently stated.
Knight, K. M.	2020	The mitochondrial DNA variant m.9032T¬†>¬†C in MT-ATP6 encoding p.(Leu169Pro) causes a complex mitochondrial neurological syndrome	Gestational age of study population was not sufficiently stated.
Knight, K. R.	2017	Group-Wide, Prospective Study of Ototoxicity Assessment in Children Receiving Cisplatin Chemotherapy (ACCL05C1): A Report From the Children's Oncology Group	Gestational age of study population was not sufficiently stated.
Knott, P. D.	2001	Sensorineural hearing loss and Kawasaki disease: A prospective study	Gestational age of study population was not sufficiently stated.
Kobas, M.	2018	Clinical characteristics, audiological and neurodevelopmental outcomes of newborns with congenital cytomegalovirus infection	No comparison of hearing impairment between preterm and full-term born infants.
Kobbernagel, H. E.	2016	Study protocol, rationale and recruitment in a European multi-centre randomized controlled trial to determine the efficacy and safety of azithromycin maintenance therapy for 6 months in primary ciliary dyskinesia	Study protocol as unsuitable study type.
Kocon, S.	2013	[Analysis of factors affecting the abnormal otoacoustic emissions in infants]	No comparison of hearing impairment between preterm and full-term born infants.
Kocyigit, M.	2020	An Investigation of Hearing (250-20,000 Hz) in Children with Endocrine Diseases and Evaluation of Tinnitus and Vertigo Symptoms	Gestational age of study population was not sufficiently stated.
Kodama, Y.	2009	Intrapartum fetal heart rate patterns in infants (> or =34 weeks) with poor neurological outcome	No comparison of hearing impairment between preterm and full-term born infants.

Kodiya, A. M.	2012	The burden of hearing loss in Kaduna, Nigeria: a 4-year study at the National Ear Care Centre	Gestational age of study population was not sufficiently stated.
Koehne, P. S.	2006	Genetic deafness in a preterm infant with a critical postnatal course	Case report as unsuitable study type.
Koga, K.	1988	Immunological study on association between mumps and infantile unilateral deafness	Gestational age of study population was not sufficiently stated.
Kohlberg, G. D.	2018	Adolescent Obesity Is an Independent Risk Factor for Sensorineural Hearing Loss: Results From the National Health and Nutrition Examination Survey 2005 to 2010	Gestational age of study population was not sufficiently stated.
Koitschev, A.	2012	Progressive familial hearing loss in Muckle-Wells syndrome	Gestational age of study population was not sufficiently stated.
Kojundzic, S. L.	2021	The applicability of magnetic resonance imaging classification system (MRICS) for cerebral palsy and its association with perinatal factors and related disabilities in a Croatian population-based sample	Connection of risk factors and/or hearing outcome depending on gestational age was not established.
Kokotas, H.	2011	Detection of deafness-causing mutations in the Greek mitochondrial genome	Gestational age of study population was not sufficiently stated.
Kolossa-Gehring, M.	2007	German Environmental Survey for children (GerES IV) - First results	Gestational age of study population was not sufficiently stated.
Kolski, H., Ford-Jones, E. L., Richardson, S., Petric, M., Nelson, S., Jamieson, F., Blaser, S., Gold, R., Otsubo, H., Heurter, H. and MacGregor, D.	1998	Etiology of acute childhood encephalitis at The Hospital for Sick Children, Toronto, 1994-1995	Gestational age of study population was not sufficiently stated.
Komazec, Z.	2007	[Cochlear implantation at the Ear, Nose and Throat Clinic of the Clinical Center of Vojvodina]	No comparison of hearing impairment between preterm and full-term born infants.
Komune, S.	1986	Recurrent meningitis due to spontaneous cerebrospinal fluid otorrhea. A case report	Case report as unsuitable study type.
Konopka, W.	2021	Cytomegalovirus infections in pregnant women as a risk of congenital deafness in a child	Gestational age of study population was not sufficiently stated.
Konukseven, O.	2017	Regional differences of Turkey in risk factors of newborn hearing loss	Connection of risk factors and/or hearing outcome depending on gestational age was not established.
Koomen, I.	2003	Parental perception of educational, behavioural and general health problems in school-age survivors of bacterial meningitis	Gestational age of study population was not sufficiently stated.
Koomen, I.	2003	Hearing loss at school age in survivors of bacterial meningitis: Assessment, incidence, and prediction	Gestational age of study population was not sufficiently stated.
Koomen, I.	2005	Academic and behavioral limitations and health-related quality of life in school-age survivors of bacterial meningitis	Gestational age of study population was not sufficiently stated.
Koparir, A.	2015	Whole-exome sequencing revealed two novel mutations in Usher syndrome	Gestational age of study population was not sufficiently stated.
Kopelovich, J. C.	2011	Early prediction of postmeningitic hearing loss in children using magnetic resonance imaging	Gestational age of study population was not sufficiently stated.
Koppelhus, U.	2011	A novel mutation in the connexin 26 gene (GJB2) in a child with clinical and histological features of keratitis-ichthyosis-deafness (KID) syndrome	Case report as unsuitable study type.
Korkmaz, H. A.	2019	A case of immune-mediated type 1 diabetes mellitus due to congenital rubella infection	Case report as unsuitable study type.
Korndewal, M. J.	2017	Long-term impairment attributable to congenital cytomegalovirus infection: a retrospective cohort study	Connection of risk factors and/or hearing outcome depending on gestational age was not established.
Korndewal, M. J.	2016	Disease burden of congenital cytomegalovirus infection at school entry age: study design, participation rate and birth prevalence	Gestational age of study population was not sufficiently stated.
Kornelisse, R. F.	1995	Pneumococcal meningitis in children: Prognostic indicators and outcome	Gestational age of study population was not sufficiently stated.
Korres, S.	2005	Newborn hearing screening: Effectiveness, importance of high-risk factors, and characteristics of infants in the neonatal intensive care unit and well-baby nursery	No comparison of hearing impairment between preterm and full-term born infants.
Korres, S.	2007	Influence of smoking on developing cochlea. Does smoking during pregnancy affect the amplitudes of transient evoked otoacoustic emissions in newborns?	Gestational age of study population was not sufficiently stated.
Korres, S. G.	2007	The effect of very low birth weight on otoacoustic emissions	Gestational age of study population does not meet defined inclusion criteria for prematurity (<37 weeks) and/or full-term birth (≥37 weeks) with cut-off stated at 37 weeks.
Korver, A. M.	2011	Causes of permanent childhood hearing impairment	Gestational age of study population was not sufficiently stated.

Korver, A. M. H.	2009	DECIBEL study: Congenital cytomegalovirus infection in young children with permanent bilateral hearing impairment in the Netherlands	Connection of risk factors and/or hearing outcome depending on gestational age was not established.
Kos, M. I.	2004	Anatomic and functional long-term results of canal wall-down mastoidectomy	Gestational age of study population was not sufficiently stated.
Kose, E.	2014	Val2Ala mutation in the Atp6v0a4 gene causes early-onset sensorineural hearing loss in children with recessive distal renal tubular acidosis: a case report	Case report as unsuitable study type.
Koskiniemi, M.	1978	Haemophilus influenzae meningitis. A comparison between chloramphenicol and ampicillin therapy with special reference to impaired hearing	Gestational age of study population was not sufficiently stated.
Kosmidou, P.	2022	Hearing Outcomes of Infants Born to Mothers With Active COVID-19 Infection	Connection of risk factors and/or hearing outcome depending on gestational age was not established.
Kosmidou, P.	2021	Newborn Hearing Screening: Analysing the Effectiveness of Early Detection of Neonatal Hearing Loss in a Hospital in Greece	Only investigation of prematurity as risk factor for hearing impairment, other risk factors not analyzed comparing preterm and full-term born infants.
Kotagal, S.	1981	Auditory evoked potentials in bacterial meningitis	Gestational age of study population was not sufficiently stated.
Kountakis, S. E.	1997	Risk factors associated with hearing loss in neonates	Only investigation of prematurity as risk factor for hearing impairment, other risk factors not analyzed comparing preterm and full-term born infants.
Kountakis, S. E.	2002	Risk factors for hearing loss in neonates: A prospective study	Gestational age of study population was not sufficiently stated.
Koyano, S.	2018	Congenital cytomegalovirus in Japan: More than 2 year follow up of infected newborns	No comparison of hearing impairment between preterm and full-term born infants.
Kraft, C. T.	2014	Risk Indicators for Congenital and Delayed-Onset Hearing Loss	Gestational age of study population was not sufficiently stated.
Kramer, S. J.	1989	Auditory brainstem responses and clinical follow-up of high-risk infants	Gestational age of study population was not sufficiently stated.
Kreicher, K. L.	2018	Audiometric assessment of pediatric patients with cystic fibrosis	Gestational age of study population was not sufficiently stated.
Kreicher, K. L.	2018	Characteristics and Progression of Hearing Loss in Children with Down Syndrome	Gestational age of study population was not sufficiently stated.
Kremer, H.	1996	Localization of the gene (or genes) for a syndrome with X-linked mental retardation, ataxia, weakness, hearing impairment, loss of vision and a fatal course in early childhood	Gestational age of study population was not sufficiently stated.
Kripps, K. A.	2020	A novel acceptor stem variant in mitochondrial tRNA(Tyr) impairs mitochondrial translation and is associated with a severe phenotype	Case report as unsuitable study type.
Kritzinger, A.	2006	Communication development of a young child with foetal retinoid syndrome: a seven-year follow-up study	Gestational age of study population was not sufficiently stated.
Kroes, H. Y.	2010	Is hearing loss a feature of Joubert syndrome, a ciliopathy?	Gestational age of study population was not sufficiently stated.
Krueger, A.	2017	Relationship of the Middle Ear Effusion Microbiome to Secretory Mucin Production in Pediatric Patients With Chronic Otitis Media	Gestational age of study population was not sufficiently stated.
Krzyzak, A.	2018	Paediatric otitis media with effusion is connected to deficits in music perception	Gestational age of study population was not sufficiently stated.
Kubba, H.	2004	Is deafness a disease of poverty? The association between socio-economic deprivation and congenital hearing impairment	Gestational age of study population was not sufficiently stated.
Kuemmerle-Deschner, J. B.	2015	Early detection of sensorineural hearing loss in Muckle-Wells-syndrome	Gestational age of study population was not sufficiently stated.
Kuemmerle-Deschner, J. B.	2013	Hearing loss in Muckle-Wells syndrome	Gestational age of study population was not sufficiently stated.
Külahli, I.	1997	Evaluation of hearing loss with auditory brainstem responses in the early and late period of bacterial meningitis in children	Gestational age of study population was not sufficiently stated.
Kumar, A.	2017	Universal Hearing Screening in Newborns Using Otoacoustic Emissions and Brainstem Evoked Response in Eastern Uttar Pradesh	Gestational age of study population was not sufficiently stated.
Kumar, A.	2020	Study on Etiological Factors, Clinical Pattern and Comorbidities in Cerebral Palsy Children of North Bihar Region	Connection of risk factors and/or hearing outcome depending on gestational age was not established.
Kumar, A.	2015	Hearing screening in a tertiary care hospital in India	Only investigation of prematurity as risk factor for hearing impairment, other risk factors not analyzed comparing preterm and full-term born infants.
Kumar, M. L.	1984	Congenital and postnatally acquired cytomegalovirus infections: long-term follow-up	Gestational age of study population was not sufficiently stated.

Kummerle-Deschner, J. B.	2010	Risk Factors for Severe Muckle-Wells Syndrome	Gestational age of study population was not sufficiently stated.
Kumor, K. M.	1984	Effect of 'high-dose' amikacin in children	Gestational age of study population was not sufficiently stated.
Kunstmann, E.	2005	Congenital hearing loss. Mutation analysis of connexin genes and genetic counselling	Gestational age of study population was not sufficiently stated.
Kurten, T.	2008	Frequency of hearing disorders in children with langerhans' cell histiocytosis	Gestational age of study population was not sufficiently stated.
Kusama, Y.	2022	Rapid progressive destruction of the cochleae in an infant due to pneumococcal meningitis	Case report as unsuitable study type.
Kuschke, S.	2020	Profile of childhood hearing loss in the Western Cape, South Africa	Gestational age of study population was not sufficiently stated.
Kushner, B.	2016	Frequency and Demographics of Gentamicin Use	Gestational age of study population was not sufficiently stated.
Kutz, J. W.	2006	Clinical predictors for hearing loss in children with bacterial meningitis	Gestational age of study population was not sufficiently stated.
Kuzniewicz, M. W.	2014	Incidence, Etiology, and Outcomes of Hazardous Hyperbilirubinemia in Newborns	No comparison of hearing impairment between preterm and full-term born infants.
Kvestad, E.	2014	Sensorineural hearing loss in children: The association with Apgar score. A registry-based study of 392 371 children in Norway	Connection of risk factors and/or hearing outcome depending on gestational age was not established.
Kwak, M.	2018	Brain Magnetic Resonance Imaging Findings of Congenital Cytomegalovirus Infection as a Prognostic Factor for Neurological Outcome	Gestational age of study population was not sufficiently stated.
Kylat, R. I.	2006	Clinical findings and adverse outcome in neonates with symptomatic congenital cytomegalovirus (SCCMV) infection	Connection of risk factors and/or hearing outcome depending on gestational age was not established.
La Mantia, I.	2018	Effects of salso-bromo-iodine thermal water in children suffering from otitis media with effusion: a randomized controlled pilot study	Gestational age of study population was not sufficiently stated.
Laakkonen, H.	2011	Neurological development in 21 children on peritoneal dialysis in infancy	No comparison of hearing impairment between preterm and full-term born infants.
Laakso, J. T.	2021	Severe acute otitis media and mastoiditis caused by group A beta-hemolytic streptococcus	Gestational age of study population was not sufficiently stated.
Labaeka, A. A.	2018	Prevalence of Hearing Impairment Among High-Risk Newborns in Ibadan, Nigeria	Only investigation of prematurity as risk factor for hearing impairment, other risk factors not analyzed comparing preterm and full-term born infants.
LaBrecque, D. R.	1982	Four generations of arteriohepatic dysplasia	Gestational age of study population was not sufficiently stated.
Lachowska, M.	2014	Second stage of Universal Neonatal Hearing Screening - A way for diagnosis and beginning of proper treatment for infants with hearing loss	Only investigation of prematurity as risk factor for hearing impairment, other risk factors not analyzed comparing preterm and full-term born infants.
Lackner, A.	2009	Effect on hearing of ganciclovir therapy for asymptomatic congenital cytomegalovirus infection: four to 10 year follow up	Gestational age of study population was not sufficiently stated.
Lafay-Cousin, L.	2013	Early cisplatin induced ototoxicity profile may predict the need for hearing support in children with medulloblastoma	Gestational age of study population was not sufficiently stated.
Lafreniere, D.	1993	Otoacoustic emissions in full-term newborns at risk for hearing loss	Case report as unsuitable study type.
Lage, M. L. C.	2019	Clinical, Neuroimaging, and Neurophysiological Findings in Children with Microcephaly Related to Congenital Zika Virus Infection	Connection of risk factors and/or hearing outcome depending on gestational age was not established.
Lalaiants, M. R.	2014	[The audiological phenotype and the prevalence of GJB2-related sensorineural loss of hearing in the infants suffering acoustic disturbances]	No comparison of hearing impairment between preterm and full-term born infants.
Lalayants, M. R.	2020	[OTOF-related auditory neuropathy spectrum disorder]	No comparison of hearing impairment between preterm and full-term born infants.
Lalwani, A. K.	2009	Predictability of cochlear implant outcome in families	Gestational age of study population was not sufficiently stated.
Lalwani, A. K.	2013	Obesity is Associated With Sensorineural Hearing Loss in Adolescents	Gestational age of study population was not sufficiently stated.
Lalwani, A. K.	2011	Secondhand smoke and sensorineural hearing loss in adolescents	Gestational age of study population was not sufficiently stated.
Lammens, F.	2013	Syndromic disorders in congenital hearing loss	Gestational age of study population was not sufficiently stated.
Lammens, F.	2013	Aetiology of congenital hearing loss: A cohort review of 569 subjects	Gestational age of study population was not sufficiently stated.

Lanari, M.	2006	Neonatal cytomegalovirus blood load and risk of sequelae in symptomatic and asymptomatic congenitally infected newborns	Gestational age of study population was not sufficiently stated.
Landier, W.	2012	Yield of Screening for Long-Term Complications Using the Children's Oncology Group Long-Term Follow-Up Guidelines	Gestational age of study population was not sufficiently stated.
Landier, W.	2014	Ototoxicity in children with high-risk neuroblastoma: prevalence, risk factors, and concordance of grading scales--a report from the Children's Oncology Group	Gestational age of study population was not sufficiently stated.
Lane, W.	1994	X-linked recessive nephritis with mental retardation, sensorineural hearing loss, and macrocephaly	Case report as unsuitable study type.
Lang-Roth, R.	2017	AUNA2: A Novel Type of Non-Syndromic Slowly Progressive Auditory Synaptopathy/Auditory Neuropathy with Autosomal-Dominant Inheritance	Gestational age of study population was not sufficiently stated.
Langer, T.	2020	Usefulness of current candidate genetic markers to identify childhood cancer patients at risk for platinum-induced ototoxicity: Results of the European PanCareLIFE cohort study	Gestational age of study population was not sufficiently stated.
Lannering, B.	2012	Hyperfractionated Versus Conventional Radiotherapy Followed by Chemotherapy in Standard-Risk Medulloblastoma: Results From the Randomized Multicenter HIT-SIOP PNET 4 Trial	Gestational age of study population was not sufficiently stated.
Lantos, P. M.	2018	Geographic and Racial Disparities in Infant Hearing Loss	Connection of risk factors and/or hearing outcome depending on gestational age was not established.
Lanvers-Kaminsky, C.	2015	Human OCT2 variant c.808G > T confers protection effect against cisplatin-induced ototoxicity	Gestational age of study population was not sufficiently stated.
Lanzieri, T. M.	2022	Progressive, Long-Term Hearing Loss in Congenital CMV Disease After Ganciclovir Therapy	Connection of risk factors and/or hearing outcome depending on gestational age was not established.
Lanzieri, T. M.	2017	Hearing Loss in Children With Asymptomatic Congenital Cytomegalovirus Infection	Connection of risk factors and/or hearing outcome depending on gestational age was not established.
Lanzieri, T. M.	2018	Hearing Trajectory in Children with Congenital Cytomegalovirus Infection	Gestational age of study population was not sufficiently stated.
Lapointe, J. R.	1984	A comparison of ampicillin-cefotaxime and ampicillin-chloramphenicol in childhood bacterial meningitis: an experience in 55 patients	Gestational age of study population was not sufficiently stated.
Lasek-Duriez, A.	2008	[Chronic exanthema in a child with congenital rubella]	Case report as unsuitable study type.
Lasisi, A. O.	2008	Early onset otitis media: risk factors and effects on the outcome of chronic suppurative otitis media	Gestational age of study population was not sufficiently stated.
Lasisi, A. O.	2014	Neonatal hearing screening in a rural/sub-urban community in Nigeria, sub-Saharan Africa-A preliminary report	Only investigation of prematurity as risk factor for hearing impairment, other risk factors not analyzed comparing preterm and full-term born infants.
Lasisi, A. O.	2007	Socio-economic status and hearing loss in chronic suppurative otitis media in Nigeria	Gestational age of study population was not sufficiently stated.
Lasisi, O. A.	2006	Challenges in management of childhood sensorineural hearing loss in sub-Saharan Africa, Nigeria	Gestational age of study population was not sufficiently stated.
Latoch, E.	2022	Late effects of childhood cancer treatment in long-term survivors diagnosed before the age of 3 years - A multicenter, nationwide study	Gestational age of study population was not sufficiently stated.
Lau, W. K.	1977	Amikacin therapy of exacerbations of Pseudomonas aeruginosa infections in patients with cystic fibrosis	Gestational age of study population was not sufficiently stated.
Lauffer, H.	1994	[Click-evoked otoacoustic emissions and acoustic brain stem potentials in early detection of hearing disorders in premature and newborn infants after neonatal critical care]	Connection of risk factors and/or hearing outcome depending on gestational age was not established.
Laurens, M. B.	2008	MRSA with progression from otitis media and sphenoid sinusitis to clival osteomyelitis, pachymeningitis and abducens nerve palsy in an immunocompetent 10-year-old patient	Case report as unsuitable study type.
Laurent, S.	2021	Molecular characterization of pathogenic OTOA gene conversions in hearing loss patients	Gestational age of study population was not sufficiently stated.
Lawrence, J.	2022	Short-course intravenous antibiotics for young infants with urinary tract infection	Connection of risk factors and/or hearing outcome depending on gestational age was not established.
Lazar, A.	2021	Identical twins affected by congenital cytomegalovirus infections showed different audio-vestibular profiles	Connection of risk factors and/or hearing outcome depending on gestational age was not established.
Lazar, C.	2010	Prevalence of the c.35delG and p.W24X mutations in the GJB2 gene in patients with nonsyndromic hearing loss from North-West Romania	Gestational age of study population was not sufficiently stated.
le Clercq, C. M. P.	2018	Association Between Portable Music Player Use and Hearing Loss Among Children of School Age in the Netherlands	Gestational age of study population was not sufficiently stated.

le Clercq, C. M. P.	2017	Prevalence of Hearing Loss Among Children 9 to 11 Years Old: The Generation R Study	Gestational age of study population was not sufficiently stated.
Le Roux, T.	2015	Profound childhood hearing loss in a South Africa cohort: Risk profile, diagnosis and age of intervention	Only investigation of prematurity as risk factor for hearing impairment, other risk factors not analyzed comparing preterm and full-term born infants.
le Roux, T.	2016	Predictors of pediatric cochlear implantation outcomes in South Africa	Only investigation of prematurity as risk factor for hearing impairment, other risk factors not analyzed comparing preterm and full-term born infants.
Leach, A.	2008	Topical ciprofloxin versus topical framycetin-gramicidin-dexamethasone in Australian aboriginal children with recently treated chronic suppurative otitis media: a randomized controlled trial	Gestational age of study population was not sufficiently stated.
Leach, A. J.	2022	Immunogenicity, otitis media, hearing impairment, and nasopharyngeal carriage 6-months after 13-valent or ten-valent booster pneumococcal conjugate vaccines, stratified by mixed priming schedules: PREVIX_COMBO and PREVIX_BOOST randomised controlled trials	Connection of risk factors and/or hearing outcome depending on gestational age was not established.
Lebel, M. H.	1989	Delayed cerebrospinal fluid sterilization and adverse outcome of bacterial meningitis in infants and children	Gestational age of study population was not sufficiently stated.
Lebel, M. H.	1988	Dexamethasone therapy for bacterial meningitis. Results of two double-blind, placebo-controlled trials	Gestational age of study population was not sufficiently stated.
Lebel, M. H.	1989	Comparative efficacy of ceftriaxone and cefuroxime for treatment of bacterial meningitis	Gestational age of study population was not sufficiently stated.
Lebel, M. H.	1989	Magnetic resonance imaging and dexamethasone therapy for bacterial meningitis	Gestational age of study population was not sufficiently stated.
Lee, B. H.	2008	Neurodevelopmental outcomes of extremely low birth weight infants exposed prenatally to dexamethasone versus betamethasone	No comparison of hearing impairment between preterm and full-term born infants.
Lee, C. L.	2021	Otorhinolaryngological Management in Taiwanese Patients with Mucopolysaccharidoses	Gestational age of study population was not sufficiently stated.
Lee, C. Y.	2022	Comprehensive Etiologic Analyses in Pediatric Cochlear Implantees and the Clinical Implications	Gestational age of study population was not sufficiently stated.
Lee, D. J.	1997	Sociodemographic and educational correlates of hearing loss in Hispanic children	Gestational age of study population was not sufficiently stated.
Lee, D. K.	2006	Salivary gland choristoma of the middle ear in an infant: a case report	Case report as unsuitable study type.
Lee, E. R.	2019	Implications of dried blood spot testing for congenital CMV on management of children with hearing loss: A preliminary report	Gestational age of study population was not sufficiently stated.
Lee, H. S.	1997	[A study on fall accident]	No comparison of hearing impairment between preterm and full-term born infants.
Lee, H. Y.	2015	Acute-Onset Tinnitus Is Associated with Contralateral Hearing in Sudden Deafness	Gestational age of study population was not sufficiently stated.
Lee, J.	2021	Uncommon clinical presentation of a common bug: Group A Streptococcus meningitis	Case report as unsuitable study type.
Lee, J. A.	2020	Factors affecting complications and comorbidities in children with cholesteatoma	Gestational age of study population was not sufficiently stated.
Lee, J. A.	2020	Hearing outcomes in children of diabetic pregnancies	Gestational age of study population was not sufficiently stated.
Lee, J. S.	2017	The neutrophil-to-lymphocyte ratio in children with sudden sensorineural hearing loss: a retrospective study	Gestational age of study population was not sufficiently stated.
Lee, Y. K.	2009	The significance of measurement of serum unbound bilirubin concentrations in high-risk infants	Connection of risk factors and/or hearing outcome depending on gestational age was not established.
Legault, G.	2011	Predicting comorbidities with neuroimaging in children with cerebral palsy	Gestational age of study population was not sufficiently stated.
Legood, R.	2009	Health related quality of life in survivors of pneumococcal meningitis	Gestational age of study population was not sufficiently stated.
Lehmann, D.	2008	The kalgoorlie otitis media research project: rationale, methods, population characteristics and ethical considerations	Gestational age of study population was not sufficiently stated.
Lehmann, D.	2008	Absent otoacoustic emissions predict otitis media in young Aboriginal children: a birth cohort study in Aboriginal and non-Aboriginal children in an arid zone of Western Australia	Gestational age of study population was not sufficiently stated.
Leibu, S.	2017	Clinical Significance of Long-Term Follow-Up of Children with Posttraumatic Skull Base Fracture	Gestational age of study population was not sufficiently stated.

Leite, J. N.	2016	Otoacoustic emissions in newborns with mild and moderate perinatal hypoxia	No comparison of hearing impairment between preterm and full-term born infants.
Leite, R. F. P.	2018	Hearing Screening in children with Congenital Zika Virus Syndrome in Fortaleza, Cear√°, Brazil, 2016	No comparison of hearing impairment between preterm and full-term born infants.
Leite, S. S.	2021	Neurodevelopmental outcomes of children with periventricular leukomalacia: the role of infection and ischemia	No comparison of hearing impairment between preterm and full-term born infants.
Lemajic-Komazec, S.	2007	[The role of current audiological tests in the early diagnosis of hearing impairment in children]	Connection of risk factors and/or hearing outcome depending on gestational age was not established.
Lempinen, L.	2022	Hearing impairment in Angolan children with acute bacterial meningitis with and without otitis media	Gestational age of study population was not sufficiently stated.
Leppert, D.	2000	Matrix metalloproteinase (MMP)-8 and MMP-9 in cerebrospinal fluid during bacterial meningitis: Association with blood-brain barrier damage and neurological sequelae	Gestational age of study population was not sufficiently stated.
Leruez-Ville, M.	2020	Quantifying the Burden of Congenital Cytomegalovirus Infection With Long-term Sequelae in Subsequent Pregnancies of Women Seronegative at Their First Pregnancy	Gestational age of study population was not sufficiently stated.
Leruez-Ville, M.	2021	Accuracy of prenatal ultrasound screening to identify fetuses infected by cytomegalovirus which will develop severe long-term sequelae	Connection of risk factors and/or hearing outcome depending on gestational age was not established.
Leruez-Ville, M.	2009	Retrospective diagnosis of congenital CMV infection in DBS from Guthrie cards: French experience	Gestational age of study population was not sufficiently stated.
Lesinski-Schiedat, A.	1999	[Temporal bone fracture after head trauma causing rhinoliquorrhea and meningitis]	Gestational age of study population was not sufficiently stated.
Leslie, G. I.	1995	Risk factors for sensorineural hearing loss in extremely premature infants	No comparison of hearing impairment between preterm and full-term born infants.
Lesperance, M. M.	2003	Mutations in the Wolfram syndrome type 1 gene (WFS1) define a clinical entity of dominant low-frequency sensorineural hearing loss	Gestational age of study population was not sufficiently stated.
Letouzey, M.	2017	Severe apparently isolated fetal ventriculomegaly and neurodevelopmental outcome	Hearing outcome not examined separately.
Leung, J.	2018	Valganciclovir Use Among Commercially and Medicaid-insured Infants With Congenital CMV Infection in the United States, 2009-2015	Gestational age of study population was not sufficiently stated.
Leung, J. C.	2016	Antenatal factors modulate hearing screen failure risk in preterm infants	No comparison of hearing impairment between preterm and full-term born infants.
Leung, W.	2000	Late effects in survivors of infant leukemia	Gestational age of study population was not sufficiently stated.
Levenson, M. J.	1989	The large vestibular aqueduct syndrome in children. A review of 12 cases and the description of a new clinical entity	Gestational age of study population was not sufficiently stated.
Levi, J.	2019	Cofactors of Pediatric Tinnitus: A Look at the Whole Picture	Gestational age of study population was not sufficiently stated.
Levy, M.	1991	Pregnancy outcome following first trimester exposure to chloroquine	Gestational age of study population was not sufficiently stated.
Lewis, M. J.	2009	Ototoxicity in children treated for osteosarcoma	Gestational age of study population was not sufficiently stated.
Leyder, M.	2016	Primary maternal cytomegalovirus infections: accuracy of fetal ultrasound for predicting sequelae in offspring	Gestational age of study population was not sufficiently stated.
Li, F. J.	2016	Clinical Study on 136 Children with Sudden Sensorineural Hearing Loss	Gestational age of study population was not sufficiently stated.
Li, L. Q.	2013	Does congenital cytomegalovirus infection lead to hearing loss by inducing mutation of the GJB2 gene?	Connection of risk factors and/or hearing outcome depending on gestational age was not established.
Li, T. C.	2015	Association between mutations in the gap junction beta 4 gene and nonsyndromic hearing loss: Genotype-phenotype correlation patterns	Gestational age of study population was not sufficiently stated.
Li, X.	2017	Oral exposure to arsenic causes hearing loss in young people aged 12-29 years and in young mice	Gestational age of study population was not sufficiently stated.
Li, Y.	2004	Predicting cisplatin ototoxicity in children: the influence of age and the cumulative dose	Gestational age of study population was not sufficiently stated.
Li, Y.	2017	Serious Adverse Reactions From Anti-tuberculosis Drugs Among 599 Children Hospitalized for Tuberculosis	Gestational age of study population was not sufficiently stated.
Liang, C.	2013	The effects and outcomes of electrolyte disturbances and asphyxia on newborns hearing	Gestational age of study population does not meet defined inclusion criteria for prematurity (<37 weeks) and/or full-term birth (≥37 weeks) with cut-off stated at 37 weeks.

Libster, R.	2012	Long-term outcomes of group B streptococcal meningitis	No comparison of hearing impairment between preterm and full-term born infants.
Lichtig, I.	1997	[Auditory behavior monitoring after bacterial meningitis. Case report]	Case report as unsuitable study type.
Lichtig, I.	2001	[Assessment of auditory behaviour and neuropsychomotor development of low weight infants]	No comparison of hearing impairment between preterm and full-term born infants.
Liddle, K.	2022	Aetiology of permanent childhood hearing loss at a population level	No comparison of hearing impairment between preterm and full-term born infants.
Liddle, K.	2022	Cochlear nerve deficiency is an important cause of auditory neuropathy spectrum disorder at a population level in children	No comparison of hearing impairment between preterm and full-term born infants.
Lien, T. H.	2011	Recurrent bacterial meningitis associated with Mondini dysplasia	Case report as unsuitable study type.
Lieu, J. E.	2006	Prediction of auditory brainstem reflex screening referrals in high-risk infants	Only investigation of prematurity as risk factor for hearing impairment, other risk factors not analyzed comparing preterm and full-term born infants.
Lieu, J. E. C.	2013	Evaluating a Prediction Model for Infant Hearing Loss	Only investigation of prematurity as risk factor for hearing impairment, other risk factors not analyzed comparing preterm and full-term born infants.
Lilly, E.	2019	More than keratitis, ichthyosis, and deafness: Multisystem effects of lethal GJB2 mutations	Connection of risk factors and/or hearing outcome depending on gestational age was not established.
Lim, B. G.	2013	Utility of Genetic Testing for the Detection of Late-Onset Hearing Loss in Neonates	No comparison of hearing impairment between preterm and full-term born infants.
Lim, D.	2020	Prevalence, risk factors and management strategies for otological problems in girls with Turner syndrome	Gestational age of study population was not sufficiently stated.
Lim, J.	2021	Outcomes of infants born to pregnant women with syphilis: a nationwide study in Korea	No comparison of hearing impairment between preterm and full-term born infants.
Lima, A. F.	2020	Is pediatric cholesteatoma more aggressive in children than in adults? A comparative study using the EAONO/JOS classification	Gestational age of study population was not sufficiently stated.
Lima, G. M.	2006	Hearing screening in a neonatal intensive care unit	Only investigation of prematurity as risk factor for hearing impairment, other risk factors not analyzed comparing preterm and full-term born infants.
Limberger, A.	2007	[Hearing loss in patients with Fabry disease]	Gestational age of study population was not sufficiently stated.
Lin, C.	2013	Risk of sudden sensorineural hearing loss in patients with systemic lupus erythematosus: a population-based cohort study	Gestational age of study population was not sufficiently stated.
Lin, C.	2020	Diagnosis and medical care for congenital cytomegalovirus infection An observational study using claims data in Japan, 2010 to 2017	Gestational age of study population was not sufficiently stated.
Lin, C., Lin, S. W., Weng, S. F. and Lin, Y. S.	2014	Risk of developing sudden sensorineural hearing loss in patients with nasopharyngeal carcinoma: a population-based cohort study	Gestational age of study population was not sufficiently stated.
Lin, J. L.	2015	Immunologic assessment and KMT2D mutation detection in Kabuki syndrome	Gestational age of study population was not sufficiently stated.
Lin, M.	2020	A novel c.287G>T NDP missense mutation in a Chinese family with Norrie disease	Gestational age of study population was not sufficiently stated.
Lin, M. C.	2012	Factors for poor prognosis of neonatal bacterial meningitis in a medical center in Northern Taiwan	Connection of risk factors and/or hearing outcome depending on gestational age was not established.
Lin, P. C.	2004	Characteristics of nosocomial bacterial meningitis in children	Connection of risk factors and/or hearing outcome depending on gestational age was not established.
Lin, P. H.	2017	Etiologic and Audiologic Characteristics of Patients With Pediatric-Onset Unilateral and Asymmetric Sensorineural Hearing Loss	Gestational age of study population was not sufficiently stated.
Lin, P. H.	2020	An integrative approach for pediatric auditory neuropathy spectrum disorders: revisiting etiologies and exploring the prognostic utility of auditory steady-state response	Only investigation of prematurity as risk factor for hearing impairment, other risk factors not analyzed comparing preterm and full-term born infants.
Lin, S. J.	2022	Biallelic variants in WARS1 cause a highly variable neurodevelopmental syndrome and implicate a critical exon for normal auditory function	Case report as unsuitable study type.
Lindenburg, I. T.	2012	Long-term neurodevelopmental outcome after intrauterine transfusion for hemolytic disease of the fetus/newborn: the LOTUS study	Connection of risk factors and/or hearing outcome depending on gestational age was not established.
Lindstrom, K.	2006	Teenage outcome after being born at term with moderate neonatal encephalopathy	No comparison of hearing impairment between preterm and full-term born infants.
Lipinski, P.	2022	Long-term outcome of patients with alpha-mannosidosis-A single center study	Gestational age of study population was not sufficiently stated.

Lipitz, S.	2010	Value of prenatal ultrasound and magnetic resonance imaging in assessment of congenital primary cytomegalovirus infection	Hearing test in preterm infants not accipable.
Lipitz, S.	2020	Revisiting short- and long-term outcome after fetal first-trimester primary cytomegalovirus infection in relation to prenatal imaging findings	Gestational age of study population was not sufficiently stated.
Lipitz, S.	2013	Risk of cytomegalovirus-associated sequelae in relation to time of infection and findings on prenatal imaging	No comparison of hearing impairment between preterm and full-term born infants.
Liskova, P.	2013	Novel OPA1 missense mutation in a family with optic atrophy and severe widespread neurological disorder	Gestational age of study population was not sufficiently stated.
Liu, A.	2017	Clinical, pathological, and genetic evaluations of Chinese patient with otodental syndrome and multiple complex odontoma: Case report	Case report as unsuitable study type.
Liu, A. P. Y.	2019	Treatment burden and long-term health deficits of patients with low-grade gliomas or glioneuronal tumors diagnosed during the first year of life	Gestational age of study population was not sufficiently stated.
Liu, C.	2019	Development of a community-based hearing loss prevention and control service model in Guangdong, China	Gestational age of study population was not sufficiently stated.
Liu, X. Y.	2001	Neurologic complications due to catheterization	Gestational age of study population was not sufficiently stated.
Liu, X. Z.	2001	Epidemiological studies on hearing impairment with preference to genetic factors in Sichuan, China	Gestational age of study population was not sufficiently stated.
Liu, Y.	2018	Hearing loss in children with e-waste lead and cadmium exposure	Gestational age of study population was not sufficiently stated.
Liu, Z.	2013	Hearing screening and diagnosis in a large sample of infants in Central China	Gestational age of study population was not sufficiently stated.
Livingstone, N.	2011	Motor skill deficits in children with partial hearing	Gestational age of study population was not sufficiently stated.
Llanes, E. G.	2004	Evoked otoacoustic emissions and auditory brainstem responses: concordance in hearing screening among high-risk children	Only investigation of prematurity as risk factor for hearing impairment, other risk factors not analyzed comparing preterm and full-term born infants.
Lo, T. H.	2022	Prognostic determinants of hearing outcomes in children with congenital cytomegalovirus infection	Gestational age of study population was not sufficiently stated.
Lobenius-Palmer, K.	2018	Accelerometer-Assessed Physical Activity and Sedentary Time in Youth With Disabilities	Gestational age of study population was not sufficiently stated.
Lodha, A.	2020	Caesarean section and neonatal survival and neurodevelopmental impairments in preterm singleton neonates	No comparison of hearing impairment between preterm and full-term born infants.
Lodha, A.	2019	Early Caffeine Administration and Neurodevelopmental Outcomes in Preterm Infants	No comparison of hearing impairment between preterm and full-term born infants.
Lodha, A.	2018	Does duration of caffeine therapy in preterm infants born <= 1250 g at birth influence neurodevelopmental (ND) outcomes at 3 years of age?	No comparison of hearing impairment between preterm and full-term born infants.
Lofkvist, U.	2020	Executive Functions, Pragmatic Skills, and Mental Health in Children With Congenital Cytomegalovirus (CMV) Infection With Cochlear Implants: A Pilot Study	Gestational age of study population was not sufficiently stated.
Lok, W.	2012	Risk factors for failing the hearing screen due to otitis media in Dutch infants	Only investigation of prematurity as risk factor for hearing impairment, other risk factors not analyzed comparing preterm and full-term born infants.
Lombardi, G.	2009	Oral valganciclovir treatment in newborns with symptomatic congenital cytomegalovirus infection	No comparison of hearing impairment between preterm and full-term born infants.
Longnecker, M. P.	2004	In utero exposure to polychlorinated biphenyls and sensorineural hearing loss in 8-year-old children	Gestational age of study population was not sufficiently stated.
Lopez, A. S.	2017	Intelligence and Academic Achievement With Asymptomatic Congenital Cytomegalovirus Infection	Gestational age of study population was not sufficiently stated.
Löppönen, H.	1989	Audiological findings of shunt-treated hydrocephalus in children	Gestational age of study population was not sufficiently stated.
Loundon, N.	2020	The French Cochlear Implant Registry (EPIIC): Perception and language results in infants with cochlear implantation under the age of 24 months	Gestational age of study population was not sufficiently stated.
Lourenco, E. A.	2008	Evoked response audiometry according to gender and age: findings and usefulness	Gestational age of study population was not sufficiently stated.
Loveridge-Easther, C.	2022	Harboyan syndrome with biallelic SLC4A11 pathogenic variants misdiagnosed as congenital CMV infection	Case report as unsuitable study type.
Lu, C. Y.	2018	Concurrent Hearing, Genetic, and Cytomegalovirus Screening in Newborns, Taiwan	Gestational age of study population was not sufficiently stated.

Lu, J. R.	2011	Screening for delayed-onset hearing loss in preschool children who previously passed the newborn hearing screening	Gestational age of study population was not sufficiently stated.
Lu, Y. Q.	2019	Sudden Sensorineural Hearing Loss in Children: Clinical Characteristics, Etiology, Treatment Outcomes, and Prognostic Factors	Gestational age of study population was not sufficiently stated.
Lucignani, G.	2021	A new MRI severity score to predict long-term adverse neurologic outcomes in children with congenital Cytomegalovirus infection	Connection of risk factors and/or hearing outcome depending on gestational age was not established.
Luerssen, K.	2004	Niikawa-Kuroki (Kabuki) syndrome and hearing impairment	Case report as unsuitable study type.
Luntz, M.	2013	Risk factors for sensorineural hearing loss in chronic otitis media	Gestational age of study population was not sufficiently stated.
Luo, H.	2019	Novel recessive PDZD7 biallelic mutations associated with hereditary hearing loss in a Chinese pedigree	Gestational age of study population was not sufficiently stated.
Luts, H.	2006	Clinical application of dichotic multiple-stimulus auditory steady-state responses in high-risk newborns and young children	Connection of risk factors and/or hearing outcome depending on gestational age was not established.
Luu, K.	2020	Long-Term Otitis Media Outcomes in Infants With Early Tympanostomy Tubes	Gestational age of study population was not sufficiently stated.
Luxon, L. M.	2003	Neuro-otological findings in Pendred syndrome	Gestational age of study population was not sufficiently stated.
Luyster, R. J.	2011	The Modified Checklist for Autism in Toddlers in extremely low gestational age newborns: individual items associated with motor, cognitive, vision and hearing limitations	Checklist, sources screened for suitable literature for review question.
Ma, X. R.	2016	Chinese children with nonsyndromic cleft lip/palate: Factors associated with hearing disorder	Gestational age of study population was not sufficiently stated.
MacAndie, C.	2003	Epidemiology of permanent childhood hearing loss in Glasgow, 1985-1994	Connection of risk factors and/or hearing outcome depending on gestational age was not established.
MacDonald, H.	1978	Congenital cytomegalovirus infection: a collaborative study on epidemiological, clinical and laboratory findings	Gestational age of study population was not sufficiently stated.
Mace, A. L.	1991	Relevant factors in the identification of hearing loss	Gestational age of study population was not sufficiently stated.
Madden, C.	2002	Pediatric cochlear implantation in auditory neuropathy	No comparison of hearing impairment between preterm and full-term born infants.
Madden, C.	2002	Clinical and audiological features in auditory neuropathy	No comparison of hearing impairment between preterm and full-term born infants.
Madden, C.	2005	Audiometric, clinical and educational outcomes in a pediatric symptomatic congenital cytomegalovirus (CMV) population with sensorineural hearing loss	No sufficient definition of prematurity stated.
Maddocks, S. T.	2020	Child functioning and disability in children living with human immunodeficiency virus in a semi-rural healthcare setting in South Africa	Gestational age of study population was not sufficiently stated.
Maes, L.	2014	Rotatory and Collic Vestibular Evoked Myogenic Potential Testing in Normal- Hearing and Hearing-Impaired Children	Gestational age of study population was not sufficiently stated.
Maes, L.	2017	Comparison of the Motor Performance and Vestibular Function in Infants with a Congenital Cytomegalovirus Infection or a Connexin 26 Mutation: A Preliminary Study	Gestational age of study population was not sufficiently stated.
Mafong, D. D.	2002	Ocular findings in children with congenital sensorineural hearing loss	Gestational age of study population was not sufficiently stated.
Maggio, M. C.	2020	Brainstem Auditory Evoked Potentials and Visual Potentials in Kawasaki Disease: An Observational Monocentric Study	Gestational age of study population was not sufficiently stated.
Magnani, C.	2015	Universal newborn hearing screening: the experience of the University Hospital of Parma	No comparison of hearing impairment between preterm and full-term born infants.
Mahboubi, H.	2013	The prevalence and characteristics of tinnitus in the youth population of the United States	Gestational age of study population was not sufficiently stated.
Mahmoud, A. A.	2002	Cockayne syndrome in three sisters with varying clinical presentation	Case report as unsuitable study type.
Mahomva, C.	2022	Diagnosis of Auditory Neuropathy Spectrum Disorder in the Neonatal Intensive Care Unit Population	Gestational age of study population does not meet defined inclusion criteria for prematurity (<37 weeks) and/or full-term birth (≥37 weeks) with cut-off stated at 37 weeks.
Mahoney, K.	2017	Risk of neurodevelopmental impairment for outborn extremely preterm infants in an Australian regional network	No comparison of hearing impairment between preterm and full-term born infants.

Maisoun, A. M.	2003	Hearing screening of neonates at risk	Gestational age of study population was not sufficiently stated.
Majumder, A.	2017	Pediatric Langerhans cell histiocytosis of the lateral skull base	Gestational age of study population was not sufficiently stated.
Makar, S. K.	2012	Nature and onset of communication disorder in pediatrics with HIV	Gestational age of study population was not sufficiently stated.
Maki-Torkko, E. M.	1998	Aetiology and risk indicators of hearing impairments in a one-year birth cohort for 1985-86 in northern Finland	Only investigation of prematurity as risk factor for hearing impairment, other risk factors not analyzed comparing preterm and full-term born infants.
Malek, A.	2019	Risk Factors for Autistic Disorder: A Case-Control Study	Connection of risk factors and/or hearing outcome depending on gestational age was not established.
Malesci, R.	2021	Targeted Audiological Surveillance Program in Campania, Italy	Only investigation of prematurity as risk factor for hearing impairment, other risk factors not analyzed comparing preterm and full-term born infants.
Malesci, R.	2022	Performance and characteristics of the Newborn Hearing Screening Program in Campania region (Italy) between 2013 and 2019	Only investigation of prematurity as risk factor for hearing impairment, other risk factors not analyzed comparing preterm and full-term born infants.
Malhotra, A.	2020	Two-year outcomes of infants enrolled in the first-in-human study of amnion cells for bronchopulmonary dysplasia	No comparison of hearing impairment between preterm and full-term born infants.
Malhotra, M.	2022	The Clinical-Audiological Cross Sectional Study of Deaf-Mute Patients in a Tertiary Care Centre of Uttarakhand State and Literature Review	Only investigation of prematurity as risk factor for hearing impairment, other risk factors not analyzed comparing preterm and full-term born infants.
Mali, M.	1989	Developmental outcome of high-risk neonates in North Staffordshire	No comparison of hearing impairment between preterm and full-term born infants.
Mallen, J. R.	2018	Characterization of newborn hearing screening failures in multigestational births	Gestational age of study population was not sufficiently stated.
Mallouk, S.	2021	Specific aspects of tympanoplasty in children: A retrospective cohort study of 95 cases	Gestational age of study population was not sufficiently stated.
Malm, D.	1995	[Alpha-mannosidosis]	Review, sources screened for suitable literature for review question.
Mamoudjy, N.	2017	Neurological outcome of patients with cryopyrin-associated periodic syndrome (CAPS)	Gestational age of study population was not sufficiently stated.
Manara, R.	2011	Brain magnetic resonance findings in symptomatic congenital cytomegalovirus infection	Gestational age of study population was not sufficiently stated.
Mancini, M. L.	1996	Sensorineural hearing loss in patients reaching chronic renal failure in childhood	Gestational age of study population was not sufficiently stated.
Mancini, P.	2008	Follow-up of cochlear implant use in patients who developed bacterial meningitis following cochlear implantation	Gestational age of study population was not sufficiently stated.
Mancini, P.	2013	Contralateral implantation in children affected by postimplant meningitis	Gestational age of study population was not sufficiently stated.
Mandour, Y. M.	2020	Audiological assessment of neonatal hyperbilirubinemia	No comparison of hearing impairment between preterm and full-term born infants.
Manfredi, A. K.	2011	Newborn hearing screening in infants born to HIV-seropositive mothers	No comparison of hearing impairment between preterm and full-term born infants.
Maniu, A. A.	2016	Mastoiditis and facial paralysis as initial manifestations of temporal bone systemic diseases - the significance of the histopathological examination	Case report as unsuitable study type.
Mannan, M. A.	2014	Newborn hearing screening: what are we missing?	Connection of risk factors and/or hearing outcome depending on gestational age was not established.
Manning, S. C.	1994	Incidence of sensorineural hearing loss in patients evaluated for tympanostomy tubes	Gestational age of study population was not sufficiently stated.
Manotas, M.	2019	Risk factors associated with congenital defects that alter hearing or vision in children born in the city of Bogota between 2002 and 2016	Only investigation of gestational age as risk factor for hearing impairment, other risk factors not analyzed comparing preterm and full-term born infants.
Mao, M. H.	2017	Surgery combined with postoperative (125) I seed brachytherapy for the treatment of mucoepidermoid carcinoma of the parotid gland in pediatric patients	Gestational age of study population was not sufficiently stated.
Maqbool, M.	2015	Screening for Hearing Impairment in High Risk Neonates: A Hospital Based Study	Gestational age of study population was not sufficiently stated.
Marei, M. M.	2021	Intravesical gentamicin instillation for the treatment and prevention of urinary tract infections in complex paediatric urology patients: evidence for safety and efficacy	Gestational age of study population was not sufficiently stated.
Marin, L. J.	2016	Prevalence and clinical aspects of CMV congenital Infection in a low-income population	Gestational age of study population was not sufficiently stated.
Marinho, A. C. A.	2020	Evaluation of newborn hearing screening program	Connection of risk factors and/or hearing outcome depending on gestational age was not established.

Marissen, J.	2020	Vancomycin-induced ototoxicity in very-low-birthweight infants	No comparison of hearing impairment between preterm and full-term born infants.
Markova, T. G.	2008	[Clinical picture of hearing defects caused by Cx26 gene mutations]	No comparison of hearing impairment between preterm and full-term born infants.
Markowitz, P. I.	1983	Autism in a child with congenital cytomegalovirus infection	Case report as unsuitable study type.
Marlin, S.	2001	Connexin 26 gene mutations in congenitally deaf children: pitfalls for genetic counseling	Gestational age of study population was not sufficiently stated.
Marlow, E. S.	2000	Sensorineural hearing loss and prematurity	No comparison of hearing impairment between preterm and full-term born infants.
Maro, II.	2016	Auditory Impairments in HIV-Infected Children	Gestational age of study population was not sufficiently stated.
Marron, M. J.	1992	HEARING AND NEURODEVELOPMENTAL OUTCOME IN SURVIVORS OF PERSISTENT PULMONARY-HYPERTENSION OF THE NEWBORN	No comparison of hearing impairment between preterm and full-term born infants.
Marsico, C.	2019	Blood Viral Load in Symptomatic Congenital Cytomegalovirus Infection	Connection of risk factors and/or hearing outcome depending on gestational age was not established.
Martens, S.	2022	Three Years of Vestibular Infant Screening in Infants With Sensorineural Hearing Loss	Gestational age of study population was not sufficiently stated.
Martin, H. C.	2007	[Cisplatin-induced hearing loss in children in relation to eye color]	Gestational age of study population was not sufficiently stated.
Martin, J. A.	1982	Aetiological factors relating to childhood deafness in the European community	Gestational age of study population was not sufficiently stated.
Martin, L. D.	1990	Haemophilus influenzae meningitis with prolonged hospital course	Gestational age of study population was not sufficiently stated.
Martines, F.	2012	On the threshold of effective well infant nursery hearing screening in Western Sicily	Gestational age of study population was not sufficiently stated.
Martines, F.	2010	The point prevalence of otitis media with effusion among primary school children in Western Sicily	Gestational age of study population was not sufficiently stated.
Martines, F.	2010	The role of atopy in otitis media with effusion among primary school children: audiological investigation	Gestational age of study population was not sufficiently stated.
Martines, F.	2013	Prelingual sensorineural hearing loss and infants at risk: Western Sicily report	Only investigation of gestational age as risk factor for hearing impairment, other risk factors not analyzed comparing preterm and full-term born infants.
Martines, F.	2007	Newborn hearing screening project using transient evoked otoacoustic emissions: Western Sicily experience	Gestational age of study population was not sufficiently stated.
Martines, F.	2012	Audiologic profile of infants at risk: Experience of a Western Sicily tertiary care centre	No investigation of risk factors for hearing impairment.
Martinez-Cruz, C. F.	2012	Hearing loss, auditory neuropathy, and neurological co-morbidity in children with birthweight <750 g	No comparison of hearing impairment between preterm and full-term born infants.
Martinez-Cruz, C. F.	2009	Cognitive Performance of School Children with Unilateral Sensorineural Hearing Loss	Gestational age of study population was not sufficiently stated.
Martinez-Cruz, C. F.	2008	Risk factors associated with sensorineural hearing loss in infants at the neonatal intensive care unit: 15-year experience at the National Institute of Perinatology (Mexico City)	No comparison of hearing impairment between preterm and full-term born infants.
Martinez-Cruz, C. F.	2017	Changes in tonal audiometry in children with progressive sensorineural hearing loss and history of Neonatal Intensive Care Unit discharge. A 20 year long-term follow-up	No comparison of hearing impairment between preterm and full-term born infants.
Martinez-Cruz, C. F.	2020	Results of the Universal Neonatal Hearing Screening in a Tertiary Care Hospital in Mexico city	No comparison of hearing impairment between preterm and full-term born infants.
Martinez-Cruz, C. F.	1995	RISK-FACTORS FOR HYPOACUSIS AND AUDIOMETRIC FINDINGS IN PRESCHOOL-CHILDREN ADMITTED TO A NEONATAL INTENSIVE-CARE UNIT	No comparison of hearing impairment between preterm and full-term born infants.
Martinez-Pacheco, M. C.	2016	Delayed diagnosis of childhood deafness: the value of false negatives in the Programme for Early Detection of Neonatal Hearing Loss	No comparison of hearing impairment between preterm and full-term born infants.
Martinez, R.	2003	[Results of one year's application of a universal protocol for the early detection of hearing loss in neonates]	No comparison of hearing impairment between preterm and full-term born infants.
Martinez, R.	2003	[Results of the application of the protocol for the early detection of hearing loss in high-risk neonates]	No comparison of hearing impairment between preterm and full-term born infants.
Martinkova, J.	2010	Tolerability and Outcomes of Kinetically Guided Therapy With Gentamicin in Critically Ill Neonates	Connection of risk factors and/or hearing outcome depending on gestational age was not established.

		During the First Week of Life: An Open-Label, Prospective Study	
Martins, L. M. N.	2010	Hearing loss in cystic fibrosis	Gestational age of study population was not sufficiently stated.
Marttila, T. I.	2005	Initiators in processes leading to hearing loss identification in Finnish children	Gestational age of study population was not sufficiently stated.
Martu, C.	2022	Serous otitis media: Clinical and therapeutic considerations, including dexamethasone (C22H29FO5) intratympanic injection	Gestational age of study population was not sufficiently stated.
Maruyama, K.	2016	Arts syndrome with a novel missense mutation in the PRPS1 gene: A case report	Case report as unsuitable study type.
Marx, R. D.	2001	Spontaneous recovery of profound post-meningitic hearing loss	Case report as unsuitable study type.
Masarweh, K.	2021	The Yield of Targeted Examination for the Detection of Symptomatic Congenital Cytomegalovirus Infection	Gestational age of study population was not sufficiently stated.
Maspero, C.	2022	Incidental Finding in Pre-Orthodontic Treatment Radiographs of an Aural Foreign Body: A Case Report	Case report as unsuitable study type.
Masri, A.	2018	Recurrent meningitis in children: etiologies, outcome, and lessons to learn	Gestational age of study population was not sufficiently stated.
Massinger, C.	2003	[Cogan-I-syndrome. A rare differential diagnosis in progressive sensorineural hearing loss]	Case report as unsuitable study type.
Masterson, T.	2005	A case of the otogenic variant of Lemierre's syndrome with atypical sequelae and a review of pediatric literature	Case report as unsuitable study type.
Mastrangelo, A.	2020	X-Linked Alport Syndrome in Women: Genotype and Clinical Course in 24 Cases	Gestational age of study population was not sufficiently stated.
Masumoto, K.	2007	Risk factors for sensorineural hearing loss in survivors with severe congenital diaphragmatic hernia	No comparison of hearing impairment between preterm and full-term born infants.
Matanda, R. N.	2005	Chronic suppurative otitis media and related complications at the University Clinic of Kinshasa	Gestational age of study population was not sufficiently stated.
Matas, C. G.	2006	Audiological and electrophysiological evaluation of children with acquired immunodeficiency syndrome (AIDS)	Gestational age of study population was not sufficiently stated.
Matas, C. G.	2010	Audiological manifestations in children and adults with AIDS	No comparison of hearing impairment between preterm and full-term born infants.
Matin, F.	2021	Monitoring of the auditory pathway maturation after early intervention during the first year of life in infants with sensorineural hearing loss	No comparison of hearing impairment between preterm and full-term born infants.
Matin, M. A.	1997	A profile of 100 complicated cases of chronic suppurative otitis media	Gestational age of study population was not sufficiently stated.
Matsubara, K.	2002	Acute coalescent mastoiditis and acoustic sequelae in an infant with severe congenital neutropenia	Case report as unsuitable study type.
Matsui, T.	2012	Outcome of cochlear implantation in children with congenital cytomegalovirus infection or GJB2 mutation	Gestational age of study population was not sufficiently stated.
Matsuo, K.	2014	Quantitative evaluation of ventricular dilatation using computed tomography in infants with congenital cytomegalovirus infection	Connection of risk factors and/or hearing outcome depending on gestational age was not established.
Matsushige, T.	2009	Serial cerebrospinal fluid neurofilament concentrations in bacterial meningitis	Gestational age of study population was not sufficiently stated.
Mattos, J. L.	2014	Intratemporal and intracranial complications of acute otitis media in a pediatric population	Gestational age of study population was not sufficiently stated.
Mattos, W. M.	2009	Newborn hearing screening program implantation analysis at a University Hospital	No comparison of hearing impairment between preterm and full-term born infants.
Matula, K.	2012	Effect of Antenatal Treatment of Maternal Periodontitis on Early Childhood Neurodevelopment	No comparison of hearing impairment between preterm and full-term born infants.
Mauk, G. W.	1991	THE EFFECTIVENESS OF SCREENING PROGRAMS BASED ON HIGH-RISK CHARACTERISTICS IN EARLY IDENTIFICATION OF HEARING IMPAIRMENT	Gestational age of study population was not sufficiently stated.
Mayatepek, E.	1993	Deafness, complement deficiencies and immunoglobulin status in patients with meningococcal diseases due to uncommon serogroups	Gestational age of study population was not sufficiently stated.
Mayr, J.	1994	Psychosocial and psychomotoric development of very low birthweight infants with necrotizing enterocolitis	Connection of risk factors and/or hearing outcome depending on gestational age was not established.
Mazita, A.	2011	Cholesteatoma in patients with congenital external auditory canal anomalies: retrospective review	Gestational age of study population was not sufficiently stated.

Mazzaferri, F.	2017	Symptomatic congenital Cytomegalovirus deafness: the impact of a six-week course of antiviral treatment on hearing improvement	No comparison of hearing impairment between preterm and full-term born infants.
Mbou, F. M.	2005	[2 years of hearing screening of high-risk infants and children in Martinique]	Gestational age of study population was not sufficiently stated.
McBrien, J.	2009	Meeting the health care needs of school-age children with intellectual disability	Gestational age of study population was not sufficiently stated.
McClay, J. E.	2002	Major and minor temporal bone abnormalities in children with and without congenital sensorineural hearing loss	Gestational age of study population was not sufficiently stated.
McClelland, R. J.	1992	Reliability and effectiveness of screening for hearing loss in high risk neonates	No comparison of hearing impairment between preterm and full-term born infants.
McCrary, H.	2019	The Role of Antioxidants in the Treatment of Congenital CMV-Related Hearing: A Case-Control Study	Gestational age of study population was not sufficiently stated.
McCrary, H.	2019	Long-term hearing outcomes of children with symptomatic congenital CMV treated with valganciclovir	Gestational age of study population was not sufficiently stated.
McCrary, H.	2020	Outcomes from an Expanded Targeted Early Cytomegalovirus Testing Program	Gestational age of study population was not sufficiently stated.
McDonald, T. J.	1984	Congenital cholesteatoma of the ear	Gestational age of study population was not sufficiently stated.
McGee, T.	1992	Absence of sensorineural hearing loss in treated infants and children with congenital toxoplasmosis	Gestational age of study population was not sufficiently stated.
McGuire, D. O.	2019	Prevalence of cerebral palsy, intellectual disability, hearing loss, and blindness, National Health Interview Survey, 2009-2016	Gestational age of study population was not sufficiently stated.
McGurgan, I. J.	2014	Neonatal hearing screening of high-risk infants using automated auditory brainstem response: a retrospective analysis of referral rates	Gestational age of study population was not sufficiently stated.
McHugh, G.	2016	Chronic Morbidity Among Older Children and Adolescents at Diagnosis of HIV Infection	Gestational age of study population was not sufficiently stated.
McJunkin, J.	2010	Complications in pediatric cochlear implants	Gestational age of study population was not sufficiently stated.
McLaughlin, S. A.	2019	Listening Difficulties in Children With Fetal Alcohol Spectrum Disorders: More Than a Problem of Audibility	Gestational age of study population was not sufficiently stated.
McLean, D. R.	1992	Neurobrucellosis: clinical and therapeutic features	Gestational age of study population was not sufficiently stated.
McLeod, R.	2006	Outcome of treatment for congenital toxoplasmosis, 1981-2004: the National Collaborative Chicago-Based, Congenital Toxoplasmosis Study	Gestational age of study population was not sufficiently stated.
McMahon, C. M.	2021	Risk factors for Australian school-age children in socio-economically disadvantaged populations not passing ear and hearing screening	Gestational age of study population was not sufficiently stated.
Megantara, I.	2021	Relation between risk factor of hearing loss and the result of otoacoustic emission in newborns at Santosa Hospital Bandung Central	Only investigation of prematurity as risk factor for hearing impairment, other risk factors not analyzed comparing preterm and full-term born infants.
Megarbane, A.	2002	Craniofacial anomalies, deafness, brachydactyly, short stature, and moderate mental retardation due to a cryptic 6p;11q translocation	Case report as unsuitable study type.
Mehl, A. L.	2002	The Colorado Newborn Hearing Screening Project, 1992-1999: On the threshold of effective population-based universal newborn hearing screening	Gestational age of study population was not sufficiently stated.
Mehler, K.	2007	[Sensorineural loss of hearing in lower registers as the main symptom of Lyme disease]	Gestational age of study population was not sufficiently stated.
Mehrjoo, Z.	2020	Limbic System Associated Membrane Protein Mutation in an Iranian Family Diagnosed with M√©ni√®re's Disease	Gestational age of study population was not sufficiently stated.
Mehta, C. H.	2020	Vitamin D Deficiency, Hypocalcemia, and Hearing Loss in Children	Gestational age of study population was not sufficiently stated.
Meijer, A. J. M.		The cumulative incidence of cisplatin-induced hearing loss in young children is higher and develops at an early stage during therapy compared with older children based on 2052 audiological assessments	Gestational age of study population was not sufficiently stated.
Melamed, R.	2020	Targeted and universal screen in term and preterm infants for congenital CMV infection	Connection of risk factors and/or hearing outcome depending on gestational age was not established.
Mencher, L. S.	1999	Neonatal asphyxia, definitive markers and hearing loss	Only investigation of gestational age as risk factor for hearing impairment, other risk factors not analyzed comparing preterm and full-term born infants.
Menezes, M. J.	2015	Mutation in mitochondrial ribosomal protein S7 (MRPS7) causes congenital sensorineural deafness,	Gestational age of study population was not sufficiently stated.

		progressive hepatic and renal failure and lactic acidemia	
Menke, L. A.	2018	Further delineation of an entity caused by CREBBP and EP300 mutations but not resembling Rubinstein-Taybi syndrome	Gestational age of study population was not sufficiently stated.
Menser, M. A.	1974	Rubella--high incidence of defects in children considered normal at birth	Gestational age of study population was not sufficiently stated.
Menser, M. A.	1967	A twenty-five-year follow-up of congenital rubella	Gestational age of study population was not sufficiently stated.
Merchant, T. E.	2009	Late effects of conformal radiation therapy for pediatric patients with low-grade glioma: prospective evaluation of cognitive, endocrine, and hearing deficits	Gestational age of study population was not sufficiently stated.
Meredith, R.	1994	Screening for hearing loss in an at-risk neonatal population using evoked otoacoustic emissions	Gestational age of study population was not sufficiently stated.
Mertens, G.	2021	More than a quarter century of cochlear implantations: a retrospective study on 1161 implantations at the Antwerp University Hospital	Gestational age of study population was not sufficiently stated.
Mesolella, M.	2013	Management of otolaryngological manifestations in mucopolysaccharidoses: our experience	Gestational age of study population was not sufficiently stated.
Messerer, M.	2009	Hearing Loss Attributable to a Cerebellopontine-Angle Arachnoid Cyst in a Child	Case report as unsuitable study type.
Mestan, K. K.	2005	Neurodevelopmental outcomes of premature infants treated with inhaled nitric oxide	No comparison of hearing impairment between preterm and full-term born infants.
Meyer, C.	1999	Neonatal screening for hearing disorders in infants at risk: Incidence, risk factors, and follow-up	Only investigation of gestational age as risk factor for hearing impairment, other risk factors not analyzed comparing preterm and full-term born infants.
Meyer, L.	2017	Analysis of archived newborn dried blood spots (DBS) identifies congenital cytomegalovirus as a major cause of unexplained pediatric sensorineural hearing loss	No comparison of hearing impairment between preterm and full-term born infants.
Michaels, M. G.	2003	Treatment of children with congenital cytomegalovirus infection with ganciclovir	No comparison of hearing impairment between preterm and full-term born infants.
Michel, F.	2016	Progressively Recovering Auditory Brainstem Response in a Cochlear-implanted Child After Meningitis: A Case Report	Case report as unsuitable study type.
Michniewicz, B.	2022	Hearing Impairment in Infants with Hypoxic Ischemic Encephalopathy Treated with Hypothermia	No comparison of hearing impairment between preterm and full-term born infants.
Migirov, L.	2010	Mastoid subperiosteal abscess as a first sign of unnoticed cholesteatoma in children	Gestational age of study population was not sufficiently stated.
Migirov, L.	2005	Otogenic intracranial complications: A review of 28 cases	Gestational age of study population was not sufficiently stated.
Migirov, L.	2005	Mastoid subperiosteal abscess: a review of 51 cases	Gestational age of study population was not sufficiently stated.
Mikkola, K.	2005	Neurodevelopmental outcome at 5 years of age of a national cohort of extremely low birth weight infants who were born in 1996-1997	Only investigation of gestational age as risk factor for hearing impairment, other risk factors not analyzed comparing preterm and full-term born infants.
Milian, O. G.	2020	Relationship of Determined Risk Factors with Hearing Disorders in Children	No comparison of hearing impairment between preterm and full-term born infants.
Miller, T. E.	2021	Congenital Cytomegalovirus Infection Following Second and Third Trimester Maternal Infection Is Associated With Mild Childhood Adverse Outcome Not Predicted by Prenatal Imaging	No comparison of hearing impairment between preterm and full-term born infants.
Mills, R. P.	1984	Subjective tinnitus in children with otological disorders	Gestational age of study population was not sufficiently stated.
Milner, K. M.	2017	Neurodevelopmental outcomes for high-risk neonates in a low-resource setting	Connection of risk factors and/or hearing outcome depending on gestational age was not established.
Milner, L. S.	1983	Recurrent meningitis due to round-window fistula in Klippel-Feil syndrome. A case report	Case report as unsuitable study type.
Milshtein, N. Y.	2016	Acute Childhood Encephalitis at 2 Tertiary Care Children's Hospitals in Israel: Etiology and Clinical Characteristics	Gestational age of study population was not sufficiently stated.
Minami, S. B.	2013	Secondary, profound, sensorineural hearing loss after recovery from haemolytic uraemic syndrome due to enterohaemorrhagic Escherichia coli, and subsequent cochlear implantation, in two Japanese children	Case report as unsuitable study type.
Minami, S. B.	2021	A High Risk of Missing Congenital Cytomegalovirus-Associated Hearing Loss through Newborn Hearing Screening in Japan	Gestational age of study population was not sufficiently stated.
Miner, L. J.	2004	Large vancomycin overdose in two premature infants with minimal toxicity	Case report as unsuitable study type.

Ming, L.	2019	A Mutational Analysis of GJB2, SLC26A4, MT-RNA1, and GJB3 in Children with Nonsyndromic Hearing Loss in the Henan Province of China	Gestational age of study population was not sufficiently stated.
Minja, B. M.	1998	Aetiology of deafness among children at the Buguruni School for the Deaf in Dar es Salaam, Tanzania	Gestational age of study population was not sufficiently stated.
Minja, B. M.	2006	Chronic suppurative otitis media in Tanzanian school children and its effects on hearing	Gestational age of study population was not sufficiently stated.
Minoda, R.	2012	A postmeningitic cochlear implant patient who was postoperatively diagnosed as having X-linked agammaglobulinemia	Case report as unsuitable study type.
Minsart, A. F.	2020	Prenatal findings, neonatal symptoms and neurodevelopmental outcome of congenital cytomegalovirus infection in a university hospital in Montreal, Quebec	Connection of risk factors and/or hearing outcome depending on gestational age was not established.
Mishaal, R. A.	2022	Appraising the need for audiological assessment before autism spectrum disorder referral	Only investigation of prematurity as risk factor for hearing impairment, other risk factors not analyzed comparing preterm and full-term born infants.
Misono, S.	2011	Congenital cytomegalovirus infection in pediatric hearing loss	Gestational age of study population was not sufficiently stated.
Mitsuiki, N.	2017	Severe neonatal CMV infection complicated with thrombotic microangiopathy successfully treated with ganciclovir	Case report as unsuitable study type.
Miura, M.	2002	Analysis of spiral ganglion cell populations in children with normal and pathological ears	Gestational age of study population was not sufficiently stated.
Miyazaki, K.	2015	Long-term outcomes of antenatal corticosteroids treatment in very preterm infants after chorioamnionitis	No comparison of hearing impairment between preterm and full-term born infants.
Miyazaki, K.	2016	Impact of chorioamnionitis on short- and long-term outcomes in very low birth weight preterm infants: the Neonatal Research Network Japan	No comparison of hearing impairment between preterm and full-term born infants.
Mizuno, T.	2009	Detection of cytomegalovirus DNA in preserved umbilical cords from patients with sensorineural hearing loss	Gestational age of study population was not sufficiently stated.
Mizushima, N.	1986	Deafness following mumps: the possible pathogenesis and incidence of deafness	Gestational age of study population was not sufficiently stated.
Mjoen, S.	1982	Auditory brainstem responses (ABR) in high-risk neonates	No comparison of hearing impairment between preterm and full-term born infants.
Mkaouar-Rebai, E.	2006	Mutational analysis of the mitochondrial 12S rRNA and tRNASer(UCN) genes in Tunisian patients with nonsyndromic hearing loss	Gestational age of study population was not sufficiently stated.
Mkaouar, R.	2021	Alpha-mannosidosis in Tunisian consanguineous families: Potential involvement of variants in GHR and SLC19A3 genes in the variable expressivity of cognitive impairment	Gestational age of study population was not sufficiently stated.
Mo, W.	2005	[Hearing screening in infants with congenital cytomegalovirus infection]	No comparison of hearing impairment between preterm and full-term born infants.
Mochizuki, H.	2019	Peripheral neuropathy induced by drinking water contaminated with low-dose arsenic in Myanmar	Gestational age of study population was not sufficiently stated.
Moctar, E. C. M.	2016	Etiology and associated GJB2 mutations in Mauritanian children with non-syndromic hearing loss	Gestational age of study population was not sufficiently stated.
Mohammed, S. T.	2021	Auditory Brainstem Evoked Response Patterns in the Neonatal Intensive Care Unit	Only investigation of gestational age as risk factor for hearing impairment, other risk factors not analyzed comparing preterm and full-term born infants.
Mojica, A. M.	2019	GATA2 Deficiency in a Pediatric Patient	Case report as unsuitable study type.
Moke, D. J.	2021	Prevalence and risk factors for cisplatin-induced hearing loss in children, adolescents, and young adults: a multi-institutional North American cohort study	Gestational age of study population was not sufficiently stated.
Molini, E.	2016	Universal newborn hearing screening in Umbria region, Italy	Gestational age of study population was not sufficiently stated.
Molini, E.	2004	Identifying congenital hearing impairment. Personal experience based on selective hearing screening	No comparison of hearing impairment between preterm and full-term born infants.
Molyneux, E. M.	2017	The Treatment of Possible Severe Infection in Infants: An Open Randomized Safety Trial of Parenteral Benzylpenicillin and Gentamicin Versus Ceftriaxone in Infants < 60 days of Age in Malawi	Gestational age of study population was not sufficiently stated.
Molyneux, E. M.	2003	The effect of HIV infection on paediatric bacterial meningitis in Blantyre, Malawi	Gestational age of study population was not sufficiently stated.
MonsetCouchard, M.	1996	Mid- and long-term outcome of 89 premature infants weighing less than 1,000 g at birth, all appropriate for gestational age	Connection of risk factors and/or hearing outcome depending on gestational age was not established.

Moore, D. R.	2017	Lifetime leisure music exposure associated with increased frequency of tinnitus	Gestational age of study population was not sufficiently stated.
Moore, J. A.	1999	Comparison of risk of conductive hearing loss among three ethnic groups of arctic audiology patients	Gestational age of study population was not sufficiently stated.
Moore, T.	2012	Neurological and developmental outcome in extremely preterm children born in England in 1995 and 2006: the EPICure studies	No comparison of hearing impairment between preterm and full-term born infants.
Moore, T.	2012	Screening for autism in extremely preterm infants: problems in interpretation	No comparison of hearing impairment between preterm and full-term born infants.
Morales Angulo, C.	2003	[Program of hearing loss early detection in newborn infants in Cantabria. Results of the first year of activities]	No comparison of hearing impairment between preterm and full-term born infants.
Morando, C.	2010	Hearing assessment in high-risk congenital diaphragmatic hernia survivors	Connection of risk factors and/or hearing outcome depending on gestational age was not established.
Morava, E.	2012	Defining the phenotype in congenital disorder of glycosylation due to ALG1 mutations	Gestational age of study population was not sufficiently stated.
Moreno, G.	2013	[Clinical characterization of cases with meningococcal disease by W135 group in Chile, 2012]	No comparison of hearing impairment between preterm and full-term born infants.
Moresco, B. L.	2018	A Quiet Disease With Loud Manifestations	Case report as unsuitable study type.
Morgenstern Isaak, A.	2015	[Recurrent meningitis due to anatomical defects: The bacteria indicates its origin]	Case reports as unsuitable study types.
Morgenstern, C.	1989	[Cytomegalovirus infection and inner ear deafness]	Gestational age of study population was not sufficiently stated.
Morimoto, N.	2018	Homozygous EDNRB mutation in a patient with Waardenburg syndrome type 1	Gestational age of study population was not sufficiently stated.
Morimoto, N.	2010	Risk factors for elevation of ABR threshold in NICU-treated infants	Only investigation of gestational age as risk factor for hearing impairment, other risk factors not analyzed comparing preterm and full-term born infants.
Morimoto, N.	2006	Hearing loss in Turner syndrome	Gestational age of study population was not sufficiently stated.
Morini, F.	2008	Hearing impairment in congenital diaphragmatic hernia: the inaudible and noiseless foot of time	No comparison of hearing impairment between preterm and full-term born infants.
Morioka, I.	2020	Efficacy and safety of valganciclovir in patients with symptomatic congenital cytomegalovirus disease Study Protocol Clinical Trial (SPIRIT Compliant)	Connection of risk factors and/or hearing outcome depending on gestational age was not established.
Morioka, I.	1996	Hearing impairment among young Chinese in a rural area	Gestational age of study population was not sufficiently stated.
Morita, M.	1998	Clinical survey of congenital cytomegalovirus infection in Japan	Gestational age of study population was not sufficiently stated.
Morita, S.	2017	The clinical features and prognosis of mumps-associated hearing loss: a retrospective, multi-institutional investigation in Japan	Gestational age of study population was not sufficiently stated.
Morlet, T.	1998	Auditory screening in high-risk pre-term and full-term neonates using transient evoked otoacoustic emissions and brainstem auditory evoked potentials	No comparison of hearing impairment between preterm and full-term born infants.
Morlet, T.	2004	Assessment of medial olivocochlear system function in pre-term and full-term newborns using a rapid test of transient otoacoustic emissions	Connection of risk factors and/or hearing outcome depending on gestational age was not established.
Morlet, T.	2001	[Hearing disorders screening in neonates at risk]	Gestational age of study population was not sufficiently stated.
Moss, P. D.	1982	Outcome of meningococcal group B meningitis	Gestational age of study population was not sufficiently stated.
Mostafa, B. E.	2021	Genetic Screening for 35delG Mutation in Egyptian Patients with Profound Sensorineural Hearing Loss Scheduled for Cochlear Implantation: A Population-Based Study	Gestational age of study population was not sufficiently stated.
Mostafa, B. E.	2022	Maternal COVID-19 and neonatal hearing loss: a multicentric survey	No comparison of hearing impairment between preterm and full-term born infants.
Moteki, H.	2014	Evaluation of cortical processing of language by use of positron emission tomography in hearing loss children with congenital cytomegalovirus infection	Gestational age of study population was not sufficiently stated.
Mousavi, B.	2015	Epidemiological Study of Child Casualties of Landmines and Unexploded Ordnances: A National Study from Iran	Gestational age of study population was not sufficiently stated.
Moxon-Emre, I.	2021	Hearing loss and intellectual outcome in children treated for embryonal brain tumors: Implications for young children treated with radiation sparing approaches	Gestational age of study population was not sufficiently stated.

Msall, M. E.	2004	Developmental vulnerability and resilience in extremely preterm infants	Editorial, sources screened for suitable literature for review question.
Muelleman, T. J.	2021	Internal Auditory Canal Diverticula in Children: A Congenital Variant	Gestational age of study population was not sufficiently stated.
Muftah, S.	2015	Prevalence of Chronic Suppurative Otitis Media (CSOM) and Associated Hearing Impairment Among School-aged Children in Yemen	Gestational age of study population was not sufficiently stated.
Mukerji, S. S.	2007	Radiology quiz case 1. Labyrinthitis ossificans (LO)-stage of fibrosis	Case report as unsuitable study type.
Mukherjee, S. S.	2013	Prevalence of Hearing Loss in High Risk Infants of Mediocre Socio-economic Background at Around One Year of Age and Their Correlation with Risk Factors	Only investigation of prematurity as risk factor for hearing impairment, other risk factors not analyzed comparing preterm and full-term born infants.
Mukherjee, S. S.	2015	Using Bera To Detect Persistence of Auditory Injury In High Risk Infants	Gestational age of study population was not sufficiently stated.
Mulheran, M.	2001	Occurrence and risk of cochleotoxicity in cystic fibrosis patients receiving repeated high-dose aminoglycoside therapy	Gestational age of study population was not sufficiently stated.
Mulheran, M.	2006	Absence of cochleotoxicity measured by standard and high-frequency pure tone audiometry in a trial of once- versus three-times-daily tobramycin in cystic fibrosis patients	Gestational age of study population was not sufficiently stated.
Mulheran, M.	2004	Evidence of subtle auditory deficit in a group of patients recovered from bacterial meningitis	Gestational age of study population was not sufficiently stated.
Mullegama, S. V.	2017	De novo loss-of-function variants in STAG2 are associated with developmental delay, microcephaly, and congenital anomalies	Gestational age of study population was not sufficiently stated.
Mung'ala-Odera, V.	2006	Prevalence and risk factors of neurological disability and impairment in children living in rural Kenya	Gestational age of study population was not sufficiently stated.
Munir, S. B.	2021	Frequency of hearing impairment in children between the ages of 2 and 10 years with middle ear infection	Gestational age of study population was not sufficiently stated.
Muniz, L. F.	2022	Audiological follow-up of children with congenital Zika syndrome	Gestational age of study population was not sufficiently stated.
Munoz, O.	1983	Hearing loss after Hemophilus influenzae meningitis. Follow-up study with auditory brainstem potentials	Gestational age of study population was not sufficiently stated.
Munro, S. C.	2005	Symptomatic infant characteristics of congenital cytomegalovirus disease in Australia	Connection of risk factors and/or hearing outcome depending on gestational age was not established.
Murakami, S.	1996	[Clinical features and prognosis of facial palsy and hearing loss in patients with Ramsay Hunt syndrome]	Connection of risk factors and/or hearing outcome depending on gestational age was not established.
Murali, C. N.	2019	Muenke syndrome: Medical and surgical comorbidities and long-term management	Gestational age of study population was not sufficiently stated.
Muranjan, M.	2014	A mistaken identity: rhabdomyosarcoma of the middle ear cleft misdiagnosed as chronic suppurative otitis media with temporal lobe abscess	Case report as unsuitable study type.
Murgasova, L.	2020	Otorhinolaryngological manifestations in 61 patients with mucopolysaccharidosis	Gestational age of study population was not sufficiently stated.
Murhekar, M.	2020	Epidemiology of Congenital Rubella Syndrome (CRS) in India, 2016-18, based on data from sentinel surveillance	Gestational age of study population was not sufficiently stated.
Murinova, L'P.	2016	PCB exposure and cochlear function at age 6 years	Connection of risk factors and/or hearing outcome depending on gestational age was not established.
Murphy, C. C.	1995	Prevalence of epilepsy and epileptic seizures in 10-year-old children: results from the Metropolitan Atlanta Developmental Disabilities Study	Gestational age of study population was not sufficiently stated.
Murray, A. D.	1988	Newborn auditory brainstem evoked responses (ABRs): longitudinal correlates in the first year	Connection of risk factors and/or hearing outcome depending on gestational age was not established.
Murray, M.	2011	Sensorineural hearing loss at 9-13 years of age in children with a history of neonatal extracorporeal membrane oxygenation	No comparison of hearing impairment between preterm and full-term born infants.
Mussi-Pinhata, M. M.	2009	Birth Prevalence and Natural History of Congenital Cytomegalovirus Infection in a Highly Seroimmune Population	Connection of risk factors and/or hearing outcome depending on gestational age was not established.
Nadeem, M. S.	2018	Identification of variants in the mitochondrial lysine-tRNA (MT-TK) gene in myoclonic epilepsy-pathogenicity evaluation and structural characterization by in silico approach	Gestational age of study population was not sufficiently stated.
Nadeem, S.	2015	Renin Angiotensin System Blocker Fetopathy: A Midwest Pediatric Nephrology Consortium Report	No comparison of hearing impairment between preterm and full-term born infants.
Nadol, J. B.	1978	Hearing loss as a sequela of meningitis	Gestational age of study population was not sufficiently stated.

Naess, A.	1994	Sequelae one year after meningococcal disease	Gestational age of study population was not sufficiently stated.
Nafstad, P.	2002	Birth weight and hearing impairment in Norwegians born from 1967 to 1993	Only investigation of prematurity as risk factor for hearing impairment, other risk factors not analyzed comparing preterm and full-term born infants.
Nagamori, T.	2010	Single cytomegalovirus strain associated with fetal loss and then congenital infection of a subsequent child born to the same mother	Case report as unsuitable study type.
Nagarajan, L.	2010	Neurodevelopmental Outcomes in Neonates With Seizures: A Numerical Score of Background Encephalography to Help Prognosticate	Connection of risk factors and/or hearing outcome depending on gestational age was not established.
Nagasawa, K.	2016	Congenital Rubella Syndrome: A Case Report on Changes in Viral Load and Rubella Antibody Titers	Case report as unsuitable study type.
Nagy, A.	2004	Incidence and outcome of congenital cytomegalovirus infection in selected groups of preterm and full-term neonates under intensive care	Connection of risk factors and/or hearing outcome depending on gestational age was not established.
Nair, V.	2021	Permanent childhood hearing impairment in infants admitted to the neonatal intensive care unit: nested case-control study	No comparison of hearing impairment between preterm and full-term born infants.
Nakanishi, H.	2011	Hereditary isolated ossicular anomalies in two generations of patients	Case report as unsuitable study type.
Nakanishi, H.	2018	Trends in the neurodevelopmental outcomes among preterm infants from 2003-2012: a retrospective cohort study in Japan	No comparison of hearing impairment between preterm and full-term born infants.
Nakanishi, H.	2018	Persistent pulmonary hypertension of the newborn in extremely preterm infants: a Japanese cohort study'	No comparison of hearing impairment between preterm and full-term born infants.
Nakashima, T.	2004	Blood flow in the ears of patients receiving cochlear implants	Gestational age of study population was not sufficiently stated.
Nakku, D.	2017	HIV status and hearing loss among children between 6 and 12 years of age at a large urban health facility in south western Uganda	Gestational age of study population was not sufficiently stated.
Nam, G. S.	2019	Hyperbilirubinemia and Follow-up Auditory Brainstem Responses in Preterm Infants	No comparison of hearing impairment between preterm and full-term born infants.
Namyslowski, G.	2001	The hearing system in newborns from the Upper Silesia. Assessment of TEOAE depending on selected parameters of delivery disorders	No comparison of hearing impairment between preterm and full-term born infants.
Nance, W. E.	2006	Importance of congenital cytomegalovirus infections as a cause for pre-lingual hearing loss	Gestational age of study population was not sufficiently stated.
Nascimento, G. B.	2020	Risk indicators for hearing loss and language acquisition and their relationship with socioeconomic, demographic and obstetric variables in preterm and term babies	Hearing outcome not examined.
Nash, R.	2014	Vestibular function in children with auditory neuropathy spectrum disorder	No comparison of hearing impairment between preterm and full-term born infants.
Nasir, J. A.	2004	Investigation of the probable causes of specific childhood disabilities in eastern Afghanistan (preliminary report)	Gestational age of study population was not sufficiently stated.
Natale, F.	2020	Isolated auditory neuropathy at birth in congenital cytomegalovirus infection	Gestational age of study population was not sufficiently stated.
Nataprawira, H. M.	2016	Outcome of tuberculous meningitis in children: the first comprehensive retrospective cohort study in Indonesia	Gestational age of study population was not sufficiently stated.
Natarajan, G.	2018	Association between sedation-analgesia and neurodevelopment outcomes in neonatal hypoxic-ischemic encephalopathy	Connection of risk factors and/or hearing outcome depending on gestational age was not established.
Natarajan, G.	2014	Functional status at 18 months of age as a predictor of childhood disability after neonatal hypoxic-ischemic encephalopathy	Connection of risk factors and/or hearing outcome depending on gestational age was not established.
Navas, L.	1992	Initial therapy of bacterial meningitis with cefuroxime: Experience in 167 children	Gestational age of study population was not sufficiently stated.
Neagu, A.	2021	Prevalence of GJB2 gene mutations correlated to presence of clinical and environmental risk factors in the etiology of congenital sensorineural hearing loss of the Romanian population	Only investigation of prematurity as risk factor for hearing impairment, other risk factors not analyzed comparing preterm and full-term born infants.
Nekahm, D.	2001	Epidemiology of permanent childhood hearing impairment in the Tyrol, 1980-94	Gestational age of study population was not sufficiently stated.
Nepal, A.	2007	The morphology of central tympanic membrane perforations	Gestational age of study population was not sufficiently stated.
Neto, A. S.	1998	[Acute mastoiditis in children]	No comparison of hearing impairment between preterm and full-term born infants.
Neuman, A.	1981	Post-meningitic hearing loss: report on three cases	Case report as unsuitable study type.

Newall, J. P.	2020	A National Survey of Hearing Loss in the Philippines	Gestational age of study population was not sufficiently stated.
Newton, V.	1990	Hearing loss and Waardenburg's syndrome: implications for genetic counselling	Gestational age of study population was not sufficiently stated.
Newton, V. E.	1989	Genetic counselling for isolated hearing loss	Gestational age of study population was not sufficiently stated.
Ng, M. M. Y.	2020	Oval window perilymph fistula in child with recurrent meningitis and unilateral hearing loss	Case report as unsuitable study type.
Ngo, R. Y.	2006	Auditory neuropathy/auditory dys-synchrony detected by universal newborn hearing screening	Case report as unsuitable study type.
Nicoll, A. M.	1988	Ocular abnormalities in deaf children: a discussion of deafness and retinal pigment changes	Gestational age of study population was not sufficiently stated.
Nicolosi, L.	2004	[Streptococcus pneumoniae meningitis in children. Case records 1985 - 2003]	Case reports as unsuitable study types.
Nie, W. Y.	2007	A case-control study on high-risk factors for newborn hearing loss in seven cities of Shandong province	No comparison of hearing impairment between preterm and full-term born infants.
Niedzielska, G.	2000	Hearing defects in children born of mothers suffering from rubella in the first trimester of pregnancy	Gestational age of study population was not sufficiently stated.
Niehaus, H. H.	1995	[Early detection and hearing aid management of pediatric unilateral hearing loss]	Gestational age of study population was not sufficiently stated.
Nigro, G.	2012	Immunoglobulin therapy of fetal cytomegalovirus infection occurring in the first half of pregnancy--a case-control study of the outcome in children	Gestational age of study population was not sufficiently stated.
Nigro, G.	1994	Ganciclovir therapy for symptomatic congenital cytomegalovirus infection in infants: a two-regimen experience	Gestational age of study population was not sufficiently stated.
Nijman, J.	2014	Genotype Distribution, Viral Load and Clinical Characteristics of Infants with Postnatal or Congenital Cytomegalovirus Infection	Connection of risk factors and/or hearing outcome depending on gestational age was not established.
Nijman, J.	2012	Hearing in preterm infants with postnatally acquired cytomegalovirus infection	No comparison of hearing impairment between preterm and full-term born infants.
Nikolopoulos, T. P.	2004	Assessing candidate children for cochlear implantation with the Nottingham Children's Implant Profile (NChIP): the first 200 children	Gestational age of study population was not sufficiently stated.
Ninane, J.	1991	Effectiveness and toxicity of cisplatin and doxorubicin (PLADO) in childhood hepatoblastoma and hepatocellular carcinoma: a SIOP pilot study	Gestational age of study population was not sufficiently stated.
Nishida, K.	2020	Prediction of Neurodevelopmental Impairment in Congenital Cytomegalovirus Infection by Early Postnatal Magnetic Resonance Imaging	No comparison of hearing impairment between preterm and full-term born infants.
Nishida, Y.	1983	Congenital rubella syndrome: function of equilibrium of 80 cases with deafness	Gestational age of study population was not sufficiently stated.
Nishio, S. Y.	2022	Etiology of hearing loss affects auditory skill development and vocabulary development in pediatric cochlear implantation cases	Gestational age of study population was not sufficiently stated.
Nishioka, K.	1987	Bilateral sensorineural hearing loss associated with Mycoplasma pneumoniae infection	Case report as unsuitable study type.
Niu, K.	2020	Risk factors and etiology of childhood hearing loss: a cohort review of 296 subjects	Gestational age of study population was not sufficiently stated.
Nivoloni, K. D. B.	2010	Newborn hearing screening and genetic testing in 8974 Brazilian neonates	Connection of risk factors and/or hearing outcome depending on gestational age was not established.
Nixon, T. R. W.	2019	Homozygous Type IX collagen variants (COL9A1, COL9A2, and COL9A3) causing recessive Stickler syndrome-Expanding the phenotype	Gestational age of study population was not sufficiently stated.
Noda, M.	2018	Paediatric varicella zoster virus infection causing sudden hearing loss	Case report as unsuitable study type.
Noda, T.	2015	Cochlear implants for mumps deafness: two paediatric cases	Case report as unsuitable study type.
Noguchi, Y.	2017	A nationwide study on enlargement of the vestibular aqueduct in Japan	Gestational age of study population was not sufficiently stated.
Noonan, K. Y.	2016	CDH23 Related Hearing Loss: A New Genetic Risk Factor for Semicircular Canal Dehiscence?	Gestational age of study population was not sufficiently stated.
Noorbakhsh, S.	2011	Viral infections detected by serology and PCR of perilymphatic fluid in children with idiopathic sensorineural hearing loss	No comparison of hearing impairment between preterm and full-term born infants.
Noorbakhsh, S.	2022	Assessment of Hearing Loss in Two-Year Follow-up Study of Neonates with Congenital Cytomegalovirus Infection	Only investigation of prematurity as risk factor for hearing impairment, other risk factors not analyzed comparing preterm and full-term born infants.

Noorbakhsh, S.	2008	Sensorineural hearing loss due to Toxoplasma gondii in children: a case-control study	Gestational age of study population was not sufficiently stated.
Norgett, E. E.	2015	A role for VAX2 in correct retinal function revealed by a novel genomic deletion at 2p13.3 causing distal Renal Tubular Acidosis: case report	Case report as unsuitable study type.
Norhafizah, S.	2020	Prevalence of allergic rhinitis in children with otitis media with effusion	Gestational age of study population was not sufficiently stated.
Norowitz, H. L.	2019	Association between otitis media infection and failed hearing screenings in children	Gestational age of study population was not sufficiently stated.
Northam, W.	2019	Pediatric nonoperative skull fractures: delayed complications and factors associated with clinic and imaging utilization	Gestational age of study population was not sufficiently stated.
Norton, M. E.	2020	Hearing Loss With Congenital Cytomegalovirus Infection	Editorial, sources screened for suitable literature for review question.
Norton, S. J.	2000	Identification of neonatal hearing impairment: Summary and recommendations	Gestational age of study population was not sufficiently stated.
Norton, S. J.	2000	Identification of neonatal hearing impairment: A multicenter investigation	Gestational age of study population was not sufficiently stated.
Norton, S. J.	2000	Identification of neonatal hearing impairment: Evaluation of transient evoked otoacoustic emission, distortion product otoacoustic emission, and auditory brain stem response test performance	Gestational age of study population was not sufficiently stated.
Norton, S. J.	2000	Identification of neonatal hearing impairment: Transient evoked otoacoustic emissions during the perinatal period	Only investigation of gestational age as risk factor for hearing impairment, other risk factors not analyzed comparing preterm and full-term born infants.
Nowzari, H.	2001	Aggressive periodontitis associated with Fanconi's anemia. A case report	Case report as unsuitable study type.
Noyola, D. E.	2001	Early predictors of neurodevelopmental outcome in symptomatic congenital cytomegalovirus infection	No comparison of hearing impairment between preterm and full-term born infants.
Noyola, D. E.	2000	Cytomegalovirus urinary excretion and long term outcome in children with congenital cytomegalovirus infection. Congenital CMV Longitudinal Study Group	Gestational age of study population was not sufficiently stated.
Numazaki, K.	2003	Intracranial calcification with congenital rubella syndrome in a mother with serologic immunity	Case report as unsuitable study type.
Nunes, A. D. S.	2020	Prevalence of Hearing Loss and Associated Factors in School-Age Individuals in an Urban Area of Northeast Brazil	Gestational age of study population was not sufficiently stated.
Nunez-Batalla, F.	2008	[Incidence of hypoacusia secondary to hyperbilirubinaemia in a universal neonatal auditory screening programme based on otoacoustic emissions and evoked auditory potentials]	No comparison of hearing impairment between preterm and full-term born infants.
Nunez-Ramos, R.	2013	Early diagnosis of congenital cytomegalovirus infection: lost opportunities	No comparison of hearing impairment between preterm and full-term born infants.
Nunez, C.	2021	Microcephaly in Australian children, 2016-2018: national surveillance study	Connection of risk factors and/or hearing outcome depending on gestational age was not established.
Nussinovitch, M.	1995	Complications of mumps requiring hospitalization in children	Gestational age of study population was not sufficiently stated.
Nylen, O.	1979	Haemophilus influenzae meningitis and hearing	Gestational age of study population was not sufficiently stated.
O'Connor, A.	2013	Initial results from the newborn hearing screening programme in Ireland	Gestational age of study population was not sufficiently stated.
O'Hara, J.	2002	Prevalence of hearing impairment in siblings of deaf children	No comparison of hearing impairment between preterm and full-term born infants.
O'Hare, A. E.	1998	Avoidable late diagnosis of significant sensorineural hearing loss: implications for practice	Gestational age of study population was not sufficiently stated.
O'Leary, S. J.	2000	Abnormal positive potentials in round window electrocochleography	Only investigation of prematurity as risk factor for hearing impairment, other risk factors not analyzed comparing preterm and full-term born infants.
O'Rourke, D.	2010	Leukoencephalopathy with Anterior Temporal Cysts Due to Congenital CMV Infection Diagnosed Retrospectively	Case report as unsuitable study type.
O'Shea, S.	1992	A lymphocyte transformation assay for the diagnosis of congenital rubella	Gestational age of study population was not sufficiently stated.
Obiako, M. N.	1987	Profound childhood deafness in Nigeria: a three year survey	Connection of risk factors and/or hearing outcome depending on gestational age was not established.
Ocal, B.	1997	Prevalence of idiopathic long QT syndrome in children with congenital deafness	Gestational age of study population was not sufficiently stated.

Odabasi, O.	1998	Middle ear pathology in day-care centre children	Gestational age of study population was not sufficiently stated.
Ogawa, H.	2006	Congenital cytomegalovirus infection diagnosed by polymerase chain reaction with the use of preserved umbilical cord in sensorineural hearing loss children	Gestational age of study population was not sufficiently stated.
Ogawa, H.	2016	Presence of cytomegalovirus in the perilymphatic fluid of patients with profound sensorineural hearing loss caused by congenital cytomegalovirus infection	Gestational age of study population was not sufficiently stated.
Ogawa, H.	2007	Etiology of severe sensorineural hearing loss in children: Independent impact of congenital cytomegalovirus infection and GJB2 mutations	Gestational age of study population was not sufficiently stated.
Oghalai, J. S.	2002	Neonatal hearing loss in the indigent	No comparison of hearing impairment between preterm and full-term born infants.
Ogün, B.	2003	Long-term outcome of neonatal hyperbilirubinaemia: subjective and objective audiological measures	No comparison of hearing impairment between preterm and full-term born infants.
Oh, W.	2010	Influence of clinical status on the association between plasma total and unbound bilirubin and death or adverse neurodevelopmental outcomes in extremely low birth weight infants	Hearing outcome not examined separately.
Oh, W.	2003	Association between peak serum bilirubin and neurodevelopmental outcomes in extremely low birth weight infants	No comparison of hearing impairment between preterm and full-term born infants.
Ohl, C.	2009	Newborn hearing screening on infants at risk	Only investigation of prematurity as risk factor for hearing impairment, other risk factors not analyzed comparing preterm and full-term born infants.
Ohlms, L. A.	1999	Establishing the etiology of childhood hearing loss	Gestational age of study population was not sufficiently stated.
Ohls, R. K.	2014	Cognitive outcomes of preterm infants randomized to darbepoetin, erythropoietin, or placebo	No comparison of hearing impairment between preterm and full-term born infants.
Ohyama, S.	2019	Efficacy of Valganciclovir Treatment Depends on the Severity of Hearing Dysfunction in Symptomatic Infants with Congenital Cytomegalovirus Infection	No comparison of hearing impairment between preterm and full-term born infants.
Ojala, P.	1973	Rubella during pregnancy as a cause of congenital hearing loss	Gestational age of study population was not sufficiently stated.
Oka, S. I.	2020	Clinical Characteristics and In Vitro Analysis of MYO6 Variants Causing Late-Onset Progressive Hearing Loss	Gestational age of study population was not sufficiently stated.
Okhovat, S. A.	2011	Evaluation of hearing loss in juvenile insulin dependent patients with diabetes mellitus	Gestational age of study population was not sufficiently stated.
Olajuyin, O. A.	2021	Aetiologies of profound bilateral sensorineural hearing loss among children in Ekiti State, South Western Nigeria	Gestational age of study population was not sufficiently stated.
Oliveira, C.	2019	Congenital or Early Acquired Deafness: An Overview of the Portuguese Situation, from Diagnosis to Follow-Up	No comparison of hearing impairment between preterm and full-term born infants.
Oliveira, C. A.	2002	Deafness resulting from mutations in the GJB2 (connexin 26) gene in Brazilian patients	Gestational age of study population was not sufficiently stated.
Oliveira, C. S.	2013	Audiological abnormalities in patients with Turner syndrome	Gestational age of study population was not sufficiently stated.
Oliveira, J. S.	2013	Risk factors and prevalence of newborn hearing loss in a private health care system of Porto Velho, Northern Brazil	Gestational age of study population was not sufficiently stated.
Olusanya, B. O.	2003	Hearing impairment in children with impacted cerumen	Gestational age of study population was not sufficiently stated.
Olusanya, B. O.	2009	Newborns at risk of sensorineural hearing loss in low-income countries	Only investigation of gestational age as risk factor for hearing impairment, other risk factors not analyzed comparing preterm and full-term born infants.
Olusanya, B. O.	2010	Perinatal profile of very low birthweight infants under a universal newborn hearing screening programme in a developing country: A case-control study	No comparison of hearing impairment between preterm and full-term born infants.
Olusanya, B. O.	2010	Is undernutrition a risk factor for sensorineural hearing loss in early infancy?	No comparison of hearing impairment between preterm and full-term born infants.
Olusanya, B. O.	2011	Predictors of early-onset permanent hearing loss in malnourished infants in Sub-Saharan Africa	No comparison of hearing impairment between preterm and full-term born infants.
Olusanya, B. O.	2013	Risk of sensorineural hearing loss in infants with abnormal head size	No comparison of hearing impairment between preterm and full-term born infants.
Olusanya, B. O.	2006	Adverse perinatal conditions in hearing-impaired children in a developing country	Only investigation of prematurity as risk factor for hearing impairment, other risk factors not analyzed comparing preterm and full-term born infants.
Olusanya, B. O.	2018	Developmental disabilities among children younger than 5 years in 195 countries and territories, 1990-2016: a systematic analysis for the Global Burden of Disease Study 2016	Connection of risk factors and/or hearing outcome depending on gestational age was not established.

Olusanya, B. O.	2009	Universal infant hearing screening programme in a community with predominant non-hospital births: a three-year experience	No comparison of hearing impairment between preterm and full-term born infants.
Olusanya, B. O.	2004	Predictors of hearing loss in school entrants in a developing country	Connection of risk factors and/or hearing outcome depending on gestational age was not established.
Olusanya, B. O.	2000	The hearing profile of Nigerian school children	Gestational age of study population was not sufficiently stated.
Olusanya, B. O.	2010	Preference for private hospital-based maternity services in inner-city Lagos, Nigeria: An observational study	No comparison of hearing impairment between preterm and full-term born infants.
Olusanya, B. O.	2008	Non-hospital delivery and permanent congenital and early-onset hearing loss in a developing country	Only investigation of gestational age as risk factor for hearing impairment, other risk factors not analyzed comparing preterm and full-term born infants.
Olusanya, B. O.	2009	Maternal and neonatal factors associated with mode of delivery under a universal newborn hearing screening programme in Lagos, Nigeria	No comparison of hearing impairment between preterm and full-term born infants.
Olusanya, B. O.	2009	Place of birth and characteristics of infants with congenital and early-onset hearing loss in a developing country	Gestational age of study population was not sufficiently stated.
Olusanya, B. O.	2009	Infants with HIV-infected mothers in a universal newborn hearing screening programme in Lagos, Nigeria	No comparison of hearing impairment between preterm and full-term born infants.
Olusanya, B. O.	2009	Are risk factors for stillbirths in low-income countries associated with sensorineural hearing loss in survivors?	Only investigation of prematurity as risk factor for hearing impairment, other risk factors not analyzed comparing preterm and full-term born infants.
Olusanya, B. O.	2009	Infants with severe neonatal jaundice in Lagos, Nigeria: incidence, correlates and hearing screening outcomes	Connection of risk factors and/or hearing outcome depending on gestational age was not established.
Onesimo, R.	2021	Embryopathy Following Maternal Biliopancreatic Diversion: Is Bariatric Surgery Really Safe?	Case report as unsuitable study type.
Onoda, R. M.	2011	Neonatal Hearing Screening: failures, hearing loss and risk indicators	No comparison of hearing impairment between preterm and full-term born infants.
Oommen, S. P.	2019	Neurodevelopmental Outcomes of Very Low Birth Weight Infants at 18-24 Months, Corrected Gestational Age in a Tertiary Health Centre: A Prospective Cohort Study	No comparison of hearing impairment between preterm and full-term born infants.
Oostenbrink, R.	2002	Sequelae after bacterial meningitis in childhood	Gestational age of study population was not sufficiently stated.
Oostenbrink, R.	2002	Early prediction of neurological sequelae or death after bacterial meningitis	Gestational age of study population was not sufficiently stated.
Oosterom, N.	2015	Neuro-Imaging Findings in Infants with Congenital Cytomegalovirus Infection: Relation to Trimester of Infection	Connection of risk factors and/or hearing outcome depending on gestational age was not established.
Orgel, E.	2012	Hearing loss among survivors of childhood brain tumors treated with an irradiation-sparing approach	Gestational age of study population was not sufficiently stated.
Orgel, E.	2016	Effect of Sensorineural Hearing Loss on Neurocognitive Functioning in Pediatric Brain Tumor Survivors	Gestational age of study population was not sufficiently stated.
Orlando, M. P.	2019	Correlation between otitis media with effusion and cranial deformation in children	Gestational age of study population was not sufficiently stated.
Orman, G.	2020	Accuracy of MR Imaging for Detection of Sensorineural Hearing Loss in Infants with Bacterial Meningitis	Connection of risk factors and/or hearing outcome depending on gestational age was not established.
Orzan, E.	2021	Reliability of parental assessment of auditory skills in young children: a cross-sectional study in Italian language	Gestational age of study population was not sufficiently stated.
Orzan, E.	2018	Uncommon Post-Meningitis Hearing Threshold Improvement: A Case Report	Case report as unsuitable study type.
Osborn, D. A.	2007	Low superior vena cava flow and effect of inotropes on neurodevelopment to 3 years in preterm infants	No comparison of hearing impairment between preterm and full-term born infants.
Osei, A. O.	2018	Screening for hearing loss among school going children	Connection of risk factors and/or hearing outcome depending on gestational age was not established.
Oskovi-Kaplan, Z. A.	2022	Newborn Hearing Screening Results of Infants Born To Mothers Who Had COVID-19 Disease During Pregnancy: A Retrospective Cohort Study	No comparison of hearing impairment between preterm and full-term born infants.
Osman, K.	1999	Lead exposure and hearing effects in children in Katowice, Poland	Gestational age of study population was not sufficiently stated.
Ospina-Garcia, J. C.	2020	Prevalence of sensorineural hearing loss in newborns in a hospital from a developing country	Connection of risk factors and/or hearing outcome depending on gestational age was not established.
Ostergaard, C.	2005	Clinical presentation and prognostic factors of Streptococcus pneumoniae meningitis according to the focus of infection	Gestational age of study population was not sufficiently stated.

Otake, H.	2006	3D-FLAIR magnetic resonance imaging in the evaluation of mumps deafness	Case report as unsuitable study type.
Ou, Y. H.	2018	Aminoglycoside-associated nonsyndromic deafness and speech disorder in mitochondrial A1555G mutation in a family: A case report	Case report as unsuitable study type.
Ouahed, J.	2021	Variants in STXBP3 are Associated with Very Early Onset Inflammatory Bowel Disease, Bilateral Sensorineural Hearing Loss and Immune Dysregulation	Gestational age of study population was not sufficiently stated.
Ouellette, C. P.	2020	Blood genome expression profiles in infants with congenital cytomegalovirus infection	Connection of risk factors and/or hearing outcome depending on gestational age was not established.
Overcash, R. T.	2016	Maternal Iodine Exposure: A Case of Fetal Goiter and Neonatal Hearing Loss	Case report as unsuitable study type.
Oysu, C.	2002	Incidence of cochlear involvement in hyperbilirubinemic deafness	Connection of risk factors and/or hearing outcome depending on gestational age was not established.
Ozdamar, O.	1983	Auditory brainstem responses in infants recovering from bacterial meningitis. Audiologic evaluation	Only investigation of prematurity as risk factor for hearing impairment, other risk factors not analyzed comparing preterm and full-term born infants.
Ozdek, A.	2010	Successful Cochlear Implantation in a Child Deafened by Mumps	Case report as unsuitable study type.
Ozen, M.	2008	Long-term effects of dexamethasone on hearing ability in children with pneumococcal meningitis	Gestational age of study population was not sufficiently stated.
Ozturk, O.	2005	Evaluation of deaf children in a large series in Turkey	No comparison of hearing impairment between preterm and full-term born infants.
Ozturk, S. E. A.	2018	The follow-up results of newborn hearing screening of Gaziosmanpasa Taksim Research and Training Hospital	Gestational age of study population was not sufficiently stated.
Pabla, H. S.	1991	Retrospective study of the prevalence of bilateral sensorineural deafness in childhood	No comparison of hearing impairment between preterm and full-term born infants.
Padmadasan, S.	2022	Prevalence of Hearing Impairment in Neonates of Mothers with Diabetes Mellitus: A Cross Sectional Study	Connection of risk factors and/or hearing outcome depending on gestational age was not established.
Page, N.	2011	Radiology quiz case 2. Labyrinthitis ossificans secondary to suppurative labyrinthitis	Case report as unsuitable study type.
Pagnini, I.	2012	Clinical features and outcome of Cogan syndrome	Gestational age of study population was not sufficiently stated.
Palacios, G. C.	2008	Audiologic and vestibular findings in a sample of Human Immunodeficiency Virus type-1-infected Mexican children under Highly Active Antiretroviral Therapy	Gestational age of study population was not sufficiently stated.
Palfrey, J. S.	1980	Selective hearing screening for young children	No comparison of hearing impairment between preterm and full-term born infants.
Palma, S.	2021	What happens when the newborn hearing screening program is integrated with congenital Cytomegalovirus infection screening? Preliminary results in a tertiary hospital	Gestational age of study population was not sufficiently stated.
Palma, S.	2021	Hearing Loss in Children: Clinical-Epidemiological Data from Two Different Provinces of the Same Region	No comparison of hearing impairment between preterm and full-term born infants.
Palma, S.	2021	Unexpected hearing improvement after treatment with valganciclovir in a child with congenital cytomegalovirus infection	Case report as unsuitable study type.
Palma, S.	2021	Newborn hearing screening programme based on an integrated hospital and community care system. Results of the first 4 years of activity	Only investigation of prematurity as risk factor for hearing impairment, other risk factors not analyzed comparing preterm and full-term born infants.
Palma, S.	2019	Hearing loss in children with congenital cytomegalovirus infection: an 11-year retrospective study based on laboratory database of a tertiary paediatric hospital	Connection of risk factors and/or hearing outcome depending on gestational age was not established.
Palomo-Carrion, R.	2021	How Does the Cause of Infantile Hemiparesis Influence Other Conditioning Factors? A Preliminary Study in a Spanish Population	Hearing outcome not examined.
Pan, L.	2021	Risk factors for hearing loss in neonates admitted to neonatal intensive care units	Only investigation of prematurity as risk factor for hearing impairment, other risk factors not analyzed comparing preterm and full-term born infants.
Panahi, R.	2014	Relationship between behavioral hearing thresholds and estimated auditory steady-state response thresholds in children with a history of neonatal hyperbilirubinemia	Connection of risk factors and/or hearing outcome depending on gestational age was not established.
Panez-Gallardo, J. K.	2021	[Cytomegalovirus hepatitis in a 2-month-old infant: a case report]	Case report as unsuitable study type.
Papaevangelou, V.	2019	Neonatal screening for congenital CMV infection stresses the importance of maternal nonprimary infection even in an area where prenatal serology testing is common	Case report as unsuitable study type.

Paping, D. E.	2022	Risk Factors For Hearing Decline From Childhood To Early Adolescence	Gestational age of study population was not sufficiently stated.
Pappas, A.	2022	Blood Biomarkers and 6- to 7-Year Childhood Outcomes Following Neonatal Encephalopathy	Connection of risk factors and/or hearing outcome depending on gestational age was not established.
Pappas, D. G.	1983	Hearing impairments and vestibular abnormalities among children with subclinical cytomegalovirus	Gestational age of study population was not sufficiently stated.
Pappas, D. G.	1983	A study of the high-risk registry for sensorineural hearing impairment	Gestational age of study population was not sufficiently stated.
Pappas, D. G.	1982	Sensorineural hearing loss: infectious agents	Gestational age of study population was not sufficiently stated.
Papsin, B. C.	2007	Cochlear implants for children with severe-to-profound hearing loss	Gestational age of study population was not sufficiently stated.
Parab, S. R.	2018	Neonatal Screening for Prevalence of Hearing Impairment in Rural Areas	Gestational age of study population was not sufficiently stated.
Paradowska, E.	2014	Cytomegalovirus Glycoprotein H Genotype Distribution and the Relationship With Hearing Loss in Children	Gestational age of study population was not sufficiently stated.
Paramita, D. V.	2020	Profile of congenital rubella syndrome in Soetomo General Hospital Surabaya, Indonesia	Gestational age of study population was not sufficiently stated.
Parisier, S. C.	1976	Recurrent meningitis secondary to idiopathic oval window CSF leak	Case report as unsuitable study type.
Park, A. H.	2014	A Diagnostic Paradigm Including Cytomegalovirus Testing for Idiopathic Pediatric Sensorineural Hearing Loss	No comparison of hearing impairment between preterm and full-term born infants.
Park, A. H.	2000	Clinical course of pediatric congenital inner ear malformations	Gestational age of study population was not sufficiently stated.
Park, B.	2014	Analysis of the Prevalence of and Risk Factors for Tinnitus in a Young Population	Gestational age of study population was not sufficiently stated.
Park, H. J.	2017	Prevalence of hearing loss and associated factors in subjects with normal otoscopy: a national cross-sectional study	Gestational age of study population was not sufficiently stated.
Park, H. R.	2020	Homozygous mutations in Pakistani consanguineous families with prelingual nonsyndromic hearing loss	Gestational age of study population was not sufficiently stated.
Park, K. H.	2014	Prevalence and associated factors of tinnitus: data from the Korean National Health and Nutrition Examination Survey 2009-2011	Gestational age of study population was not sufficiently stated.
Parmar, S.	2019	Prevalence of Otitis Media with Effusion in Children with Hearing Loss	Gestational age of study population was not sufficiently stated.
Parner, E. T.	2007	Hearing loss diagnosis followed by meningitis in Danish children, 1995-2004	Gestational age of study population was not sufficiently stated.
Parodi, M.	2017	Childhood psychogenic hearing loss: Identification and diagnosis	Gestational age of study population was not sufficiently stated.
Partridge, E. A.	2014	Incidence and factors associated with sensorineural and conductive hearing loss among survivors of congenital diaphragmatic hernia	Only investigation of gestational age as risk factor for hearing impairment, other risk factors not analyzed comparing preterm and full-term born infants.
Parving, A.	1983	Epidemiology of hearing loss and aetiological diagnosis of hearing impairment in childhood	Gestational age of study population was not sufficiently stated.
Parving, A.	1984	Aetiological diagnosis in hearing-impaired children--clinical value and application of a modern examination programme	Gestational age of study population was not sufficiently stated.
Parving, A.	1977	Audiometric and ophthalmological findings in rubella deafness	Gestational age of study population was not sufficiently stated.
Parving, A.	1980	Congenital hearing loss and rubella infection	Gestational age of study population was not sufficiently stated.
Parving, A.	1994	The causes of profound hearing impairment in a school for the deaf--a longitudinal study	Gestational age of study population was not sufficiently stated.
Paryani, S. G.	1985	Sequelae of acquired cytomegalovirus infection in premature and sick term infants	Connection of risk factors and/or hearing outcome depending on gestational age was not established.
Pasman, J. W.	1992	The effect of preterm birth on brainstem, middle latency and cortical auditory evoked responses (BMC AERs)	Connection of risk factors and/or hearing outcome depending on gestational age was not established.
Pasquini, L.	2014	The utility of infection screening in isolated mild ventriculomegaly: an observational retrospective study on 141 fetuses	Connection of risk factors and/or hearing outcome depending on gestational age was not established.
Pass, R. F.	2006	Congenital cytomegalovirus infection following first trimester maternal infection: Symptoms at birth and outcome	Connection of risk factors and/or hearing outcome depending on gestational age was not established.

Pass, R. F.	1980	Outcome of symptomatic congenital cytomegalovirus infection: results of long-term longitudinal follow-up	Connection of risk factors and/or hearing outcome depending on gestational age was not established.
Passos, J.	2015	Late Cerebrovascular Complications After Radiotherapy for Childhood Primary Central Nervous System Tumors	Connection of risk factors and/or hearing outcome depending on gestational age was not established.
Pasternak, Y.	2018	Valganciclovir Is Beneficial in Children with Congenital Cytomegalovirus and Isolated Hearing Loss	Connection of risk factors and/or hearing outcome depending on gestational age was not established.
Pastorino, G.	2005	The Milan project: A newborn hearing screening programme	Connection of risk factors and/or hearing outcome depending on gestational age was not established.
Patatt, F. S. A.	2021	Hearing of neonates without risk indicators for hearing loss and use of antimalarial drugs during pregnancy: a historical cohort study in the Northern Region of Brazil	Connection of risk factors and/or hearing outcome depending on gestational age was not established.
Patel, V.	2015	Treatment of keratitis-ichthyosis-deafness (KID) syndrome in children: a case report and review of the literature	Case report as unsuitable study type.
Pathirana, J.	2020	Neurological and growth outcomes in South African children with congenital cytomegalovirus: A cohort study	No comparison of hearing impairment between preterm and full-term born infants.
Pati, S. K.	2013	Genotypic Diversity and Mixed Infection in Newborn Disease and Hearing Loss in Congenital Cytomegalovirus Infection	Gestational age of study population was not sufficiently stated.
Patra, K.	2006	Grades I-II intraventricular hemorrhage in extremely low birth weight infants: effects on neurodevelopment	Connection of risk factors and/or hearing outcome depending on gestational age was not established.
Paul, A.	2017	FDXR Mutations Cause Sensorial Neuropathies and Expand the Spectrum of Mitochondrial Fe-S-Synthesis Diseases	Gestational age of study population was not sufficiently stated.
Paul, A.	2017	Unilateral Sensorineural Hearing Loss: Medical Context and Etiology	Gestational age of study population was not sufficiently stated.
Paul, A. C.	2001	Malignant otitis externa in an infant with selective IgA deficiency: a case report	Case report as unsuitable study type.
Paul, V. K.	1998	Neurodevelopmental outcome of 'at risk' nursery graduates	Hearing outcome not examined.
Paulke-Korinek, M.	2014	Characteristics of invasive pneumococcal disease in hospitalized children in Austria	Gestational age of study population was not sufficiently stated.
Pearce, P. S.	1988	Hearing and verbal-cognitive abilities in high-risk preterm infants prone to otitis media with effusion	No comparison of hearing impairment between preterm and full-term born infants.
Pearson, F.	2013	Childhood infections, but not early life growth, influence hearing in the Newcastle thousand families birth cohort at age 14¬†years	Gestational age of study population was not sufficiently stated.
Peckham, C. S.	1972	Clinical and laboratory study of children exposed in utero to maternal rubella	Gestational age of study population was not sufficiently stated.
Peckham, C. S.	1979	Congenital rubella deafness: a preventable disease	Gestational age of study population was not sufficiently stated.
Peckham, C. S.	1987	Congenital cytomegalovirus infection: a cause of sensorineural hearing loss	Gestational age of study population was not sufficiently stated.
Pedersen, C. K.	2022	Prevalence and causes of paediatric hearing loss in a rural province of Zimbabwe: A cross-sectional study	Gestational age of study population was not sufficiently stated.
Pedersen, S. S.	1987	Cumulative and acute toxicity of repeated high-dose tobramycin treatment in cystic fibrosis	Gestational age of study population was not sufficiently stated.
Pedersen, T. I.	2010	Clinical characteristics of Haemophilus influenzae meningitis in Denmark in the post-vaccination era	Connection of risk factors and/or hearing outcome depending on gestational age was not established.
Pehere, N. K.	2019	Prevalence of ophthalmic disorders among hearing-impaired school children in Guntur district of Andhra Pradesh	Gestational age of study population was not sufficiently stated.
Peixoto, S.	2018	Low-grade intraventricular hemorrhage and neurodevelopment at 24 months of age	No comparison of hearing impairment between preterm and full-term born infants.
Peleva, E.	2014	Hearing loss in a pediatric patient following cisplatin chemotherapy and subsequent exposure to excessive noise	Case report as unsuitable study type.
Peleva, E.	2014	Incidence of Platinum-Induced Ototoxicity in Pediatric Patients in Quebec	Gestational age of study population was not sufficiently stated.
Pellegrinelli, L.	2019	Diagnosis of congenital CMV infection via DBS samples testing and neonatal hearing screening: an observational study in Italy	Gestational age of study population was not sufficiently stated.
Pellegrini, S.	2012	Intratemporal complications from acute otitis media in children: 17 cases in two years	No comparison of hearing impairment between preterm and full-term born infants.
Peltola, H.	2009	Improving the outcomes in children with bacterial meningitis	Gestational age of study population was not sufficiently stated.

Peltola, H.	1984	C-reactive protein as a detector of organic complications during recovery from childhood purulent meningitis	Gestational age of study population was not sufficiently stated.
Peltola, H.	2016	Predicting Outcome of Childhood Bacterial Meningitis With a Single Measurement of C-Reactive Protein	Gestational age of study population was not sufficiently stated.
Peltola, H.	2010	Hearing impairment in childhood bacterial meningitis is little relieved by dexamethasone or glycerol	Gestational age of study population was not sufficiently stated.
Peltola, H.	2007	Adjuvant glycerol and/or dexamethasone to improve the outcomes of childhood bacterial meningitis: a prospective, randomized, double-blind, placebo-controlled trial	Gestational age of study population was not sufficiently stated.
Penaranda, A.	2015	Otoscopic and audiological findings in different populations of 5-14 year-old schoolchildren in Colombia	Gestational age of study population was not sufficiently stated.
Peng, W.	2020	Low penetrance of hearing loss in two Chinese families carrying the mitochondrial tRNASer(UCN) mutations	Gestational age of study population was not sufficiently stated.
Pengpid, S.	2019	HIV status, knowledge, attitudes and behaviour of persons with and without disability in South Africa: evidence from a national population-based survey	Gestational age of study population was not sufficiently stated.
Penido, N. D.	2016	Complications of otitis media - a potentially lethal problem still present	Gestational age of study population was not sufficiently stated.
Pereira, P. K.	2007	[Newborn hearing screening program: association between hearing loss and risk factors]	The definition of preterm birth and term birth was not clear, with no indication of the limit of </≥ 37 weeks of gestation.
Perrott, S.	2003	A population-based study of prognostic factors related to major disability in very preterm survivors	Connection of risk factors and/or hearing outcome depending on gestational age was not established.
Persson, F.	2022	Hearing loss after bacterial meningitis, a retrospective study	Gestational age of study population was not sufficiently stated.
Perwein, T.	2011	Survival and Late Effects in Children With Stage 4 Neuroblastoma	Gestational age of study population was not sufficiently stated.
Pessoa, L.	2011	Clinical aspects of congenital syphilis with Hutchinson's triad	Case report as unsuitable study type.
Peter, V. Z., Paken, J. and Joseph, L.	2020	An audiological profile of a cohort of school-aged children with HIV and AIDS attending an antiretroviral clinic in South Africa	Gestational age of study population was not sufficiently stated.
Petersen, H.	2014	Long-term effects from bacterial meningitis in childhood and adolescence on postural control	Gestational age of study population was not sufficiently stated.
Peterson, J.	2020	Genetic Testing for Congenital Bilateral Hearing Loss in the Context of Targeted Cytomegalovirus Screening	Connection of risk factors and/or hearing outcome depending on gestational age was not established.
Petersson, S.	1999	Primary megalencephaly at birth and low intelligence level	Gestational age of study population was not sufficiently stated.
Phanguphangu, M.	2022	Auditory manifestations in HIV-infected children at a state hospital in South Africa	Gestational age of study population was not sufficiently stated.
Philip, T.	1987	A phase II study of high-dose cisplatin and VP-16 in neuroblastoma: a report from the Soci√©t√© Fran√ßaise d'Oncologie P√©diatrique	Gestational age of study population was not sufficiently stated.
Philips, B.	2014	Cochlear implants in children deafened by congenital cytomegalovirus and matched Connexin 26 peers	Gestational age of study population was not sufficiently stated.
Phipps, K.	2019	Childhood medulloblastoma-a single institution's historical perspective on survival and functional morbidity	Gestational age of study population was not sufficiently stated.
Picone, O.	2013	A series of 238 cytomegalovirus primary infections during pregnancy: description and outcome	Gestational age of study population was not sufficiently stated.
Pikis, A.	1996	Long-term sequelae of pneumococcal meningitis in children	Gestational age of study population was not sufficiently stated.
Pilania, R. K.	2019	Congenital rubella syndrome at tertiary care hospital in North India: Results from a retrospective assessment	Connection of risk factors and/or hearing outcome depending on gestational age was not established.
Pillion, J. P.	2008	Audiological findings in osteogenesis imperfecta	Gestational age of study population was not sufficiently stated.
Pinheiro, T.	2021	Congenital cytomegalovirus infection: from suspicion to confirmation	No comparison of hearing impairment between preterm and full-term born infants.
Pinninti, S. G.	2016	CLINICAL PREDICTORS OF SENSORINEURAL HEARING LOSS AND COGNITIVE OUTCOME IN INFANTS WITH SYMPTOMATIC CONGENITAL CYTOMEGALOVIRUS INFECTION	Gestational age of study population was not sufficiently stated.
Piotrowska, A.	2015	Tinnitus reported by children aged 7 and 12 years	Gestational age of study population was not sufficiently stated.

Pisacane, A.	2013	Feasibility and effectiveness of a population-based newborn hearing screening in an economically deprived region of Italy	Gestational age of study population was not sufficiently stated.
Pitaro, J.	2016	Sudden sensorineural hearing loss in children: Etiology, management, and outcome	Gestational age of study population was not sufficiently stated.
Pittet-Metrailler, M. P.	2019	Neurodevelopmental outcome at early school age in a Swiss national cohort of very preterm children	Only investigation of gestational age as risk factor for hearing impairment, other risk factors not analyzed comparing preterm and full-term born infants.
Plyler, E.	2021	Three Cases of Recovery from Sensorineural Hearing Loss in the First Year of Life: Implications for Monitoring and Management	Case report as unsuitable study type.
Polat, E.	2020	Assessment of Hearing Function in Children with Inflammatory Bowel Disease	Gestational age of study population was not sufficiently stated.
Polonenko, M. J.	2017	Cortical organization restored by cochlear implantation in young children with single sided deafness	Gestational age of study population was not sufficiently stated.
Pomeroy, S. L.	1990	Seizures and other neurologic sequelae of bacterial meningitis in children	Gestational age of study population was not sufficiently stated.
Poonual, W.	2016	Risk factors for hearing loss in infants under universal hearing screening program in Northern Thailand	Gestational age of study population was not sufficiently stated.
Poonual, W.	2017	Outcome of Early Identification and Intervention on Infants with Hearing Loss Under Universal Hearing Screening Program	Gestational age of study population was not sufficiently stated.
Poonual, W.	2017	Hearing loss screening tool (COBRA score) for newborns in primary care setting	Gestational age of study population was not sufficiently stated.
Popova, D. P.	2012	Prevalence of GBJ2 mutations in patients with severe to profound congenital nonsyndromic sensorineural hearing loss in Bulgarian population	Gestational age of study population was not sufficiently stated.
Postal, M.	2009	C1494T mitochondrial DNA mutation, hearing loss, and aminoglycosides antibiotics	Gestational age of study population was not sufficiently stated.
Potasman, I.	1995	Congenital toxoplasmosis: a significant cause of neurological morbidity in Israel?	Gestational age of study population was not sufficiently stated.
Pourarian, S.	2012	Prevalence of hearing loss in newborns admitted to neonatal intensive care unit	Only investigation of gestational age as risk factor for hearing impairment, other risk factors not analyzed comparing preterm and full-term born infants.
Powell, R. H.	1996	The Birmingham bone anchored hearing aid programme: paediatric experience and results	Gestational age of study population was not sufficiently stated.
Pozzi, R. S. L.	2021	Universal newborn hearing screening program and perinatal and congenital infections in neonates attended in South Brazil	Gestational age of study population was not sufficiently stated.
Prakash, R.		Importance of TORCH Profile in Hearing Impaired Paediatric Population Planned for Cochlear Implant: A Study From an Eastern India Tertiary Care Centre	Gestational age of study population was not sufficiently stated.
Preece, P. M.	1984	Congenital cytomegalovirus infection	Connection of risk factors and/or hearing outcome depending on gestational age was not established.
Prera, N.	2014	[Progressive hearing impairment with deletion in GJB2 gene despite normal newborn hearing screening]	Gestational age of study population was not sufficiently stated.
Preto, C.	2018	Congenital cytomegalovirus infection in an extremely preterm newborn exposed to chemotherapy in utero	Case report as unsuitable study type.
Prinsley, P.	2013	An audit of 'dead ear' after ear surgery	Gestational age of study population was not sufficiently stated.
Propst, E. J.	2006	Auditory responses in cochlear implant users with and without GJB2 deafness	Gestational age of study population was not sufficiently stated.
Pruszewicz, A.	2001	Low birth weight as a risk factor of hearing loss	No comparison of hearing impairment between preterm and full-term born infants.
Pryor, S. P.	2005	Investigation of the role of congenital cytomegalovirus infection in the etiology of enlarged vestibular aqueducts	Gestational age of study population was not sufficiently stated.
Psarommatis, I.	2011	Reversible auditory brainstem responses screening failures in high risk neonates	Gestational age of study population was not sufficiently stated.
Psarommatis, I.	2017	Recovery of Abnormal ABR in Neonates and Infants at Risk of Hearing Loss	Connection of risk factors and/or hearing outcome depending on gestational age was not established.
Puhakka, L.	2022	Hearing outcome in congenitally CMV infected children in Finland - Results from follow-up after three years age	Gestational age of study population was not sufficiently stated.
Puia-Dumitrescu, M.	2018	Evaluation of Gentamicin Exposure in the Neonatal Intensive Care Unit and Hearing Function at Discharge	No comparison of hearing impairment between preterm and full-term born infants.
Punia, R. S.	2019	Fungal Suppurative Otitis Media (Histopathology) Among Patients in North India	Gestational age of study population was not sufficiently stated.

Punnett, A.	2004	Ototoxicity following pediatric hematopoietic stem cell transplantation: A prospective cohort study	Gestational age of study population was not sufficiently stated.
Purdy, S. C.	2019	Hearing and ear status of Pacific children aged 11 years living in New Zealand: the Pacific Islands families hearing study	Gestational age of study population was not sufficiently stated.
Puttasiddaiah, P. M.	2020	Implantation of bone-anchored hearing device using a three-dimensional template in a child	Case report as unsuitable study type.
Pysden, K. S.	2009	Cogan's syndrome: a rare cause of meningoencephalitis	Case report as unsuitable study type.
Qaddoumi, I.	2012	Carboplatin-associated ototoxicity in children with retinoblastoma	Gestational age of study population was not sufficiently stated.
Qazi, S. A.	1996	Dexamethasone and bacterial meningitis in Pakistan	Gestational age of study population was not sufficiently stated.
Qian, Y.	2018	Sudden Sensorineural Hearing Loss in Children: A Report of 75 Cases	Gestational age of study population was not sufficiently stated.
Qu, C.	2012	Microarray-based mutation detection of pediatric sporadic nonsyndromic hearing loss in China	Gestational age of study population was not sufficiently stated.
Quintana, M. V.	2017	Evaluation of family history of permanent hearing loss in childhood as a risk indicatorin universal screening	Only investigation of gestational age as risk factor for hearing impairment, other risk factors not analyzed comparing preterm and full-term born infants.
Qvist, E.	2002	Neurodevelopmental outcome in high-risk patients after renal transplantation in early childhood	Hearing outcome not examined separately.
Rabico-Costa, D.	2020	Platinum-drugs Ototoxicity in Pediatric Patients With Brain Tumors: A 10-Year Review	Gestational age of study population was not sufficiently stated.
Rahko, T.	1978	Results of hearing testing at 7-year follow-up of kanamycin-treated newborn infants	Connection of risk factors and/or hearing outcome depending on gestational age was not established.
Rahko, T.	1995	HEARING AND ACUTE OTITIS-MEDIA IN 13-YEAR-OLD CHILDREN	Gestational age of study population was not sufficiently stated.
Rahman, M. M.	2002	Congenital hearing impairment associated with rubella: lessons from Bangladesh	Gestational age of study population was not sufficiently stated.
Rai, N.	2013	Universal screening of newborns to detect hearing impairment--is it necessary?	Connection of risk factors and/or hearing outcome depending on gestational age was not established.
Rai, N.		Role of Otoacoustic Emission Test in Early Diagnosis of Hearing Impairment in Infants	Connection of risk factors and/or hearing outcome depending on gestational age was not established.
Rais-Bahrami, K.	2004	Use of furosemide and hearing loss in neonatal intensive care survivors	Gestational age of study population was not sufficiently stated.
Raivio, M.	1978	Hearing disorders after Haemophilus influenzae meningitis. Comparison of different drug regimens	Gestational age of study population was not sufficiently stated.
Raja, N. S.	2006	Invasive meningococcal disease in the University of Malaya Medical Centre, Kuala Lumpur, Malaysia	Gestational age of study population was not sufficiently stated.
Rajagopal, R.	2017	Challenges of Treating Childhood Medulloblastoma in a Country With Limited Resources: 20 Years of Experience at a Single Tertiary Center in Malaysia	Gestational age of study population was not sufficiently stated.
Rajan, N.	2009	Tumor mapping in 2 large multigenerational families with CYLD mutations: implications for disease management and tumor induction	Gestational age of study population was not sufficiently stated.
Rajasingham, C. R.	2008	Serious neurologic sequelae in cases of meningitis arising from infection by conjugate vaccine-related and nonvaccine-related serogroups of Streptococcus pneumoniae	Gestational age of study population was not sufficiently stated.
Rajput, K.	2020	Ototoxicity-induced hearing loss and quality of life in survivors of paediatric cancer	Gestational age of study population was not sufficiently stated.
Ramirez Inscoe, J. M.	2004	Cochlear implantation in children deafened by cytomegalovirus: speech perception and speech intelligibility outcomes	Gestational age of study population was not sufficiently stated.
Ramma, L.	2016	The prevalence of hearing impairment within the Cape Town Metropolitan area	Gestational age of study population was not sufficiently stated.
Ramsay, M. E.	1991	Outcome of confirmed symptomatic congenital cytomegalovirus infection	Gestational age of study population was not sufficiently stated.
Rao, R. S.	2002	Hearing impairment and ear diseases among children of school entry age in rural South India	Gestational age of study population was not sufficiently stated.
Rastogi, S.	2013	Effects of ventilation on hearing loss in preterm neonates: Nasal continuous positive pressure does not increase the risk of hearing loss in ventilated neonates	No comparison of hearing impairment between preterm and full-term born infants.
Ratynska, J.	2001	Correlations between risk factors for hearing impairment and TEOAE screening test outcome in neonates at risk for hearing loss	Connection of risk factors and/or hearing outcome depending on gestational age was not established.

Rauscher, C.	2009	Pontine tegmental cap dysplasia: the severe end of the clinical spectrum	Case report as unsuitable study type.
Raveh, E.	2007	Auditory neuropathy: clinical characteristics and therapeutic approach	No comparison of hearing impairment between preterm and full-term born infants.
Rawlinson, W. D.	2018	Neonates with congenital Cytomegalovirus and hearing loss identified via the universal newborn hearing screening program	Gestational age of study population was not sufficiently stated.
Raynor, E.	2021	Loss to follow up of failed hearing screen and missed opportunities to detect congenital cytomegalovirus are better identified with the implementation of a new electronic health record system protocol	No comparison of hearing impairment between preterm and full-term born infants.
Raynor, E. M.		Impact of maternal cytomegalovirus seroconversion on newborn and childhood hearing loss triological thesis 2022-2023	Connection of risk factors and/or hearing outcome depending on gestational age was not established.
Raza, M.	2008	Frequency of Otitis Media with Effusion in Recurrent Upper Respiratory Tract Infection in Children	Gestational age of study population was not sufficiently stated.
Razi, M. S.	1994	Effects of adverse perinatal events on hearing	Connection of risk factors and/or hearing outcome depending on gestational age was not established.
Rechia, I. C.	2016	Intensive care unit: results of the Newborn Hearing Screening	No comparison of hearing impairment between preterm and full-term born infants.
Reddy, M. V.	2004	An epidemiological study on children with syndromic hearing loss	Gestational age of study population was not sufficiently stated.
Reeves, M. J.	2010	Neonatal cochlear function: measurement after exposure to acoustic noise during in utero MR imaging	Connection of risk factors and/or hearing outcome depending on gestational age was not established.
Reid, K.	2022	Health implications of maltreated children exposed to domestic violence	Gestational age of study population was not sufficiently stated.
Reinert, P.	1993	Epidemiology of Haemophilus influenzae type b disease in France	Gestational age of study population was not sufficiently stated.
Reis, F.	2019	Hearing Assessment of Neonates at Risk for Hearing Loss at a Hearing Health High Complexity Service: An Electrophysiological Assessment	Only investigation of prematurity as risk factor for hearing impairment, other risk factors not analyzed comparing preterm and full-term born infants.
Reiterer, E.	2019	A long-term follow-up study on otoacoustic emissions testing in paediatric patients with severe malaria in Gabon	Gestational age of study population was not sufficiently stated.
Ren, J.	2004	[Sudden sensorineural hearing loss in children]	Gestational age of study population was not sufficiently stated.
Resch, B.	2015	The whole spectrum of cystic periventricular leukomalacia of the preterm infant: results from a large consecutive case series	No comparison of hearing impairment between preterm and full-term born infants.
Restuti, R. D.	2021	Clinical and intraoperative findings for dangerous chronic suppurative otitis media in paediatric cases	Gestational age of study population was not sufficiently stated.
Rettenbach, R.	1999	Do deaf people see better? Texture segmentation and visual search compensate in adult but not in juvenile subjects	Gestational age of study population was not sufficiently stated.
Reyes, M. P.	1989	Vancomycin during pregnancy: does it cause hearing loss or nephrotoxicity in the infant?	Connection of risk factors and/or hearing outcome depending on gestational age was not established.
Reynolds, D. W.	1974	Inapparent congenital cytomegalovirus infection with elevated cord IgM levels. Casual relation with auditory and mental deficiency	Connection of risk factors and/or hearing outcome depending on gestational age was not established.
Reynolds, M. R.	2017	Vital Signs: Update on Zika Virus-Associated Birth Defects and Evaluation of All US Infants with Congenital Zika Virus Exposure - US Zika Pregnancy Registry, 2016	Gestational age of study population was not sufficiently stated.
Rheault, M. N.	2006	Sarcoidosis presenting with hearing loss and granulomatous interstitial nephritis in an adolescent	Case report as unsuitable study type.
Rhee, C. K.	1999	Audiologic evaluation of neonates with severe hyperbilirubinemia using transiently evoked otoacoustic emissions and auditory brainstem responses	No comparison of hearing impairment between preterm and full-term born infants.
Rhee, J.	2019	Hearing loss in Korean adolescents: The prevalence thereof and its association with leisure noise exposure	Gestational age of study population was not sufficiently stated.
Rhee, J.	2020	y Prevalence, associated factors, and comorbidities of tinnitus in adolescents	Gestational age of study population was not sufficiently stated.
Ricci, M. F.	2015	Chronic Neuromotor Disability After Complex Cardiac Surgery in Early Life	Gestational age of study population was not sufficiently stated.
Rice, M. L.	2012	Language impairment in children perinatally infected with HIV compared to children who were HIV-exposed and uninfected	Gestational age of study population was not sufficiently stated.
Rice, M. L.	2018	Risk for Speech and Language Impairments in Preschool Age HIV-exposed Uninfected Children With In Utero Combination Antiretroviral Exposure	Gestational age of study population was not sufficiently stated.

Richardson, M. P.	1997	Hearing loss during bacterial meningitis	Gestational age of study population was not sufficiently stated.
Richardson, M. P.	1997	Acute otitis media and otitis media with effusion in children with bacterial meningitis	Gestational age of study population was not sufficiently stated.
Richardson, M. P.	1998	Otoacoustic emissions as a screening test for hearing impairment in children recovering from acute bacterial meningitis	Gestational age of study population was not sufficiently stated.
Richter, D.	2001	A contiguous deletion syndrome of X-linked agammaglobulinemia and sensorineural deafness	Case report as unsuitable study type.
Riethmueller, J.	2009	Tobramycin Once- vs Thrice-Daily for Elective Intravenous Antipseudomonal Therapy in Pediatric Cystic Fibrosis Patients	Gestational age of study population was not sufficiently stated.
Rieubland, C.	2009	Two Cases of Trisomy 16 Mosaicism Ascertained Postnatally	Case report as unsuitable study type.
Riga, M.	2005	Etiological diagnosis of bilateral, sensorineural hearing impairment in a pediatric Greek population	Connection of risk factors and/or hearing outcome depending on gestational age was not established.
Rikitake, M.	2018	Bilateral Deafness as a Complication of the Vaccination-A Case Report	Case report as unsuitable study type.
Riley, D. N.	1997	Myringotomy and ventilation tube insertion: A ten-year follow-up	Gestational age of study population was not sufficiently stated.
Riordan, A.	1993	Children who are seen but not referred: hearing assessment after bacterial meningitis	Gestational age of study population was not sufficiently stated.
Rishi, E.	2018	Retinal Detachment in 31 Eyes with Retinitis Pigmentosa	Gestational age of study population was not sufficiently stated.
Rivera, T.	2001	[Hearing screening in children with risk factors of hearing loss in the area 3 of Madrid]	No comparison of hearing impairment between preterm and full-term born infants.
Roberts, J. L.	1982	Auditory brainstem responses in preterm neonates: maturation and follow-up	Connection of risk factors and/or hearing outcome depending on gestational age was not established.
Robertson, C. M.	1995	Neurodevelopmental outcome after neonatal extracorporeal membrane oxygenation	No comparison of hearing impairment between preterm and full-term born infants.
Robertson, C. M. T.	2019	Avoiding Furosemide Ototoxicity Associated With Single-Ventricle Repair in Young Infants	Gestational age of study population was not sufficiently stated.
Robertson, C. M. T.	2009	Permanent Bilateral Sensory and Neural Hearing Loss of Children After Neonatal Intensive Care Because of Extreme Prematurity: A Thirty-Year Study	No comparison of hearing impairment between preterm and full-term born infants.
Robertson, M. S.	2019	Asymmetric sensorineural hearing loss is a risk factor for late-onset hearing loss in pediatric cancer survivors following cisplatin treatment	Gestational age of study population was not sufficiently stated.
Robinson, G. C.	1964	HEARING LOSS IN INFANTS OF TUBERCULOUS MOTHERS TREATED WITH STREPTOMYCIN DURING PREGNANCY	Case report as unsuitable study type.
Roeser, R. J.	1975	Recovery of auditory function following meningitic deafness	Case report as unsuitable study type.
Roine, I.	2000	Randomized trial of four vs. seven days of ceftriaxone treatment for bacterial meningitis in children with rapid initial recovery	Gestational age of study population was not sufficiently stated.
Roine, I.	2015	Ataxia and Its Association with Hearing Impairment in Childhood Bacterial Meningitis	Gestational age of study population was not sufficiently stated.
Roine, I.	2014	Fluctuation in hearing thresholds during recovery from childhood bacterial meningitis	Gestational age of study population was not sufficiently stated.
Roine, I.	2013	Hearing impairment and its predictors in childhood bacterial meningitis in Angola	Gestational age of study population was not sufficiently stated.
Roine, I.	2009	Microbial genome count in cerebrospinal fluid compared with clinical characteristics in pneumococcal and Haemophilus influenzae type b meningitis in children	Gestational age of study population was not sufficiently stated.
Roine, I.	2010	Influence of Malnutrition on the Course of Childhood Bacterial Meningitis	Gestational age of study population was not sufficiently stated.
Roizenblatt, J.	1979	Median cleft face syndrome or frontonasal dysplasia: a case report with associated kidney malformation	Case report as unsuitable study type.
Romero, A. C.	2017	Auditory Alterations in Children Infected by Human Immunodeficiency Virus Verified Through Auditory Processing Test	Gestational age of study population was not sufficiently stated.
Ronchi, A.	2020	Evaluation of clinically asymptomatic high risk infants with congenital cytomegalovirus infection	No comparison of hearing impairment between preterm and full-term born infants.
Ronner, E. A.	2022	Congenital Cytomegalovirus Targeted Screening Implementation and Outcomes: A Retrospective Chart Review	Gestational age of study population was not sufficiently stated.

Rosalina, E.	2020	Congenital Rubella Syndrome profile of audiology outpatient clinic in Surabaya, Indonesia	Gestational age of study population was not sufficiently stated.
Rösch, D.	1998	[Acquired toxoplasmosis with cerebral involvement and subsequent hearing loss]	Case report as unsuitable study type.
Rosenhall, U.	1980	Hearing alterations following meningitis. 1. Hearing improvement	Case report as unsuitable study type.
Rosenhall, U.	1978	Auditory function after Haemophilus influenczae meningitis	Gestational age of study population was not sufficiently stated.
Rosenthal, L. S.	2009	Cytomegalovirus Shedding and Delayed Sensorineural Hearing Loss Results From Longitudinal Follow-up of Children With Congenital Infection	Gestational age of study population does not meet defined inclusion criteria for prematurity (<37 weeks) and/or full-term birth (≥37 weeks) with cut-off stated at 37 weeks.
Ross, D. S.	2010	Highly Variable Population-Based Prevalence Rates of Unilateral Hearing Loss After the Application of Common Case Definitions	Gestational age of study population was not sufficiently stated.
Ross, S. A.	2017	Newborn Dried Blood Spot Polymerase Chain Reaction to Identify Infants with Congenital Cytomegalovirus-Associated Sensorineural Hearing Loss	Gestational age of study population was not sufficiently stated.
Ross, S. A.	2006	Hearing loss in children with congenital cytomegalovirus infection born to mothers with preexisting immunity	Connection of risk factors and/or hearing outcome depending on gestational age was not established.
Ross, S. A.	2009	Cytomegalovirus Blood Viral Load and Hearing Loss in Young Children With Congenital Infection	Gestational age of study population was not sufficiently stated.
Ross, S. A.	2007	GJB2 and GJB6 mutations in children with congenital cytomegalovirus infection	Gestational age of study population was not sufficiently stated.
Roth, D. A.	2008	Preauricular skin tags and ear pits are associated with permanent hearing impairment in newborns	Gestational age of study population was not sufficiently stated.
Roth, D. A. E.	2006	Low prevalence of hearing impairment among very low birthweight infants as detected by universal neonatal hearing screening	No comparison of hearing impairment between preterm and full-term born infants.
Roth, D. A. E.	2017	Contribution of targeted saliva screening for congenital CMV-related hearing loss in newborns who fail hearing screening	No comparison of hearing impairment between preterm and full-term born infants.
Rout, N.	2008	Risk factors of hearing impairment in Indian children: a retrospective case-file study	Only investigation of prematurity as risk factor for hearing impairment, other risk factors not analyzed comparing preterm and full-term born infants.
Rovers, M. M.	2000	The effect of ventilation tubes on language development in infants with otitis media with effusion: A randomized trial	Connection of risk factors and/or hearing outcome depending on gestational age was not established.
Rovito, R.	2018	Impact of congenital cytomegalovirus infection on transcriptomes from archived dried blood spots in relation to long-term clinical outcome	No comparison of hearing impairment between preterm and full-term born infants.
Rowe, S. J.	1991	An evaluation of ABR audiometry for the screening and detection of hearing loss in ex-SCBU infants	No comparison of hearing impairment between preterm and full-term born infants.
Royackers, L.	2011	Hearing status in children with congenital cytomegalovirus: up-to-6-years audiological follow-up	Gestational age of study population was not sufficiently stated.
Royackers, L.	2013	Long-term audiological follow-up of children with congenital cytomegalovirus	Gestational age of study population was not sufficiently stated.
Rubie, H.	2003	Individual dosing of carboplatin based on drug monitoring in children receiving high-dose chemotherapy	Gestational age of study population was not sufficiently stated.
Rueegg, C. S.	2013	Health-related quality of life in survivors of childhood cancer: the role of chronic health problems	Gestational age of study population was not sufficiently stated.
Ruggieri, M.	2005	Earliest clinical manifestations and natural history of neurofibromatosis type 2 (NF2) in childhood: A study of 24 patients	Gestational age of study population was not sufficiently stated.
Ruiter, M.	2006	A novel 2.3 Mb microduplication of 12q24.21q24.23 detected by genome-wide tiling-path resolution array comparative genomic hybridization in a girl with syndromic mental retardation	Case report as unsuitable study type.
Rumstadt, J. W.	2012	Pedaudiological diagnostics in the first year of life. Clinical follow-up, risk factors, and middle ear function	No comparison of hearing impairment between preterm and full-term born infants.
Russ, S. A.	2002	Six year effectiveness of a population based two tier infant hearing screening programme	Gestational age of study population was not sufficiently stated.
Ruvalo, C.	1982	Intrauterinely acquired Pseudomonas infection in the neonate	Case report as unsuitable study type.
Ruzzenente, B.	2018	Inhibition of mitochondrial translation in fibroblasts from a patient expressing the KARS p.(Pro228Leu) variant and presenting with sensorineural deafness, developmental delay, and lactic acidosis	Gestational age of study population was not sufficiently stated.

Ryding, M.	2002	Auditory consequences of recurrent acute purulent otitis media	Gestational age of study population was not sufficiently stated.
Ryding, M.	2005	Hearing loss after "refractory" secretory otitis media	Gestational age of study population was not sufficiently stated.
Ryding, M.	2005	Course and long-term outcome of 'refractory' secretory otitis media	Gestational age of study population was not sufficiently stated.
Sabbag, J. C.	2017	Neonatal Hearing Screening in primary health care and family health care	Only investigation of prematurity as risk factor for hearing impairment, other risk factors not analyzed comparing preterm and full-term born infants.
Sabbagh, S.	2021	Neonatal Hearing Screening: Prevalence of Unilateral and Bilateral Hearing Loss and Associated Risk Factors	Only investigation of gestational age as risk factor for hearing impairment, other risk factors not analyzed comparing preterm and full-term born infants.
Sabroske, E.	2018	Passing the Newborn Hearing Screen Does Not Always Exclude Acquired Hearing Loss Due to Congenital Infection	Case report as unsuitable study type.
Sachdeva, K.	2017	Outcomes of Newborn Hearing Screening Program: A Hospital Based Study	Connection of risk factors and/or hearing outcome depending on gestational age was not established.
Sadarangani, M.	2015	Outcomes of invasive meningococcal disease in adults and children in Canada between 2002 and 2011: a prospective cohort study	Gestational age of study population was not sufficiently stated.
Sadeghi, A. M.	2013	Expressivity of hearing loss in cases with Usher syndrome type IIA	Gestational age of study population was not sufficiently stated.
Sadighi, J.	2005	Congenital rubella syndrome in Iran	Gestational age of study population was not sufficiently stated.
Saeed, H. S.	2022	Enlarged Vestibular Aqueduct: Disease Characterization and Exploration of Potential Prognostic Factors for Cochlear Implantation	Gestational age of study population was not sufficiently stated.
Saglam, D.	2017	Autosomal recessive osteopetrosis with a unique imaging finding: multiple encephaloceles	Case report as unsuitable study type.
Saha, A.	2013	Hearing status in children with frequently relapsing and steroid resistant nephrotic syndrome	Gestational age of study population was not sufficiently stated.
Saha, S. K.	2009	Neurodevelopmental sequelae in pneumococcal meningitis cases in Bangladesh: a comprehensive follow-up study	Gestational age of study population was not sufficiently stated.
Saigal, S.	1982	The outcome in children with congenital cytomegalovirus infection. A longitudinal follow-up study	Connection of risk factors and/or hearing outcome depending on gestational age was not established.
Sajjad, M.	2008	Causes of childhood deafness in Pukhtoonkhwa Province of Pakistan and the role of consanguinity	Gestational age of study population was not sufficiently stated.
Sakamoto, A.	2015	Retrospective diagnosis of congenital cytomegalovirus infection in children with autism spectrum disorder but no other major neurologic deficit	Connection of risk factors and/or hearing outcome depending on gestational age was not established.
Sakata, H.	2005	[A study of bacterial meningitis in Hokkaido between 1999 and 2003]	No comparison of hearing impairment between preterm and full-term born infants.
Sakata, H.	2000	[A study of bacterial meningitis in Hokkaido between 1994 and 1998]	No comparison of hearing impairment between preterm and full-term born infants.
Saki, N.	2019	Evaluation of cytomegalovirus DNA in periymphatic fluid in patients with sensorineural hearing loss using PCR technique	Gestational age of study population was not sufficiently stated.
Salamon, A. S.	2014	Neuroimaging and neurodevelopmental outcome of preterm infants with a periventricular haemorrhagic infarction located in the temporal or frontal lobe	No comparison of hearing impairment between preterm and full-term born infants.
Salamy, A.	1988	Neonatal status: an objective scoring method for identifying infants at risk for poor outcome	Connection of risk factors and/or hearing outcome depending on gestational age was not established.
Salamy, A.	1989	Neonatal status and hearing loss in high-risk infants	No comparison of hearing impairment between preterm and full-term born infants.
Salas, A. A.	2013	Histological characteristics of the fetal inflammatory response associated with neurodevelopmental impairment and death in extremely preterm infants	No comparison of hearing impairment between preterm and full-term born infants.
Saliba, M.	2007	Susac syndrome and ocular manifestation in a 14-year-old girl	Case report as unsuitable study type.
Salih, M. A.	1990	Childhood acute bacterial meningitis in the Sudan: an epidemiological, clinical and laboratory study	Gestational age of study population was not sufficiently stated.
Salih, M. A.	1990	Features of a large epidemic of group A meningococcal meningitis in Khartoum, Sudan in 1988	Gestational age of study population was not sufficiently stated.
Salih, M. A.	1990	Clinical features and complications of epidemic group A meningococcal disease in Sudanese children	Gestational age of study population was not sufficiently stated.
Salih, M. A.	1990	Endemic bacterial meningitis in Sudanese children: aetiology, clinical findings, treatment and short-term outcome	Gestational age of study population was not sufficiently stated.

Salih, M. A.	1991	Long term sequelae of childhood acute bacterial meningitis in a developing country. A study from the Sudan	Gestational age of study population was not sufficiently stated.
Salome, S.	2020	The Natural History of Hearing Disorders in Asymptomatic Congenital Cytomegalovirus Infection	No comparison of hearing impairment between preterm and full-term born infants.
Salt, A.	2006	Outcome at 2 years for very low birthweight infants in a geographical population: Risk factors, cost, and impact of congenital anomalies	Connection of risk factors and/or hearing outcome depending on gestational age was not established.
Saluja, S.	2010	Auditory neuropathy spectrum disorder in late preterm and term infants with severe jaundice	Connection of risk factors and/or hearing outcome depending on gestational age was not established.
Salvago, P.	2022	Risk Factors for Sensorineural Hearing Loss and Auditory Maturation in Children Admitted to Neonatal Intensive Care Units: Who Recovered?	Connection of risk factors and/or hearing outcome depending on gestational age was not established.
Salvago, P.	2013	Prevalence and risk factors for sensorineural hearing loss: Western Sicily overview	Only investigation of prematurity as risk factor for hearing impairment, other risk factors not analyzed comparing preterm and full-term born infants.
Salvinelli, F.	2004	Preserved otoacoustic emissions in postparotitis profound unilateral hearing loss: a case report	Case report as unsuitable study type.
Salwen, K. M.	1987	Increased incidence of childhood bacterial meningitis. A 25-year study in a defined population in Sweden	Gestational age of study population was not sufficiently stated.
Samdi, M. T.	2017	Risk Factors and Identifiable Causes of Hearing Impairment among Pediatric Age Group in Kaduna, Nigeria	Connection of risk factors and/or hearing outcome depending on gestational age was not established.
Samileh, N.	2008	Role of cytomegalovirus in sensorineural hearing loss of children: A case-control study Tehran, Iran	Gestational age of study population was not sufficiently stated.
Sampath, V.	2005	Risk factors for adverse neurodevelopment in extremely low birth weight infants with normal neonatal cranial ultrasound	No comparison of hearing impairment between preterm and full-term born infants.
Sanchez, N.	2019	Phenotypic spectrum of neonatal CHARGE syndrome	Connection of risk factors and/or hearing outcome depending on gestational age was not established.
Sanecka, A.	2016	QTc prolongation in patients with hearing loss: Electrocardiographic and genetic study	Gestational age of study population was not sufficiently stated.
Sanfins, M. D.	2020	Otoacoustic Emissions in Children with Long-Term Middle Ear Disease	Gestational age of study population was not sufficiently stated.
Sankar, J.	2007	Role of dexamethasone and oral glycerol in reducing hearing and neurological sequelae in children with bacterial meningitis	Gestational age of study population was not sufficiently stated.
Santorelli, F. M.	1996	Maternally inherited cardiomyopathy and hearing loss associated with a novel mutation in the mitochondrial tRNA(Lys) gene (G8363A)	Gestational age of study population was not sufficiently stated.
Santra, B.	2019	Heavy metal blood levels and hearing loss in children of West Bengal, India	Gestational age of study population was not sufficiently stated.
Sarafraz, M.	2009	A practical screening model for hearing loss in Iranian school-aged children	Gestational age of study population was not sufficiently stated.
Sarica, S.	2018	An evaluation of hearing in infants administered with colistin in the premature neonatal intensive care unit	No comparison of hearing impairment between preterm and full-term born infants.
Sarkar, S.	2010	Otitis Media with Effusion in Children and Its Correlation with Foreign Body in the External Auditory Canal	Gestational age of study population was not sufficiently stated.
Sarker, M. Z.	2020	Preoperative Evaluation and Surgical Outcome of Cochlear Implantation in NIENT	Only investigation of prematurity as risk factor for hearing impairment, other risk factors not analyzed comparing preterm and full-term born infants.
Sarmadi, A.	2020	A novel pathogenic variant in the LRTOMT gene causes autosomal recessive non-syndromic hearing loss in an Iranian family	Case report as unsuitable study type.
Sasireka, B. I.	2019	Clinical Experience on Hearing Screening in Twins and Triplets: A Retrospective Study	Connection of risk factors and/or hearing outcome depending on gestational age was not established.
Sassen, M. L.	1994	Otitis media, respiratory tract infections and hearing loss in pre-term and low birthweight infants	Connection of risk factors and/or hearing outcome depending on gestational age was not established.
Satish, H. S.	2019	Screening of Newborn Hearing at a Tertiary Care Hospital in South India	Only investigation of prematurity as risk factor for hearing impairment, other risk factors not analyzed comparing preterm and full-term born infants.
Sato, T.	2020	Outcomes of regional-based newborn hearing screening for 35,461 newborns for 5 years in Akita, Japan	Gestational age of study population was not sufficiently stated.
Satterfield-Nash, A.	2017	Health and Development at Age 19-24 Months of 19 Children Who Were Born with Microcephaly and Laboratory Evidence of Congenital Zika Virus Infection During the 2015 Zika Virus Outbreak - Brazil, 2017	Gestational age of study population was not sufficiently stated.
Saunders, J. E.	2009	Aminoglycoside ototoxicity in Nicaraguan children: Patient risk factors and mitochondrial DNA results	Gestational age of study population was not sufficiently stated.

Saunders, J. E.	2007	Prevalence and etiology of hearing loss in rural Nicaraguan children	Connection of risk factors and/or hearing outcome depending on gestational age was not established.
Sawhney, D.	2021	Down Syndrome Survey in South Indian Population-Understanding Inheritance, Perceptions, Interventions and Diagnosis	Gestational age of study population was not sufficiently stated.
Sayeb, M.	2019	A Tunisian family with a novel mutation in the gene CYP4F22 for lamellar ichthyosis and co-occurrence of hearing loss in a child due to mutation in the SLC26A4 gene	Case report as unsuitable study type.
Scaramuzzino, F.	2022	Secondary cytomegalovirus infections: How much do we still not know? Comparison of children with symptomatic congenital cytomegalovirus born to mothers with primary and secondary infection	Connection of risk factors and/or hearing outcome depending on gestational age was not established.
Schaad, U. B.	1993	Dexamethasone therapy for bacterial meningitis in children. Swiss Meningitis Study Group	Gestational age of study population was not sufficiently stated.
Schaad, U. B.	1990	A comparison of ceftriaxone and cefuroxime for the treatment of bacterial meningitis in children	Gestational age of study population was not sufficiently stated.
Schell, M. J.	1989	Hearing loss in children and young adults receiving cisplatin with or without prior cranial irradiation	Gestational age of study population was not sufficiently stated.
Schelonka, R. L.	2020	Mortality and Neurodevelopmental Outcomes in the Heart Rate Characteristics Monitoring Randomized Controlled Trial	No comparison of hearing impairment between preterm and full-term born infants.
Schendel, D.	2008	Birth weight and gestational age characteristics of children with autism, including a comparison with other developmental disabilities	Only investigation of gestational age as risk factor for hearing impairment, other risk factors not analyzed comparing preterm and full-term born infants.
Schildroth, A. N.	1994	Congenital Cytomegalovirus and Deafness	Gestational age of study population was not sufficiently stated.
Schimmenti, L. A.	2008	Infant hearing loss and connexin testing in a diverse population	Gestational age of study population was not sufficiently stated.
Schleiss, M. R.	2022	Antiviral Therapy and Its Long-Term Impact on Hearing Loss Caused by Congenital Cytomegalovirus: Much Remains to Be Learned!	Gestational age of study population was not sufficiently stated.
Schlottmann, A.	1996	[Sudden deafness and increased toxoplasmosis IgM titer]	Case report as unsuitable study type.
Schmidt, B.	2003	Impact of bronchopulmonary dysplasia, brain injury, and severe retinopathy on the outcome of extremely low-birth-weight infants at 18 months: results from the trial of indomethacin prophylaxis in preterms	Connection of risk factors and/or hearing outcome depending on gestational age was not established.
Schmidt, P.	2007	[Systematic hearing screening for newborns in the Champagne-Ardennes region: 32,500 births in 2 years of experience]	Gestational age of study population was not sufficiently stated.
Schmitz, J.	2021	Genome-wide association study and polygenic risk score analysis for hearing measures in children	Gestational age of study population was not sufficiently stated.
Schmitz, J.	2012	Vitamin A supplementation in preschool children and risk of hearing loss as adolescents and young adults in rural Nepal: randomised trial cohort follow-up study	Gestational age of study population was not sufficiently stated.
Schmutzhard, J.	2013	Release of intracranial pressure leads to improvement of otoacoustic emissions--a case report of a Kenyan child with complicated tuberculous meningitis	Case report as unsuitable study type.
Schmutzhard, J.	2015	Severe malaria in children leads to a significant impairment of transitory otoacoustic emissions--a prospective multicenter cohort study	Gestational age of study population was not sufficiently stated.
Schneeberger, P. M.	1994	Variable outcome of a congenital cytomegalovirus infection in a quadruplet after primary infection of the mother during pregnancy	Case report as unsuitable study type.
Schnohr, C.	2019	Self-reported hearing impairment among Greenlandic adolescents: Item development and findings from the Health Behaviour in School-aged Children study 2018	Gestational age of study population was not sufficiently stated.
Schnur, R. E.	1997	Acute lymphoblastic leukemia in a child with the CHIME neuroectodermal dysplasia syndrome	Case report as unsuitable study type.
Schonberger, J.	2000	Dilated cardiomyopathy and sensorineural hearing loss - A heritable syndrome that maps to 6q23-24	Gestational age of study population was not sufficiently stated.
Schönweiler, B.	2001	[Cochlear hearing loss following Mycoplasma pneumoniae infection]	Gestational age of study population was not sufficiently stated.
Schoub, B. D.	1990	Symptomatic rubella re-infection in early pregnancy and subsequent delivery of an infected but minimally involved infant. A case report	Case report as unsuitable study type.
Schreiber, J. E.	2014	Examination of risk factors for intellectual and academic outcomes following treatment for pediatric medulloblastoma	Gestational age of study population was not sufficiently stated.
Schrijver, I.	2006	Two patients with the V37I/235delC genotype: are radiographic cochlear anomalies part of the phenotype?	Case report as unsuitable study type.

Schumacher, R. E.	1991	Follow-up of infants treated with extracorporeal membrane oxygenation for newborn respiratory failure	No comparison of hearing impairment between preterm and full-term born infants.
Schwab, J.	2004	Varicella zoster virus meningitis in a previously immunized child	Case report as unsuitable study type.
Schwartz, J.	1987	Blood lead, hearing thresholds, and neurobehavioral development in children and youth	Gestational age of study population was not sufficiently stated.
Schwarz, Y.,	2017	Newborn hearing screening failure and maternal factors during pregnancy	Gestational age of study population was not sufficiently stated.
Scinicariello, F.	2019	Association of Obesity with Hearing Impairment in Adolescents	Gestational age of study population was not sufficiently stated.
Sculerati, N.	2000	Analysis of a cohort of children with sensory hearing loss using the SCALE systematic nomenclature	Only investigation of prematurity as risk factor for hearing impairment, other risk factors not analyzed comparing preterm and full-term born infants.
Seddon, J. A.	2013	Hearing loss in children treated for multidrug-resistant tuberculosis	Gestational age of study population was not sufficiently stated.
Sedel, F.	2006	Atypical Gilles de la Tourette syndrome with beta-mannosidase deficiency	Case report as unsuitable study type.
See, H.	2006	Chronic otitis and hearing loss revealing a disseminated tuberculosis in a child	Case report as unsuitable study type.
Seely, D. R.	1995	Hearing loss prevalence and risk factors among Sierra Leonean children	Gestational age of study population was not sufficiently stated.
Seethapathy, J.	2021	Distortion product otoacoustic emissions in very preterm infants: A longitudinal study	Only investigation of prematurity as risk factor for hearing impairment, other risk factors not analyzed comparing preterm and full-term born infants.
Seguya, A.	2021	Feasibility of establishing an infant hearing screening program and measuring hearing loss among infants at a regional referral hospital in south western Uganda	Only investigation of prematurity as risk factor for hearing impairment, other risk factors not analyzed comparing preterm and full-term born infants.
Seidman, M. D.	2018	Surgical Management for Dysplastic or Congenitally Absent Oval Window	Gestational age of study population was not sufficiently stated.
Seixas, R. R.	1993	[Objective auditory evaluation by evoked potentials]	No comparison of hearing impairment between preterm and full-term born infants.
Selander, J.	2016	Maternal Occupational Exposure to Noise during Pregnancy and Hearing Dysfunction in Children: A Nationwide Prospective Cohort Study in Sweden	Gestational age of study population was not sufficiently stated.
Selikowitz, M.	1992	Health problems and health checks in school-aged children with Down syndrome	Gestational age of study population was not sufficiently stated.
Sell, E. J.	1985	Persistent fetal circulation. Neurodevelopmental outcome	No comparison of hearing impairment between preterm and full-term born infants.
Sellars, S.	1976	Aetiology of deafness in white children in the Cape	Only investigation of prematurity as risk factor for hearing impairment, other risk factors not analyzed comparing preterm and full-term born infants.
Sells, C. J.	1975	Sequelae of central-nervous-system enterovirus infections	Gestational age of study population was not sufficiently stated.
Selten, J. P.	2015	Risks for nonaffective psychotic disorder and bipolar disorder in young people with autism spectrum disorder: a population-based study	Connection of risk factors and/or hearing outcome depending on gestational age was not established.
Sennaroglu, G.	2011	Risk Factors for Hearing Loss and Results of Newborn Hearing Screening in Rural Area	No comparison of hearing impairment between preterm and full-term born infants.
Sergi, P.	2001	A hospital based universal neonatal hearing screening programme using click-evoked otoacoustic emissions	Gestational age of study population was not sufficiently stated.
Serin, G.	2011	Auditory Screening Program of Newborns with Risk and Well Babies in Turkey	Gestational age of study population was not sufficiently stated.
Sethi, A.	2005	Primary tuberculous petrositis	Case report as unsuitable study type.
Sever, J. L.	1988	Toxoplasmosis: maternal and pediatric findings in 23,000 pregnancies	Gestational age of study population was not sufficiently stated.
Sha, Y.	2019	Novel NOG (p.P42S) mutation causes proximal symphalangism in a four-generation Chinese family	Gestational age of study population was not sufficiently stated.
Shah, I.	2014	High dose versus low dose steroids in children with tuberculous meningitis	Gestational age of study population was not sufficiently stated.
Shahar-Nissan, K.	2022	Retrospective identification of congenital cytomegalovirus infection using dried blood samples - missed opportunities and lessons	Connection of risk factors and/or hearing outcome depending on gestational age was not established.
Shahid, R.	2016	Risk Factors for Failed Newborn Otoacoustic Emissions Hearing Screen	Only investigation of gestational age as risk factor for hearing impairment, other risk factors not analyzed comparing preterm and full-term born infants.

Shan, R. B.	2009	Growth and Development of Infants with Asymptomatic Congenital Cytomegalovirus Infection	No comparison of hearing impairment between preterm and full-term born infants.
Shankaran, S.	2017	Effect of Depth and Duration of Cooling on Death or Disability at Age 18 Months Among Neonates With Hypoxic-Ischemic Encephalopathy A Randomized Clinical Trial	No comparison of hearing impairment between preterm and full-term born infants.
Sharawat, I. K.	2019	Recurrent Streptococcus pneumoniae meningitis and Mondini dysplasia: Association or causation?	Case report as unsuitable study type.
Shargorodsky, J.	2010	Change in Prevalence of Hearing Loss in US Adolescents	Gestational age of study population was not sufficiently stated.
Shargorodsky, J.	2011	Heavy Metals Exposure and Hearing Loss in US Adolescents	Gestational age of study population was not sufficiently stated.
Sharma, D.	2021	Early Neurodevelopmental Outcome of Neonates with Gestation 35 Weeks or More with Serum Bilirubin in Exchange Range Without Encephalopathy: A Prospective Observational Study	No comparison of hearing impairment between preterm and full-term born infants.
Sharma, K.	2016	Preventive Audiology: Screening for Hearing Impairment in Children Having Recurrent URTI	Gestational age of study population was not sufficiently stated.
Sharma, N.	2019	Assessment of risk factors for developmental delays among children in a rural community of North India: A cross-sectional study	Connection of risk factors and/or hearing outcome depending on gestational age was not established.
Sharma, S. C.	1989	Cochlear toxicity of streptomycin in man	Gestational age of study population was not sufficiently stated.
Sheahan, P.	2004	Middle ear disease in children with congenital velopharyngeal insufficiency	Gestational age of study population was not sufficiently stated.
Shehata-Dieler, W. E.	2002	[Universal newborn hearing screening program in W√ºrzburg. Experience with more than 4000 newborns and the influence of non-pathological factors on the test results]	No comparison of hearing impairment between preterm and full-term born infants.
Sher, G. and Naeem, M.	2014	A novel CHSY1 gene mutation underlies Temtamy preaxial brachydactyly syndrome in a Pakistani family	Gestational age of study population was not sufficiently stated.
Shi, K.	2021	Gadolinium-based contrast agent for Magnetic Resonance Imaging as a predictor of postmeningitic hearing loss in children	Connection of risk factors and/or hearing outcome depending on gestational age was not established.
Shigematsu, Y.	1991	Mucopolysaccharidosis VI (Maroteaux-Lamy syndrome) with hearing impairment and pupillary membrane remnants	Case report as unsuitable study type.
Shih, L.	1988	Effects of maternal cocaine abuse on the neonatal auditory system	Connection of risk factors and/or hearing outcome depending on gestational age was not established.
Shikano, H.	2015	Mondini dysplasia with recurrent bacterial meningitis caused by three different pathogens	Case report as unsuitable study type.
Shimizu, D.	2019	The evaluation of the appropriate gentamicin use for preterm infants	No comparison of hearing impairment between preterm and full-term born infants.
Shirin, S.	2008	Rare presentations of neurobrucellosis	Case report as unsuitable study type.
Shkalim-Zemer, V.	2015	Highly effective reduced toxicity dose-intensive pilot protocol for non-metastatic limb osteogenic sarcoma (SCOS 89)	Gestational age of study population was not sufficiently stated.
Shmueli, E.	2017	Congenital Cytomegalovirus Infection After a Multiple Birth Pregnancy	Connection of risk factors and/or hearing outcome depending on gestational age was not established.
Shokoohi, R.	2021	The relationship between chronic exposure to arsenic through drinking water and hearing function in exposed population aged 10-49 years: A cross-sectional study	Gestational age of study population was not sufficiently stated.
Shrestha, B. L.	2020	Universal Neonatal Hearing Screening: An Experience at Tertiary Care Hospital	No comparison of hearing impairment between preterm and full-term born infants.
Shukla, A.	2022	Role of Hearing Screening in High-Risk Newborns	Only investigation of gestational age as risk factor for hearing impairment, other risk factors not analyzed comparing preterm and full-term born infants.
Shusterman, D.	1992	Usefulness of computed tomographic scan in the evaluation of sensorineural hearing loss in children	Gestational age of study population was not sufficiently stated.
Shyama, M.	2001	Malocclusions and traumatic injuries in disabled schoolchildren and adolescents in Kuwait	Gestational age of study population was not sufficiently stated.
Sibley, C. H.	2015	A 24-month open-label study of canakinumab in neonatal-onset multisystem inflammatory disease	Gestational age of study population was not sufficiently stated.
Sibley, C. H.	2012	Sustained response and prevention of damage progression in patients with neonatal-onset multisystem inflammatory disease treated with anakinra: a cohort study to determine three- and five-year outcomes	Gestational age of study population was not sufficiently stated.

Siddique, A. K.		Prevalence of Hearing Impairment in High-Risk Neonates at Kalaburagi Region of Northern Karnataka: A Hospital-Based Cross-Sectional Study	Only investigation of prematurity as risk factor for hearing impairment, other risk factors not analyzed comparing preterm and full-term born infants.
Sidell, D.	2014	Risk Factors for Preoperative and Postoperative Hearing Loss in Children Undergoing Pressure Equalization Tube Placement	Connection of risk factors and/or hearing outcome depending on gestational age was not established.
Siegler, R. L.	1992	New syndrome involving the visual, auditory, respiratory, gastrointestinal, and renal systems	Case report as unsuitable study type.
Siem, G.	2010	Causes of hearing impairment in the Norwegian paediatric cochlear implant program	Connection of risk factors and/or hearing outcome depending on gestational age was not established.
Silkes, E. D.	1985	Progressive hearing loss following Haemophilus influenzae meningitis	Case report as unsuitable study type.
Silva, D. P.	2009	Analysis of transient otoacoustic emissions and brainstem evoked auditory potentials in neonates with hyperbilirubinemia	Gestational age of study population was not sufficiently stated.
Silva, D. P.	2013	Auditory steady state response in hearing assessment in infants with cytomegalovirus	Case report as unsuitable study type.
Silveira Netto, L. F.	2009	The impact of chronic suppurative otitis media on children's and teenagers' hearing	Gestational age of study population was not sufficiently stated.
Silver, M. K.	2018	Prenatal organophosphate insecticide exposure and infant sensory function	Connection of risk factors and/or hearing outcome depending on gestational age was not established.
Silvola, J. T.	2020	Endoscopic findings and long-term hearing results for pediatric unilateral conductive hearing loss	Gestational age of study population was not sufficiently stated.
Simchen, M. J.	2006	Fetal cardiac calcifications: report of four prenatally diagnosed cases and review of the literature	Case report as unsuitable study type.
Simoes, E. A.	2016	Otitis Media and Its Sequelae in Kenyan Schoolchildren	Gestational age of study population was not sufficiently stated.
Simon, T.	2002	[Efficiency and side effects of antiretroviral treatment of HIV infected pregnant women]	No comparison of hearing impairment between preterm and full-term born infants.
Simon, T.	2002	The incidence of hearing impairment after successful treatment of neuroblastoma	Gestational age of study population was not sufficiently stated.
Simpson, A.	2017	Socioeconomic status as a factor in Indigenous and non-Indigenous children with hearing loss: analysis of national survey data	Gestational age of study population was not sufficiently stated.
Simsek, G. K.	2020	Hearing screening failure rate in newborn infants with hypoxic ischemic encephalopathy	No comparison of hearing impairment between preterm and full-term born infants.
Singh, A.	2021	Association between Hyperbilirubinemia and Hearing Screen Failure in the Neonatal Intensive Care Unit in Infants Born Preterm	No comparison of hearing impairment between preterm and full-term born infants.
Singh, K.	1996	Auditory profile in children recovering from bacterial meningitis	Gestational age of study population was not sufficiently stated.
Singh, K. B.	1998	Comparative ABR profile in high risk infants	No comparison of hearing impairment between preterm and full-term born infants.
Singh, L.	2018	Early Neurodevelopmental Outcome of Very Low Birthweight Neonates with Culture- positive Blood Stream Infection: A Prospective Cohort Study	Connection of risk factors and/or hearing outcome depending on gestational age was not established.
Singh, S.	2020	Factors associated with deaf-mutism in children attending special schools of rural central India: A survey	Only investigation of prematurity as risk factor for hearing impairment, other risk factors not analyzed comparing preterm and full-term born infants.
Singhal, G.	2022	A Rare Case of Severe Post Adenoidectomy Secondary Haemorrhage	Case report as unsuitable study type.
Sininger, Y. S.	2000	Identification of neonatal hearing impairment: Auditory brain stem responses in the perinatal period	Gestational age of study population does not meet defined inclusion criteria for prematurity (<37 weeks) and/or full-term birth (≥37 weeks) with cut-off stated at 37 weeks.
Sitka, U.	1998	Hearing screening in neonates with risk factors of hearing impairment	No comparison of hearing impairment between preterm and full-term born infants.
Sivakumaran, T. A.	2013	Performance evaluation of the next-generation sequencing approach for molecular diagnosis of hereditary hearing loss	Gestational age of study population was not sufficiently stated.
Sizun, J.	1998	Neuro-intellectual outcome at school age for 62 children with gestational age under 32 weeks	Hearing outcome not examined.
Skevas, A.	1992	Perceptive bilateral deafness following clinical tetanus to a neonate	Case report as unsuitable study type.
Skou, A. S.	2014	Mitochondrial 12S ribosomal RNA A1555G mutation associated with cardiomyopathy and hearing loss following high-dose chemotherapy and repeated aminoglycoside exposure	Case report as unsuitable study type.

Slaghekke, F.	2014	Neurodevelopmental outcome in twin anemia-polycythemia sequence after laser surgery for twin-twin transfusion syndrome	Hearing outcome not examined.
Slovik, Y.	2020	Predicting hearing loss in children according to the referrer and referral cause	Gestational age of study population was not sufficiently stated.
Smiechura, M.	2014	Congenital and acquired cytomegalovirus infection and hearing evaluation in children	Gestational age of study population was not sufficiently stated.
Smilga, A. S.	2018	Neonatal Infection in Children With Cerebral Palsy: A Registry-Based Cohort Study	Hearing outcome not examined.
Smiljkovic, M.	2020	Blood viral load in the diagnostic workup of congenital cytomegalovirus infection	Connection of risk factors and/or hearing outcome depending on gestational age was not established.
Smit, E.	2013	Factors Associated with Permanent Hearing Impairment in Infants Treated with Therapeutic Hypothermia	Gestational age of study population does not meet defined inclusion criteria for prematurity (<37 weeks) and/or full-term birth (≥37 weeks) with cut-off stated at 37 weeks.
Smith, A.	2017	Permanent Childhood Hearing Impairment: Aetiological Evaluation of Infants identified through the Irish Newborn Hearing Screening Programme	Only investigation of prematurity as risk factor for hearing impairment, other risk factors not analyzed comparing preterm and full-term born infants.
Smith, A. F.	2017	Prevalence of hearing-loss among HAART-treated children in the Horn of Africa	Gestational age of study population was not sufficiently stated.
Smith, A. W.	1996	Randomised controlled trial of treatment of chronic suppurative otitis media in Kenyan schoolchildren	Gestational age of study population was not sufficiently stated.
Smyth, A.	2005	Once versus three-times daily regimens of tobramycin treatment for pulmonary exacerbations of cystic fibrosis - the TOPIC study: a randomised controlled trial	Gestational age of study population was not sufficiently stated.
Smyth, V.	1988	Audiological management in the recovery phase of bacterial meningitis	Gestational age of study population was not sufficiently stated.
Snedeker, J. D.	1990	Subdural effusion and its relationship with neurologic sequelae of bacterial meningitis in infancy: a prospective study	Gestational age of study population was not sufficiently stated.
Soares, J. C.	2009	Tone burst evoked otoacoustic emissions in neonates	No comparison of hearing impairment between preterm and full-term born infants.
Sobhani, M.	2019	Clinical and molecular assessment of 13 Iranian families with Wolfram syndrome	Gestational age of study population was not sufficiently stated.
Sobie, S.	1987	Necrotizing external otitis in children: report of two cases and review of the literature	Case report as unsuitable study type.
Sobol, S. E.	2004	Actinomycosis of the temporal bone: a report of a case	Case report as unsuitable study type.
Sochet, A. A.	2013	The importance of small for gestational age in the risk assessment of infants with critical congenital heart disease	No comparison of hearing impairment between preterm and full-term born infants.
Sogebi, O. A.	2021	Early hearing threshold changes and peculiarities of audiometric assessments among patients in a drug-resistant tuberculosis treatment center	Gestational age of study population was not sufficiently stated.
Sogebi, O. A.	2017	Hearing thresholds in patients with drug-resistant tuberculosis: baseline audiogram configurations and associations	Gestational age of study population was not sufficiently stated.
Sogebi, O. A.	2021	Asymptomatic Otitis Media With Effusion in Children With Adenoid Enlargement	Gestational age of study population was not sufficiently stated.
Soleimani, R.	2020	Comparing the prevalence of attention deficit hyperactivity disorder in hearing-impaired children with normal-hearing peers	Gestational age of study population was not sufficiently stated.
Soliman, S. E.	2018	Clinical and genetic associations for carboplatin-related ototoxicity in children treated for retinoblastoma: A retrospective noncomparative single-institute experience	Gestational age of study population was not sufficiently stated.
Solmaz, F.	2016	Does amikacin treatment cause subclinical hearing loss in patients with cystic fibrosis?	Gestational age of study population was not sufficiently stated.
Solorzano-Santos, F.	2001	[Congenital rubella syndrome in infants treated at a pediatrics hospital]	No comparison of hearing impairment between preterm and full-term born infants.
Song, C. M.	2021	Retinopathy of Prematurity and Hearing Impairment in Infants Born with Very-Low-Birth-Weight: Analysis of a Korean Neonatal Network Database	Connection of risk factors and/or hearing outcome depending on gestational age was not established.
Song, M. H.	2011	CHD7 mutational analysis and clinical considerations for auditory rehabilitation in deaf patients with CHARGE syndrome	Gestational age of study population was not sufficiently stated.
Sonoda, S.	2004	Two patients with severe corneal disease in KID syndrome	Case report as unsuitable study type.
Sorri, M.	1995	OTITIS-MEDIA AND LONG-TERM FOLLOW-UP OF HEARING	Gestational age of study population was not sufficiently stated.

Soul, J. S.	2021	A Pilot Randomized, Controlled, Double-Blind Trial of Bumetanide to Treat Neonatal Seizures	No comparison of hearing impairment between preterm and full-term born infants.
Sowinska-Seidler, A.	2015	Hyperosmia, ectrodactyly, mild intellectual disability, and other defects in a male patient with an X-linked partial microduplication and overexpression of the KAL1 gene	Case report as unsuitable study type.
Speleman, K.	2012	Prevalence of risk factors for sensorineural hearing loss in NICU newborns	Gestational age of study population was not sufficiently stated.
Sriyapai, T.	2022	Ototoxicity and long-term hearing outcome in pediatric patients receiving cisplatin	Gestational age of study population was not sufficiently stated.
Stadio, A. D.	2019	Sensorineural Hearing Loss in Newborns Hospitalized in Neonatal Intensive Care Unit: An Observational Study	Only investigation of prematurity as risk factor for hearing impairment, other risk factors not analyzed comparing preterm and full-term born infants.
Stagno, S.	1977	Auditory and visual defects resulting from symptomatic and subclinical congenital cytomegaloviral and toxoplasma infections	Gestational age of study population was not sufficiently stated.
Stamm, A. C.	1984	Nonspecific necrotizing petrositis: an unusual complication of otitis in children	Case report as unsuitable study type.
Stark, A. R.	2014	Death or neurodevelopmental impairment at 18 to 22 months corrected age in a randomized trial of early dexamethasone to prevent death or chronic lung disease in extremely low birth weight infants	No comparison of hearing impairment between preterm and full-term born infants.
Stark, C. R.	1984	Short- and long-term risks after exposure to diagnostic ultrasound in utero	Review, sources screened for suitable literature for review question.
Stehel, E. K.	2008	Newborn hearing screening and detection of congenital cytomegalovirus infection	Connection of risk factors and/or hearing outcome depending on gestational age was not established.
Stein-Zamir, C.	2014	The clinical features and long-term sequelae of invasive meningococcal disease in children	Gestational age of study population was not sufficiently stated.
Stein, L.	1983	The hearing-impaired infant: patterns of identification and habilitation	Gestational age of study population was not sufficiently stated.
Stein, L. K.	1990	The hearing-impaired infant: patterns of identification and habilitation revisited	Gestational age of study population was not sufficiently stated.
Steinlin, M. I.	1996	Late intrauterine Cytomegalovirus infection: Clinical and neuroimaging findings	Case report as unsuitable study type.
Steinmacher, J.	2008	Neurodevelopmental follow-up of very preterm infants after proactive treatment at a gestational age of > or = 23 weeks	No comparison of hearing impairment between preterm and full-term born infants.
Steinsvag, S. K.	2007	Nasal symptoms and signs in children suffering from asthma	Gestational age of study population was not sufficiently stated.
Stennert, E.	1978	The etiology of neurosensory hearing defects in preterm infants	No comparison of hearing impairment between preterm and full-term born infants.
Sterkers, J. M.	1980	[Mondini's syndrome. Recurrent meningitis. Surgical cure (author's transl)]	Case report as unsuitable study type.
Stevens, J. P.	2003	Long term outcome of neonatal meningitis	Connection of risk factors and/or hearing outcome depending on gestational age was not established.
Stewart, M. G.	1999	Is parental perception an accurate predictor of childhood hearing loss? A prospective study	Connection of risk factors and/or hearing outcome depending on gestational age was not established.
Stockard, J. E.	1990	Transient elevation of threshold of the neonatal auditory brain stem response	Gestational age of study population was not sufficiently stated.
Stocks, R. M.	1998	Malignant infantile osteopetrosis: otolaryngological complications and management	Case report as unsuitable study type.
Stoinska, B.	2011	Neurological and developmental disabilities in ELBW and VLBW: follow-up at 2 years of age	No comparison of hearing impairment between preterm and full-term born infants.
Stoll, B. J.	2004	Neurodevelopmental and growth impairment among extremely low-birth-weight infants with neonatal infection	No comparison of hearing impairment between preterm and full-term born infants.
Strachan, D.	1996	Long-term follow-up of children inserted with T-tubes as a primary procedure for otitis media with effusion	Gestational age of study population was not sufficiently stated.
Straughan, A. J.	2021	Feel the Burn! Fireworks-related Otolaryngologic Trauma	Gestational age of study population was not sufficiently stated.
Strauss, M.	1985	A clinical pathologic study of hearing loss in congenital cytomegalovirus infection	Gestational age of study population was not sufficiently stated.
Strebel, S.	2022	Severity of hearing loss after platinum chemotherapy in childhood cancer survivors	Gestational age of study population was not sufficiently stated.
Streitenberger, E. R.	2011	[Audiologic and molecular screening for hearing loss by 35delG mutation in connexin 26 gene and congenital cytomegalovirus infection]	No comparison of hearing impairment between preterm and full-term born infants.

Streppel, M.	1998	Epidemiology and etiology of acquired hearing disorders in childhood in the Cologne area	Connection of risk factors and/or hearing outcome depending on gestational age was not established.
Streppel, M.	2000	Epidemiology of hereditary hearing disorders in childhood - A retrospective study in Germany with special regard to ethnic factors	Gestational age of study population was not sufficiently stated.
Stritt, S.	2016	A gain-of-function variant in DIAPH1 causes dominant macrothrombocytopenia and hearing loss	Gestational age of study population was not sufficiently stated.
Strizek, B.	2015	Safety of MR Imaging at 1.5 T in Fetuses: A Retrospective Case-Control Study of Birth Weights and the Effects of Acoustic Noise	No comparison of hearing impairment between preterm and full-term born infants.
Stromland, K.	2007	Oculo-auriculo-vertebral spectrum: Associated anomalies, functional deficits and possible developmental risk factors	Connection of risk factors and/or hearing outcome depending on gestational age was not established.
Stromland, K.	2005	CHARGE association in Sweden: Malformations and functional deficits	Connection of risk factors and/or hearing outcome depending on gestational age was not established.
Su, B. M.	2017	Prevalence of Hearing Loss in US Children and Adolescents Findings From NHANES 1988-2010	Gestational age of study population was not sufficiently stated.
Sudan, M.	2013	Cell phone exposures and hearing loss in children in the Danish National Birth Cohort	No comparison of hearing impairment between preterm and full-term born infants.
Suganuma, E.	2018	10-year follow-up of congenital cytomegalovirus infection complicated with severe neurological findings in infancy: a case report	Case report as unsuitable study type.
Suganuma, E.	2021	Efficacy, safety, and pharmacokinetics of oral valganciclovir in patients with congenital cytomegalovirus infection	Connection of risk factors and/or hearing outcome depending on gestational age was not established.
Sugimoto, K.	2014	Childhood Cogan syndrome with aortitis and anti-neutrophil cytoplasmic antibody-associated glomerulonephritis	Case report as unsuitable study type.
Sugiura, M.	2005	Sudden sensorineural hearing loss associated with inner ear anomaly	Gestational age of study population was not sufficiently stated.
Sugiura, S.	2003	Detection of human cytomegalovirus DNA in perilymph of patients with sensorineural hearing loss using real-time PCR	Gestational age of study population was not sufficiently stated.
Sujatha, R.	2016	Prediction of Neurodevelopmental Outcome of Preterm Babies Using Risk Stratification Score	No comparison of hearing impairment between preterm and full-term born infants.
Sultesz, M.	2010	Prevalence and risk factors for allergic rhinitis in primary schoolchildren in Budapest	Gestational age of study population was not sufficiently stated.
Sumpter, R.	2011	Health-related quality-of-life and behavioural outcome in survivors of childhood meningitis	Gestational age of study population was not sufficiently stated.
Sun, B. Z.	2020	Association of Preeclampsia in Term Births With Neurodevelopmental Disorders in Offspring	Connection of risk factors and/or hearing outcome depending on gestational age was not established.
Sun, J. H.	2003	[Early detection of hearing impairment in high-risk infants of NICU]	Only investigation of gestational age as risk factor for hearing impairment, other risk factors not analyzed comparing preterm and full-term born infants.
Sun, L.	2015	[Features of clinical phenotype and genotype in Alport syndrome: a monocentric study]	Comparison of preterm infants with risk factors to term-born infants without risk factors cannot answer the research question.
Sun, Y.	2018	Novel RNASET2 Pathogenic Variants in an East Asian Child with Delayed Psychomotor Development	Gestational age of study population was not sufficiently stated.
Sundaram, A.	2017	Extended-interval Dosing of Gentamicin in Premature Neonates Born at < 32 Weeks' Gestation and > 7 Days of age	No comparison of hearing impairment between preterm and full-term born infants.
Sundqvist, A.	2014	Understanding minds: early cochlear implantation and the development of theory of mind in children with profound hearing impairment	Gestational age of study population was not sufficiently stated.
Sunwoo, W.	2018	Extremely common radiographic finding of cochlear nerve deficiency among infants with prelingual single-sided deafness and its clinical implications	Connection of risk factors and/or hearing outcome depending on gestational age was not established.
Suppiej, A.	2009	Neurodevelopmental outcome in preterm histological chorioamnionitis	No comparison of hearing impairment between preterm and full-term born infants.
Suresh, G.	1997	Lack of deafness in Crigler-Najjar syndrome type 1: A patient survey	Connection of risk factors and/or hearing outcome depending on gestational age was not established.
Sutton, G. J.	1997	Risk factors for childhood sensorineural hearing loss in the Oxford Region	Only investigation of gestational age as risk factor for hearing impairment, other risk factors not analyzed comparing preterm and full-term born infants.
Suwannatrai, P.	2022	Hearing screening outcomes in pediatric critical care survivors: a 1-year report	Gestational age of study population was not sufficiently stated.
Suzuki, N.	2004	Relation between predischarge auditory brainstem responses and clinical factors in high-risk infants	No comparison of hearing impairment between preterm and full-term born infants.

Suzuki, Y.	2009	Cochlear implantation in a case of bilateral sensorineural hearing loss due to mumps	Case report as unsuitable study type.
Svendsen, M. B.	2020	Neurological sequelae remain frequent after bacterial meningitis in children	Gestational age of study population was not sufficiently stated.
Swanepoel, D.	2013	Childhood hearing loss and risk profile in a South African population	Gestational age of study population was not sufficiently stated.
Swierniak, W.	2020	Personal Music Players Use and Other Noise Hazards among Children 11 to 12 Years Old	Gestational age of study population was not sufficiently stated.
Swift, V. M.	2020	Djaalinj Waakinj (listening talking): Rationale, cultural governance, methods, population characteristics - an urban Aboriginal birth cohort study of otitis media	Connection of risk factors and/or hearing outcome depending on gestational age was not established.
Swigonski, N.	1987	Hearing screening of high risk newborns	No comparison of hearing impairment between preterm and full-term born infants.
Swinnen, F. K.	2011	Osteogenesis Imperfecta: the audiological phenotype lacks correlation with the genotype	Gestational age of study population was not sufficiently stated.
Synmon, B.	2017	Clinical and radiological spectrum of intracranial tuberculosis: A hospital based study in Northeast India	Gestational age of study population was not sufficiently stated.
Synnes, A.	2017	Determinants of developmental outcomes in a very preterm Canadian cohort	No comparison of hearing impairment between preterm and full-term born infants.
Synnes, A. R.	2012	Incidence and pattern of hearing impairment in children with ‚â§ 800 g birthweight in British Columbia, Canada	No comparison of hearing impairment between preterm and full-term born infants.
Syrogiannopoulos, G. A.	1994	Dexamethasone therapy for bacterial meningitis in children: 2- versus 4-day regimen	Gestational age of study population was not sufficiently stated.
Szabo, C.	2010	Treatment of persistent middle ear effusion in cleft palate patients	Gestational age of study population was not sufficiently stated.
Szczaluba, K.	2017	Isolated Hearing Impairment Caused by SPATA5 Mutations in a Family with Variable Phenotypic Expression	Case report as unsuitable study type.
Tabrizi, A. G.	2017	BIRTH BY CESAREAN DELIVERY ON NEWBORN HEARING SCREENING TEST: A RETROSPECTIVE STUDY	No comparison of hearing impairment between preterm and full-term born infants.
Tagawa, M.	2009	Retrospective Diagnosis of Congenital Cytomegalovirus Infection at a School for the Deaf by Using Preserved Dried Umbilical Cord	Missing gestational age of control collective.
Taghdiri, M. M.	2008	Auditory Evaluation of High Risk Newborns by Automated Auditory Brain Stem Response	Gestational age of study population was not sufficiently stated.
Taha, A. A.	2010	Prevalence and risk factors of hearing impairment among primary-school children in Shebin El-kom District, Egypt	Only investigation of prematurity as risk factor for hearing impairment, other risk factors not analyzed comparing preterm and full-term born infants.
Taiji, H.	2012	[Clinical features associated with sudden hearing loss in children]	No comparison of hearing impairment between preterm and full-term born infants.
Taipale, A.	2012	Hearing loss in Angolan children with sickle-cell disease	Gestational age of study population was not sufficiently stated.
Taipale, A.	2011	Chronic suppurative otitis media in children of Luanda, Angola	Gestational age of study population was not sufficiently stated.
Taipale, A.	2011	Otorhinolaryngological findings and hearing in HIV-positive and HIV-negative children in a developing country	Gestational age of study population was not sufficiently stated.
Takagi, A.	2022	Incidence of Mumps Deafness in Japan, 2005-2017: Analysis of Japanese Insurance Claims Database	Gestational age of study population was not sufficiently stated.
Takahashi, R.	2007	Severe postnatal cytomegalovirus infection in a very premature infant	Case report as unsuitable study type.
Takechi, K.	1999	[A case of keratitis, ichthyosis, and deafness syndrome with Hutchinson's triad-like symptoms]	Case report as unsuitable study type.
Takemoto, K.	2021	Outcomes in symptomatic preterm infants with postnatal cytomegalovirus infection	Hearing outcome not examined separately.
Talaat, H. S.	2014	Dose passive smoking induce sensorineural hearing loss in children?	Gestational age of study population was not sufficiently stated.
Talero-Gutierrez, C.	2011	Epidemiology of prelingual sensorineural hearing impairment at a children's center in Bogota, Colombia between 1997 and 2008	Only investigation of gestational age as risk factor for hearing impairment, other risk factors not analyzed comparing preterm and full-term born infants.
Tamrat, G.	2001	The prevalence and characteristics of physical and sensory disabilities in Northern Ethiopia	Gestational age of study population was not sufficiently stated.
Tan, S. S.	2016	Factors contributing to the longitudinal development of social participation in individuals with cerebral palsy	Gestational age of study population was not sufficiently stated.

Tan, V. Y.	2012	Acoustic brainstem implant in a post-meningitis deafened child-Lessons learned	Case report as unsuitable study type.
Tanaka-Kitajima, N.	2005	Ganciclovir therapy for congenital cytomegalovirus infection in six infants	No comparison of hearing impairment between preterm and full-term born infants.
Tanaka, C.	1996	Sensorineural deafness in siblings with adenosine deaminase deficiency	Case report as unsuitable study type.
Tanaka, Y.	2016	[Newborn Hearing Screening and Subsequent Diagnostic Evaluation: Analysis and Outcomes of 6,063 Infants Born in a Community Hospital]	No comparison of hearing impairment between preterm and full-term born infants.
Tang, F.	2017	Novel compound heterozygous mutations in the OTOF Gene identified by whole-exome sequencing in auditory neuropathy spectrum disorder	Gestational age of study population was not sufficiently stated.
Tang, K.	2019	Screening of mitochondrial tRNA mutations in 300 infants with hearing loss	Gestational age of study population was not sufficiently stated.
Tang, S. F.	1996	Low-dose inhaled nitric oxide for neonates with pulmonary hypertension	No comparison of hearing impairment between preterm and full-term born infants.
Tang, Y. L.	2018	[A family with Allan-Herndon-Dudley syndrome due to SLC16A2 gene mutation]	Case report as unsuitable study type.
Tanphaichitr, A.	2014	Incidence of ototoxicity in pediatric patients with transfusion-dependent thalassemia who are less well-chelated by mono- and combined therapy of iron chelating agents	Gestational age of study population was not sufficiently stated.
Tarailo-Graovac, M.	2015	The genotypic and phenotypic spectrum of PIGA deficiency	Case report as unsuitable study type.
Tarkkanen, J.	1966	Unilateral deafness in children	Gestational age of study population was not sufficiently stated.
Tarshish, Y.	2016	Risk Factors for Hearing Loss in Patients with Cystic Fibrosis	Gestational age of study population was not sufficiently stated.
Tasci, Y.	2010	Newborn hearing screening programme outcomes in a research hospital from Turkey	Connection of risk factors and/or hearing outcome depending on gestational age was not established.
Tassiopoulos, K.	2016	Following young people with perinatal HIV infection from adolescence into adulthood: the protocol for PHACS AMP Up, a prospective cohort study	Gestational age of study population was not sufficiently stated.
Tatli, B.	2005	Not a new leukodystrophy but congenital cytomegalovirus infection	Case report as unsuitable study type.
Tatli, M. M.	2007	Feasibility of neonatal hearing screening program with two-stage transient otoacoustic emissions in Turkey	Only investigation of prematurity as risk factor for hearing impairment, other risk factors not analyzed comparing preterm and full-term born infants.
Tavakol, M.	2014	Otological Findings in Pediatric Patients with Hypogammaglobulinemia	Gestational age of study population was not sufficiently stated.
Taylor, H. G.	1990	The sequelae of Haemophilus influenzae meningitis in school-age children	Gestational age of study population was not sufficiently stated.
Taylor, H. G.	1998	Acute-phase neurologic complications of Haemophilus influenzae type b meningitis: association with developmental problems at school age	Gestational age of study population was not sufficiently stated.
Taylor, M. G.	2021	Two Cases of Cronobacter Sakazakii Meningitis in Infants: The Importance Of Early Advanced Brain Imaging and Public Health Reporting	Case report as unsuitable study type.
Taylor, M. G.	2022	Plasma Metagenomic Sequencing Expedites Diagnosis of Disseminated BCG in an Infant With IKBKB Mutation	Case report as unsuitable study type.
Tedeschi, A. S.	2015	The Prevalence of Congenital Hearing Loss in Neonates with Down Syndrome	Gestational age of study population was not sufficiently stated.
Teek, R.	2013	Hearing impairment in Estonia: An algorithm to investigate genetic causes in pediatric patients	Gestational age of study population was not sufficiently stated.
Teele, D. W.	1990	Acoustic reflectometry for assessment of hearing loss in children with middle ear effusion	Gestational age of study population was not sufficiently stated.
Teissier, N.	2013	[Audiophonological evaluation of 16 children fitted with cochlear implants for sensorineural hearing loss induced by bacterial meningitis]	Gestational age of study population was not sufficiently stated.
Teixeira, D. C.	2021	Risk Factors for Severe Outcomes in Bacterial Meningitis	Gestational age of study population was not sufficiently stated.
Teke, T. A.	2015	Neurobrucellosis in children: Case series from Turkey	Gestational age of study population was not sufficiently stated.
Tekin, M.	2000	Fluorescence in situ hybridization detectable mosaicism for Angelman syndrome with biparental methylation	Case report as unsuitable study type.
Ten Dam, E.	2013	Age of diagnosis and evaluation of consequences of submucous cleft palate	Gestational age of study population was not sufficiently stated.

Ten Hove, C. H.	2016	Long-Term Neurodevelopmental Outcome after Doxapram for Apnea of Prematurity	Hearing outcome not examined separately.
Teo, D. T.	2004	Spontaneous cerebrospinal fluid otorrhoea via oval window: an obscure cause of recurrent meningitis	Case report as unsuitable study type.
Ter Meulen, J.	1996	Hunting of peridomestic rodents and consumption of their meat as possible risk factors for rodent-to-human transmission of Lassa virus in the Republic of Guinea	Gestational age of study population was not sufficiently stated.
Thakkar, D.	2018	Brainstem-Evoked Response Audiometry in Pediatric Age Group	Gestational age of study population was not sufficiently stated.
Thangavelu, K.	2019	Prevalence and risk factors for hearing loss in high-risk neonates in Germany	Connection of risk factors and/or hearing outcome depending on gestational age was not established.
The Victorian Infant Collaborative Study Group	1996	Surgery and the tiny baby: sensorineural outcome at 5 years of age. The Victorian Infant Collaborative Study Group	No comparison of hearing impairment between preterm and full-term born infants.
The Victorian Infant Collaborative Study Group	2000	Postnatal corticosteroids and sensorineural outcome at 5 years of age	No comparison of hearing impairment between preterm and full-term born infants.
Theodoridou, K.	2013	Association of treatment for bacterial meningitis with the development of sequelae	Gestational age of study population was not sufficiently stated.
Thibault, M.	2011	Genetic syndromes that mimic congenital infections: Report of 2 cases	Case report as unsuitable study type.
Thiringer, K.	1984	Perinatal risk factors in the aetiology of hearing loss in preschool children	Gestational age of study population was not sufficiently stated.
Thirunavukkarasu, K. and Geetha, C.	2015	One-year prevalence and risk factors of tinnitus in children with otological problems	Gestational age of study population was not sufficiently stated.
Thomas, D. G.	1992	Outcome of paediatric bacterial meningitis 1979-1989	Gestational age of study population was not sufficiently stated.
Thompson, C. S.	2020	Sudden onset hearing loss following intra-abdominal surgery: an unusual association	Case report as unsuitable study type.
Thompson, P. W.	2017	Mandibular Osteomyelitis and Cervical Lymphadenitis Due to Mycobacterium abscessus: Surgical Management of a Pediatric Cohort With a Shared Epidemiologic Exposure	Gestational age of study population was not sufficiently stated.
Thomsen, J.	1979	High dosage tobramycin treatment of children with cystic fibrosis. Bacteriological effect and clinical ototoxicity	Gestational age of study population was not sufficiently stated.
Thomson, I. S.	1976	A prospective study of children "at risk" for deafness	No comparison of hearing impairment between preterm and full-term born infants.
Thong, Y. H.	1978	Abnormal neutrophil chemotaxis in a syndrome of unusual facies, proportionate small stature and sensorineural deafness-mutism	Case report as unsuitable study type.
Thorne, J. A.	2003	Middle ear problems in Aboriginal school children cause developmental and educational concerns	Gestational age of study population was not sufficiently stated.
Thornton, A. R.	1993	External- and middle-ear factors affecting evoked otoacoustic emissions in neonates	Gestational age of study population was not sufficiently stated.
Tian, T.	2018	Identification of a novel MYO6 mutation associated with autosomal dominant non-syndromic hearing loss in a Chinese family by whole-exome sequencing	Gestational age of study population was not sufficiently stated.
Tian, Y.	2021	Increased diagnosis of enlarged vestibular aqueduct by multiplex PCR enrichment and next-generation sequencing of the SLC26A4 gene	Gestational age of study population was not sufficiently stated.
Tiedt, N. J.	2013	Paediatric chronic suppurative otitis media in the Free State Province: Clinical and audiological features	Gestational age of study population was not sufficiently stated.
Tiensoli, L. O.	2007	[Hearing screening in a public hospital in Belo Horizonte, Minas Gerais State, Brazil: hearing impairment and risk factors in neonates and infants]	No comparison of hearing impairment between preterm and full-term born infants.
Tieri, L.	1984	Sudden deafness in children	Gestational age of study population was not sufficiently stated.
Tissera, K. A.	2022	Hearing Stability in Patients With Unilateral Hearing Loss Due to Congenital CMV	Gestational age of study population was not sufficiently stated.
Tobaiqy, M.	2011	Parental reporting of adverse drug reactions associated with attention-deficit hyperactivity disorder (ADHD) medications in children attending specialist paediatric clinics in the UK	Gestational age of study population was not sufficiently stated.
Tobiansky, R.	1995	Neurodevelopmental outcome in very low birthweight infants with necrotizing enterocolitis requiring surgery	No comparison of hearing impairment between preterm and full-term born infants.
Toivonen, J.	2021	Facial Paralysis From Post-transplant Lymphoproliferative Disorder	Case report as unsuitable study type.

Toizumi, M.	2019	Characteristics of Patent Ductus Arteriosus in Congenital Rubella Syndrome	Connection of risk factors and/or hearing outcome depending on gestational age was not established.
Toizumi, M.	2014	Mortality associated with pulmonary hypertension in congenital rubella syndrome	Connection of risk factors and/or hearing outcome depending on gestational age was not established.
Toizumi, M.	2017	Sensory defects and developmental delay among children with congenital rubella syndrome	Gestational age of study population was not sufficiently stated.
Tokat, T.	2018	Cochlear Implantation in Postmeningitic Deafness	Gestational age of study population was not sufficiently stated.
Tollenaar, L. S. A.	2021	Twin Anemia Polycythemia Sequence: Knowledge and Insights After 15 Years of Research	Review, sources screened for suitable literature for review question.
Tommiska, V.	2003	A national two year follow up study of extremely low birthweight infants born in 1996-1997	Connection of risk factors and/or hearing outcome depending on gestational age was not established.
Tonekaboni, S. H.	2009	Neurobrucellosis: a partially treatable cause of vision loss	Case report as unsuitable study type.
Tong, M. C.	2006	Risk factors for otitis media with effusion in Chinese schoolchildren: a nested case-control study and review of the literature	Hearing outcome not examined.
Toome, L.	2013	Follow-up study of 2-year-olds born at very low gestational age in Estonia	No comparison of hearing impairment between preterm and full-term born infants.
Toral-Martinon, R.	2003	[Effects of Cisplatin on Auditory Function in Children with cancer. Otoacoustic Emission Evaluation]	No comparison of hearing impairment between preterm and full-term born infants.
Torii, Y.	2019	Serological screening of immunoglobulin M and immunoglobulin G during pregnancy for predicting congenital cytomegalovirus infection	Hearing outcome not examined.
Torre, P.	2015	Hearing assessment data in HIV-infected and uninfected children of Cape Town, South Africa	Gestational age of study population was not sufficiently stated.
Torre, P.	2012	Hearing Loss in Perinatally HIV-infected and HIV-exposed but Uninfected Children and Adolescents	Only investigation of prematurity as risk factor for hearing impairment, other risk factors not analyzed comparing preterm and full-term born infants.
Torre, P. R.	2016	Newborn Hearing Screenings in Human Immunodeficiency Virus-Exposed Uninfected Infants	Only investigation of prematurity as risk factor for hearing impairment, other risk factors not analyzed comparing preterm and full-term born infants.
Torrecillas, V.	2020	Should You Follow the Better-Hearing Ear for Congenital Cytomegalovirus Infection and Isolated Sensorineural Hearing Loss?	Gestational age of study population was not sufficiently stated.
Torretta, S.	2016	Topical administration of hyaluronic acid in children with recurrent or chronic middle ear inflammations	Gestational age of study population was not sufficiently stated.
Torretta, S.	2018	Phenotype Profiling and Allergy in Otitis-Prone Children	Connection of risk factors and/or hearing outcome depending on gestational age was not established.
Torrico, P.	2004	[Age influence in otoacoustic emissions for hearing loss screening in infants]	No comparison of hearing impairment between preterm and full-term born infants.
Tos, M.	2000	Cochlear implantation of Danish prelingually deaf children	Gestational age of study population was not sufficiently stated.
Toumpas, C. J.	2015	Congenital cytomegalovirus infection is a significant cause of moderate to profound sensorineural hearing loss in Queensland children	Gestational age of study population was not sufficiently stated.
Townsend, C. L.	2013	Long-term Outcomes of Congenital Cytomegalovirus Infection in Sweden and the United Kingdom	Connection of risk factors and/or hearing outcome depending on gestational age was not established.
Townsend, C. L.	2011	Surveillance of congenital cytomegalovirus in the UK and Ireland	Connection of risk factors and/or hearing outcome depending on gestational age was not established.
Trahair, T. N.	2007	Long-term outcomes in children with high-risk neuroblastoma treated with autologous stem cell transplantation	Gestational age of study population was not sufficiently stated.
Trammer, R. M.	1992	Narcotic and nicotine effects on the neonatal auditory system	Connection of risk factors and/or hearing outcome depending on gestational age was not established.
Trevisan, C. P.	2008	Facioscapulohumeral muscular dystrophy: A multicenter study on hearing function	Gestational age of study population was not sufficiently stated.
Trinidad Ruiz, G.	2003	[Early detection of hearing loss. Example of intervention in public health]	No comparison of hearing impairment between preterm and full-term born infants.
Triono, A.	2018	Congenital Rubella Syndrome Screening for Newborn in Yogyakarta, Indonesia	Only investigation of gestational age as risk factor for hearing impairment, other risk factors not analyzed comparing preterm and full-term born infants.
Triono, A.	2021	Potentially predictive factors for hearing function improvement in pediatric cytomegalovirus infection therapy	Only investigation of prematurity as risk factor for hearing impairment, other risk factors not analyzed comparing preterm and full-term born infants.
Trotman, H.	2009	Pneumococcal Meningitis in Jamaican Children	Gestational age of study population was not sufficiently stated.

Trozzi, M.	2015	Cochlear re-implant rates in children: 20 years experience in a quaternary paediatric cochlear implant centre	Gestational age of study population was not sufficiently stated.
Tsige, S.	2021	Cerebral palsy in children: subtypes, motor function and associated impairments in Addis Ababa, Ethiopia	Gestational age of study population was not sufficiently stated.
Tsubota, M.	2008	Mumps virus may damage the vestibular nerve as well as the inner ear	Case report as unsuitable study type.
Tucci, D. L.	1992	Primary lymphoma of the temporal bone	Case report as unsuitable study type.
Tudehope, D.	1995	Changing patterns of survival and outcome at 4 years of children who weighted 500-999 g at birth	No comparison of hearing impairment between preterm and full-term born infants.
Tudehope, D.	1992	AUDIOLOGICAL EVALUATION OF VERY-LOW-BIRTH-WEIGHT INFANTS	No comparison of hearing impairment between preterm and full-term born infants.
Tufatulin, G. S.	2021	[Epidemiological study of hearing impairments in children: prevalence, structure, amplification, and social factors]	No comparison of hearing impairment between preterm and full-term born infants.
Tungvachirakul, V.	2011	Newborn hearing screening at Rajavithi Hospital, Thailand: hearing loss in infants not admitting in intensive care unit	Missing control collective without hearing impairment.
Tuppurainen, K.	1988	The KID-syndrome in Finland. A report of four cases	Case report as unsuitable study type.
Turan, C.	2019	Cisplatin ototoxicity in children: risk factors and its relationship with polymorphisms of DNA repair genes ERCC1, ERCC2, and XRCC1	Gestational age of study population was not sufficiently stated.
Turchetta, R.	2012	Modifications of auditory brainstem responses (ABR): observations in full-term and pre-term newborns	No investigation of risk factors for hearing impairment.
Turner, K. M.	2014	Incidence and Impact of CMV Infection in Very Low Birth Weight Infants	No comparison of hearing impairment between preterm and full-term born infants.
Twardella, D.	2011	Hearing loss in adolescents due to leisure noise. The OHRKAN study	Gestational age of study population was not sufficiently stated.
Tweed, E. J.	2016	Five-minute Apgar score and educational outcomes: retrospective cohort study of 751 369 children	No comparison of hearing impairment between preterm and full-term born infants.
Uchida, A.	2020	Clinical Factors Associated With Congenital Cytomegalovirus Infection: A Cohort Study of Pregnant Women and Newborns	Connection of risk factors and/or hearing outcome depending on gestational age was not established.
Uda, K.	2019	Ototoxicity and Nephrotoxicity With Elevated Serum Concentrations Following Vancomycin Overdose: A Retrospective Case Series	Gestational age of study population was not sufficiently stated.
Ueda, K.	1986	Incidence of congenital rubella syndrome in Japan (1965-1985). A nationwide survey of the number of deaf children with history of maternal rubella attending special schools for the deaf in Japan	Gestational age of study population was not sufficiently stated.
Uematsu, M.	2016	Asymptomatic congenital cytomegalovirus infection with neurological sequelae: A retrospective study using umbilical cord	No comparison of hearing impairment between preterm and full-term born infants.
Ullrich, D.	2002	[Might the cooperation with neuropediatricians improve the treatment of hearing impaired children: retrospective analysis of a small group of children with hearing aids]	Gestational age of study population was not sufficiently stated.
Ulrick, N.	2017	RMND1-Related Leukoencephalopathy With Temporal Lobe Cysts and Hearing Loss-Another Mendelian Mimicker of Congenital Cytomegalovirus Infection	Gestational age of study population was not sufficiently stated.
Ulusoy, S.	2014	The results of national newborn hearing screening (NNHS) data of 11,575 newborns from west part of Turkey	Gestational age of study population was not sufficiently stated.
Umehara, T.	2019	Risk Factors and Prognostic Factors of Hearing Impairment in Neonatal Intensive Care Unit-Treated Infants	Only investigation of gestational age as risk factor for hearing impairment, other risk factors not analyzed comparing preterm and full-term born infants.
Unal, M.	1998	Sudden total bilateral deafness due to asymptomatic mumps infection	Case report as unsuitable study type.
Underbjerg, M.	2012	The effects of low to moderate alcohol consumption and binge drinking in early pregnancy on selective and sustained attention in 5-year-old children	Hearing outcome not examined separately.
Unhanand, M.	1993	Gram-negative enteric bacillary meningitis: a twenty-one-year experience	Only investigation of prematurity as risk factor for hearing impairment, other risk factors not analyzed comparing preterm and full-term born infants.
Unlu, I.	2015	When should automatic auditory brainstem response test be used for newborn hearing screening?	No comparison of hearing impairment between preterm and full-term born infants.
Upadhyay, K.		Outcome of Universal Neonatal Hearing Screening Programme at a Tertiary Care Centre: A Prospective Study	Connection of risk factors and/or hearing outcome depending on gestational age was not established.

Upfold, L. J.	1970	Deafness following rubella in pregnancy	Gestational age of study population was not sufficiently stated.
Upfold, L. J.	1988	Children with hearing aids in the 1980s: etiologies and severity of impairment	Only investigation of prematurity as risk factor for hearing impairment, other risk factors not analyzed comparing preterm and full-term born infants.
Upfold, L. J.	1982	Childhood deafness in Australia. Incidence and maternal rubella, 1949-1980	Gestational age of study population was not sufficiently stated.
Uppal, P.	2014	Refractory Otitis Media: An Unusual Presentation of Childhood Granulomatosis With Polyangiitis	Case report as unsuitable study type.
Usami, S. I.	2017	Etiology of single-sided deafness and asymmetrical hearing loss	Gestational age of study population was not sufficiently stated.
Uus, K.	2006	Effectiveness of population-based newborn hearing screening in England: Ages of interventions and profile of cases	Gestational age of study population was not sufficiently stated.
Uwayezu, D.	2020	Prevalence of dental caries and associated risk factors in children living with disabilities in Rwanda: a cross-sectional study	Hearing outcome not examined separately.
Uysal, I. ñ.	2012	Evaluation of cochlear function using transient evoked otoacoustic emission in children with Familial Mediterranean Fever	Gestational age of study population was not sufficiently stated.
Valdes, R. H.	2020	Chronic interstitial nephritis of nontraditional causes in Salvadoran agricultural communities	Gestational age of study population was not sufficiently stated.
Valeriani, V.	2022	Hearing impairment in children living with HIV in Haiti	Gestational age of study population was not sufficiently stated.
Valkama, A. M.	2000	Prediction of permanent hearing loss in high-risk preterm infants at term age	Only investigation of prematurity as risk factor for hearing impairment, other risk factors not analyzed comparing preterm and full-term born infants.
Vallino-Napoli, L. D.	1996	Audiologic and otologic characteristics of Pfeiffer syndrome	Gestational age of study population was not sufficiently stated.
Van Bang, N.	2014	Surveillance of congenital rubella syndrome (CRS) in tertiary care hospitals in Hanoi, Vietnam during a rubella epidemic	Connection of risk factors and/or hearing outcome depending on gestational age was not established.
Van Bergen, N. J.	2020	Deficiencies in vesicular transport mediated by TRAPPC4 are associated with severe syndromic intellectual disability	Gestational age of study population was not sufficiently stated.
van den Hondel, D.	2013	Sensorineural Hearing Loss and Language Development Following Neonatal Extracorporeal Membrane Oxygenation	No comparison of hearing impairment between preterm and full-term born infants.
van Dongen, H. R.	1993	Blind, deaf and mute after a status epilepticus caused by hyperpyrexia from shigellosis--a case report with a four-year follow-up	Case report as unsuitable study type.
Van Houtte, E.	2014	Middle and inner ear malformations in two siblings exposed to valproic acid during pregnancy: A case report	Case report as unsuitable study type.
van Kerschaver, E.	2013	Universal neonatal hearing screening in Flanders reveals socio-demographic risk factors for hearing impairment	No comparison of hearing impairment between preterm and full-term born infants.
Van Kerschaver, E.	2013	Socio-demographic determinants of hearing impairment studied in 103 835 term babies	No comparison of hearing impairment between preterm and full-term born infants.
van Manen, M.	2013	Early childhood outcomes of infants born with gastroschisis	Connection of risk factors and/or hearing outcome depending on gestational age was not established.
van Naarden, K.	1999	Relative and attributable risks for moderate to profound bilateral sensorineural hearing impairment associated with lower birth weight in children 3 to 10 years old	Preterm infants with risk factor compared to full-term born infants without risk factor.
van Noort-van der Spek, I. L.	2017	Normal neonatal hearing screening did not preclude sensorineural hearing loss in two-year-old very preterm infants	No comparison of hearing impairment between preterm and full-term born infants.
Van Riper, L. A.	1999	ABR hearing screening for high-risk infants	Gestational age of study population was not sufficiently stated.
van Straaten, H. L.	1996	Evaluation of an automated auditory brainstem response infant hearing screening method in at risk neonates	Gestational age of study population was not sufficiently stated.
van Straaten, H. L. M.	2003	Implementation of a nation-wide automated auditory brainstem response hearing screening programme in neonatal intensive care units	Gestational age of study population was not sufficiently stated.
van Well, G. T. J.	2012	Polymorphisms in Toll-Like Receptors 2, 4, and 9 Are Highly Associated with Hearing Loss in Survivors of Bacterial Meningitis	Gestational age of study population was not sufficiently stated.
van Zwol, A.	2008	Neurodevelopmental outcomes of very low-birth-weight infants after enteral glutamine supplementation in the neonatal period	Hearing outcome not examined separately.
Vancor, E.	2019	Results of a Targeted Screening Program for Congenital Cytomegalovirus Infection in Infants Who Fail Newborn Hearing Screening	No comparison of hearing impairment between preterm and full-term born infants.

Vargas-Poussou, R.	2006	Genetic investigation of autosomal recessive distal renal tubular acidosis: evidence for early sensorineural hearing loss associated with mutations in the ATP6V0A4 gene	Gestational age of study population was not sufficiently stated.
Vartiainen, E.	1993	Fate of patients with bilateral cholesteatoma	Gestational age of study population was not sufficiently stated.
Vashistha, I.	2016	Prevalence of Hearing Impairment in High Risk Infants	Only investigation of prematurity as risk factor for hearing impairment, other risk factors not analyzed comparing preterm and full-term born infants.
Vasilopoulou, V. A.	2011	Prognostic factors related to sequelae in childhood bacterial meningitis: Data from a Greek meningitis registry	Gestational age of study population was not sufficiently stated.
Vaswani, N. D.	2021	Seven versus Ten Days Antibiotics Course for Acute Pyogenic Meningitis in Children: A Randomized Controlled Trial	Gestational age of study population was not sufficiently stated.
Vatovec, J.	2001	Otoacoustic emissions and auditory assessment in infants at risk for early brain damage	Gestational age of study population was not sufficiently stated.
Vedovato, S.	2015	Sensorineural hearing loss in very low birth weight infants with histological chorioamnionitis	No comparison of hearing impairment between preterm and full-term born infants.
Veen, S.	1993	Hearing loss in very preterm and very low birthweight infants at the age of 5 years in a nationwide cohort	No comparison of hearing impairment between preterm and full-term born infants.
Vejtorp, M.	1980	Rubella IgM antibodies in sera from infants born after maternal rubella later than the 12th week of pregnancy	Gestational age of study population was not sufficiently stated.
Velepic, M. M.	2004	Gastroesophageal reflux and sequelae of chronic tubotympanal disorders in children	Gestational age of study population was not sufficiently stated.
Vella-Brincat, J. W.	2011	Are gentamicin and/or vancomycin associated with ototoxicity in the neonate? A retrospective audit	Only investigation of gestational age as risk factor for hearing impairment, other risk factors not analyzed comparing preterm and full-term born infants.
Venkateswaran, S.	2008	Comorbidities and clinical determinants of outcome in children with spastic quadriplegic cerebral palsy	Only investigation of prematurity as risk factor for hearing impairment, other risk factors not analyzed comparing preterm and full-term born infants.
Ventriculomegaly Trial Group	1994	Randomised trial of early tapping in neonatal posthaemorrhagic ventricular dilatation: results at 30 months. Ventriculomegaly Trial Group	Gestational age of study population was not sufficiently stated.
Verbeeck, J.	2008	Detection of Perinatal Cytomegalovirus Infection and Sensorineural Hearing Loss in Belgian Infants by Measurement of Automated Auditory Brainstem Response	Gestational age of study population was not sufficiently stated.
Verduin, E. P.	2010	Long-Term follow up after intra-Uterine transfusionS; the LOTUS study	Study protocol as unsuitable study type.
Verhaeghe, C.	2022	Neurodevelopment at 5 years of age for preterm-born children according to mode of conception: a cohort study	No comparison of hearing impairment between preterm and full-term born infants.
Verjan-Carrillo, E. J.	2021	Factors associated with increased odds of sensorineural hearing loss in infants exposed to the Zika virus during pregnancy	No comparison of hearing impairment between preterm and full-term born infants.
Verma, A. K.	1995	Epidemiology of chronic suppurative otitis media and deafness in a rural area and developing an intervention strategy	Gestational age of study population was not sufficiently stated.
Vidgoff, J.	1977	Mannosidosis in three brothers--a review of the literature	Case report as unsuitable study type.
Vieira, W. A.	2014	Ototoxicity evaluation in medulloblastoma patients treated with involved field boost using intensity-modulated radiation therapy (IMRT): a retrospective review	Gestational age of study population was not sufficiently stated.
Vienny, H.	1984	Early diagnosis and evolution of deafness in childhood bacterial meningitis: a study using brainstem auditory evoked potentials	Connection of risk factors and/or hearing outcome depending on gestational age was not established.
Vijayalakshmi, P.	2003	Visual outcome of cataract surgery in children with congenital rubella syndrome	No comparison of hearing impairment between preterm and full-term born infants.
Vila, P. M.	2017	Infectious complications of pediatric cochlear implants are highly influenced by otitis media	Gestational age of study population was not sufficiently stated.
Viljoen, D. L.	1988	Childhood deafness in Zimbabwe	Gestational age of study population was not sufficiently stated.
Viljoen, D. L.	1983	Familial aggregation of streptomycin ototoxicity: autosomal dominant inheritance?	Gestational age of study population was not sufficiently stated.
Vince, D. J.	1970	The hospital incidence and clinical significance of congenital heart malformations resulting from rubella embryopathy	Gestational age of study population was not sufficiently stated.
Viner, R. M.	2012	Outcomes of invasive meningococcal serogroup B disease in children and adolescents (MOSAIC): a case-control study	Gestational age of study population was not sufficiently stated.

Vinnars, M. T.	2015	Association between cerebral palsy and microscopically verified placental infarction in extremely preterm infants	No comparison of hearing impairment between preterm and full-term born infants.
Visentin, S.	2012	Early Primary Cytomegalovirus Infection in Pregnancy: Maternal Hyperimmunoglobulin Therapy Improves Outcomes Among Infants at 1 Year of Age	Gestational age of study population was not sufficiently stated.
Visnjar, T.	2022	Biallelic ATOH1 Gene Variant in Siblings With Pontocerebellar Hypoplasia, Developmental Delay, and Hearing Loss	Connection of risk factors and/or hearing outcome depending on gestational age was not established.
Vital, I.	2015	Distortion-product otoacoustic emissions testing in neonates treated with an aminoglycoside in a neonatal intensive care unit	Only investigation of prematurity as risk factor for hearing impairment, other risk factors not analyzed comparing preterm and full-term born infants.
Vitanza, N. A.	2016	Noncarboplatin-induced Sensorineural Hearing Loss in a Patient With an Intracranial Nongerminomatous Germ Cell Tumor	Gestational age of study population was not sufficiently stated.
Vohr, B. R.	2006	Beneficial effects of breast milk in the neonatal intensive care unit on the developmental outcome of extremely low birth weight infants at 18 months of age	No comparison of hearing impairment between preterm and full-term born infants.
Vohr, B. R.	2000	Identification of neonatal hearing impairment: Characteristics of infants in the neonatal intensive care unit and well-baby nursery	Connection of risk factors and/or hearing outcome depending on gestational age was not established.
Vohr, B. R.	2000	Neurodevelopmental and functional outcomes of extremely low birth weight infants in the National Institute of Child Health and Human Development Neonatal Research Network, 1993-1994	No comparison of hearing impairment between preterm and full-term born infants.
Vollmer, B.	2004	Postnatally acquired cytomegalovirus infection via breast milk: effects on hearing and development in preterm infants	No comparison of hearing impairment between preterm and full-term born infants.
Vomero, A.	2019	Malignant Infantile osteopetrosis	Case report as unsuitable study type.
Vos, H. I.	2016	Replication of a genetic variant in ACYP2 associated with cisplatin-induced hearing loss in patients with osteosarcoma	Gestational age of study population was not sufficiently stated.
Voss, S. S.	2022	Risk of sequelae after invasive meningococcal disease	Gestational age of study population was not sufficiently stated.
Wadhawan, R.	2009	Twin Gestation and Neurodevelopmental Outcome in Extremely Low Birth Weight Infants	No comparison of hearing impairment between preterm and full-term born infants.
Waissbluth, S.	2018	Incidence and associated risk factors for platinum-induced ototoxicity in pediatric patients	Gestational age of study population was not sufficiently stated.
Wake, M.	2016	Population Outcomes of Three Approaches to Detection of Congenital Hearing Loss	Gestational age of study population was not sufficiently stated.
Walch, C.	2000	Bilateral sensorineural hearing disorders in children: etiology of deafness and evaluation of hearing tests	Gestational age of study population was not sufficiently stated.
Wald, E. R.	1995	Dexamethasone therapy for children with bacterial meningitis. Meningitis Study Group	Gestational age of study population was not sufficiently stated.
Walsh, M. C.	2010	Two-year neurodevelopmental outcomes of ventilated preterm infants treated with inhaled nitric oxide	No comparison of hearing impairment between preterm and full-term born infants.
Walsh, M. C.	2005	Extremely low birthweight neonates with protracted ventilation: mortality and 18-month neurodevelopmental outcomes	No comparison of hearing impairment between preterm and full-term born infants.
Walsh, T.	2006	Genomic analysis of a heterogeneous Mendelian phenotype: multiple novel alleles for inherited hearing loss in the Palestinian population	Gestational age of study population was not sufficiently stated.
Walter, S.	2008	Congenital cytomegalovirus: association between dried blood spot viral load and hearing loss	Gestational age of study population was not sufficiently stated.
Wändell, P.	2021	Hearing impairment among children in Sweden with foreign-born parents and natives: A national Swedish study	Gestational age of study population was not sufficiently stated.
Wang, C. H.	2017	Prevalence and independent risk factors for hearing impairment among very low birth weight infants	No comparison of hearing impairment between preterm and full-term born infants.
Wang, H.	2021	Usher syndrome type 2A complicated with glycogen storage disease type 3 due to paternal uniparental isodisomy of chromosome 1 in a sporadic patient	Gestational age of study population was not sufficiently stated.
Wang, J.	2018	Cross-sectional epidemiology of hearing loss in Australian children aged 11-12 years old and 25-year secular trends	Gestational age of study population was not sufficiently stated.
Wang, J.	2022	Does inflammation mediate the association between obesity and hearing status in mid-childhood and mid-life?	Gestational age of study population was not sufficiently stated.
Wang, J.	2019	Inflammation and hearing status in mid-childhood and mid-life: a population-based cross-sectional study	Gestational age of study population was not sufficiently stated.
Wang, J.	2018	How body composition influences hearing status by mid-childhood and mid-life: The Longitudinal Study of Australian Children	Gestational age of study population was not sufficiently stated.

Wang, L.	2022	Identification of novel compound heterozygous mutations of the MYO15A gene with autosomal recessive non-syndromic hearing loss	Gestational age of study population was not sufficiently stated.
Wang, L.	2021	Novel compound heterozygous mutations of DNAH5 identified in a pediatric patient with Kartagener syndrome: case report and literature review	Case report as unsuitable study type.
Wang, L. A.	2018	Prolonged furosemide exposure and risk of abnormal newborn hearing screen in premature infants	No comparison of hearing impairment between preterm and full-term born infants.
Wang, Q. J.	2019	Nationwide population genetic screening improves outcomes of newborn screening for hearing loss in China	Gestational age of study population was not sufficiently stated.
Wang, Q. M.	2021	A NovelCREBBPin-Frame Deletion Variant in a Chinese Girl with Atypical Rubinstein-Taybi Syndrome Phenotypes	Case report as unsuitable study type.
Wang, R.	2002	Infants of diabetic mothers are at increased risk for the oculo-auriculo-vertebral sequence: A case-based and case-control approach	Case report as unsuitable study type.
Wang, S.	2022	De novo Splice Site Mutation of the CHD7 Gene in a Chinese Patient with Typical CHARGE Syndrome	Case report as unsuitable study type.
Wang, Y.	2017	Pathogenicity of two COQ7 mutations and responses to 2,4-dihydroxybenzoate bypass treatment	Case report as unsuitable study type.
Wang, Y. H.	2020	Cytomegalovirus enteritis with intractable diarrhea in infants from a tertiary care center in China	Gestational age of study population was not sufficiently stated.
Ward, J. D.	2022	The clinical impact of maternal COVID-19 on mothers, their infants, and placentas with an analysis of vertical transfer of maternal SARS-CoV-2-specific IgG antibodies	Connection of risk factors and/or hearing outcome depending on gestational age was not established.
Wasserman, E. E.	2012	Childhood IQ, hearing loss, and maternal thyroid autoimmunity in the Baltimore Collaborative Perinatal Project	Gestational age of study population was not sufficiently stated.
Waters, A.	2010	Human Cytomegalovirus UL144 Is Associated with Viremia and Infant Development Sequelae in Congenital Infection	Gestational age of study population was not sufficiently stated.
Watkin, P.	2012	Postneonatal care pathways and the identification of deafness	Gestational age of study population was not sufficiently stated.
Watkin, P. M.	1996	Outcomes of neonatal screening for hearing loss by otoacoustic emission	Gestational age of study population was not sufficiently stated.
Watkin, P. M.	1991	Neonatal at risk screening and the identification of deafness	Connection of risk factors and/or hearing outcome depending on gestational age was not established.
Waugh, J.	1996	Prevalence and aetiology of neurological impairment in extremely low birthweight infants	No comparison of hearing impairment between preterm and full-term born infants.
Weatherly, R. A.	1991	cis-platinum ototoxicity in children	Gestational age of study population was not sufficiently stated.
Weber, P. C.	1997	Congenital cholesteatomas in the tympanic membrane	Case report as unsuitable study type.
Wei, L.	2022	A Novel Missense Mutation in TWNK Gene Causing Perrault Syndrome Type 5 in a Chinese Family and Review of the Literature	Case report as unsuitable study type.
Wei, M.	2019	Cisplatin-induced Ototoxicity in Children With Solid Tumor	Gestational age of study population was not sufficiently stated.
Weichbold, V.	2006	Universal newborn hearing screening and postnatal hearing loss	Gestational age of study population was not sufficiently stated.
Weil, C.	2022	Health Care Resource Utilization and Economic Burden Associated With Congenital Cytomegalovirus Infection: A Longitudinal Analysis of Data From Clinical Practice at a Large Health Care Provider in Israel	Connection of risk factors and/or hearing outcome depending on gestational age was not established.
Weimer, K. E. D.	2020	Association of Adverse Hearing, Growth, and Discharge Age Outcomes With Postnatal Cytomegalovirus Infection in Infants With Very Low Birth Weight	No comparison of hearing impairment between preterm and full-term born infants.
Weisglas-Kuperus, N.	1993	Hearing and language in preschool very low birthweight children	No comparison of hearing impairment between preterm and full-term born infants.
Weiss, A.	2017	Long-term auditory complications after childhood cancer: A report from the Swiss Childhood Cancer Survivor Study	Gestational age of study population was not sufficiently stated.
Weiss, J. P.	2012	fMRI evaluation of cochlear implant candidacy in diffuse cortical cytomegalovirus disease	Case report as unsuitable study type.
Weissenstein, A.	2012	Progressive hearing loss after completion of cisplatin chemotherapy is common and more pronounced in children without spontaneous otoacoustic emissions before chemotherapy	Gestational age of study population was not sufficiently stated.

Weitzman, M.	2013	Maternal prenatal smoking and hearing loss among adolescents	Gestational age of study population was not sufficiently stated.
Welling, D. B.	2003	Predictive factors in pediatric stapedectomy	Gestational age of study population was not sufficiently stated.
Wellman, M. B.	2003	Sensorineural hearing loss in postmeningitic children	Gestational age of study population was not sufficiently stated.
Wells, E. M.	2018	Longitudinal assessment of late-onset neurologic conditions in survivors of childhood central nervous system tumors: a Childhood Cancer Survivor Study report	Gestational age of study population was not sufficiently stated.
Wells, M.	1982	Congenital abnormalities of the ear in perinatal deaths	Case report as unsuitable study type.
Welzel, T.	2022	Variant in the PLCG2 Gene May Cause a Phenotypic Overlap of APLAID/PLAID: Case Series and Literature Review	Case report as unsuitable study type.
Wen, J.	2021	Establishment of an iPSC line (CSUXHi004-A) from a patient with Waardenburg syndrome type I caused by a PAX3 splice mutation	Case report as unsuitable study type.
Wen, L. Z.	2002	Cytomegalovirus infection in pregnancy	Connection of risk factors and/or hearing outcome depending on gestational age was not established.
Wendt, W. J.	2019	Enlarged Vestibular Aqueduct Syndrome Sudden Hearing Loss in a Child with a Cerebral Shunt	Case report as unsuitable study type.
Wenzhi, H.	2015	Heterozygous deletion at the SOX10 gene locus in two patients from a Chinese family with Waardenburg syndrome type II	Gestational age of study population was not sufficiently stated.
Werler, M. M.	2004	Demographic and reproductive factors associated with hemifacial microsomia	Gestational age of study population was not sufficiently stated.
Wertzner, H. F.	2007	Phonological performance measured by speech severity indices compared with correlated factors	Gestational age of study population was not sufficiently stated.
West, A. N.	2021	Identification of Perinatal Risk Factors for Auditory Neuropathy Spectrum Disorder	Only investigation of prematurity as risk factor for hearing impairment, other risk factors not analyzed comparing preterm and full-term born infants.
West, S. K.	2015	Electroretinogram assessment of children with sensorineural hearing loss: implications for screening	Gestational age of study population was not sufficiently stated.
Whatley, W. S.	2006	Systemic absorption of gentamicin nasal irrigations	Gestational age of study population was not sufficiently stated.
Whelan, K.	2011	Auditory complications in childhood cancer survivors: a report from the childhood cancer survivor study	Gestational age of study population was not sufficiently stated.
Whitelaw, A.	1994	RANDOMIZED TRIAL OF EARLY CAPPING IN NEONATAL POSTHEMORRHAGIC VENTRICULAR DILATATION - RESULTS AT 30 MONTHS	Connection of risk factors and/or hearing outcome depending on gestational age was not established.
Wickremasinghe, A. C.	2015	Risk of Sensorineural Hearing Loss and Bilirubin Exchange Transfusion Thresholds	Gestational age of study population does not meet defined inclusion criteria for prematurity (<37 weeks) and/or full-term birth (≥37 weeks) with cut-off stated at 37 weeks.
Wickremasinghe, A. C.	2012	Neurodevelopmental outcomes following two different treatment approaches (early ligation and selective ligation) for patent ductus arteriosus	No comparison of hearing impairment between preterm and full-term born infants.
Widen, J. E.	2000	Identification of neonatal hearing impairment: Hearing status at 8 to 12 months corrected age using a visual reinforcement audiometry protocol	Gestational age of study population was not sufficiently stated.
Widen, S. E.	2018	Headphone listening habits, hearing thresholds and listening levels in Swedish adolescents with severe to profound HL and adolescents with normal hearing	Gestational age of study population was not sufficiently stated.
Widziszowska, A.	2011	Assessment of hearing organ activity in a group of neonates with central nervous system impairment	No comparison of hearing impairment between preterm and full-term born infants.
Wien, M. A.	2017	The association among prematurity, cochlear hyperintensity, and hearing loss	No comparison of hearing impairment between preterm and full-term born infants.
Wiener-Vacher, S. R.	2012	Vestibular impairment after bacterial meningitis delays infant posturomotor development	Gestational age of study population was not sufficiently stated.
Wiener-Vacher, S. R.	2018	Epidemiology of Vestibular Impairments in a Pediatric Population	Gestational age of study population was not sufficiently stated.
Wild, N. J.	1989	Onset and severity of hearing loss due to congenital rubella infection	Gestational age of study population was not sufficiently stated.
Wild, N. J.	1990	Delayed detection of congenital hearing loss in high risk infants	Gestational age of study population was not sufficiently stated.
Wiley, S.	2006	GJB2 mutations and additional disabilities in a pediatric cochlear implant population	Gestational age of study population was not sufficiently stated.

Wilfert, C. M.	1981	Longitudinal assessment of children with enteroviral meningitis during the first three months of life	Gestational age of study population was not sufficiently stated.
Wilken, B.	1995	[Hearing disorders in children less than 16 months of age after bacterial meningitis with reference to cerebrospinal fluid elastase]	Gestational age of study population was not sufficiently stated.
Wilking, A. N.	2014	Central Nervous System Manifestations in Pediatric Patients With Influenza A H1N1 Infection During the 2009 Pandemic	Gestational age of study population was not sufficiently stated.
Williams, B. A.	1992	LIMB DEFORMITY AND METAPHYSEAL ABNORMALITIES IN THALASSEMIA MAJOR	Gestational age of study population was not sufficiently stated.
Williams, E. J.	2014	Feasibility and acceptability of targeted screening for congenital CMV-related hearing loss	Connection of risk factors and/or hearing outcome depending on gestational age was not established.
Williams, K. B.	2019	Homozygosity for a mutation affecting the catalytic domain of tyrosyl-tRNA synthetase (YARS) causes multisystem disease	Connection of risk factors and/or hearing outcome depending on gestational age was not established.
Williams, L. L.	1993	Persistently altered T cell immunity in high school students with the congenital rubella syndrome and profound hearing loss	Gestational age of study population was not sufficiently stated.
Williamson, I.	2009	A double-blind randomised placebo-controlled trial of topical intranasal corticosteroids in 4-to 11-year-old children with persistent bilateral otitis media with effusion in primary care	Gestational age of study population was not sufficiently stated.
Williamson, W. D.	1992	Progressive hearing loss in infants with asymptomatic congenital cytomegalovirus infection	Connection of risk factors and/or hearing outcome depending on gestational age was not established.
Williamson, W. D.	1982	Symptomatic congenital cytomegalovirus. Disorders of language, learning, and hearing	Gestational age of study population was not sufficiently stated.
Williamson, W. D.	1990	Asymptomatic congenital cytomegalovirus infection. Audiologic, neuroradiologic, and neurodevelopmental abnormalities during the first year	Connection of risk factors and/or hearing outcome depending on gestational age was not established.
Wilson, C. B.	1980	Development of adverse sequelae in children born with subclinical congenital Toxoplasma infection	Connection of risk factors and/or hearing outcome depending on gestational age was not established.
Wilson, M. G.	2013	Hearing loss in congenital diaphragmatic hernia (CDH) survivors: is it as prevalent as we think?	No comparison of hearing impairment between preterm and full-term born infants.
Wilson, N. W.	1990	Posttraumatic meningitis in adolescents and children	Gestational age of study population was not sufficiently stated.
Wilunda, C.	2018	Exposure to tobacco smoke prenatally and during infancy and risk of hearing impairment among children in Japan: A retrospective cohort study	No comparison of hearing impairment between preterm and full-term born infants.
Winkel, S.	1978	Possible effects of kanamycin and incubation in newborn children with low birth weight	Gestational age of study population was not sufficiently stated.
Winterstein, A. G.	2013	Sensorineural Hearing Loss Associated with Neomycin Eardrops and Nonintact Tympanic Membranes	Gestational age of study population was not sufficiently stated.
Wiseman, D. H.	2013	A novel syndrome of congenital sideroblastic anemia, B-cell immunodeficiency, periodic fevers, and developmental delay (SIFD)	Gestational age of study population was not sufficiently stated.
Wolff, A. B.	1987	Demographics of meningitis-induced hearing impairment: implications for immunization of children against Hemophilus influenzae type B	Gestational age of study population was not sufficiently stated.
Wolter, N. E.	2012	Non-accidental caustic ear injury: two cases of profound cochleo-vestibular loss and facial nerve injury	Case report as unsuitable study type.
Wong, L. Y.	2017	Otoacoustic Emissions in Rural Nicaragua: Cost Analysis and Implications for Newborn Hearing Screening	Only investigation of prematurity as risk factor for hearing impairment, other risk factors not analyzed comparing preterm and full-term born infants.
Wood, S.	1998	Anomalous screening outcomes from click-evoked otoacoustic emissions and auditory brainstem response tests	No comparison of hearing impairment between preterm and full-term born infants.
Wood, S. A.	2013	Effectiveness of targeted surveillance to identify moderate to profound permanent childhood hearing impairment in babies with risk factors who pass newborn screening	Gestational age of study population was not sufficiently stated.
Woodward, L. J.	2006	Neonatal MRI to predict neurodevelopmental outcomes in preterm infants	No comparison of hearing impairment between preterm and full-term born infants.
Woolfenden, S.	2019	Impact of social disadvantage on cerebral palsy severity	Connection of risk factors and/or hearing outcome depending on gestational age was not established.
Woolley, A. L.	1999	Risk factors for hearing loss from meningitis in children: the Children's Hospital experience	Gestational age of study population was not sufficiently stated.
Worsoe, L.	2010	Factors Associated with the Occurrence of Hearing Loss after Pneumococcal Meningitis	Gestational age of study population was not sufficiently stated.
Wortmann, S.	2006	Association of 3-methylglutaconic aciduria with sensori-neural deafness, encephalopathy, and Leigh-	Gestational age of study population was not sufficiently stated.

		like syndrome (MEGDEL association) in four patients with a disorder of the oxidative phosphorylation	
Wrobel, M. J.	2014	The risk factor profile of children covered by the Polish universal neonatal hearing screening program and its impact on hearing loss incidence	Only investigation of prematurity as risk factor for hearing impairment, other risk factors not analyzed comparing preterm and full-term born infants.
Wroblewska-Seniuk, K.	2005	The results of newborn hearing screening by means of transient evoked otoacoustic emissions	Only investigation of prematurity as risk factor for hearing impairment, other risk factors not analyzed comparing preterm and full-term born infants.
Wroblewska-Seniuk, K.	2018	Sensorineural and conductive hearing loss in infants diagnosed in the program of universal newborn hearing screening	Only investigation of gestational age as risk factor for hearing impairment, other risk factors not analyzed comparing preterm and full-term born infants.
Wroblewska-Seniuk, K.	2017	Hearing impairment in premature newborns-Analysis based on the national hearing screening database in Poland	No comparison of hearing impairment between preterm and full-term born infants.
Wroblewska-Seniuk, K.	2017	The results of newborn hearing screening by means of transient otoacoustic emissions - has anything changed over 10 years?	Only investigation of gestational age as risk factor for hearing impairment, other risk factors not analyzed comparing preterm and full-term born infants.
Wu, B. L.	2002	Effectiveness of sequencing connexin 26 (GJB2) in cases of familial or sporadic childhood deafness referred for molecular diagnostic testing	Gestational age of study population was not sufficiently stated.
Wu, C. Y.	1993	Neonatal hearing impairment complicated by neonatal pneumonia. Application of auditory evoked potentials in neonatology	Connection of risk factors and/or hearing outcome depending on gestational age was not established.
Wu, G. T.	2017	Is routine audiometric testing necessary for children with isolated preauricular lesions?	Gestational age of study population was not sufficiently stated.
Wu, H. M.	2011	Clinical features, acute complications, and outcome of Salmonella meningitis in children under one year of age in Taiwan	Gestational age of study population was not sufficiently stated.
Wu, J. J. Y.	2021	Retrospective comparison of death or neurodevelopmental outcomes in extremely low birth weight preterm infants following different management options of haemodynamically significant patent ductus arteriosus	No comparison of hearing impairment between preterm and full-term born infants.
Wu, M.	2020	4q27 deletion and 7q36.1 microduplication in a patient with multiple malformations and hearing loss: a case report	Case report as unsuitable study type.
Wu, N. A. L.	2022	Long-term neurocognitive and quality of life outcomes in survivors of pediatric hematopoietic cell transplant	Gestational age of study population was not sufficiently stated.
Wu, T. N.	1993	Comparison of blood pressure in deaf-mute children and children with normal hearing: association between noise and blood pressure	Gestational age of study population was not sufficiently stated.
Wu, W.	2020	[Clinical analysis of complications of suppurative otitis media in children]	No comparison of hearing impairment between preterm and full-term born infants.
Wu, X. H.	2020	A prospective observational study to investigate the correlation analysis between neonatal hyperbilirubinemia and deafness gene Study protocol clinical trial (SPIRIT compliant)	Study protocol as unsuitable study type.
Xiang, J. L.	2022	Utility of Whole Genome Sequencing for Population Screening of Deafness-Related Genetic Variants and Cytomegalovirus Infection in Newborns	Gestational age of study population was not sufficiently stated.
Xiao, C.	2021	Genetic etiology study of four Chinese families with two nonsyndromic deaf children in succession by targeted next-generation sequencing	Gestational age of study population was not sufficiently stated.
Xiao, T.	2015	Association between mode of delivery and failure of neonatal acoustic emission test: a retrospective analysis	No comparison of hearing impairment between preterm and full-term born infants.
Xing, G. Q.	2015	Identification of OSBPL2 as a novel candidate gene for progressive nonsyndromic hearing loss by whole-exome sequencing	Gestational age of study population was not sufficiently stated.
Xoinis, K.	2007	Extremely low birth weight infants are at high risk for auditory neuropathy	No comparison of hearing impairment between preterm and full-term born infants.
Xu, J.	2019	Relationship research between auditory neuropathy spectrum disorder and exchange transfusion in neonates with severe hyperbilirubinemia	Connection of risk factors and/or hearing outcome depending on gestational age was not established.
Xu, X.	2011	An epidemiologic study of tinnitus in a population in Jiangsu Province, China	Gestational age of study population was not sufficiently stated.
Yadav, S. S.	2010	Cytomegalovirus Infection in Six Neonates	Gestational age of study population was not sufficiently stated.
Yamada, H.	2020	A cohort study of the universal neonatal urine screening for congenital cytomegalovirus infection	Gestational age of study population was not sufficiently stated.
Yamaguchi, A.	2017	Screening for seemingly healthy newborns with congenital cytomegalovirus infection by quantitative real-time polymerase chain reaction using newborn urine: an observational study	Connection of risk factors and/or hearing outcome depending on gestational age was not established.

Yamamoto, A. Y.	2020	Contribution of Congenital Cytomegalovirus Infection to Permanent Hearing Loss in a Highly Seropositive Population: The Brazilian Cytomegalovirus Hearing and Maternal Secondary Infection Study	Connection of risk factors and/or hearing outcome depending on gestational age was not established.
Yamamoto, Y.	2016	Otological complications associated with hyperbaric oxygen therapy	Gestational age of study population was not sufficiently stated.
Yan, H.	2008	Genetic variations in the gB, UL144 and UL149 genes of human cytomegalovirus strains collected from congenitally and postnatally infected Japanese children	Gestational age of study population was not sufficiently stated.
Yan, Z.	2013	Nasopharyngeal carcinoma in children and adolescents in an endemic area: a report of 185 cases	Gestational age of study population was not sufficiently stated.
Yanagita, N.	1986	A comparative study of mumps deafness and idiopathic profound sudden deafness	Gestational age of study population was not sufficiently stated.
Yanagita, N.	1994	Estimated annual number of patients treated for sensorineural hearing loss in Japan. Results of a nationwide epidemiological survey in 1987	Gestational age of study population was not sufficiently stated.
Yancey, A.	2012	Risk factors for cisplatin-associated ototoxicity in pediatric oncology patients	Gestational age of study population was not sufficiently stated.
Yang, C. Q.	2021	Report of a case with ferredoxin reductase (FDXR) gene variants in a Chinese boy exhibiting hearing loss, visual impairment, and motor retardation	Case report as unsuitable study type.
Yang, E. Y.	1993	Auditory brain stem responses to air- and bone-conducted clicks in the audiological assessment of at-risk infants	Connection of risk factors and/or hearing outcome depending on gestational age was not established.
Yang, J. J.	2010	Prospective variants screening of connexin genes in children with hearing impairment: genotype/phenotype correlation	Gestational age of study population was not sufficiently stated.
Yang, L. L.	2022	Comparative Effects of Valganciclovir and Ganciclovir on the Congenital Cytomegalovirus Infection and Hearing Loss: A Randomized Controlled Trial	Connection of risk factors and/or hearing outcome depending on gestational age was not established.
Yang, M.	2022	A novel de novo missense mutation in EFTUD2 identified by whole-exome sequencing in mandibulofacial dysostosis with microcephaly	Case report as unsuitable study type.
Yang, S. M.	2018	Hearing-loss-associated gene detection in neonatal intensive care unit	Only investigation of prematurity as risk factor for hearing impairment, other risk factors not analyzed comparing preterm and full-term born infants.
Yang, T. H.	2021	The prevalence and demographic features of congenital cytomegalovirus infection in an urban area of East Asia: A population-based study	No comparison of hearing impairment between preterm and full-term born infants.
Yankey, H.	2018	Efficacy of topical 2% mupirocin ointment for treatment of tympanostomy tube otorrhea caused by community-acquired methicillin resistant Staphylococcus aureus	Gestational age of study population was not sufficiently stated.
Yano, S. T.	2017	Fever-Induced Paroxysmal Weakness and Encephalopathy, a New Phenotype of ATP1A3 Mutation	Case report as unsuitable study type.
Yao, Q. X.	2019	Benign paroxysmal positional vertigo in children	Gestational age of study population was not sufficiently stated.
Yasui, N.	2014	Cisplatin-induced Hearing Loss: The Need for a Long-term Evaluating System	Gestational age of study population was not sufficiently stated.
Yeat, S. W.	1997	Post meningitic sensori-neural hearing loss in children--alterations in hearing level	Gestational age of study population was not sufficiently stated.
Yee-Arellano, H. M.	2006	Universal newborn hearing screening in Mexico: Results of the first 2 years	Gestational age of study population was not sufficiently stated.
Yehudai, N.	2015	Risk factors for sensorineural hearing loss in pediatric chronic otitis media	Gestational age of study population was not sufficiently stated.
Yelverton, J. C.	2013	Risk Factors Associated With Unilateral Hearing Loss	Gestational age of study population was not sufficiently stated.
Yesilipek, A. M.	2012	Successful unrelated bone marrow transplantation in two siblings with alpha-mannosidosis	Case report as unsuitable study type.
Yiengprugsawan, V.	2013	Longitudinal analysis of ear infection and hearing impairment: findings from 6-year prospective cohorts of Australian children	Gestational age of study population was not sufficiently stated.
Yildirim, Z. Y.	2020	Primary coenzyme Q10 Deficiency-6 (COQ10D6): Two siblings with variable expressivity of the renal phenotype	Case report as unsuitable study type.
Yildiz, G.	2022	Hearing test results of newborns born from the coronavirus disease 2019 (COVID-19) infected mothers: A tertiary center experience in Turkey	Connection of risk factors and/or hearing outcome depending on gestational age was not established.
Yilmaz, F. H.	2021	Is there a relationship between causative microorganisms and hearing loss in neonatal sepsis?	Connection of risk factors and/or hearing outcome depending on gestational age was not established.

Yilmaz, Y. Z.	2022	The Relationship Between the Presence of Severe Acute Respiratory Syndrome-Coronavirus-2 during Pregnancy and Neonatal Hearing Loss	No comparison of hearing impairment between preterm and full-term born infants.
Yilmazer, R.	2016	Follow-Up Results of Newborns after Hearing Screening at a Training and Research Hospital in Turkey	Gestational age of study population was not sufficiently stated.
Yliherva, A.	2001	Linguistic and motor abilities of low-birthweight children as assessed by parents and teachers at 8 years of age	Connection of risk factors and/or hearing outcome depending on gestational age was not established.
Ylijoki, M.	2020	Neurodevelopmental outcome of preterm twins at 5 years of age	No comparison of hearing impairment between preterm and full-term born infants.
Yock, T. I.	2016	Long-term toxic effects of proton radiotherapy for paediatric medulloblastoma: a phase 2 single-arm study	Gestational age of study population was not sufficiently stated.
Yokota, T.	2013	Novel treatment strategy for Japanese newborns with high serum unbound bilirubin	No comparison of hearing impairment between preterm and full-term born infants.
Yoon, P. J.	2003	The need for long-term audiologic follow-up of neonatal intensive care unit (NICU) graduates	Case report as unsuitable study type.
Yoon, P. J.	2020	Novel Variants in Hearing Loss Genes and Associations With Audiometric Thresholds in a Multi-ethnic Cohort of US Patients With Cochlear Implants	Gestational age of study population was not sufficiently stated.
Yoong, S.	2005	Audit of local performance compared with standards recommended by the national guidelines for aetiologic investigation of permanent childhood hearing impairment	Gestational age of study population was not sufficiently stated.
Yoong, S. Y.	2005	Families affected by deafness: hospital services uptake in a multiethnic population	Gestational age of study population was not sufficiently stated.
Yorulmaz, A.	2017	Evaluation and Importance of Our Newborn Hearing Screening Results	Connection of risk factors and/or hearing outcome depending on gestational age was not established.
Yoshida, H.	2009	Cochlear Implantation in Children With Congenital Cytomegalovirus Infection	Gestational age of study population was not sufficiently stated.
Yoshida, H.	2017	Long-term Outcomes of Cochlear Implantation in Children With Congenital Cytomegalovirus Infection	Gestational age of study population was not sufficiently stated.
Yoshida, S.	2002	[Neonatal auditory screening with automated ABR]	No comparison of hearing impairment between preterm and full-term born infants.
Yoshida, S.	2018	Prenatal Alcohol Exposure and Suspected Hearing Impairment Among Children: A Population-based Retrospective Cohort Study	Only investigation of gestational age as risk factor for hearing impairment, other risk factors not analyzed comparing preterm and full-term born infants.
Yoshikawa, S.	2004	The effects of hypoxia, premature birth, infection, ototoxic drugs, circulatory system and congenital disease on neonatal hearing loss	Only investigation of gestational age as risk factor for hearing impairment, other risk factors not analyzed comparing preterm and full-term born infants.
Yoshimura, H.	2020	Genetic testing has the potential to impact hearing preservation following cochlear implantation	Gestational age of study population was not sufficiently stated.
Yoshinaga-Itano, C.	2017	Early Hearing Detection and Vocabulary of Children With Hearing Loss	Gestational age of study population was not sufficiently stated.
Young, N. M.	2000	Postmeningitic ossification in pediatric cochlear implantation	Gestational age of study population was not sufficiently stated.
Young, N. M.	2011	Limitations of Universal Newborn Hearing Screening in Early Identification of Pediatric Cochlear Implant Candidates	Gestational age of study population was not sufficiently stated.
Young, S. L.	2022	Novel biallelic USH2A variants in a patient with usher syndrome type IIA- a case report	Case report as unsuitable study type.
Young, W. Y.	2005	Extremely low penetrance of hearing loss in four Chinese families with the mitochondrial 12S rRNA A1555G mutation	Gestational age of study population was not sufficiently stated.
Yow, M. D.	1988	Epidemiologic characteristics of cytomegalovirus infection in mothers and their infants	Connection of risk factors and/or hearing outcome depending on gestational age was not established.
Yu, S.	2021	Apparent homozygosity for a novel splicing variant in EPS8 causes congenital profound hearing loss	Case report as unsuitable study type.
Yu, W. H.	2022	Early-life respiratory trajectories and neurodevelopmental outcomes in infants born very and extremely preterm: A retrospective study	No comparison of hearing impairment between preterm and full-term born infants.
Yuan, H. J.	2005	Cosegregation of the G7444A mutation in the mitochondrial COI/tRNA(Ser(UCN)) genes with the 12S rRNA A1555G mutation in a Chinese family with aminoglycoside-induced and nonsyndromic hearing loss	Gestational age of study population was not sufficiently stated.
Yucel, A.	2019	Newborn hearing screening results of refugees living in our city and the factors affecting the results	Only investigation of gestational age as risk factor for hearing impairment, other risk factors not analyzed comparing preterm and full-term born infants.
Yucel, H.	2022	Evaluation of neonatal hearing screening results of newborns with premature retinopathy	No comparison of hearing impairment between preterm and full-term born infants.

Yue, W. L.	1988	Otolaryngological problems associated with thrombocytopenia purpura in children	Gestational age of study population was not sufficiently stated.
Yuksel, F.	2021	Analysis of Newborn Hearing Screening Test Results of Children with Down Syndrome	No comparison of hearing impairment between preterm and full-term born infants.
Yuksel, F.	2013	Gastroesophageal Reflux Disease in Children With Chronic Otitis Media With Effusion	Gestational age of study population was not sufficiently stated.
Yun, C. F.	2017	Prevalence and Social Risk Factors for Hearing Impairment in Chinese Children-A National Survey	Gestational age of study population was not sufficiently stated.
Yun, Z. Y.	2016	[An analysis of various diseases and hearing screening in NICU infants]	Only investigation of prematurity as risk factor for hearing impairment, other risk factors not analyzed comparing preterm and full-term born infants.
Yunis, J. J.	1976	Clinical manifestations of mannosidosis--a longitudinal study	Gestational age of study population was not sufficiently stated.
Zafar, S.	2020	Novel Mutations in CLPP, LARS2, CDH23, and COL4A5 Identified in Familial Cases of Prelingual Hearing Loss	Gestational age of study population was not sufficiently stated.
Zagolski, O.	2007	Vestibular system in infants after systemic aminoglycoside therapy	Gestational age of study population was not sufficiently stated.
Zagolski, O.	2008	Vestibular-evoked myogenic potentials and caloric stimulation in infants with congenital cytomegalovirus infection	Gestational age of study population was not sufficiently stated.
Zagolski, O.	2009	Vestibular-evoked myogenic potentials and caloric tests in infants with congenital rubella	Gestational age of study population was not sufficiently stated.
Zagolski, O.	2006	Functional evaluation of the vestibular organ in infants with risk factors for hearing loss occurring in the perinatal period	No comparison of hearing impairment between preterm and full-term born infants.
Zahlanie, Y.	2019	Possible canine source of Streptococcus equi subspecies zooepidemicus causing meningitis in an infant	Case report as unsuitable study type.
Zajac-Ratajczak, I.	2016	Assesment effectiveness of treatment Eustachian tube dysfunction using pneumatic inhaler AMSA	Gestational age of study population was not sufficiently stated.
Zakzouk, S. M.	1996	Hearing impairment among "at risk' children	No comparison of hearing impairment between preterm and full-term born infants.
Zakzouk, S. M.	1996	Prevalence of sensorineural hearing loss due to rubella in Saudi children	Gestational age of study population was not sufficiently stated.
Zakzouk, S. M.	1996	Prevalence of severe to profound sensorineural hearing loss in children having family members with hearing impairment	Gestational age of study population was not sufficiently stated.
Zakzouk, S. M.	1994	Hearing impairment among children in Saudi Arabia: familial incidence and potential risk factors	No comparison of hearing impairment between preterm and full-term born infants.
Zakzouk, S. M.	1999	A survey of childhood hearing impairment	Gestational age of study population was not sufficiently stated.
Zang, Z.	2008	Distortion product otoacoustic emissions at 6 months in term infants after perinatal hypoxia-ischaemia or with a low Apgar score	No comparison of hearing impairment between preterm and full-term born infants.
Zaputovic, S.	2005	Molecular analysis in diagnostic procedure of hearing impairment in newborns	Gestational age of study population was not sufficiently stated.
Zargi, M.	1992	Effects of recurrent otitis media in infancy on auditory perception and speech	Gestational age of study population was not sufficiently stated.
Zatz, M.	2012	Assessing pathogenicity for novel mutation/sequence variants: the value of healthy older individuals	Gestational age of study population was not sufficiently stated.
Zavattoni, M.	2014	Maternal, Fetal, and Neonatal Parameters for Prognosis and Counseling of HCMV Congenital Infection	Connection of risk factors and/or hearing outcome depending on gestational age was not established.
Zawawi, F.	2022	Otolaryngology Manifestations of Primary Ciliary Dyskinesia: A Multicenter Study	Gestational age of study population was not sufficiently stated.
Zawawi, F.	2018	Emberger syndrome: A rare association with hearing loss	Case report as unsuitable study type.
Zeeshan, F.	2018	Hearing impairment after acute bacterial meningitis in children	Gestational age of study population was not sufficiently stated.
Zeng, X.	2020	Combined hearing screening and genetic screening of deafness among Hakka newborns in China	Connection of risk factors and/or hearing outcome depending on gestational age was not established.
Zeytinoglu, A.	2019	Investigation of Congenital CMV Infection with the Presence of CMV DNA in Saliva Samples of New Born Babies	Connection of risk factors and/or hearing outcome depending on gestational age was not established.
Zhai, F.	2021	Risk Factors for Failure in First-Time Hearing Screening Tests among High-Risk Neonates in Neonatal Intensive Care Unit	Only investigation of prematurity as risk factor for hearing impairment, other risk factors not analyzed comparing preterm and full-term born infants.

Zhai, R.	2020	Auditory Neuropathy Spectrum Disorder (ANSD)-Clinical Characteristics and Pathogenic Variant Analysis of Three Nonsyndromic Deafness Families	Gestational age of study population was not sufficiently stated.
Zhang, C.	2021	Mutation analysis of TCOF1 gene in Chinese Treacher Collins syndrome patients	Gestational age of study population was not sufficiently stated.
Zhang, F.	2016	Identification of a novel mutation in SLC26A4 gene in a Chinese family with enlarged vestibular aqueduct syndrome	Gestational age of study population was not sufficiently stated.
Zhang, H.	2006	Sampling survey of disability in 0-6 year-old children in China	Gestational age of study population was not sufficiently stated.
Zhang, J.	2019	Infantile epilepsy with multifocal myoclonus caused by TBC1D24 mutations	Gestational age of study population was not sufficiently stated.
Zhang, J.	2013	Newborn hearing concurrent genetic screening for hearing impairment-a clinical practice in 58,397 neonates in Tianjin, China	Connection of risk factors and/or hearing outcome depending on gestational age was not established.
Zhang, L.	1992	Development of the brainstem auditory pathway in low birthweight and perinatally asphyxiated children with neurological sequelae	No comparison of hearing impairment between preterm and full-term born infants.
Zhang, M. J.	2016	[Risk factors for hearing impairment induced by cytomegalovirus infection]	No comparison of hearing impairment between preterm and full-term born infants.
Zhang, Q.	2016	[Neurodevelopmental outcomes of extremely low birth weight and very low birth weight infants and related influencing factors]	No comparison of hearing impairment between preterm and full-term born infants.
Zhang, Q. J.	2016	High frequency of OTOF mutations in Chinese infants with congenital auditory neuropathy spectrum disorder	Connection of risk factors and/or hearing outcome depending on gestational age was not established.
Zhang, R.	2022	Outcome Analysis of Severe Hyperbilirubinemia in Neonates Undergoing Exchange Transfusion	No comparison of hearing impairment between preterm and full-term born infants.
Zhang, X.	2019	The value of ABR- and ASSR-based hearing estimation in young children with congenital monaural malformation (atresia)	Gestational age of study population was not sufficiently stated.
Zhang, Y.	2019	Mutations in both SAMD9 and SLC19A2 genes caused complex phenotypes characterized by recurrent infection, dysphagia and profound deafness - a case report for dual diagnosis	Case report as unsuitable study type.
Zhang, Y. M.	2020	The combined novel KCNQ1 frameshift I145Sfs*92 and nonsense W392X variants caused Jervell and Lange-Nielsen syndrome in a Chinese infant presenting with sustained foetal bradycardia	Case report as unsuitable study type.
Zhao, H.	2004	Maternally inherited aminoglycoside-induced and nonsyndromic deafness is associated with the novel C1494T mutation in the mitochondrial 12S rRNA gene in a large chinese family	Gestational age of study population was not sufficiently stated.
Zhao, X.	2019	Genotyping and audiological characteristics of infants with a single-allele SLC26A4 mutation	Gestational age of study population was not sufficiently stated.
Zhao, X. L.	2018	[Analysis of clinical audiology and etiology in 72 twins aged 0-4 years]	No comparison of hearing impairment between preterm and full-term born infants.
Zhou, G.	2015	Objective vestibular testing of children with dizziness and balance complaints following sports-related concussions	Gestational age of study population was not sufficiently stated.
Zhou, G.	2018	Total serum bilirubin levels and sensorineural hearing loss in the US adolescents: NHANES 2007-2010	Gestational age of study population was not sufficiently stated.
Zhou, Y. J.	2017	The potential dysfunction of otolith organs in patients after mumps infection	Gestational age of study population was not sufficiently stated.
Zhu, Q. W.	2021	Assessment of Hearing Screening Combined With Limited and Expanded Genetic Screening for Newborns in Nantong, China	Connection of risk factors and/or hearing outcome depending on gestational age was not established.
Zhu, Y. J.	2012	Cryptococcal meningitis in immunocompetent children	Gestational age of study population was not sufficiently stated.
Zhu, Y. Q.	2022	Association Between Expanded Genomic Sequencing Combined With Hearing Screening and Detection of Hearing Loss Among Newborns in a Neonatal Intensive Care Unit	Connection of risk factors and/or hearing outcome depending on gestational age was not established.
Zorowka, P.	1993	Serial measurements of transient evoked otoacoustic emissions (TEOAEs) in healthy newborns and in newborns with perinatal infection	No comparison of hearing impairment between preterm and full-term born infants.
Zumach, A.	2009	Otitis media and speech-in-noise recognition in school-aged children	Gestational age of study population was not sufficiently stated.
Zuo, K. J.	2016	Clinical Outcomes of Osseointegrated Prosthetic Auricular Reconstruction in Patients With a Compromised Ipsilateral Temporoparietal Fascial Flap	Gestational age of study population was not sufficiently stated.
Zych, M.	2018	The report of the Polish Universal Neonatal Hearing Screening Program in 2016	Gestational age of study population was not sufficiently stated.

*	2013	Encouraging outcomes for Sweden's extremely preterm babies	Hearing outcome not examined.

Figure S1. Diagnostic Algorithm for Early Detection and Monitoring of Hearing Impairment in Preterm and High-Risk Infants

This diagnostic algorithm is designed for research use in preterm and high-risk infants (e.g., NICU admission, exposure to ototoxic medication, ventilation, recurrent infections). It supports early detection and structured long-term monitoring of hearing impairment, especially in children with elevated risk for CHL, OME, or APD. It is not intended as a universal screening pathway for all children.

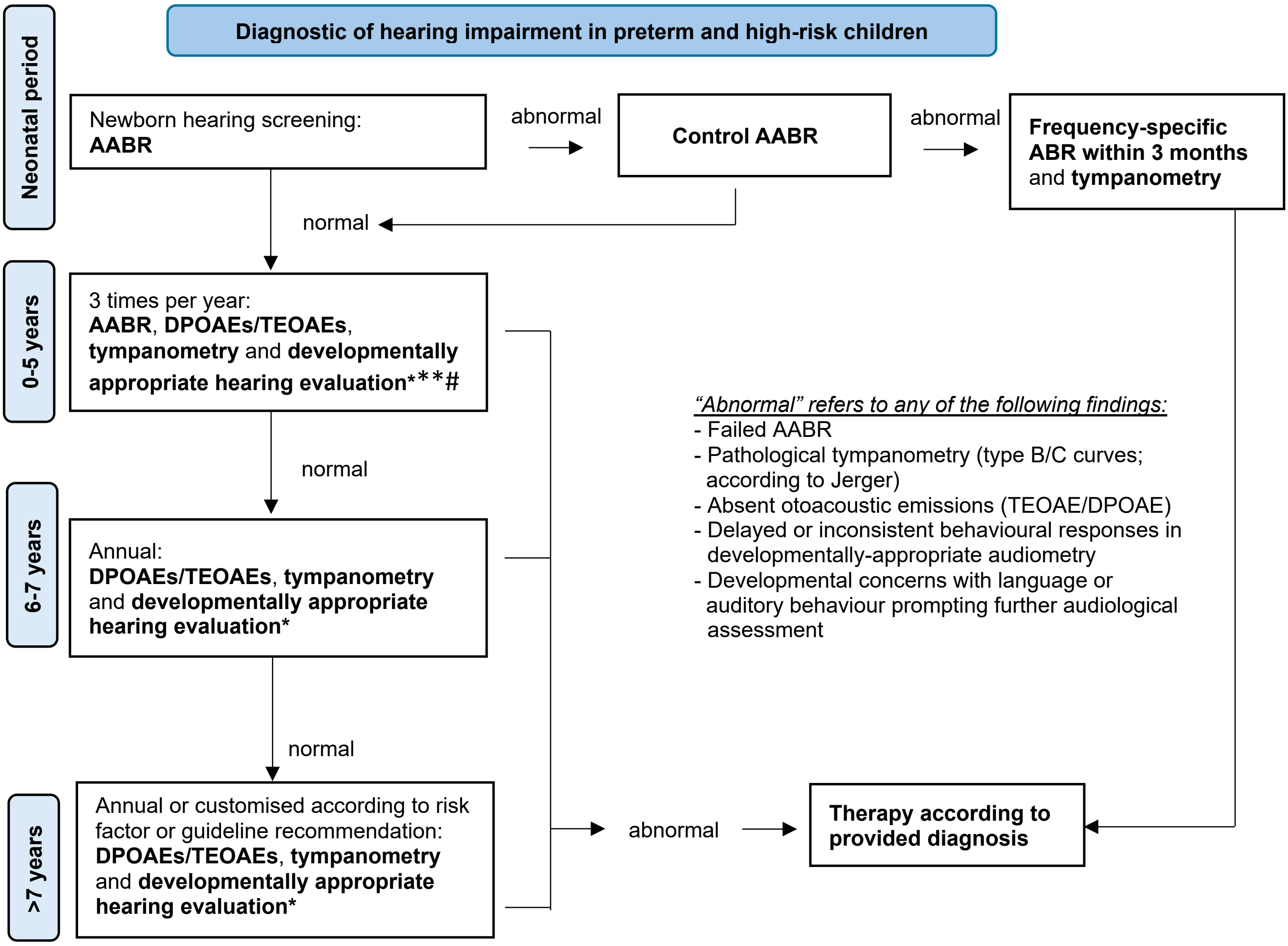

* ***Developmentally appropriate hearing assessment methods:***

- for every age group: wideband tympanometry, stapedius reflex, otoacoustic emissions (TEOAE/ DPOAE) and AABR.
  - if AABR is abnormal and in a healthy middle ear, proceeding with ABR (Click, Chirp or MUSIC module) for further evaluation is recommended as long as only behavioural response thresholds can be obtained and true hearing thresholds are not yet measurable (due to developmental state)
  - ear microscopy or videootoscopy (if required)
- 3-6 months: behavioural observation audiometry/ distraction audiometry
  - MMN (Mismatch Negativity) to detect auditory discrimination (applicable from ~6 months)
- 6 months - 2.5 years: Visual Reinforcement Audiometry (VRA)
  - P300/LAEP to detect cognitive processing of auditory input (applicable from ~3 years)
- 2.5 - 5 years: conditioned response audiometry
  - CERA to detect central auditory maturation (usable from infancy)
- 3 years onwards: Pure-tone audiometry
- 7 years onwards: Auditory Processing Disorder using standardized and language-dependent tests. Objective, language-independent diagnostics provide earlier insight into central auditory development. Cortical and cognitive auditory evoked potentials (MMN, P300 and CERA) are considered to support early, language-independent assessment of auditory processing, especially in multilingual or language-impaired children.
- From infancy onward, objective electrophysiological tests (e.g., CERA) may be helpful, especially in children with neurological risk factors or when behavioral testing is inconclusive.

In children with persistent OME or CHL, classification into treatment pathways (watchful waiting, conservative, or surgical therapy) should be documented and monitored over time.
Regular follow-up is recommended up to age 10 in children at risk (e.g., preterm, recurrent OME, CHL, APD).
